# Tunable *Endo*/*Exo* Selectivity in Direct Catalytic Asymmetric 1,3‐Dipolar Cycloadditions with Polyfunctional Lewis Acid / Azolium–Aryloxide Catalysts

**DOI:** 10.1002/anie.202508024

**Published:** 2025-07-07

**Authors:** Adrian Bürstner, Patrick M. Becker, Alexander Allgaier, Lucca Pfitzer, Daniel M. Wanner, Johanna Dollinger, Felix Willig, Justin Herrmann, Vukoslava Miskov‐Pajic, Andreas C. Hans, Wolfgang Frey, Joris van Slageren, Johannes Kästner, René Peters

**Affiliations:** ^1^ Universität Stuttgart, Institut für Organische Chemie Pfaffenwaldring 55 D‐70569 Stuttgart Germany; ^2^ Universität Stuttgart, Institut für Theoretische Chemie Pfaffenwaldring 55 D‐70569 Stuttgart Germany; ^3^ Universität Stuttgart, Institut für Physikalische Chemie Pfaffenwaldring 55 D‐70569 Stuttgart Germany

**Keywords:** 3‐dipolar cycloadditions, Asymmetric catalysis, DFT, Polyfunctional catalysis, Pyrrolidines

## Abstract

Catalytic asymmetric 1,3‐dipolar cycloadditions (1,3‐DCA) using iminoesters as ylide precursors offer a powerful approach to accessing stereochemically complex, biologically relevant pyrrolidines. Although previous studies have already achieved impressive stereoselectivities, catalytic productivity remains a challenge, with turnover numbers (TON) typically below 20. In this article, we introduce a novel concept for catalytic 1,3‐DCA that enables remarkable productivity for both *endo* (TON up to 4000) and the more challenging *exo* products (TON up to 1500). This approach, making use of modular polyfunctional Lewis acid/azolium‐aryloxide catalysts, allows for precise control over *endo*‐ and *exo*‐diastereoselectivity. The switch from *endo*‐ to *exo*‐selectivity is accomplished by modifying the metal center, the azolium moiety, and steric factors. As detailed DFT studies reveal, both the *endo*‐ and *exo*‐selective catalyst systems exhibit an almost perfect spatial alignment of their key functional sites, allowing for a unique interplay of Brønsted acids and bases, Lewis acids, and hydrogen bonding. The computational studies further demonstrate that these polyfunctional catalysts dramatically lower the energetic barriers of the concerted or stepwise cycloaddition key steps. However, they also precisely orchestrate and accelerate all accompanying transformations—reminiscent of enzymatic machineries.

## Graphical abstract

A novel concept for catalytic asymmetric 1,3‐dipolar cycloadditions with iminoesters employs modular polyfunctional Lewis acid/azolium‐aryloxide betaine catalysts, controlling endo‐ and exo‐selectivity through adjustments of metal center, azolium, and sterics. DFT studies reveal an almost perfect spatial fit of functional sites, enabling precise orchestration of the catalytically relevant moieties involved—reminiscent of enzymatic catalysis.

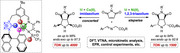

## Introduction

**Scheme 1:**
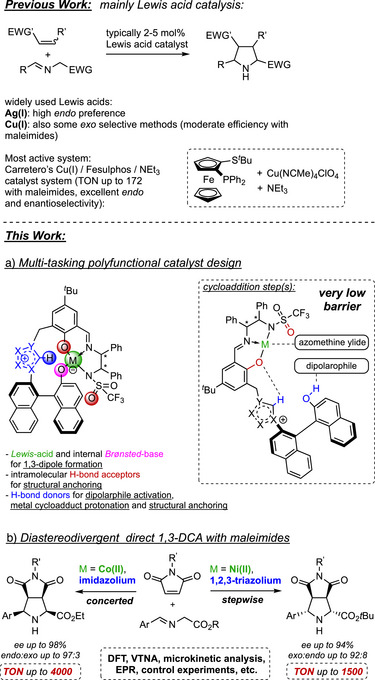
State‐of‐the‐art of 1,3‐DCA, the new concept and use.

1,3‐Dipolar cycloadditions provide a powerful strategy for the construction of stereochemically complex, highly functionalized 5‐membered rings containing heteroatoms.^[^ ^1^, ^2^ ^]^ Pyrrolidines are among the most important nitrogen‐containing heterocycles. They are found in a huge number of bioactive natural products^[^ ^3^, ^4^, ^5^, ^6^, ^7^ ^]^ and are essential as chiral key motifs in a large number of active pharmaceutical ingredients,^[^ ^8^ ^]^ which are prescribed, e.g., as antibacterial, antifungal, and tumor‐therapeutic agents.^[^ ^9^, ^10^, ^11^, ^12^, ^13^ ^]^ This explains the demand for efficient stereoselective methods for the preparation of pyrrolidines. A large number of catalytic asymmetric 1,3‐DCA of azomethine ylides and electron‐poor olefins has been developed to provide a straightforward, elegant access. Various Lewis acid catalysts have been reported to allow for high stereocontrol despite the fact that up to 16 stereoisomers might, in principle, be formed.^[^ ^9^, ^10^, ^11^, ^12^, ^13^ ^]^ Silver^[^ ^19^, ^20^, ^21^ ^]^ and copper^[^ ^22^, ^23^, ^24^, ^25^ ^]^ catalysts, in particular Carretero's Cu(I) Fesulphos, are well‐established and frequently used (Scheme 1, *top*).^[^ ^14^, ^15^, ^16^, ^17^, ^18^ ^]^ Other central metals have been occasionally reported.^[^ ^26^, ^27^, ^28^, ^29^, ^30^, ^31^, ^32^, ^33^ ^]^

As pointed out in a recent review by Adrio and Carretero, most of the best 1,3‐DCA methods using a specific type of dipolarophile manage the efficient synthesis of either *endo* or *exo* isomers, whereas stereodivergent approaches providing different diastereomers on demand are quite rare.^[^ ^14^, ^34^, ^35^, ^36^, ^37^ ^]^

Using maleimides as dipolarophile, reported catalyst loadings are typically 2–10 mol% to form almost exclusively *endo* products.^[^ ^28^, ^29^, ^31^, ^38^, ^39^, ^40^, ^41^, ^42^, ^43^, ^44^, ^45^, ^46^, ^47^, ^48^, ^49^, ^50^, ^51^, ^52^, ^53^ ^]^ Carretero's Cu(I)/Fesulphos constitutes an important exception (Scheme 1, *top*), as catalyst loadings of 0.5 mol% were proficient to allow for TON up to 172.^[^ ^24^ ^]^ Synthesizing *exo* diastereomers with a combination of high yields, TONs, enantio‐ and diastereoselectivity is still a challenge.

Herein, we report a new concept for 1,3‐DCA, utilizing an intramolecular synergy of a Lewis acid and an azolium–aryloxide moiety (Scheme 1, *middle*). By this, diastereodivergence and remarkable productivity were attained with azomethine ylides and maleimides (Scheme 1, *bottom*). Depending on the choice of

– the metal center: Co(II) versus Ni(II)
– the azolium unit: imidazolium versus 1,2,3‐triazolium
– the azomethine ylide's ester unit

both the *endo* and *exo* diastereomers are selectively accessible in high yields using remarkably low catalyst loadings. TON up to 4000 (*endo*) and 1500 (*exo*) were realized. Control experiments, kinetic, spectroscopic, and very detailed DFT studies revealed that the cooperative interplay between Lewis acid and the azolium–aryloxide moiety is crucial for achieving high activities and selectivities. Computation revealed that the different catalyst functions allow for an enzyme‐related mode of action in terms of their necessity and interplay in various individual steps. They can precisely explain the *endo*‐ and *exo*‐selective outcomes by making use of the same catalyst concept, yet employing different catalyst modules. They can also explain the mechanistic differences and why different individual steps or sequences are limiting. As a common feature, both catalysts allow for very low barriers in C─C bond formation, thus demonstrating the powerful polyfunctional activation of the cycloaddition itself. Moreover, like in enzymes, the entropic part of the overall activation barrier is tiny.

## Results and Discussion

### Development and Optimization Studies

The investigation started with our previously reported Cu(II)/imidazolium‐naphthoxide catalyst **C1** (Table 1),^[^ ^54^, ^55^, ^56^, ^57^, ^58^ ^]^ using the 1,3‐DCA of glycine iminoethylester **1a** with *N*‐methylmaleimide **2A** as a model reaction. With 5 mol% catalyst, pyrrolidine **3aA** was formed in nearly quantitative yield and with good enantioselectivity, yet low *endo*‐selectivity (entry 1). With the new Zn(II), Ni(II), and Co(II) complexes **C2**–**C4**, which were readily prepared in few steps with high yields in close analogy to **C1** (see Supporting Information), yields were again very high and stereoselectivity was significantly improved (entries 2–4). Co(II) complex **C4** provided the highest diastereoselectivity and was selected for further studies.^[^ ^59^ ^]^ Its diastereomer **C5** showed a mismatched behavior (entry 5), favoring the other enantiomer. The loading of **C4** could be largely decreased, still allowing for high stereoselectivity (entries 6–7). With just 0.025 mol% of **C4** at 40 °C, **3aA** was formed in quantitative yield (TON 4000), while high stereoselectivity was still accomplished (entry 7).

**Table 1:** Development of the *endo*‐selective title reaction.

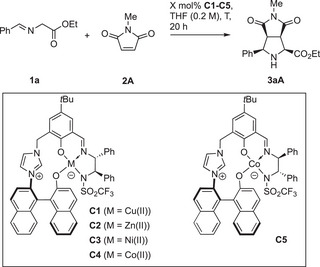									
	**C**	M	X (mol%)	T / (°C)	Conv.^a)^ / (%)	Yield^a)^ / (%)	*endo*: *exo* ^a)^	*ee* ^b)^ (%)	TON
1	**C1**	Cu(II)	5	25	99	99	77:23	90	20
2	**C2**	Zn(II)	5	25	>99	>99	92:8	96	20
3	**C3**	Ni(II)	5	25	>99	97	91:9	99	19
4	**C4**	Co(II)	5	25	>99	99	94:6	95	20
5	**C5**	Co(II)	5	25	>99	>99	78:22	−85	20
6	**C4**	Co(II)	0.1	25	99	99	94:6	98	990
7	**C4**	Co(II)	0.025	40	>99	>99	93:7	97	4000

^a)^ Determined by ^1^H‐NMR in the presence of an internal standard.

^b)^ Enantiomeric excess of the *endo* isomer determined by HPLC. A negative value indicates the major formation of the optical antipode of the one depicted.

During the catalyst screening, different alkoxy residues in the iminoesters were tested. It was found that Ni(II) catalyst **C3**, which allowed for good *endo* selectivity with ethylester substrate **1a** (Table 1, entry 3), permitted a low *exo*‐preference with *tert*.‐butyl ester **1b** (Table 2, entry 1).

**Table 2:** Development of the *exo*‐selective title reaction.

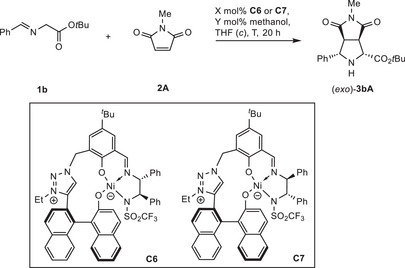										
	**C**	X (mol%)	Y (mol%)	*c* / (M)	*T* / (°C)	Conv.^a)^/(%)	Yield^a)^ /(%)	*endo*: *exo* ^a)^	*ee* (%)^b)^	TON
1	**C3**	5	−	0.2	25	95	86	30:70	−66	17
2	**C6**	5	−	0.2	25	98	88	11:89	−72	18
3	**C7**	5	−	0.2	25	94	74	20:80	90	15
4	**C7**	5	−	0.5	0	93	86	11:89	93	17
5	**C7**	5	−	1.0	0	82	80	8:92	93	16
6	**C7**	0.05	−	2.0	10	85	75	13:87	91	1500
7	**C7**	0.05	50	2.0	10	82	75	11:89	92	1500

^a)^ Determined by ^1^H‐NMR in the presence of an internal standard.

^b)^ Enantiomeric excess of the *exo* isomer determined by HPLC. Negative values indicate the major formation of the optical antipode of the one depicted.

With 1,2,3‐triazolium catalysts **C6** and **C7** the *exo* selectivity and enantioselectivity were further improved.^[^ ^60^, ^61^, ^62^, ^63^, ^64^, ^65^ ^]^ **C6** gave higher *exo* selectivity, but a moderate *ee* (entry 2), while its diastereomer **C7** allowed for high *ee*, but moderate *exo* preference (entry 3).

However, the highest *exo*‐ and enantioselectivity could be obtained with **C7** at 0 °C at higher concentration (*exo*:*endo* = 92:8, *ee* = 93%, entry 5). Gratifyingly, also for the challenging *exo*‐isomer synthesis, low catalyst loadings can be applied. A TON of 1500 was attained with 0.05 mol% catalyst at 10 °C (entry 6). Upon addition of 50 mol% of methanol, the stereoselectivity could be slightly improved (*exo*:*endo* = 89:11, *ee* = 92%, entry 7).^[^ ^66^ ^]^

### Reaction Scope

The generality of both the *endo*‐ and *exo*‐selective approaches was studied using different iminoester and maleimide derivatives (0.2 mmol). With Co(II) catalyst **C4**, the reaction conditions of Table 1, entry 7 were used, but adapting catalyst loadings to substrate reactivities. Different loadings were investigated, and the in our eyes most attractive compromise of loading and reaction outcome is listed in Table 3. Methyl, ethyl, and benzyl esters were compared and provided almost identical results (entries 1–3). Apparently, the electronic influence of different substituents R^2^ on arylimino moiety is relatively small, as all investigated σ‐ and π‐donors and σ‐ and π‐acceptors in the *para*‐position were well accommodated (entries 4, 5, 6, and 9, respectively). Substitution at *meta*‐ and *ortho*‐position was studied for a chloro substituent and led to similar results as *para*‐substitution (entries 7–8).^[^ ^67^, ^68^ ^]^

**Table 3:** Study of the reaction scope for the *endo*‐selective Co(II) catalysis.

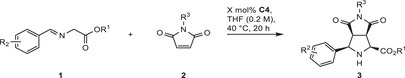										
	**3**	**1**	R^1^	R^2^	**2**	R^3^	X / (mol%)	Yield^a)^ / (%)	*endo* :*exo* ^b)^	*ee* ^c)^ (%)
1	**aA**	**a**	Et	H	**A**	Me	0.05	84	93:7	97
1‘^d)^	**aA**	**a**	Et	H	**A**	Me	0.1	76	93:7	98
2	**cA**	**c**	Me	H	**A**	Me	0.05	83	93:7	94
3	**dA**	**d**	Bn	H	**A**	Me	0.05	82	95:5	95
4	**eA**	**e**	Et	4‐OMe	**A**	Me	0.1	83	94:6	94
5	**fA**	**f**	Et	4‐Me	**A**	Me	0.1	99	93:7	96
6	**gA**	**g**	Et	4‐Cl	**A**	Me	0.025	98	92:8	93
7	**hA**	**h**	Et	3‐Cl	**A**	Me	0.05	>99	92:8	90
8	**iA**	**i**	Et	2‐Cl	**A**	Me	0.2	>99	97:3	93
9	**jA**	**j**	Et	4‐NO_2_	**A**	Me	0.1	97	88:12	75
10	**aB**	**a**	Et	H	**B**	H	0.3	98	94:6	94
11	**aC**	**a**	Et	H	**C**	Ph	0.025	86	93:7	86
12	**aD**	**a**	Et	H	**D**	Bn	0.1	97	92:8	95

^a)^ Yield of isolated product after column chromatography.

^b)^ Determined by ^1^H‐NMR from the crude product.

^c)^ Enantiomeric excess of the *endo* isomer determined by HPLC.

^d)^ Scale up to 2.0 mmol.

A notable feature is the tolerance of unprotected maleimide substrate **2B** (R^3^ = H), still providing high yield and stereoselectivity with a **C4** loading of 0.3 mol%. With an N‐Ph residue R^3^, very high productivity was found, albeit the *ee* was decreased to 86%, whereas with R^3^ = benzyl as a removable protective group, a high *ee* value was accomplished.

With Ni(II) catalyst **C7**, the reaction conditions of Table 2, entry 7 were employed, using 0.2–1 mol% as catalyst loading (Table 4). The dependence on substituent R^2^ (entries 2–5) does not apparently follow an electronic tendency regarding productivity. In all cases, good to high yields (75%–96%) and *ee* values (87%–94%) were attained. N‐Ph and N‐Bn were also well tolerated (entries 6 and 7), whereas the unprotected maleimide **2B** was unreactive (not shown). In general, the *exo*‐isomer was formed as the major product with moderate (entries 2 and 4) to good (entries 1, 3, 6, and 7) *dr* values, whereas with R^2^ = NO_2_, the *dr* was low (entry 5).

**Table 4:** Study of the reaction scope for the *exo*‐selective Ni(II) catalysis.

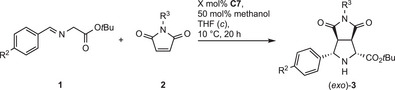									

	(*exo*)‐3	**1**	R^2^	**2**	R^3^	X / (mol%)	Yield^a)^ / (%)	*endo*:*exo* ^b)^	*ee* ^c)^ (%)
1	**bA**	**b**	H	**A**	Me	0.5	95	11:89	94
1‘	**bA**	**b**	H	**A**	Me	0.2	87	13:87	93
2	**kA**	**k**	OMe	**A**	Me	2.0	93	20:80	90
3	**lA**	**l**	Cl	**A**	Me	0.5	96	8:92	91
4	**mA**	**m**	Me	**A**	Me	0.5	92	18:82	89
5	**nA**	**n**	NO_2_	**A**	Me	1.0	75	31:69	87
6	**bC**	**b**	H	**C**	Ph	1.0	87	10:90	91
7	**bD**	**b**	H	**D**	Bn	1.0	>99	9:91	91

^a)^ Yield of isolated product after column chromatography.

^b)^ Determined by ^1^H‐NMR from the crude product.

^c)^ Enantiomeric excess of the *exo* isomer determined by HPLC.

^d)^ Scale up: 3.0 mmol **2A**.

### Catalyst Recycling

For recyclability studies, catalysts **C4** and **C7** were separated after 20 h from the reaction mixtures by filtration over silica gel. Silica protonates the active catalysts to give the precatalysts, which stick on silica. After elution of product, active catalysts are regenerated by 1 vol% *i*Pr_2_NEt (**C4**) or Et_3_N in THF (**C7**). For **C4**, it was found that the very high yields and stereoselectivities were maintained for at least four cycles (Table 5, entries 1–4). For **C7**, yields decreased from 91% to 77% after 4 runs, but high stereoselectivity was maintained (entries 5–8).

**Table 5:** Catalyst recycling.

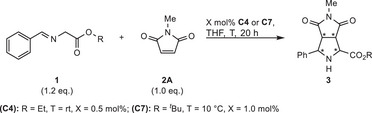				
Run	**C**	Yield^a)^ / (%)	*endo* :*exo* ^b)^	*ee* Major diastereomer^c)^ (%)
1	**C4**	>99	93:7	97
2	**C4**	>99	93:7	96
3	**C4**	>99	93:7	96
4	**C4**	>99	93:7	96
5	**C7**	91	10:90	94
6	**C7**	87	10:90	93
7	**C7**	89	13:87	93
8	**C7**	77	9:91	93

^a)^ Yield of isolated product after column chromatography.

^b)^ Determined by ^1^H‐NMR from the crude product.

^c)^ Enantiomeric excess of the *endo* isomer determined by HPLC.

### Mechanistic Investigations

#### Control Experiments

To learn which architecture and functional groups in the polyfunctional systems are essential for their performance, a number of control catalyst systems **CC** was investigated. For high *endo* selectivity the imidazolium moiety in **C4** is advantageous compared to a 1,2,3‐triazolium moiety in the control catalyst **CC1** (Table 6, entry 1). Azolium rings are beneficial for high enantioselectivity, as judged from **CC2** missing this feature, which gives nearly racemic product (entry 2). Furthermore, the axially chiral betaine element provides activity advantages compared to the simpler imidazolium phenolate in **CC3**. While good results were attained with 5 mol% of **CC3** (entry 3), no product was detected with 0.1 mol% (entry 4). This is in strong contrast to the use of **C4** (see Table 1, entries 6–7). Like expected, imidazolium catalyst **CC4** lacking the basic aryloxide showed a further decrease in activity (Table 6, entries 5 and 6).^[^ ^69^, ^70^, ^71^, ^72^, ^73^, ^74^, ^75^ ^]^

**Table 6:** Study of control catalysts **CC** for the *endo*‐selective Co(II) catalysis.

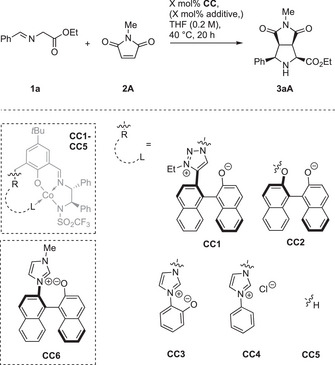							
	X / (mol%)	**CC**	additive	conv.^a)^ / (%)	yield^a)^ / (%)	*endo*: *exo* ^a)^	ee^b)^ / (%)
1	5	**CC1**	−	99	89	84:16	92
2	5	**CC2**	−	87	77	89:11	14
3	5	**CC3**	−	90	90	97:3	80
4	0.1	**CC3**	−	<2	<2	−	−
5	5	**CC4**	−	63	57	95:5	84
6	0.1	**CC4**	−	<2	<2	−	−
7	5	**CC5**	−	99	54	85:15	1
8	5	**CC5**	Cs_2_CO_3_	>99	11	76:24	74
9	5	**CC5 **+** CC6**	−	>99	71	67:33	82

^a)^ Determined by ^1^H‐NMR in the presence of an internal standard.

^b)^ Enantiomeric excess of the *endo* isomer determined by HPLC.

The simple complex **CC5** also displayed catalytic activity at high loadings, but gave nearly racemic product (entry 7). In the presence of Cs_2_CO_3_ as an external base the formation of large side product quantities was found, resulting in a poor yield, while enantioselectivity was largely improved (entry 8). Combining **CC5** with betaine fragment **CC6** in a binary catalyst system resulted in decreased *endo* selectivity and also considerable side product formation. These results indicate that an intramolecular interplay of the various functional groups is essential for the high performance of **C4**.

A similar program was performed for the *exo*‐selective reaction (Table 7). Surprisingly, catalyst **CC8** featuring an N‐mesyl ligand donor provided mainly *endo* product (entry 1), revealing the impact of the N‐triflyl donor in **C7**. We also investigated phenolate **CC9** (entry 2), lacking the element of axial chirality. Compared to the standard system **C7**, it is less active and does not favor the formation of the *exo* isomer. The binaphthyl architecture thus seems to help to accomplish a suitable structure to overwrite the inherent *endo* preference. Also, the aryloxide metal distance seems to be crucial, because for biphenolate **CC10**, which is structurally more similar to **C7**, we noticed *exo*‐selectivity (entry 3), yet still much lower than for **C7**.

**Table 7:** Study of control catalysts **CC** for the *exo*‐selective Ni(II) catalysis.

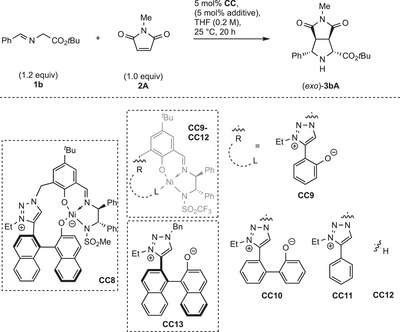						
	**CC**	Additive	Conv.^a)^ / (%)	Yield^a)^ / (%)	*exo*: *endo* ^a)^	*ee exo* ^b)^ / (%)
1	**CC8**	−	97	97	31:69	82
2	**CC9**	−	86	76	46:54	83
3	**CC10**	−	99	98	77:23	78
4	**CC11**	−	0	0	−	−
5	**CC12**	−	0	0	−	−
6	**CC12**	Cs_2_CO_3_	0	0	−	−
7	**CC12 **+ **CC13**	−	36	11	22:78	11

^a)^ Determined by ^1^H‐NMR in the presence of an internal standard.

^b)^ Enantiomeric excesses determined by HPLC.

Interestingly, the catalysts **CC11** and **CC12,** lacking an aryloxide, showed no catalytic activity (entries 4–6). In contrast, the binary catalyst system of **CC12** and **CC13** gave a small amount of product (entry 7) with poor stereoselectivity.

Table 7 thus suggests that for the *exo*‐selective synthesis, the sophisticated intramolecular interplay between the Ni center and the triazolium aryloxide with a precisely elaborated chiral environment is crucial for high performance.

#### Spectroscopic Studies

The properties of **C4** and **C7** were studied by paramagnetic Evans‐NMR, SQUID (superconducting quantum interference device) magnetometry, and EPR to gain insight into the electronic structure of the catalyst in a solid state as well as in solution. SQUID measurements of the molar magnetic susceptibility χ_m_ of both **C4** and its precatalyst (featuring the neutral naphthol unit) were performed on powder samples. Both samples show a constant χ_m_ *T* of around 2.3 cm^3 ^mol^−1 ^K at high temperatures and a strong decrease below 100 K (Figures S25 and S26).

Simulations of the susceptibility‐temperature product χ_m_ *T* for both compounds using the spin Hamiltonian formalism reveals a high‐spin state (*S *= 3/2) with zero‐field splitting (ZFS) parameters of *D* = +38 cm^−1^ and *E/D = *0.30(3).^[^ ^76^ ^]^ The slight difference in χ_m_ *T* at room temperature between **C4** and its precatalyst is due to different *g*_iso_ in **C4** (*g*_iso _= 2.22(1)) and its precatalyst (*g*_iso _= 2.28(1)). Furthermore, Evans method NMR measurements on **C4** and the precatalyst were performed in THF.^[^ ^77^, ^78^ ^]^ The paramagnetic shift confirms the high‐spin state (*S *= 3/2), but *g*_iso_ is slightly lower. EPR experiments using frozen solutions confirm these trends and are shown in the Supporting Information (Figure S28).

SQUID measurements on **C7** and its precatalyst (again featuring the neutral naphthol unit) show low χ_m_ *T* of 0.07 cm^3^ mol^−1 ^K across the whole temperature range with a slight increase above 270 K to 0.2 cm^3 ^mol^−1 ^K at 300 K for **C7**. For a Ni *d* ^8^‐system in a high spin configuration (*S* = 1) χ_m_ *T* = 1 cm^3^ mol^−1 ^K (assuming *g*_iso_ = 2) and for low spin (S = 0) χ_m_ *T* = 0 cm^3 ^mol^−1 ^K would be expected. These results indicate that **C7** does not exist in a single magnetic conformation and a small part of the compound is paramagnetic. CASSCF calculated magnetic parameters based on PBEh‐3c/def2‐mSVP geometries support these results. The quantum chemical calculations strongly suggest this species to be the catalytically relevant one.

Concentration‐dependent UV–Vis studies of catalysts **C4** and **C7** show a linear absorption to concentration relationship thus pointing to monomeric catalyst species (see Supporting Information, Figures S32 and S34). This assumption is further supported by DFT computations, according to which the dimerization of the catalysts is either endergonic (**C7**) or not possible due to steric hindrance (**C4**). Moreover, the linear dependencies of catalyst *ee* and product *ee* values for both systems are consistent with monomeric catalyst species (see Supporting Information, Figures S29 and S30).

#### Computational and Kinetic Studies

**Scheme 2:**
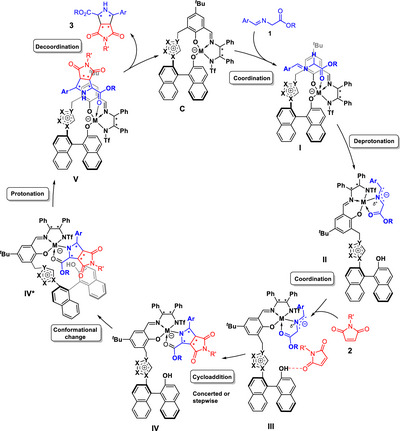
Proposed simplified catalytic cycle.

Based on the control experiments and the spectroscopic results in combination with previous experimental and computational studies on related Cu(II) catalysts reported by our groups,^[^ ^54^, ^65^, ^69^ ^]^ we used the simplified general catalytic cycle shown in Scheme 2 as working hypothesis for both the *endo*‐ and *exo*‐selective methods.

According to this mechanistic model, iminoester **1** initially coordinates to catalyst **C**, thus forming **I**. This is followed by a proton shift from the iminoester to the catalyst's naphthoxide moiety, giving azomethine ylide complex **II**. As a next step, maleimide **2** binds to **II** via a hydrogen bond to the generated naphthol‐OH, forming **III**. This allows for a quasi‐intramolecular (concerted or stepwise) cycloaddition. After the formation of the cycloadduct complex **IV**, a conformational change of the catalyst to IV* is necessary to enable protonation of the cycloadduct‐metal bond by the naphthol moiety to form the catalyst/product adduct **V**. Subsequent product dissociation closes the catalytic cycle.

Following that mechanistic model, comprehensive computational studies were conducted on the (B3LYP‐D3(BJ)/def2‐TZVP/ COSMO(THF)) level of theory on PBEh‐3c/def2‐mSVP geometries (for details see Supporting Information)^[^ ^79^, ^80^, ^81^, ^82^, ^83^, ^84^, ^85^, ^86^, ^87^, ^88^, ^89^, ^90^, ^91^, ^92^ ^]^ to give—in combination with kinetic and spectroscopic investigations—detailed mechanistic pictures for both the *endo*‐ and *exo*‐selective methods. In fact, according to our calculations, both reactions follow the general mechanism outlined above, but differ in various aspects that are important for the catalytic outcome.

#### Endo‐selective Approach With Catalyst **C4**

**Figure 1:**
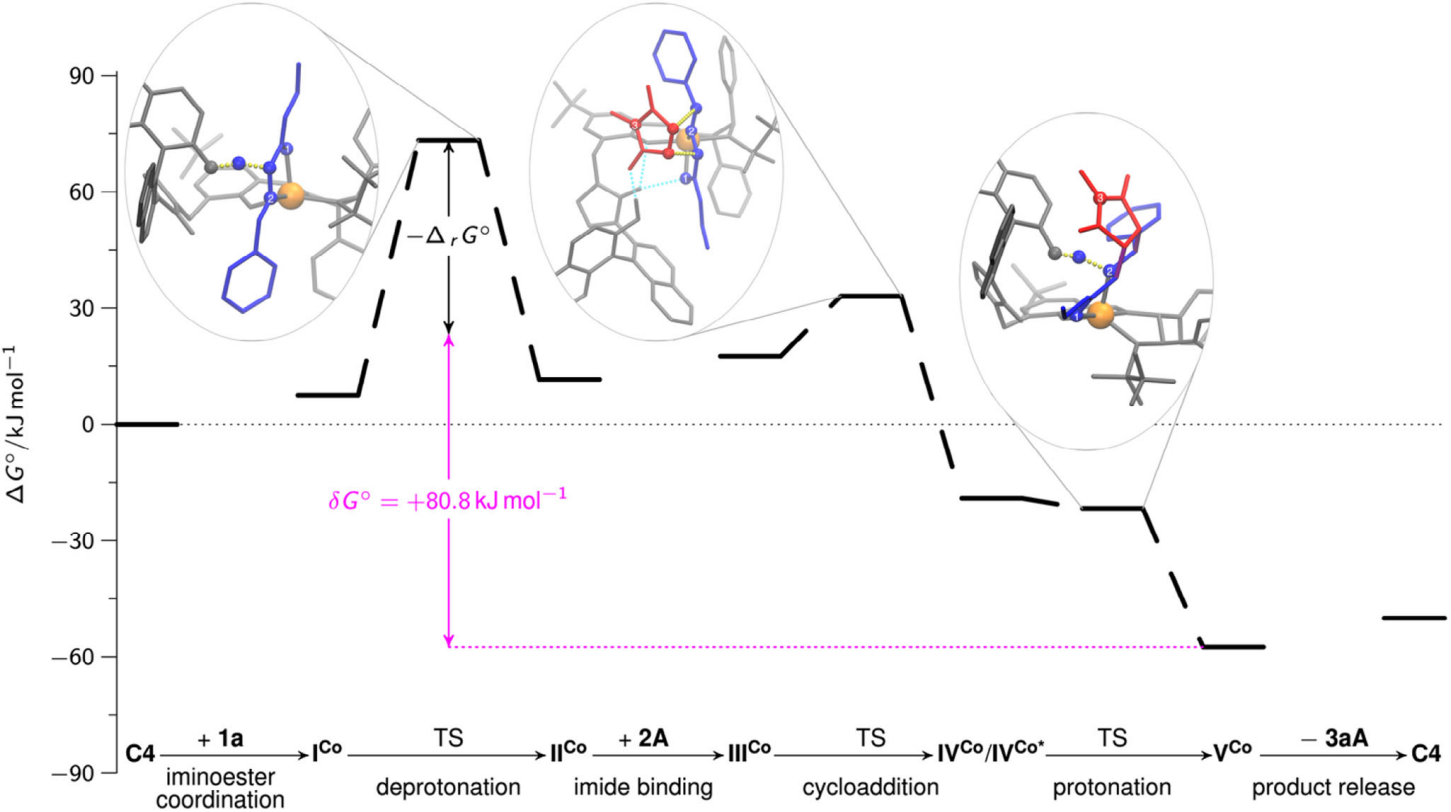
Free energy profile for the *endo*‐selective method with **C4** evaluating the proposed catalytic cycle (see Scheme 2) by DFT. **C4** and the isolated substrates (**1a**, **2A**) were chosen as the reference state (Δ*G°* = 0 kJ mol^−1^). In addition, the transition state structures for deprotonation, cycloaddition, and cycloadduct protonation are shown. The following color code was used for the structural representations: The catalyst is shown in gray, and the Co metal center is highlighted in orange. The 1,3‐dipole is shown in blue; the dipolarophile in red, and the novel C,C bonds in purple. Selected atoms are shown as balls, and the bonds to be formed or broken are indicated by yellow dots. To facilitate orientation, the carbonyl O‐atom of the iminoester is labeled “1” and the N‐atom “2”. The N‐atom of the maleimide is denoted by “3”. Δ*G°* denotes the Gibbs free energy, while δ*G°* refers to the energetic span.

As the magnetic measurements in solid state and solution (*vide supra*) show **C4** to be a high‐spin Co(II) complex, we evaluated the reaction pathway by DFT calculations on the respective quartet potential energy surface (PES). The predicted Gibbs free energy profile for the *endo*‐configured main stereoisomer with a thermodynamic driving force of Δ*_r_G*° = −50.0 kJ mol^−1^ is shown in Figure 1. Our DFT calculations suggest that the initial iminoester coordination to **C4** proceeds in a monodentate way via the ester moiety and is slightly endergonic. This step is followed by a slow and endergonic intramolecular deprotonation of the coordinated iminoester by the naphthoxide moiety to form the ylide‐like **II^Co^**, in which the Co(II) center adopts a trigonal‐bipyramidal geometry with elongated axial bonds. The formally neutral donor atoms coordinate axially, while the negatively charged ones form the equatorial plane. The subsequent imide association via H‐bond to the naphthol unit in **III^Co^** is again endergonic.

**Figure 2:**
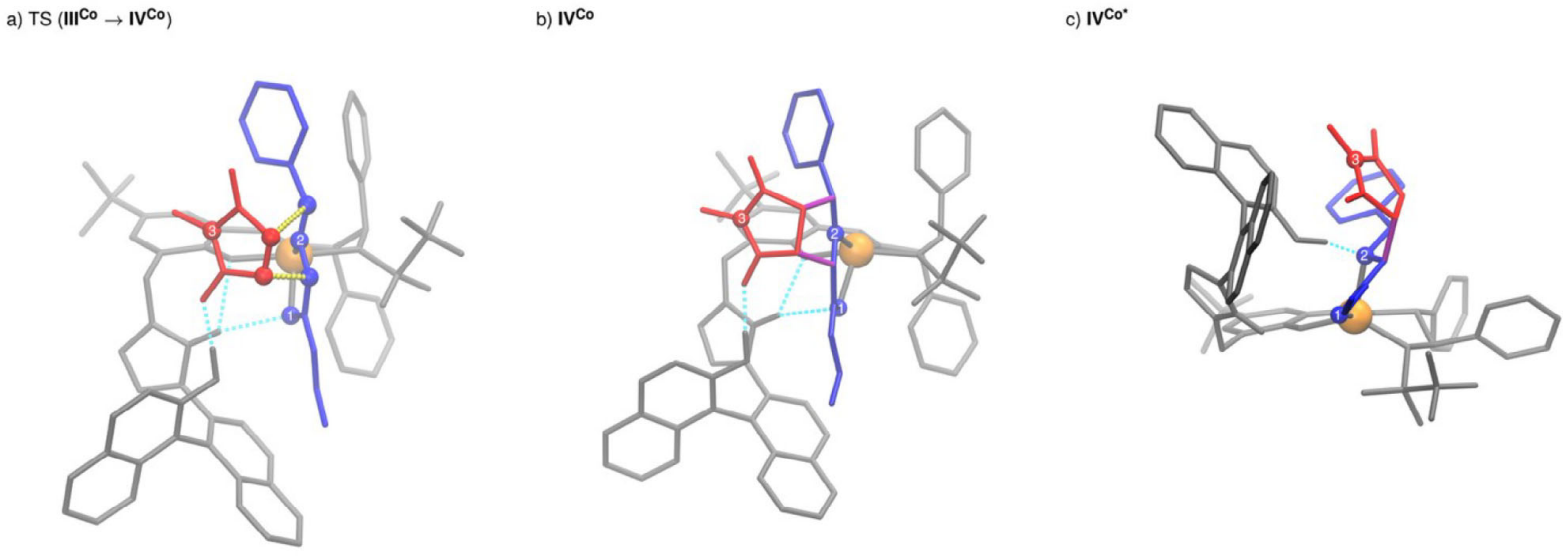
Molecular structures of TS(**III^Co^** →**IV^Co^**) (a; left), **IV^Co^** (b; middle) and IV^Co*^ (c; right). The following color code was used for this: The catalyst is shown in gray, the Co metal center is highlighted in orange, the 1,3‐dipole is shown in blue, the dipolarophile in red, and the novel C,C bonds in purple. Hydrogen bonds are indicated by light blue lines, and the bonds to be formed by yellow dotted lines. To facilitate orientation, the carbonyl O‐atom of the iminoester is labeled “1” and the N‐atom “2.” The N‐atom of the maleimide is denoted by “3.”

In contrast, the actual cycloaddition step forming two new C,C‐bonds is calculated to be strongly exergonic. Interestingly, it proceeds in a concerted manner for the *endo* pathway, while typically stepwise mechanisms have been suggested and calculated for other catalysts.^[^ ^93^, ^94^, ^95^ ^]^ In transition state TS(**III^Co^** →**IV^Co^**) (Figure 2, left), in addition to the hydrogen bond activation of the maleimide, the C–H acidic imidazolium forms a double hydrogen bond with the 1,3‐dipole and the phenoxy moiety of the ligand. The latter can serve as a structural anchor, rigidifying and stabilizing the assembly. As a result of the simultaneous activation of both substrates adopting a suitable spatial orientation for a quasi‐intramolecular step, a remarkably low barrier of 15.5 kJ mol^−1^ (enthalpic part: 9.8 kJ mol^−1^, entropic part: 5.7 kJ mol^−1^) is found for this event thus demonstrating the power of the polyfunctional catalysis concept in this key step. The chiral metal sphere and the axially‐chiral binaphthyl moiety form a well‐defined chiral binding pocket that stabilizes the spatial orientation required for the concerted mechanisms and effectively reduces the activation entropy. Moreover, by the precisely defined alignment of both substrates within the catalyst's chiral environment, high enantioselectivity is achieved.

For the competing *exo*‐pathway with **C4**, a stepwise cycloaddition mechanism was found requiring to surpass a significantly higher barrier (see Supporting Information, Figure S37).

The reaction steps following the C,C‐bond formations are particularly characteristic for the catalytic mechanism with **C4**. As shown in Figure 2, the naphthol unit in **IV^Co^** forms a hydrogen bond to the phenoxy unit of the ligand sphere. To enable the highly exergonic protonation of the cycloadduct, this bond must be broken, allowing for an isoenergetic conformational change of the imidazolium/naphthol unit. This change of **IV^Co^** to its conformer IV^Co*^ restores the trigonal‐bipyramidal coordination of the Co(II) center and permits the formation of a hydrogen bond between the naphthol unit and the pyrrolidinate nitrogen of the initial cycloadduct, thus facilitating a quasi‐barrierless proton transfer to form **V^Co^**. This Brønsted acid/base reaction step closely resembles enzymatically catalyzed proton transfer reactions.^[^ ^96^, ^97^ ^]^

The product release from **V^Co^** closes the catalytic cycle by regenerating **C4**. **V^Co^** is energetically favored compared to the iminoester/ catalyst complex **I^Co^** by −7.5 kJ mol^−1^.

According to the energetic span model,^[^ ^98^ ^]^ the catalytic activity of a system, measured as TOF, is usually determined by only two states: the turnover‐determining intermediate (TDI) and the turnover‐determining transition state (TDTS). Whenever the TDI appears before the TDTS in the catalysis cycle, the energetic span is determined by the Gibbs free energy difference between those two states. Otherwise, the energetic span is the sum of that Gibbs free energy difference and the thermodynamic driving force. The energetic span model enables the direct comparison between Gibbs free energy profiles obtained by quantum chemistry and the experimental kinetic investigations. Following the DFT evaluation of our mechanistic model (Figure 1), the energetic span (+80.8 kJ mol^−1^) of the investigated, *endo*‐selective catalytic reaction is given by **V^Co^** as TDI and the TS of the deprotonation step (**I^Co^** → **II^Co,a^**) as TDTS. Interestingly, the calculated entropic part of the activation barrier is almost zero (0.04 kJ mol^−1^), which is another similarity to enzymatic catalysis and might be explained by the rigidified catalyst structure, resulting from structural anchoring by H‐bond interactions.^[^ ^99^, ^100^ ^]^

The calculated span is in line with our experimental kinetic investigations, based on the determination of an empirical rate law by the variable time normalization analysis (VTNA) method by Burés.^[^ ^101^ ^]^ The best fit for the normalization of the time scale axis was achieved by the following equation:

$$r=k{\left[C4\right]}^{1.10}{\left[2A\right]}^{0.85}{\left[1a\right]}^{0.15}{\left[3aA\right]}^{-0.15}$$

with an experimental rate constant of *k*_obs_ = 10^3^ (L mol^−1^)^0.9 ^h^−1^. The resulting apparent barrier for the catalytic reaction **1a** + **2A** → **3aA** is + 76.1 kJ mol^−1^ and thus close to the calculated barrier. The empirical rate law is in agreement with the iminoester deprotonation as the most critical step for turnover.

Using a microkinetic model, barriers can be assigned to individual chemical steps from experimentally measured concentrations. For this purpose, the ordinary differential equation system resulting from the mechanistic hypothesis is solved, and the barriers of the individual reaction steps are optimized in order to best replicate the experimentally measured concentration profiles. In order to better understand the *endo*‐selective DCA, we decided to fit such a microkinetic model using methods (see Supporting Information) similar to those previously reported for an asymmetric hydroboration by our groups.^[^ ^102^ ^]^ According to our microkinetic model, the barrier of the iminoester deprotonation has the major influence on the kinetics, which is consistent with the identification of the corresponding TS as TDTS. Furthermore, the apparent barrier for the catalytic reaction **1a** + **2A** → **3aA** from our microkinetic model is found to be +75.6 kJ mol^−1^, which again agrees well with the calculated energetic span of + 80.8 kJ mol^−1^ that can be deducted from the Gibbs free energy profile (Figure 1) and the apparent barrier from VTNA analysis (*vide supra*).

#### Exo‐selective Approach With Catalyst **C7**

Since the magnetic measurements of the activated catalyst **C7** in solid state and solution (vide supra) combined with ^1^H‐NMR spectra suggest the presence of a low‐spin Ni(II) complex as largely dominating species which, however, also contains a paramagnetic high‐spin Ni(II) fraction, it was unclear which species is catalytically active. Hence, we studied central reaction intermediates on both the singlet and triplet PES (see Supporting Information). Our calculations indicate that the azomethine ylide coordination as a bidentate ligand to the catalyst is strongly endergonic on the singlet PES, while that step is only slightly uphill on the triplet PES. Moreover, the calculated iminoester deprotonation barrier in the singlet case is +111.8 kJ mol^−1^, which contrasts to the found triplet barrier of + 58.9 kJ mol^−1^ and is too high for the experimental reaction conditions and outcome. Thus, it is very likely that the minor high‐spin fraction of the Ni(II) complex is the catalytically active species.

**Figure 3:**
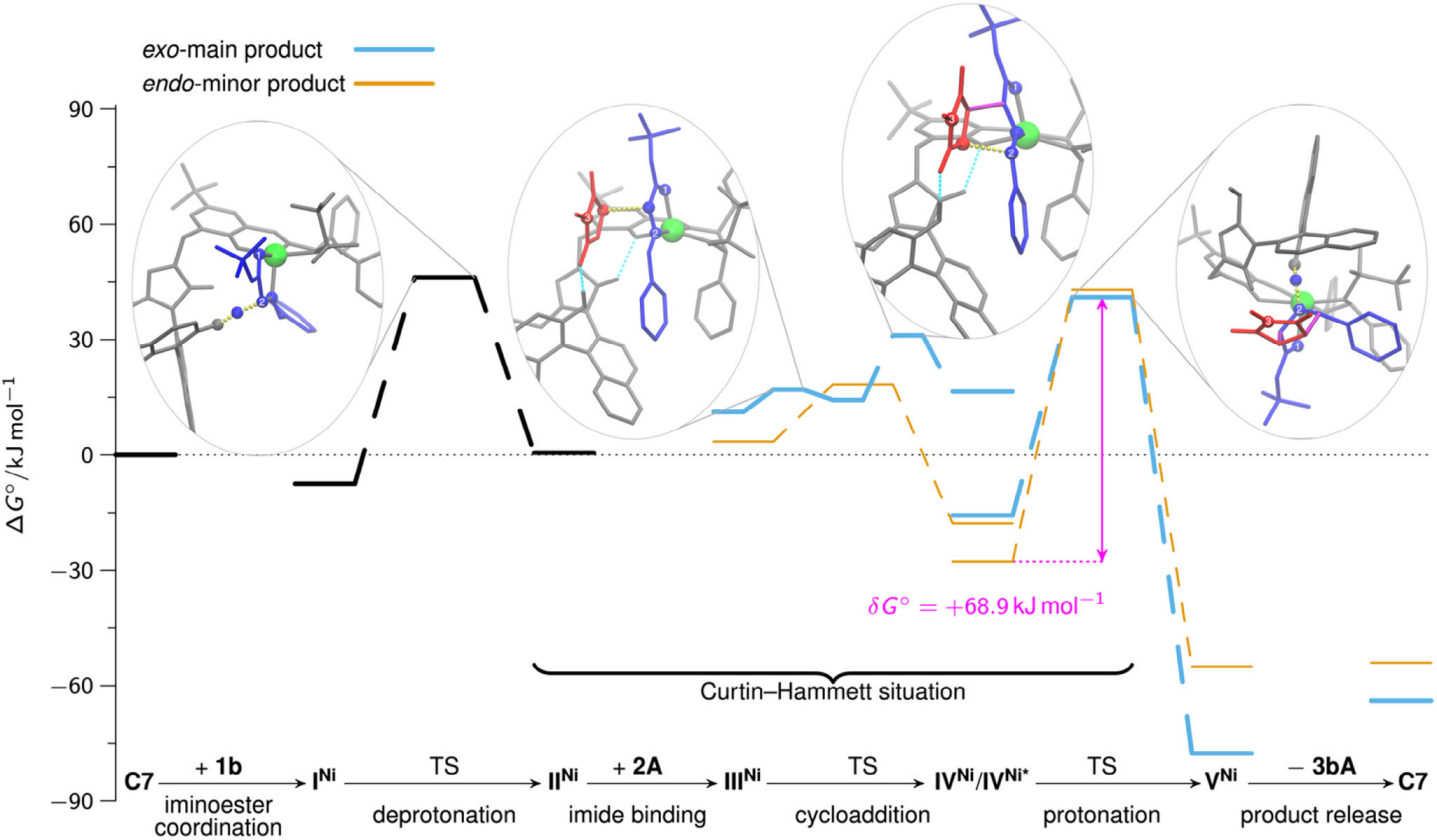
Free energy profiles for the *exo*‐selective method with **C7** evaluating the proposed catalytic cycle (see Scheme 2) by DFT. **C7** and the isolated substrates (**1b**, **2A**) were chosen as the reference state (Δ*G°* = 0 kJ mol^−1^). In addition, the transition state structures for deprotonation, C,C‐bond formations, and cycloadduct protonation are shown in the case of the *exo*‐main product (light blue profile; the competing *endo* profile is shown in orange). The following color code was used for the structural representations: The catalyst is shown in gray, and the Ni metal center is highlighted in green. The 1,3‐dipole is shown in blue; the dipolarophile in red, and the novel C,C bonds in purple. Selected atoms are shown as balls, and the bonds to be formed or broken are indicated by yellow dots. Selected atoms are shown as balls, and the bonds to be formed or broken are indicated by yellow dots. To facilitate orientation, the carbonyl O‐atom of the iminoester is labeled “1” and the N‐atom “2.” The N‐atom of the maleimide is denoted by “3.” Δ*G°* denotes the Gibbs free energy, while δ*G°* refers to the energetic span.

The predicted Gibbs free energy reaction profiles for the main *exo*‐ and minor *endo*‐product **3bA** with thermodynamic driving forces of Δ*_r_G*° = −62.1 and −54.0 kJ mol^−1^, respectively, are shown in Figure 3. In case of **C7**, the iminoester coordination to the catalyst is exergonic. The subsequent proton shift to the aryloxide moiety of the catalyst is endergonic, but has a comparatively low barrier. In contrast to the analogous Co(II)‐species derived from **C4**, the Ni(II) center in **II^Ni^** (and **III^Ni^** as well as **IV^Ni^**) adopts a square pyramidal geometry by coordination of both the ligand and the ylide.

**Figure 4:**
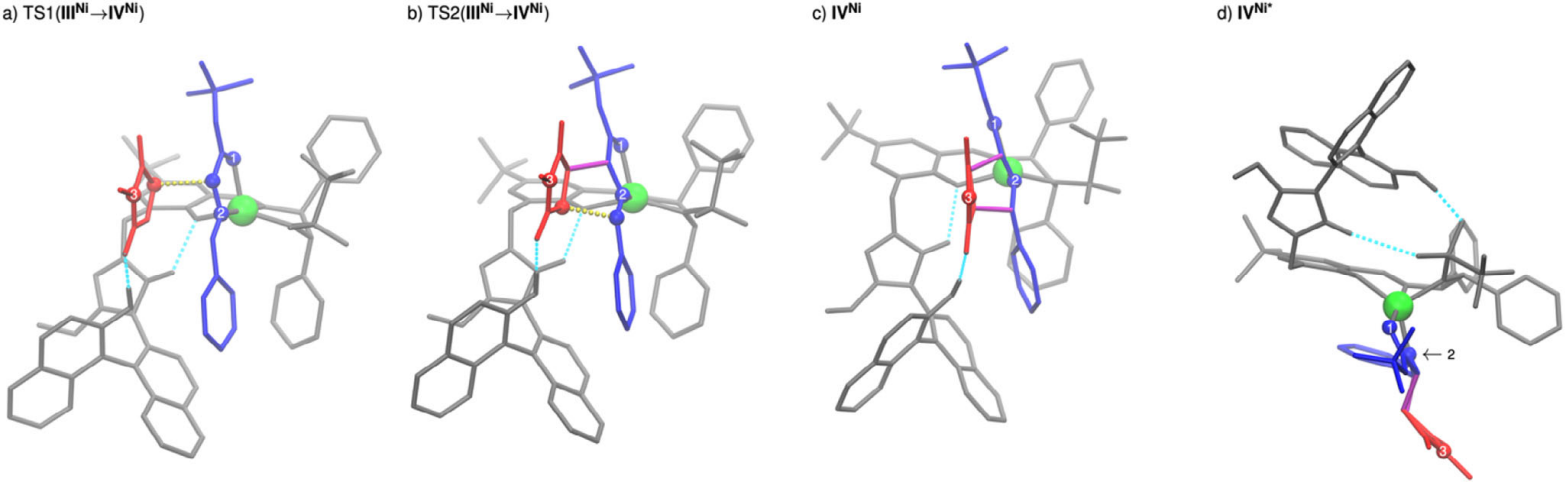
Molecular structures of the TSs of both consecutive steps of the *exo*‐cycloaddition: a) first C,C‐bond formation (**III^Ni^** → **IV^Ni^**), and b) second C,C‐bond formation (**III^Ni^** → **IV^Ni^**); in addition, intermediate **IV^Ni^** c) and its conformer IV^Ni*^ d) are shown for the *exo*‐pathway. The catalyst is shown in gray, the Ni metal center is highlighted in green, the 1,3‐dipole is shown in blue, the dipolarophile in red, and the novel C,C‐bonds in purple. Hydrogen bonds are indicated by light blue dotted lines, and the bonds to be formed by yellow dotted lines. To make orientation easier, the carbonyl O‐atom of the iminoester is labeled “1” and the N‐atom “2.” The N‐atom of the maleimide is denoted by “3.”

Again, imide binding to **II^Ni^** is endergonic. From imide adduct **III^Ni^** C,C‐bond formations occur in either concerted or stepwise manner, depending on the stereoisomer formed. To further investigate this result, 2D PES scans were performed for both possible *exo* enantiomers, varying both C─C bond lengths systematically (Supporting Information). It was found that only in the case where stabilization of a potential Michael intermediate by hydrogen bonding between it and the naphthol‐OH group is possible, a stepwise C,C‐bond formation can be achieved. In all other cases, an asynchronous concerted mechanism is observed. The described stabilization is only possible for one of the two *exo*‐product enantiomers, which is the main product using **C7**. In that case, in the TSs of both C,C‐bond formation steps (Figure 4a,b), the C–H acidic triazolium moiety forms a hydrogen bond with the phenoxy moiety of the ligand in addition to the hydrogen bond activation of the maleimide. Similarly to the concerted C,C‐bond formation with **C4** (Figure 2a), this serves as a structural anchor to stabilize the spatial position of the azolium / naphthol moiety.

After C,C‐bond formation, also **IV^Ni^** (Figure 4c) needs to undergo a conformational change to form IV^Ni*^, which is stabilized by a hydrogen bond between the naphthol OH group and the sulfonyl motif before protonation of the cycloadduct can take place. This change also results in a strong twisting of the azolium/naphthol moiety. In IV^Ni*^, it is possible to bind the naphthol OH to the Ni(II) center in an octahedral coordination geometry, allowing protonation of the *exo*‐configured cycloadduct (Figures 3 and 4d). Protonation of the minor *endo*‐isomer follows the same mechanism. Regeneration **C7** by product release closes the catalytic cycle. That dissociation is calculated to be endergonic for both *exo* and *endo* products.

Due to the low barrier of the C,C‐bond formations, they are reversible for both the *exo* and the *endo* reaction paths. In addition, the barrier of the cycloadduct protonation from IV^Ni*^ is higher than the barrier of the back‐reaction to **III^Ni^**. This results in a Curtin–Hammett situation, which means that IV^Ni*^ (*exo*) and IV^Ni*^ (*endo*) are in equilibrium with each other. As the barrier to cycloadduct protonation is lower for the *exo*‐product than for the *endo*‐product, the *exo*‐product is preferentially formed.

Based on our computational model, the *exo*:*endo*‐ratio is determined by the Gibbs free energy difference between the protonation TSs, i.e., TS IV^Ni*^ → **V^Ni^**. This value is found to be sensitive to the DFT functional used (see Supporting Information). B3LYP‐D3(BJ)/def2‐TZVP/COSMO(THF) yields a value of about 68:32. Using COSMO‐RS^[^ ^103^, ^104^, ^105^ ^]^ instead of COSMO^[^ ^106^, ^107^, ^108^ ^]^ as an implicit solvent mode predicts an *exo*:*endo*‐ratio of 88:12 (experimental: 92:8).

The empirical rate‐law determined by VTNA

$$r=k{\left[C7\right]}^{0.9}{\left[1b\right]}^{0.9}{\left[2A\right]}^{0.7}{\left[3bA\right]}^{-0.1}$$

is in agreement with the calculated mechanism. According to the energetic span model, the cycloadduct state IV^Ni*^ and the subsequent protonation TS determine the TOF of the *exo*‐selective catalysis with **C7**. An energetic span of +68.9 kJ mol^−1^ is calculated in that case.

## Conclusions

In summary, we have developed a new catalyst concept for 1,3‐DCA, which allows selective access to both the *endo* and *exo* diastereomers on demand from azomethine ylides and maleimides. Two highly productive polyfunctional catalysts were found, each offering a mode of action that is reminiscent of enzymatic catalysis in various aspects.

For the *endo*‐selective 1,3‐DCA with **C4**, the combination of experimental and computational mechanistic studies shows that the high diastereo‐ and enantioselectivity, as well as the excellent productivity, can be attributed to a nearly perfect spatial fit between the functional groups of the catalyst and the functional units of both reaction partners throughout the whole catalytic cycle. Once the limiting activation barrier for deprotonation is overcome, a simple rotation around the CH₂ unit, bridging the metal‐sphere and the azolium/naphthol motif, enables the functional groups of the catalyst to be arranged in a nearly optimal position for all individual steps of the catalytic cycle, in particular for the low‐barrier concerted cycloaddition event. The resulting structures of the catalytic cycle are stabilized by the interplay of hydrogen bonding, allowing for a cooperative mode of substrate activation. This shows a strong analogy to the working principles of enzymes.

To enable the desired switch in diastereoselectivity, it is necessary to hamper this main, inherently preferred pathway. This can be achieved by changing the metal center from Co(II) to Ni(II), thus changing the preferred metal coordination sphere, while also varying the azolium moiety (to facilitate twisting of the azolium / naphthol moiety) and increasing the steric demand of the ester residue of the iminoester (Et vs. *^t^* Bu).

In case of the *exo*‐selective catalysis with **C7**, the modified coordination polyhedron of the Ni(II) center enables the formation of a structurally well‐defined binding pocket after deprotonation of the iminoester by forming a hydrogen bond between the naphthol group and the sulfonyl unit. As a result, the acidic proton of the naphthol group is in close proximity to the pyrrolidinate nitrogen atom, which enables a facilitated proton transfer. The selectivity is a result of a Curtin–Hammett situation and is strongly dependent on steric interactions.

This interdisciplinary study demonstrates the large potential of the modular polyfunctional Lewis acid/azolium aryloxide catalysts in terms of productivity and well‐defined stereocontrol. The mode of action is comparable to an enzymatic machinery, which is capable of performing and controlling a number of sequential events in a very precise manner. The efficiency of these systems culminates in a facilitation of the actual key step in such a way that their barrier is lower than that of the seemingly more trivial steps. However, as the reported catalyst systems are also capable of controlling and accelerating these steps, their efficiency is without precedent.

## Conflict of Interests

The authors declare no conflict of interest.

## Supporting information



Supporting Information

Supporting Information

## Data Availability

The data that support the findings of this study are available in the Supporting Information of this article.
