# Supplementary material for: Tunable Endo/Exo Selectivity in Direct Catalytic Asymmetric 1,3‐Dipolar Cycloadditions with Polyfunctional Lewis Acid / Azolium–Aryloxide Catalysts
Source: Angew Chem Int Ed Engl. 2025 Jul 7;64(34):e202508024. doi: 10.1002/anie.202508024 (PMC12363618; doi:10.1002/anie.202508024)
Supplement: Supplementary file 1 — Supporting Information [file ANIE-64-e202508024-s002.pdf]

Supporting Information

Tunable *Endo*/*Exo* Selectivity in Direct Catalytic Asymmetric 1,3-Dipolar Cycloadditions with Polyfunctional Lewis Acid / Azolium-Aryloxide Catalysts

Adrian Bürstner,[a]§ Patrick M. Becker,[b]§ Alexander Allgaier,[c] Lucca Pfitzer,[a] Daniel M. Wanner,[a] Johanna Dollinger,[a] Felix Willig,[a] Justin Herrmann,[a] Vukoslava Miskov-Pajic,[a] Andreas C. Hans,[a] Wolfgang Frey,[a] Joris van Slageren,[c] Johannes Kästner,[b] and René Peters*[a]

[a] Institut für Organische Chemie, Universität Stuttgart, Pfaffenwaldring 55, D-70569 Stuttgart, Germany

[b] Institut für Theoretische Chemie, Universität Stuttgart, Pfaffenwaldring 55, D-70569 Stuttgart, Germany

[c] Institut für Physikalische Chemie, Universität Stuttgart, Pfaffenwaldring 55, D-70569 Stuttgart, Germany

§ Both authors contributed equally

Email: rene.peters@oc.uni-stuttgart.de

Table of Contents

TABLE OF CONTENTS **2**

1 GENERAL REMARKS **7**

2 GENERAL PROCEDURES **9**

2.1 GP1: ALKYLATION WITH MEERWEIN SALT 9

2.2 GP2: IMINE CONDENSATION 9

2.3 GP3: COMPLEXATION 10

2.4 GP4: CATALYST ACTIVATION PROCEDURE 10

2.5 GP5: SYNTHESIS OF GLYCINE-BASED IMINOESTERS 11

2.6 GP6: CATALYTIC ASYMMETRIC 1,3-DIPOLAR CYCLOADDITION OF AZOMETHINE YLIDES TO MALEIMIDES USING *LEWIS*-ACID-PHENOXYIMINE-COMPLEXES 11

2.7 GP7: CATALYTIC ASYMMETRIC 1,3-DIPOLAR CYCLOADDITION OF AZOMETHINE YLIDES TO MALEIMIDES AT LOW CATALYST LOADINGS (<0.1 MOL%) 12

2.8 GP8: RECYCLING OF THE *LEWIS*-ACID-PHENOXYIMINE-COMPLEXES 13

3 PRECURSOR SYNTHESIS **14**

3.1 SYNTHESIS OF (*R*)-1-(2'-HYDROXY-[1,1'-BINAPHTHALEN]-2-YL)-3-METHYL-1*H*-IMIDAZOL-3-IUM IODIDE (CC6*HI) . 14

3.2 SYNTHESIS OF 5-TERT-BUTYL-3-((3-ETHYL-4-PHENYL-TRIAZOL-3-IUM-1-YL)-METHYL)-2-HYDROXY-BENZALDEHY HEXAFLUOROPHOSPHATE (LP1) 15

4 LIGAND SYNTHESIS **16**

4.1 SYNTHESIS OF 3-(5-(*TERT*-BUTYL)-3-((*E*)-(((1*R*,2*R*)-1,2-DIPHENYL-2-((TRIFLUOROMETHYL)SULFONAMIDO)ETHYL)IMINO)METHYL)-2-HYDROXYBENZYL)-1-PHENYL-1*H*-IMIDAZOL-3-IUM CHLORIDE (L1) 16

4.2 SYNTHESIS OF ((1*S*,2*S*)-2-((5-*TERT*-BUTYL-3-((3-ETHYL-4-PHENYL-TRIAZOL-3-IUM-1-YL)-METHYL)-2-HYDROXY-PHENYL)-METHYLENAMINO)-1,2-DIPHENYL-ETHYL)-1,1,1-TRIFLUORO-METHANESULFONAMIDE HEXAFLUOROPHOSPHATE (L2) .. 17

5 SYNTHESES OF THE COMPLEXES **18**

5.1 SYNTHESIS OF THE ZN(II)-PRECATALYST 3-(5-(*TERT*-BUTYL)-3-((((1*R*,2*R*)-1,2-DIPHENYL-2-((TRIFLUOROMETHYL)SULFONAMIDO)ETHYL)IMINO)METHYL)-2-OXYBENZYL)-1-((*R*)-2'-HYDROXY-[1,1'-BINAPHTHALEN]-2-YL)-1*H*-IMIDAZOLE-3-IUM-ZN(II) CHLORIDE (C2*HCL) 18

5.2 SYNTHESIS OF THE CO(II)-PRECATALYST 3-(5-(*TERT*-BUTYL)-3-((((1*S*,2*S*)-1,2-DIPHENYL-2-((TRIFLUOROMETHYL)SULFONAMIDO)ETHYL)IMINO)METHYL)-2-OXYBENZYL)-1-((*R*)-2'-HYDROXY-[1,1'-BINAPHTHALEN]-2-YL)-1*H*-IMIDAZOLE-3-IUM-CO(II) CHLORIDE (C5*HCL) 19

5.3 SYNTHESIS OF THE CO(II)-PRECATALYST 3-(5-(*TERT*-BUTYL)-3-((((1*R*,2*R*)-1,2-DIPHENYL-2-((TRIFLUOROMETHYL)SULFONAMIDO)ETHYL)IMINO)METHYL)-2-OXYBENZYL)-1-((*R*)-2'-HYDROXY-[1,1'-BINAPHTHALEN]-2-YL)-1*H*-IMIDAZOLE-3-IUM-CO(II) CHLORIDE (C4*HCL) 20

5.4 SYNTHESIS OF THE CO(II)-PRECATALYST (*R*)-2'-(3-(5-(*TERT*-BUTYL)-3-((*E*)-(((1*R*,2*R*)-1,2-DIPHENYL-2-((TRIFLUOROMETHYL)SULFONAMIDO)ETHYL)IMINO)METHYL)-2-OXYBENZYL)-1*H*-IMIDAZOL-3-IUM-1-YL)-[1,1'-BINAPHTHALEN]-2-OLATE-COBALT(II) (C4) 21

5.5 SYNTHESIS OF THE CO(II)-PRECATALYST 3-(5-(*TERT*-BUTYL)-3-((((1*S*,2*S*)-1,2-DIPHENYL-2-((TRIFLUOROMETHYL)SULFONAMIDO)ETHYL)IMINO)METHYL)-2-OXYBENZYL)-1-((*S*)-2'-HYDROXY-[1,1'-BINAPHTHALEN]-2-YL)-1*H*-IMIDAZOLE-3-IUM-CO(II) CHLORIDE (*ENT*-C4*HCL) 22

5.6 SYNTHESIS OF THE CO(II)-PRECATALYST (1*R*,2*R*)-2-((5-(*TERT*-BUTYL)-2-OXY-3-((*R*)-((2'-OXY-[1,1'-BINAPHTHALEN]-2-YL)OXY)METHYL)BENZYLIDENE)AMINO)-1,2-DIPHENYLETHYL)-1,1,1-TRIFLUOROMETHANE-SULFONAMIDE-CO(II) (CC2*H) ... 23

5.7 SYNTHESIS OF THE CO(II)-PRECATALYST 3-(5-(*TERT*-BUTYL)-3-((((1*R*,2*R*)-1,2-DIPHENYL-2-((TRIFLUOROMETHYL)SULFONAMIDO)ETHYL)IMINO)METHYL)-2-OXYBENZYL)-1-(2-HYDROXYPHENYL)-1H-IMIDAZOL-3-IUM-CO(II) CHLORIDE (CC3*HCL) 24

5.8 SYNTHESIS OF THE CO(II)-PRECATALYST 3-(5-(*TERT*-BUTYL)-3-((((1*R*,2*R*)-1,2-DIPHENYL-2-((TRIFLUOROMETHYL)SULFONAMIDO)ETHYL)IMINO)METHYL)-2-OXYBENZYL)-1-PHENYL-1*H*-IMIDAZOL-3-IUM-CO(II) CHLORIDE (CC4) 25

5.9 SYNTHESIS OF THE CO(II)-PRECATALYST ((1*R*,2*R*)-2-((5-(*TERT*-BUTYL)-2-OXY-3-METHYLBENZYLIDENE)AMINO)-1,2-DIPHENYLETHYL)-1,1,1-TRIFLUOROMETHANE-SULFONAMIDE-CO(II) (CC5) 26

5.10 SYNTHESIS OF THE NI(II)-PRECATALYST 1-(5-(*TERT*-BUTYL)-3-((((1*S*,2*S*)-1,2-DIPHENYL-2-((TRIFLUOROMETHYL)SULFONAMIDO)ETHYL)IMINO)METHYL)-2-OXYBENZYL)-3-ETHYL-4-((*R*)-2'-HYDROXY-[1,1'-BINAPHTHALEN]-2-YL)-1*H*-1,2,3-TRIAZOL-3-IUM-NI(II) HEXAFLUOROPHOSPHATE(V) (C7*HPF_6) 27

5.11 SYNTHESIS OF THE NI(II)-PRECATALYST (*R*)-2'-(1-(5-(*TERT*-BUTYL)-3-((*E*)-(((1*S*,2*S*)-1,2-DIPHENYL-2-((TRIFLUOROMETHYL)SULFONAMIDO)ETHYL)IMINO)METHYL)-2-OXYBENZYL)-3-ETHYL-1*H*-1,2,3-TRIAZOL-3-IUM-4-YL)-[1,1'-BINAPHTHALEN]-2-OLATE-NICKEL(II) (C7) 29

5.12 SYNTHESIS OF THE NI(II)-PRECATALYST 1-(5-(*TERT*-BUTYL)-3-((((1*R*,2*R*)-1,2-DIPHENYL-2-((TRIFLUOROMETHYL)SULFONAMIDO)ETHYL)IMINO)METHYL)-2-OXYBENZYL)-3-ETHYL-4-((*R*)-2'-HYDROXY-[1,1'-BINAPHTHALEN]-2-YL)-1*H*-1,2,3-TRIAZOL-3-IUM-NI(II) HEXAFLUOROPHOSPHATE(V) (C6*HPF_6) 30

5.13 SYNTHESIS OF THE CO(II)-PRECATALYST 1-(5-(*TERT*-BUTYL)-3-((((1*S*,2*S*)-1,2-DIPHENYL-2-((TRIFLUOROMETHYL)SULFONAMIDO)ETHYL)IMINO)METHYL)-2-OXYBENZYL)-3-ETHYL-4-((*R*)-2'-HYDROXY-[1,1'-BINAPHTHALEN]-2-YL)-1*H*-1,2,3-TRIAZOL-3-IUM-CO(II) HEXAFLUOROPHOSPHATE(V) (CC1*HPF_6) 31

5.14 SYNTHESIS OF THE NI(II)-PRECATALYST 1-(5-(*TERT*-BUTYL)-3-((((1*R*,2*R*)-1,2-DIPHENYL-2-((TRIFLUOROMETHYL)SULFONAMIDO)ETHYL)IMINO)METHYL)-2-OXYBENZYL)-3-ETHYL-4-((*S*)-2'-HYDROXY-[1,1'-BINAPHTHALEN]-2-YL)-1*H*-1,2,3-TRIAZOL-3-IUM-NI(II) HEXAFLUOROPHOSPHATE(V) (*ENT*-C7*HPF_6) 32

5.15 SYNTHESIS OF THE NI(II)-PRECATALYST 1-(5-(*TERT*-BUTYL)-3-((((1*R*,2*R*)-1,2-DIPHENYL-2-(METHYLSULFONAMIDO)ETHYL)IMINO)METHYL)-2-OXYBENZYL)-3-ETHYL-4-((*R*)-2'-HYDROXY-[1,1'-BINAPHTHALEN]-2-YL)-1*H*-1,2,3-TRIAZOL-3-IUM-NI(II) HEXAFLUOROPHOSPHATE(V) (CC8*HPF_6) 33

5.16 SYNTHESIS OF THE NI(II)-PRECATALYST 1-(5-(*TERT*-BUTYL)-3-((((1*S*,2*S*)-1,2-DIPHENYL-2-((TRIFLUOROMETHYL)SULFONAMIDO)ETHYL)IMINO)METHYL)-2-OXYBENZYL)-3-ETHYL-4-(2'-HYDROXY-[1,1'-BIPHENYL]-2-YL)-1*H*-1,2,3-TRIAZOL-3-IUM-NI(II) HEXAFLUOROPHOSPHATE(V) (CC10*HPF_6) 34

5.17 Synthesis of the Ni(II)-Precatalyst 1-(5-(*tert*-Butyl)-3-((((1*S*,2*S*)-1,2-diphenyl-2-((trifluoromethyl)sulfonamido)ethyl)imino)methyl)-2-oxybenzyl)-3-ethyl-4-(2-hydroxyphenyl)-1*H*-1,2,3-triazol-3-ium-Ni(II) Hexafluorophosphate(V) (CC9*HPF_6) ... 35

5.18 Synthesis of the Ni(II)-Precatalyst 1-(5-(*tert*-Butyl)-3-((((1*S*,2*S*)-1,2-diphenyl-2-((trifluoromethyl)sulfonamido)ethyl)imino)methyl)-2-oxybenzyl)-3-ethyl-4-phenyl-1*H*-1,2,3-triazol-3-ium-Ni(II) Hexafluorophosphate(V) (CC11) ... 37

5.19 Synthesis of the Ni(II)-Precatalyst ((1*S*,2*S*)-2-((5-(*tert*-Butyl)-2-oxy-3-methylbenzylidene)amino)-1,2-diphenylethyl)-1,1,1-trifluoromethane-sulfonamide-Ni(II) (CC12) ... 38

6 Substrate Synthesis ... 40

6.1 Synthesis of Methyl-(*E*)-2-(benzylideneamino)acetate (1c)[7,8] ... 40

6.2 Synthesis of Ethyl-(*E*)-2-(benzylideneamino)acetate (1a)[7] ... 40

6.3 Synthesis of Benzyl-(*E*)-2-(benzylideneamino)acetate (1d) ... 40

6.4 Synthesis of Ethyl-(*E*)-2-((4-methoxybenzylidene)amino)acetate (1e)[7] ... 41

6.5 Synthesis of Ethyl-(*E*)-2-((4-methylbenzylidene)amino)acetate (1f)[7,9] ... 41

6.6 Synthesis of Ethyl-(*E*)-2-((4-chlorobenzylidene)amino)acetate (1g)[7,10] ... 41

6.7 Synthesis of Ethyl-(*E*)-2-((3-chlorobenzylidene)amino)acetate (1h)[7,11] ... 42

6.8 Synthesis of Ethyl-(*E*)-2-((2-chlorobenzylidene)amino)acetate (1i)[7,12] ... 42

6.9 Synthesis of Ethyl-(*E*)-2-((4-nitrobenzylidene)amino)acetate (1j)[7,10] ... 43

6.10 Synthesis of *tert*-Butyl-(*E*)-2-(benzylideneamino)acetate (1b)[7,13] ... 43

6.11 Synthesis of *tert*-Butyl-(*E*)-2-((4-methoxybenzylidene)amino)acetate (1k)[7,14] ... 43

6.12 Synthesis of *tert*-Butyl-(*E*)-2-((4-chlorobenzylidene)amino)acetate (1l)[7,15] ... 44

6.13 Synthesis of *tert*-Butyl-(*E*)-2-((4-methylbenzyliden)amino)acetat (1m)[7,16] ... 44

6.14 Synthesis of *tert*-Butyl-(*E*)-2-((4-nitrobenzylidene)amino)acetate (1n)[7,17] ... 44

7 Synthesis of Asymmetric Cycloaddition Products ... 46

7.1 Synthesis of Methyl-(1*S*,3*R*,3a*S*,6a*R*)-5-methyl-4,6-dioxo-3-phenyloctahydropyrrolo[3,4-*c*]pyrrole-1-carboxylate (3cA)[2,18–20] ... 46

7.2 Synthesis of ethyl-(1*S*,3*R*,3a*S*,6a*R*)-5-methyl-4,6-dioxo-3-phenyloctahydropyrrolo[3,4-*c*]pyrrol-1-carboxylate (3aA)[2,5,21] ... 46

7.3 Synthesis of benzyl-(1*S*,3*R*,3a*S*,6a*R*)-5-methyl-4,6-dioxo-3-phenyloctahydropyrrolo[3,4-c]pyrrole-1-carboxylate (3dA) ... 47

7.4 Synthesis of ethyl-(1*S*,3*R*,3a*S*,6a*R*)-3-(4-methoxyphenyl)-5-methyl-4,6-dioxooctahydropyrrolo[3,4-c]pyrrole-1-carboxylate (3eA)[2,22] ... 48

7.5 Synthesis of ethyl-(1*S*,3*R*,3a*S*,6a*R*)-5-methyl-4,6-dioxo-3-(*p*-tolyl)octahydropyrrolo[3,4-c]pyrrole-1-carboxylate (3fA) ... 49

7.6 Synthesis of ethyl-(1*S*,3*R*,3a*S*,6a*R*)-3-(4-chlorophenyl)-5-methyl-4,6-dioxooctahydropyrrolo[3,4-*c*]pyrrole-1-carboxylate (3gA)[2,5,23] ... 50

7.7 Synthesis of ethyl-(1*S*,3*R*,3a*S*,6a*R*)-3-(3-chlorophenyl)-5-methyl-4,6-dioxooctahydropyrrolo[3,4-c]pyrrole-1-carboxylate (3hA) 50
7.8 Synthesis of Ethyl-3-(2-chlorophenyl)-5-methyl-4,6-dioxooctahydropyrrolo[3,4-*c*]pyrrole-1-carboxylate (3iA) 51
7.9 Synthesis of ethyl-(1*S*,3*R*,3a*S*,6a*R*)-5-methyl-3-(4-nitrophenyl)-4,6-dioxooctahydropyrrolo[3,4-c]pyrrole-1-carboxylate (3jA) 52
7.10 Synthesis of ethyl-(1*S*,3*R*,3a*S*,6a*R*)-4,6-dioxo-3-phenyloctahydropyrrolo[3,4-c]pyrrole-1-carboxylate (3aB) 53
7.11 Synthesis of ethyl-(1*S*,3*R*,3a*S*,6a*R*)-4,6-dioxo-3,5-diphenyloctahydropyrrolo[3,4-*c*]pyrrole-1-carboxylate (3aC)[2,5,24] 54
7.12 Synthesis of ethyl-(1*S*,3*R*,3a*S*,6a*R*)-5-benzyl-4,6-dioxo-3-phenyloctahydropyrrolo[3,4-*c*]pyrrole-1-carboxylate (3aD) 55
7.13 Synthesis of *tert*-butyl-(1*R*,3*S*,3a*S*,6a*R*)-5-methyl-4,6-dioxo-3-phenyloctahydropyrrolo[3,4-*c*]pyrrole-1-carboxylate (3bA) 56
7.14 Synthesis of *tert*-butyl-(1*R*,3*S*,3a*S*,6a*R*)-3-(4-methoxyphenyl)-5-methyl-4,6-dioxooctahydropyrrolo[3,4-*c*]pyrrole-1-carboxylate (3kA) 57
7.15 Synthesis of *tert*-butyl-(1*R*,3*S*,3a*S*,6a*R*)-3-(4-chlorophenyl)-5-methyl-4,6-dioxooctahydropyrrolo[3,4-*c*]pyrrole-1-carboxylate (3lA) 57
7.16 Synthesis of *tert*-butyl-(1*R*,3*S*,3a*S*,6a*R*)-5-methyl-4,6-dioxo-3-(*p*-tolyl)octahydropyrrolo[3,4-*c*]pyrrole-1-carboxylate (3mA) 58
7.17 Synthesis of *tert*-butyl-(1*R*,3*S*,3a*S*,6a*R*)-5-methyl-3-(4-nitrophenyl)-4,6-dioxooctahydropyrrolo[3,4-*c*]pyrrole-1-carboxylate (3nA) 59
7.18 Synthesis of *tert*-butyl-(1*R*,3*S*,3a*S*,6a*R*)-4,6-dioxo-3,5-diphenyloctahydropyrrolo[3,4-*c*]pyrrole-1-carboxylate (3bC) 60
7.19 Synthesis of *tert*-butyl-(1*R*,3*S*,3a*S*,6a*R*)-5-benzyl-4,6-dioxo-3-phenyloctahydropyrrolo[3,4-*c*]pyrrole-1-carboxylate (3bD) 61

8 DETERMINATION OF THE RELATIVE AND ABSOLUTE CONFIGURATION OF PYRROLIDINES 63

9 KINETIC STUDIES 69

9.1 VTNA 69
9.2 NMR Data for VTNA 79

10 SPECTROSCOPY 89

10.1 Co(II)-imidazolium-phenoxyimine complex C4 89
10.2 Ni(II)-triazolium-phenoxyimine complex C7 90

11 MAGNETOMETRY 92

12 EPR-EXPERIMENTS 96

13 INVESTIGATION OF A POSSIBLE NON-LINEAR EFFECT 98

14 UV-VIS EXPERIMENTS 102

14.1 *BEER*'S PLOT 102

15 COMPUTATIONAL STUDIES 106

15.1 GENERAL 106
15.2 GENERATING STRUCTURE FOR C4 AND C7 107
15.3 THE *EXO*-SELECTIVE APPROACH WITH C7 108
15.4 THE *ENDO*-SELECTIVE APPROACH WITH C4 113
15.5 COMPARISON OF THE *ENDO*- AND THE *EXO*-SELECTIVE APPROACH 120
15.6 DATA BEHIND THE FIGURES 125

16 NMR DATA 126

17 CHROMATOGRAMS 144

18 *DR*-VALUES OF CATALYTIC REACTIONS 163

19 REFERENCES 174

1 General Remarks

Unless otherwise stated (dry solvent), the syntheses were carried out under atmospheric conditions. For work under inert gas conditions, the glass equipment was heated for 5 minutes under high vacuum at 600 °C or heated for 18 h in a drying oven at 150 °C and cooled under high vacuum. The solvents for column chromatography and further workup steps, such as petroleum ether, ethyl acetate, dichloromethane, diethyl ether, and *n*-pentane, were purchased commercially and distilled before further use. The solvents for work under inert gas conditions, such as toluene, acetonitrile, dichloromethane, tetrahydrofuran, n-pentane, and diethyl ether, were dried in a solvent purification system (*MBRAUN MB SPS-800*). Other dry solvents were either dried according to literature procedures[1] or purchased commercially from *Sigma-Aldrich* and used without further purification. The molecular sieves (3 and 4Å) and the silica gel used for the purification of the imino esters were dried for at least 5 days at 150 °C and cooled under high vacuum before use. Unless otherwise stated, all chemicals used in this work were purchased from the commercial suppliers *Sigma-Aldrich*, *Fluorochem, Fluka*, *ABCR*, *TCI, Acros Organics* or *Alfa Aesar* and used without further purification. The nickel(II)-acetylacetonate used for the complexation was recrystallized from methanol following a literature procedure[1] and then dried in a Kugelrohr distillation apparatus at 100 °C under high vacuum for 18 h. The maleimides (*N*-methyl, *N*-H, *N*-phenyl) were purchased from *Sigma-Aldrich* and used without further purification. The benzaldehyde derivatives for the synthesis of the imino esters were purified according to a known literature procedure.[1] The ligands and complexes in this work were prepared according to literature procedures.[2–5] The hydrochlorides for the synthesis of the imino esters were purchased from *Sigma-Aldrich* or *Fluorochem* and used without further purification.

The ^{1}H-NMR measurements were performed on a *Bruker Avance* (300, 400, 500, and 700 MHz). All NMR measurements in this work were performed at a temperature of 298 K. The spectrometer frequency and the deuterated solvent used can be found in the individual synthesis instructions. The ^{13}C-NMR measurements were performed on a *Bruker Avance* (176 MHz). The ^{19}F-NMR measurements were performed on a *Bruker Avance* (376 MHz). The IR-measurements were performed by the Analytical Service Department of the Institute of Organic Chemistry at the University of Stuttgart using a *Bruker Alpha* FT-IR spectrometer. The UV-Vis measurements were performed on a *Perkin Elmer Lambda* 365 spectrometer. The mass spectrometry measurements were performed by the Analytical Service Department of the Institute of Organic Chemistry at the University of Stuttgart. An Exactive GC Orbitrap MS System was used for the EI and CI measurements. An Exactive Plus Orbitrap Orbitrap MS System was used for the ESI measurements. The ee-values were determined by HPLC measurements on a chiral stationary phase. A *VWR Elite LaChrom* system with *Hitachi* modules was used for this purpose. Chiral HPLC columns of the types *Chiralpak IH* (25 cm)

and *Chiralpak IA* (25 cm) from *Daicel* were used as the stationary phase in this study. The solvent mixture used, the flow rate, the detector wavelength, and the corresponding retention times can be found in the individual synthesis instructions. The determination of the specific rotation of chiral compounds was carried out using a *Perkin Elmer* 241 polarimeter, using the Na^{D} line (λ = 589 nm), the Hg double line (λ = 578 nm), and the Hg line (λ = 546 nm) in a 10 cm quartz glass cuvette. The concentration and solvent used for the measurements are listed in the corresponding synthesis instructions. The uncorrected melting ranges of the corresponding compounds were determined using a *Büchi Melting Point* B-535. For the measurements, the samples were prepared in an open glass capillary. The crystal structures were determined by Dr. Wolfgang Frey of the Institute of Organic Chemistry at the University of Stuttgart.

The preligands used for Co(II)-imidazolium-phenoxyimine catalyst **C4** and Ni(II)-triazolium-phenoxyimine catalyst **C7** were synthesized following the literature known routes.[2-4]

2 General Procedures

2.1 GP1: Alkylation with Meerwein salt

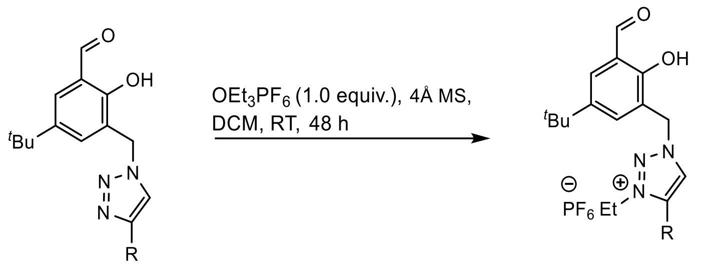

According to a literature procedure,[4,6] the triazoles (1.0 equiv.) were dissolved in dry DCM (0.5 mL/mmol) in the presence of molecular sieves (4Å) and triethyloxonium hexafluorophosphate (1.0 equiv.) was added as a stock solution in DCM (0.5 mL/mmol) at room temperature and stirred for 48 h. Subsequently, methanol was added to the reaction (5 mL) and the reaction was stirred for another 30 min at room temperature. Afterwards, the solvent was removed under reduced pressure, the solid was redissolved in DCM (5 mL) and washed with demineralized water (5 ml). The aqueous phase was washed with DCM and the combined organic layers were dried over $NaSO_4$ and the solvent was removed under reduced pressure. For further purification, the solid was dissolved in DCM (2 mL) and added dropwise for precipitation in *n*-pentane (40 mL). The solid was washed in the ultrasonic bath, centrifuged and the supernatant solution was decanted. This procedure was repeated two times, and the triazolium-precursor was dried in high-vacuum.

2.2 GP2: Imine condensation

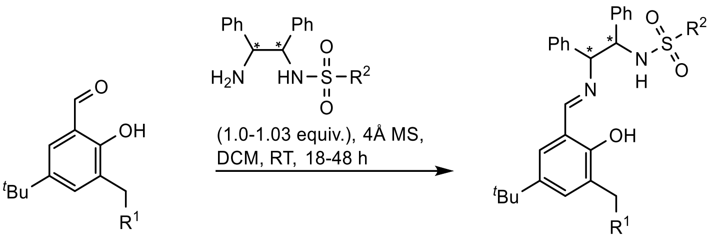

Using a slightly modified literature procedure,[2] the aldehyde (1.0 equiv.) and the sulfonamide (1.0-1.03 equiv.) were dissolved in dry DCM (1.0 mL/mmol) in the presence of molecular sieves (4Å) and stirred for 24-48 h at room temperature. Afterwards, the reaction mixture was diluted with DCM (5 mL), filtered over a small pad of celite and the filter cake was washed with DCM (5 mL). After removing the solvent under reduced pressure, for further purification the crude product was dissolved in DCM (1 mL) and added dropwise for precipitation in a mixture of *n*-pentane and diethylether (9:1, 40 mL). The solid was washed in the ultrasonic bath, centrifuged and the supernatant solution was decanted. This procedure was repeated two times, and the triazolium-preligand was dried in high-vacuum.

2.3 GP3: Complexation

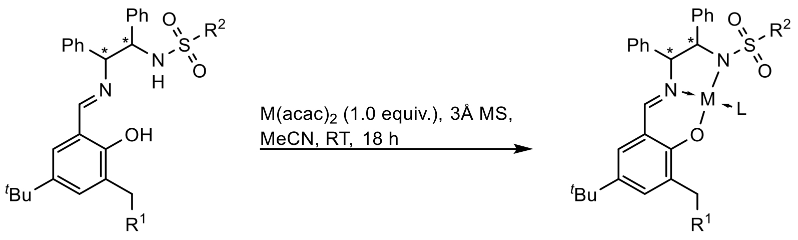

Using a slightly modified literature procedure,[2] the preligand (1.0 equiv.) and the metal(II)-source (1.0 equiv.) were dissolved in dry acetonitrile (10 mg preligand per mL solvent) in the presence of molecular sieves (3Å) and stirred for 18 h at room temperature. Afterwards, the reaction mixture was diluted with DCM (5 mL), filtered over a small pad of celite and the filter cake was washed with DCM (5 mL). After removing the solvent under reduced pressure, for further purification the crude product was dissolved in DCM (1 mL) and added dropwise for precipitation in of *n*-pentane (40 mL). The solid was washed in the ultrasonic bath, centrifuged and the supernatant solution was decanted. This procedure was repeated two times, and the phenoxyimine-complex was dried in high-vacuum.

2.4 GP4: Catalyst activation procedure

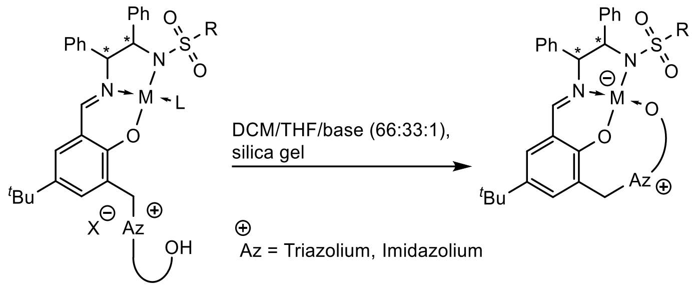

Using a slightly modified literature procedure,[2,5] a *Pasteur* pipette filled with silica gel was first rinsed with the activator solution of DCM:THF:base (66:33:1) (10 mL). The non-activated Lewis acid-phenoxyimine complex was then dissolved in the activator solution (3 mL) and applied to silica gel. The activated complex was then eluted with the activator solution (20 mL). After removing the solvent under reduced pressure, the solid was dried for 1 h under high vacuum and then freeze-dried three times with liquid nitrogen. Afterwards, dry *n*-pentane (5 mL) was added and the suspended solid was first washed in an ultrasonic bath, centrifuged, and the supernatant solution was decanted. After drying the complex for at least 3 h under high vacuum, it could be used directly in the catalytic reaction.

2.5 GP5: Synthesis of glycine-based iminoesters

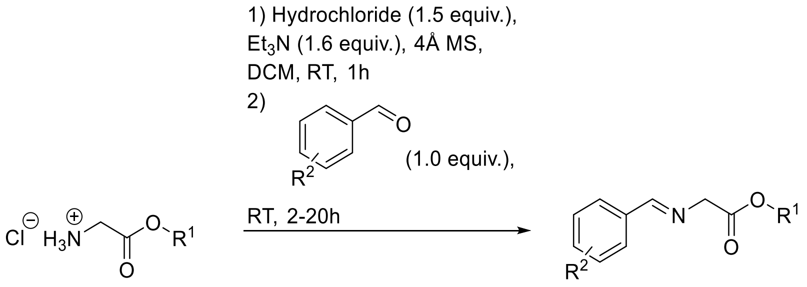

Using a modified literature procedure,[7] the hydrochloride (1.5 equiv.) was suspended in dry and degassed DCM (volumes in the respective procedures) with molecular sieves (4 Å). After the addition of triethylamine (1.6 equiv.) the reaction was stirred for 1 h at room temperature. Afterwards the aldehyde (1.0 equiv.) was added either directly (liquid aldehydes) or as a stock solution in dry, degassed DCM (1 mL), and the reaction mixture was stirred for 2–20 h at room temperature. Then, the solid reaction components were then filtered off, and the filter cake was rinsed with DCM (10 mL). The reaction mixture was diluted with DCM (50 mL) and washed with demineralized water (3 x 30 mL) and saturated sodium chloride solution (30 mL), dried over sodium sulfate, and coevaporated under reduced pressure with the addition of dry toluene (8 mL). After removing the toluene, dry DCM (1 mL) was added, and the crude product was filtered through a *Pasteur* pipette with oven-dried silica gel and eluted with additional dry DCM (1.5 mL). After removing the solvent under reduced pressure, the product was dried for a further 2 h under high vacuum. The product was then used in catalysis without further workup or frozen for storage at −24 °C under nitrogen atmosphere.

2.6 GP6: Catalytic asymmetric 1,3-dipolar cycloaddition of azomethine ylides to maleimides using *Lewis*-acid-phenoxyimine-complexes

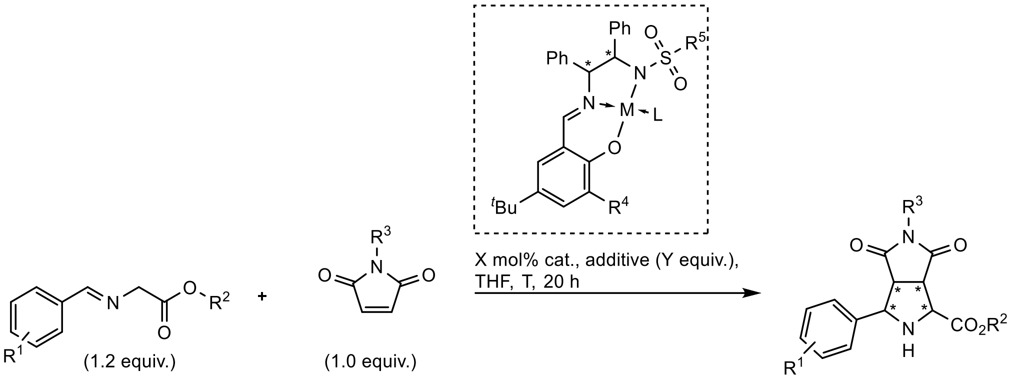

Using a modified literature procedure,[2] for the catalytic asymmetric 1,3-dipolar cycloaddition using *Lewis*-acid-phenoxyimine-complexes, the catalyst (X mol%) and maleimide (1.0 equiv.) were added to the synthesis tube of the *Heidolph Synthesis I* parallel synthesizer and then dried under high vacuum for 30 min. To start the reaction, the synthesis tube was set to the desired temperature, and then the imino ester (1.2 equiv.) was added as a tempered stock

solution in the mixture of THF and the additive (Y equiv.) and shaken at a rotation frequency of 250 rpm for 20 h. To terminate the reaction, the reaction mixture was diluted with a mixture of PE:EE (1:1, 2 mL), filtered through silica gel, and rinsed with PE:EE (1:1, 10 mL). After removal of the solvent under reduced pressure, mesitylene (25 µL) was added as an internal standard to determine the yield, conversion, and diastereomeric ratio by ^{1}H-NMR spectroscopy. The crude products obtained were further purified by column chromatography.

2.7 GP7: Catalytic asymmetric 1,3-dipolar cycloaddition of azomethine ylides to maleimides at low catalyst loadings (<0.1 mol%)

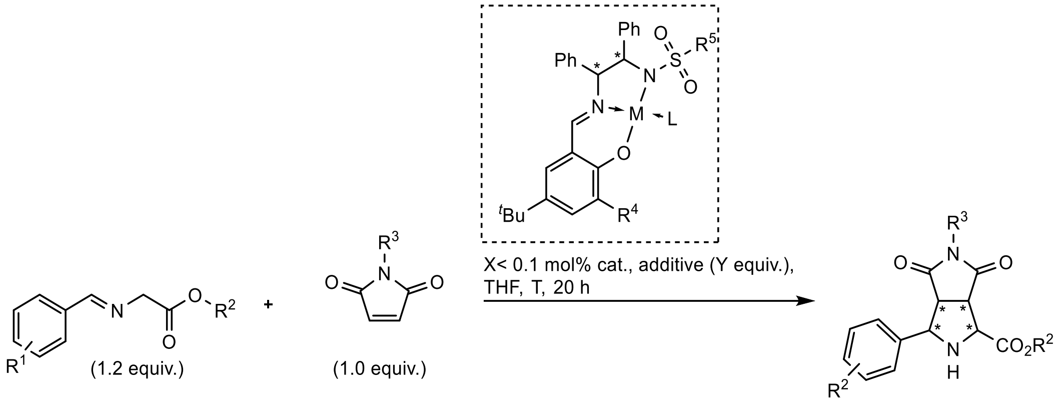

Using a modified literature procedure,[2,5] a stock solution of the complex (X<0.1 mol%) was first prepared in dry DCM. The concentration was chosen so that, with a sample weight of 0.5 mg of complex, the amount of substance required for the reaction was contained in 500 µL of dry DCM. Under nitrogen atmosphere, the appropriate amount of the stock solution was transferred into the synthesis tube of the *Heidolph Synthesis I* parallel synthesizer. The solvent was removed by flushing the reaction vessel with nitrogen, and the resulting complex was dried for a further 10 min under high vacuum. The maleimide (1.0 equiv.) was then added and dried for a further 30 min under high vacuum. To start the reaction, the synthesis tube was heated to the desired temperature, and then the imino ester (1.2 equiv.) was added as a tempered stock solution in the mixture of THF and the additive (Y equiv.) and shaken at a rotation frequency of 250 rpm for 20 h. To terminate the reaction, the reaction mixture was diluted with a mixture of PE:EE (1:1, 2 mL), filtered through silica gel, and rinsed with PE:EE (1:1, 10 mL). After removal of the solvent under reduced pressure, mesitylene (25 mL) was added as an internal standard to determine the yield, conversion, and diastereomeric ratio by ^{1}H-NMR spectroscopy. The crude products obtained were further purified by column chromatography.

2.8 GP8: Recycling of the *Lewis*-acid-phenoxyimine-complexes

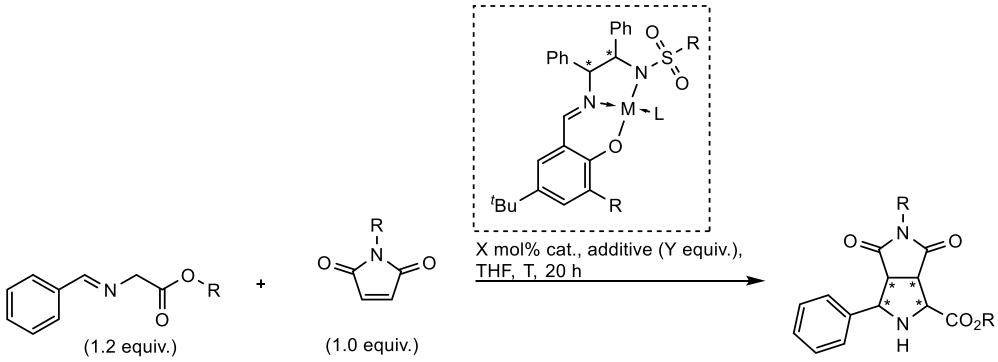

For the recycling of the polyfunctional *Lewis*-acid/betaine-complexes for the 1,3-dipolar cycloaddition, the complex (X mol%) and the maleimide (1.0 equiv.) were first weighed into the synthesis tube of the *Heidolph Synthesis I* parallel synthesis apparatus according to an adapted literature procedure[2] based on GP6 and then dried under high vacuum for 30 min. To start the reaction, the synthesis tube was set to the desired temperature, and then the imino ester (1.2 equiv.) was added as a tempered stock solution in the mixture of THF and the additive (Y equiv.) and shaken at a rotation frequency of 250 rpm for 20 h. To separate the catalyst after the reaction time, the solvent (THF) had to be removed under vacuum before the crude product was dissolved in a solvent mixture of PE:EE (1:1, 2 mL), filtered through silica gel and eluted with PE:EE (1:1, 10 mL). After removing the solvent under reduced pressure, mesitylene (25 µL) was added as an internal standard to determine the yield, conversion, and diastereomeric ratio by ^{1}H-NMR spectroscopy. The crude products obtained were further purified by column chromatography. The *Lewis*-acid-phenoxyimine-complex was then eluted from the silica gel with a mixture of THF:DIPEA/triethylamine (99:1, 10 mL), and the solvent was removed under reduced pressure. The complex was then freeze-dried three times with liquid nitrogen. In the final step, dry *n*-pentane (5 mL) was added. The suspended solid was first washed in an ultrasonic bath, centrifuged, and the supernatant solution was decanted. After the activated *Lewis*-acid-phenoxyimine-complex was dried for at least 3 h under high vacuum, the reactivated catalyst could be used in the next catalytic reaction.

3 Precursor Synthesis

3.1 Synthesis of (*R*)-1-(2'-Hydroxy-[1,1'-binaphthalen]-2-yl)-3-methyl-1*H*-imidazol-3-ium Iodide (CC6*HI)

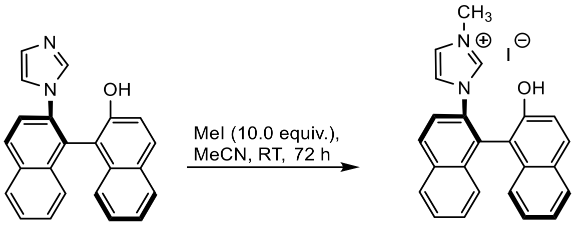

Using a modified literature procedure,[5] the imidazole starting material (33.1 mg, 98.3 μmol, 1.0 equiv.) was dissolved in dry acetonitrile (1.5 mL) at room temperature. After the addition of methyl iodide (61 μL, 980 μmol, 10.0 equiv.), the reaction was stirred for 72 h. Afterwards, demineralized water (2 mL) was added and stirred for a further 30 min. The reaction mixture was then extracted three times with DCM (5 mL each), and the combined organic phases were dried over sodium sulfate. After removal of the solvent under reduced pressure, the crude product was dissolved in DCM (2 mL) and precipitated in *n*-pentane (30 mL). The suspended solid was first washed in an ultrasonic bath, then centrifuged and decanted. The previously described purification method was repeated two more times, and the product was dried for 3 h under high vacuum. (*R*)-1-(2'-Hydroxy-[1,1'-binaphthalene]-2-yl)-3-methyl-1H-imidazol-3-ium iodide **CC6*HI** was obtained as a beige solid (25.7 mg, 53.7 μmol, 55%). The product was used without further purification.

$C_{24}H_{19}IN_2O$, MW: 478.33 g/mol. **MP:** 186.9 °C. **$[\alpha]^{20}_D$ (*c* = 1.0 mg/mL, DCM)**: +54.0°. **^{1}H-NMR (400 MHz, $CDCl_3$):** δ = *δ* 9.25 (s, 1H), 8.03 (d, *J* = 8.7 Hz, 1H), 7.93 (d, *J* = 8.4 Hz, 1H), 7.80–7.68 (m, 4H), 7.59–7.55 (m, 1H), 7.38–7.33 (*m*, 2H), 7.38 (*s*, 1H), 7.30–7.26 (*m*, 1H), 7.22-7.19 (*m*, 1H), 6.85 (*d*, *J* = 8.4 Hz, 1H), 6.83 (*t*, *J* = 1.7 Hz, 1H), 6.76 (*t*, *J* = 1.7 Hz, 1H), 3.90 (*s*, 3H).**^{13}C-NMR (176 MHz, $CDCl_3$):** *δ* = 152.5, 136.8, 134.1, 133.5, 133.0, 131.5, 131.1, 130.5, 128.7, 128.6, 128.6, 128.2, 128.2, 127.5, 127.4, 123.7, 123.6, 123.1, 122.8, 122.5, 119.6, 113.3, 77.3, 77.2, 77.0, 37.9. **IR ($CDCl_3$):** $\tilde{\nu}$ = 3145, 2197, 1623, 1548, 1508, 1432, 1344, 1270, 1208, 1098, 916, 817, 750, 728, 649. **HRMS (ESI) m/z:** Calculated for $C_{24}H_{19}N_2O$ ($[M]^+$): 351.1492. Measured: 351.1487.

3.2 Synthesis of 5-tert-Butyl-3-((3-ethyl-4-phenyl-triazol-3-ium-1-yl)-methyl)-2-hydroxy-benzaldehy Hexafluorophosphate (LP1)

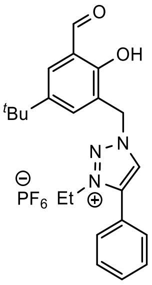

Synthesis of the triazolium-aldehyde precursor **LP1** was performed according to **GP1**.[4,6] Therefore, the triazol (50.8 mg, 0.15 mmol, 1.0 equiv.) and triethyloxonium hexafluorophosphate (37.6 mg, 0.15 mmol, 1.0 equiv.) were reacted. **LP1** was isolated as an off-white solid (70.2 mg, 0.15 mmol, >99%).

$C_{22}H_{26}F_6N_3O_2P$, MW: 509.43 g/mol. **MP:** >160 °C (Decomposition). **^{1}H-NMR (400 MHz, $CDCl_3$):** *δ* = 11.34 (*s*, 1H, Ar-O*H*), 9.93 (s, 1H, Ar-C*H*O), 8.16 (*s*, 1H, C=C*H*-N), 7.99 (*d*, *J* = 2.4 Hz, 1H, Ar-*H*), 7.69 (*d*, *J* = 2.5 Hz, 1H, Ar-*H*), 7.64-7.50 (*m*, 5H, Ar-*H*), 5.86 (*s*, 2H, Ar-CH_2-N), 4.55-4.48 (*q*, *J* = 7.4 Hz, 2H, N-CH_2-CH_3), 1.60 (*t*, *J* = 7.4 Hz, 3H, N-CH_2-CH_3), 1.38 (*s*, 9H, Ar-C(CH_3)$_3$). **^{13}C-NMR (176 MHz, $CDCl_3$):** *δ* = 196.9, 158.0, 144.5, 142.8, 137.6, 132.5, 132.1, 130.0, 129.7, 128.6, 122.0, 120.5, 118.9, 53.1, 47.5, 34.6, 31.3, 14.4. **^{19}F-NMR (376 MHz, $CDCl_3$):** *δ* = –74.5, –72.6 (*d*, *J* = 712.7 Hz, PF_6^-). **^{31}P-NMR (162 MHz, $CDCl_3$):** *δ* = –144.5 (*sept*, *J* = 712.2 Hz, PF_6^-). **IR (solid):** ṽ = 3166, 2961, 2873, 1654, 1467, 1275, 1220, 1009, 827, 766, 696, 555. **HRMS (ESI) m/z:** Calculated for $[M-PF_6]^+$ $C_{22}H_{32}N_3O_2^+$: 364.2020. Measured: 364.2024.

4 Ligand Synthesis

4.1 Synthesis of 3-(5-(*tert*-Butyl)-3-((*E*)-(((1*R*,2*R*)-1,2-diphenyl-2-((trifluoromethyl)sulfonamido)ethyl)imino)methyl)-2-hydroxybenzyl)-1-phenyl-1*H*-imidazol-3-ium Chloride (L1)

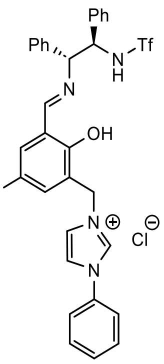

The synthesis of the phenolimine preligand **L1** was carried out according to **GP2**.[2] The aldehyde (30.0 mg, 80.0 µmol, 1.0 equiv.) and the sulfonamide (27.9 mg, 80.0 µmol, 1.0 equiv.) were reacted in dry DCM (3 mL). The phenolimine preligand **L1** was obtained as a yellow solid (55.1 mg, 78.0 µmol, 98%).

$C_{36}H_{36}ClF_3N_4O_3S$, MW: 697.21 g/mol. **MP:** >195 °C (Decomposition). **$[\alpha]^{20}_D$ (*c* = 1.0 mg/mL, DCM)**: +91.0°. **^{1}H-NMR (700 MHz, $CDCl_3$):** *δ* = 10.57 (*br*, 1H, Ar-O*H*), 8.31 (*s*, 1H, Ar-*H*), 8.13 (*s*, 1H, Ar-*H*), 8.01 (*s*, 1H, Ar-*H*), 7.56-7.43 (*m*, 7H, Ar-*H*), 7.32 (s, 1H, Ar-*H*), 7.25 (*s*, 1H, Ar-*H*), 7.18-7.09 (*m*, 6H, Ar-*H*), 7.05 (*m*, 2H, Ar-*H*), 6.95 (*s*, 1H, Ar-*H*), 5.96 (*d*, *J* = 13.7 Hz, 1H, CH_2), 5.48 (*m*, 2H, CH_2/CH), 4.99 (*d*, *J* = 10.7 Hz, 1H, C*H*), 1.09 (*s*, 9H, C(CH_3)$_3$). **^{13}C-NMR (125 MHz, $CDCl_3$):** δ = 167.4, 157.3, 141.7, 139.2, 138.3, 135.9, 134.8, 132.6, 130.5, 130.1, 129.5, 128.9, 128.5, 128.3, 128.1, 127.7, 127.5, 124.6, 122.0, 121.5, 119.5, 118.2, 114.4, 73.6, 65.7, 48.5, 34.1, 31.3 **^{19}F-NMR (376 MHz, $CDCl_3$):** δ = –77.84 (*s*). **IR (solid):** ṽ = 2963, 2868, 1630, 1600, 1370, 1226, 1192, 1147. **HRMS (ESI) m/z:** Calculated for: $C_{36}H_{36}F_3N_4O_3S$ [M]$^+$: 661.2455, Measured: 661.2455.

4.2 Synthesis of ((1*S*,2*S*)-2-((5-*tert*-Butyl-3-((3-ethyl-4-phenyl-triazol-3-ium-1-yl)-methyl)-2-hydroxy-phenyl)-methylenamino)-1,2-diphenyl-ethyl)-1,1,1-trifluoro-methanesulfonamide Hexafluorophosphate (L2)

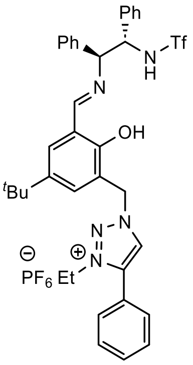

The synthesis of the phenolimine preligand **L2** was carried out according to **GP2**.[2] The aldehyde (28.2 mg, 0.06 mmol, 1.0 equiv.) and the sulfonamide (19.1 mg, 0.06 mmol, 1.0 equiv.) were reacted in dry DCM (3 mL). The phenolimine preligand **L2** was obtained as a yellow solid (36.3 mg, 0.06 mmol, >99%).

$C_{37}H_{39}F_9N_5O_3PS$, MW: 835.77 g/mol. **MP:** >170 °C (Decomposition). **$[\alpha]^{20}_D$ (*c* = 1.0 mg/mL, DCM)**: +73°. **^{1}H-NMR (500 MHz, $CDCl_3$):** *δ* = 13.77 (*br*, 1H, Ar-O*H*), 8.47 (*s*, 1H, Ar-C*H*=N), 8.29 (*s*,1H, C=C*H*-N), 7.54-7.42 (*m*, 6H, Ar-*H*), 7.37 (*d*, *J* = 2.4 Hz, 1H, Ar-*H*), 7.15-6.97 (*m*, 10H, Ar-*H*), 5.74 (*d*, *J* = 14.1 Hz, 1H, Ar-CH_2-N), 5.55 (*d*, *J* = 14.1 Hz, 1H, Ar-CH_2-N), 4.95 (*d*, *J* = 9.2 Hz, 1H,SO_2-C*H*), 4.68 (*d*, *J* = 9.2 Hz, 1H, Ar-C*H*=N), 4.44 (*q*, *J* = 7.3 Hz, 2H, N-CH_2-CH_3), 1.52 (*t*, *J* = 7.2 Hz, 2H, N-CH_2-CH_3), 1.22 (*s*, 9H, Ar-C(CH_3)$_3$). **^{13}C-NMR (125 MHz, $CDCl_3$):** *δ* = 157.6, 142.9, 138.5, 136.9, 132.0, 131.8, 131.5, 129.6, 129.5, 128.7, 128.6, 128.1, 127.9, 127.3, 121.9, 117.9, 77.2, 65.2, 53.8, 47.2, 34.2, 31.3, 14.3. **^{19}F-NMR (376 MHz, $CDCl_3$):** δ = –71.6, –73.5 (*d*, *J* = 712.3 Hz, PF_6^-), –77.9 (*s*, C*F*$_3$). **^{31}P-NMR (162 MHz, $CDCl_3$):** *δ* = –144.9 (*sept*, *J* = 712.2 Hz, PF_6^-).**IR ($CDCl_3$):** ṽ = 3327, 2957, 1632, 14560, 1337, 1229, 1196, 1146, 1046, 840, 698, 558. **HRMS (ESI) m/z:** Calculated for: $[M-PF_6]^+$ $C_{37}H_{39}F_3N_5O_3S^+$: 690.2720. Measured: 690.2724.

5 Syntheses of the Complexes

5.1 Synthesis of the Zn(II)-Precatalyst 3-(5-(*tert*-Butyl)-3-((((1*R*,2*R*)-1,2-diphenyl-2-((trifluoromethyl)sulfonamido)ethyl)imino)methyl)-2-oxybenzyl)-1-((*R*)-2'-hydroxy-[1,1'-binaphthalen]-2-yl)-1*H*-imidazole-3-ium-Zn(II) Chloride (C2*HCl)

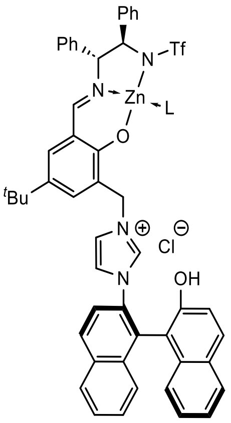

Synthesis of the Zn(II)-imidazolium-phenoxyimine-complex **C2*HCl** was performed according to **GP3**.[2] Therefore, the imidazolium-phenoxyimin-preligand (20.00 mg, 0.023 mmol, 1.0 equiv.) and Zn(acac)$_2$ (5.93 mg, 0.023 mmol, 1.0 equiv.) were reacted in dry acetonitrile (2 mL). The Zn(II)-imidazolium-phenoxyimine-complex **C2*HCl** was isolated as an off-white solid (18.70 mg, 0.020 mmol, 87%).

Due to the presence of multiple complex-species in CDCl$_3$, structural analysis by NMR-spectroscopy is impossible.

$C_{50}H_{42}ClF_3N_4O_4SZn$, MW: 952.80 g/mol. **MP**: 233 °C (decomposition). **[*α*]$^{20}_{D}$ (*c* = 1.0 mg/mL, DCM)**: –33.8°. **IR (CDCl$_3$):** ṽ = 3342, 3140, 3061, 2957, 1631, 1594, 1514, 1453, 1392, 1310, 1182, 1092, 1070, 816, 750, 701. **HRMS (ESI) m/z:** Calculated for $[M-Cl]^+$ **$C_{50}H_{42}ZnF_3N_4O_4S^+$**: 915.2144; Measured: 915.2169.
UV-Vis (DCM):

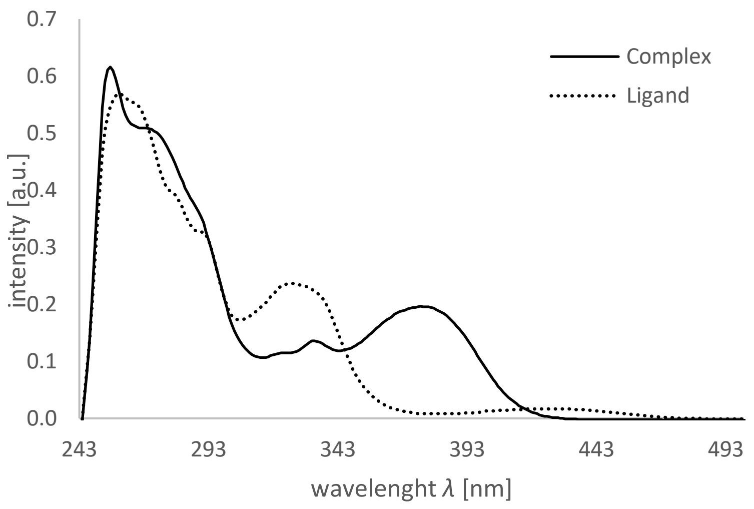

5.2 Synthesis of the Co(II)-Precatalyst 3-(5-(*tert*-Butyl)-3-((((1*S*,2*S*)-1,2-diphenyl-2-((trifluoromethyl)sulfonamido)ethyl)imino)methyl)-2-oxybenzyl)-1-((*R*)-2'-hydroxy-[1,1'-binaphthalen]-2-yl)-1*H*-imidazole-3-ium-Co(II) Chloride (C5*HCl)

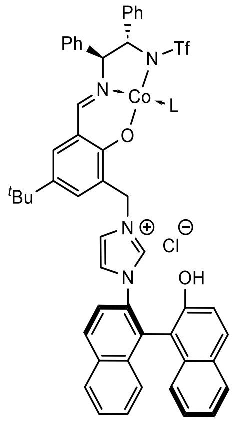

Synthesis of the Co(II)-imidazolium-phenoxyimin-complex **C5*HCl** was performed according to **GP3**.[2] Therefore, the imidazolium-phenoxyimine-preligand (35.3 mg, 0.0397 mmol, 1.0 equiv.) and Co(acac)$_2$ (10.2 mg, 0.0397 mmol, 1.0 equiv.) were reacted in dry acetonitrile (3 mL). The Co(II)-imidazolium-phenoxyimine-complex **C5*HCl** was isolated as a green solid (38.5 mg, 0.0399 mmol, <99%).

Structural analysis by NMR spectroscopy is not possible due to the resulting line broadening caused by the paramagnetic properties of the complex.

$C_{50}H_{42}ClCoF_3N_4O_4S$, MW: 946.35 g/mol. **MP**: >200 °C (decomposition). **$[\alpha]^{20}_D$ (*c* = 0.14 mg/mL, DCM)**: +142.9°. **IR (CDCl$_3$):** $\tilde{\nu}$ = 3061, 2960, 1624, 1544, 1510, 1453, 1435, 1365, 1309, 1274, 1178, 1095, 1066, 933, 816, 750, 700. **HRMS (ESI) m/z:** Calculated for $[M\text{-}Cl]^+$ **$C_{50}H_{42}CoF_3N_4O_4S^+$**: 910.2205; Measured: 910.2274.
UV-Vis (DCM):

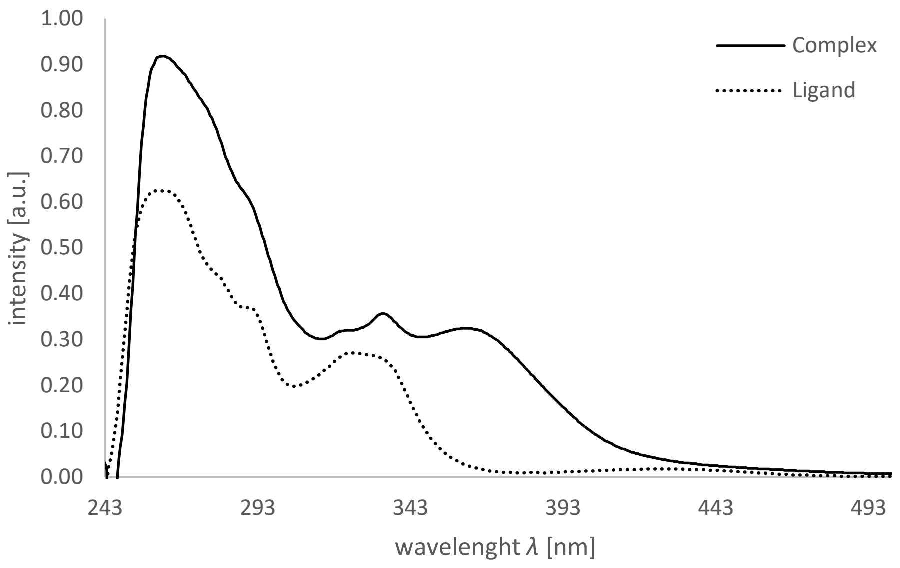

5.3 Synthesis of the Co(II)-Precatalyst 3-(5-(*tert*-Butyl)-3-((((1*R*,2*R*)-1,2-diphenyl-2-((trifluoromethyl)sulfonamido)ethyl)imino)methyl)-2-oxybenzyl)-1-((*R*)-2'-hydroxy-[1,1'-binaphthalen]-2-yl)-1*H*-imidazole-3-ium-Co(II) Chloride (C4*HCl)

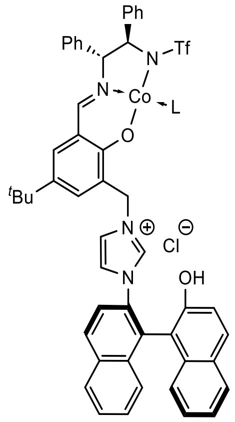

Synthesis of the Co(II)-imidazolium-phenoxyimin-complex **C4*HCl** was performed according to **GP3**.[2] Therefore, the imidazolium-phenoxyimine-preligand (46.6 mg, 0.0524 mmol, 1.0 equiv.) and Co(acac)$_2$ (13.1 mg, 0.0524 mmol, 1.0 equiv.) were reacted in dry acetonitrile (5 mL). The Co(II)-imidazolium-phenoxyimine-complex **C4*HCl** was isolated as a green solid (48.7 mg, 0.0515 mmol, 98%).

Structural analysis by NMR spectroscopy is not possible due to the resulting line broadening caused by the paramagnetic properties of the complex.

$C_{50}H_{42}ClCoF_3N_4O_4S$, MW: 946.35 g/mol. **MP**: >219 °C (Decomposition). **$[\alpha]^{20}_D$ (*c* = 1.0 mg/mL, DCM)**: +102.8°. **IR ($CDCl_3$):** $\tilde{\nu}$ = 3061, 2955, 1626, 1544, 1509, 1453,

1435, 1308, 1274, 1209, 1178, 1095, 1065, 923, 817, 751, 700, 618, 514, 425. **HRMS (ESI) m/z:** Calculated for [M-Cl]$^+$ **$C_{50}H_{42}CoF_3N_4O_4S^+$** : 910.2205; Measured: 910.2204.

UV-Vis (DCM):

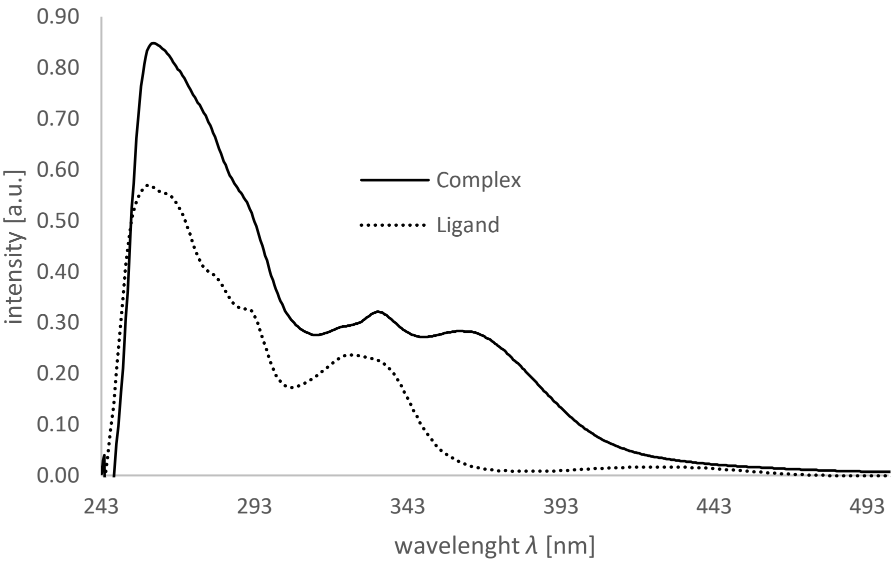

5.4 Synthesis of the Co(II)-Precatalyst (*R*)-2'-(3-(5-(*tert*-Butyl)-3-((*E*)-(((1*R*,2*R*)-1,2-diphenyl-2-((trifluoromethyl)sulfonamido)ethyl)imino)methyl)-2-oxybenzyl)-1*H*-imidazol-3-ium-1-yl)-[1,1'-binaphthalen]-2-olate-Cobalt(II) (C4)

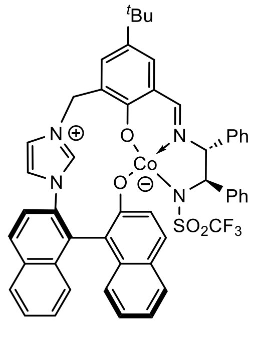

Synthesis of the Co(II)-imidazolium-phenoxyimine-complex **C4** was performed according to **GP4**.[2,5] Therefore, the non-activated complex **C4*HCl** (8.3 mg, 8.81 µmol.) was activated using the activator solvent of DCM:THF:DIPEA (66:33:1, 20 mL). The activated complex **C4** was isolated as a brown solid (5.8 mg, 6.37 µmol, 72%).

Structural analysis by NMR spectroscopy is not possible due to the resulting line broadening caused by the paramagnetic properties of the complex.

$C_{51}H_{44}F_3N_5NiO_4S$, MW: 938.69 g/mol. **MP:** >250 °C (Decomposition). **[*α*]$^{20}_D$ (*c* = 1.0 mg/mL, DCM)**: +48. **IR ($CDCl_3$):** ṽ = 3139, 3058, 2958, 2901, 2869, 1621, 1590, 1543, 1452, 1425,

1364,1320, 1246, 1209, 1172, 1095, 1066, 952, 931, 814, 748, 700, 600, 561, 512. **HRMS (ESI) m/z:** Calculated for $[M+H]^+$ $\mathbf{C_{50}H_{42}CoF_3N_4O_4S^+}$: 910.2205; Measured: 910.2207.

UV-Vis (DCM):

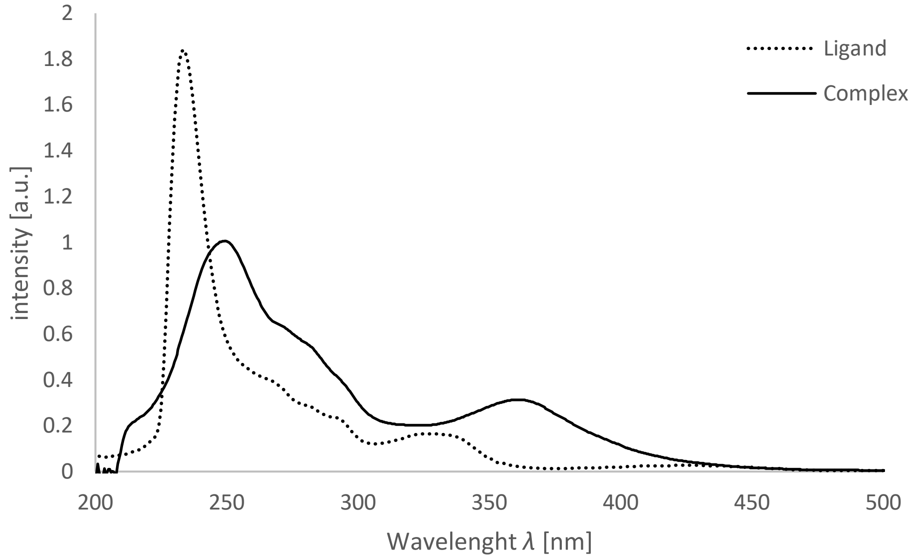

5.5 Synthesis of the Co(II)-Precatalyst 3-(5-(*tert*-Butyl)-3-((((1*S*,2*S*)-1,2-diphenyl-2-((trifluoromethyl)sulfonamido)ethyl)imino)methyl)-2-oxybenzyl)-1-((*S*)-2'-hydroxy-[1,1'-binaphthalen]-2-yl)-1*H*-imidazole-3-ium-Co(II) Chloride (*ent*-C4*HCl)

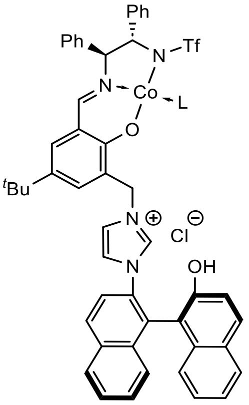

Synthesis of the Co(II)-imidazolium-phenoxyimine-complex ***ent*-C4*HCl** was performed according to **GP3**.[2] Therefore, the imidazolium-phenoxyimine-preligand (24.6 mg, 27.6 µmol, 1.0 equiv.) and $Co(acac)_2$ (7.1 mg, 27.6 µmol, 1.0 equiv.) were reacted in dry acetonitrile (3 mL). The Co(II)-imidazolium-phenoxyimine-complex ***ent*-C4*HCl** was isolated as a green solid (18.0 mg, 19.0 µmol, 69%).

Structural analysis by NMR spectroscopy is not possible due to the resulting line broadening caused by the paramagnetic properties of the complex.

$C_{50}H_{42}ClCoF_3N_4O_4S$, MW: 946.35 g/mol. **MP**: >212 °C (Decomp). **$[\alpha]^{20}_D$ (c = 1.0 mg/mL, DCM)**: −84°. **IR ($CDCl_3$):** ṽ = 3140, 3061, 3029, 2961, 2905, 2870, 2253, 2203, 1988, 1962, 1708, 1625, 1543, 1513, 1453, 1435, 1390, 1309, 1274, 1248, 1210, 1181, 1095, 1067, 1027, 970, 910, 817, 750, 701, 646, 604, 515,479, 422. **HRMS (ESI) m/z:** Calculated for $[M-Cl]^+$ **$C_{50}H_{42}CoF_3N_4O_4S^+$**: 910.2205; Measured: 910.2189.

UV-Vis (DCM):

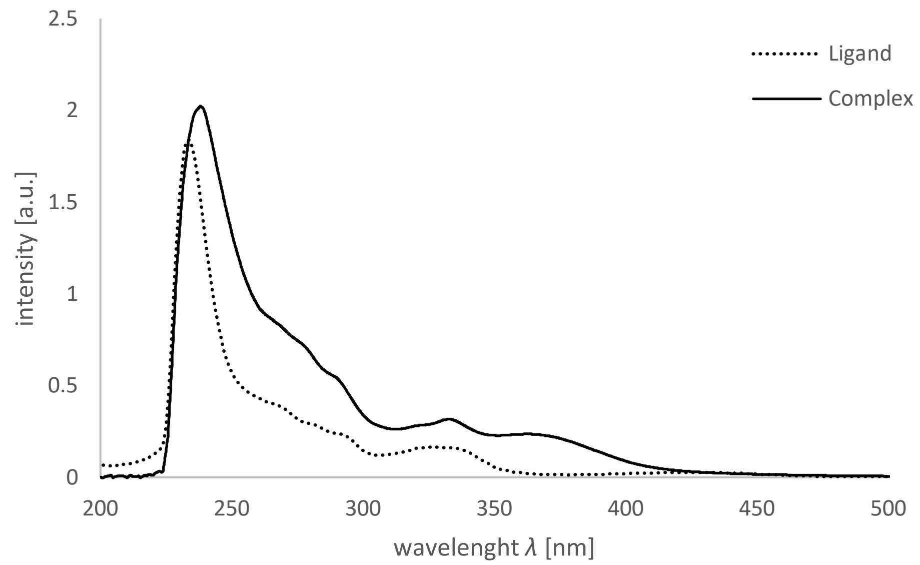

5.6 Synthesis of the Co(II)-Precatalyst (1*R*,2*R*)-2-((5-(*tert*-Butyl)-2-oxy-3-((*R*)-((2'-oxy-[1,1'-binaphthalen]-2-yl)oxy)methyl)benzylidene)amino)-1,2-diphenylethyl)-1,1,1-trifluoromethane-sulfonamide-Co(II) (CC2*H)

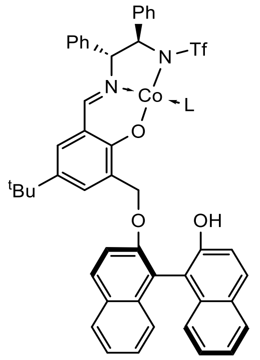

Synthesis of the Co(II)-phenoxyimine-complex **CC2*H** was performed according to **GP3**.[2] Therefore, the imidazolium-phenoxyimine-preligand (42.7 mg, 53.1 µmol, 1.0 equiv.) and $Co(acac)_2$ (13.7 mg, 53.1 µmol, 1.0 equiv.) were reacted in dry acetonitrile (4 mL). The Co(II)-imidazolium-phenoxyimin-complex **CC2*H** was isolated as a brown solid (20.7 mg, 24.1 µmol, 45%).

Due to the presence of multiple complex-species in $CDCl_3$, structural analysis by NMR-spectroscopy is impossible.

$C_{47}H_{39}CoF_3N_2O_5S$, MW: 859.83 g/mol. **MP.**: >166 °C (Decomposition). **[α]$^{20}_{D}$ (*c* = 1.0 mg/mL, DCM)**: +70°. **IR ($CDCl_3$):** $\tilde{\nu}$ = 3533, 3060, 3029, 2961, 2925, 2866, 2163, 2020, 1961, 1621,1591, 1519, 1456, 1381, 1307, 1270, 1187, 1146, 1069, 1020, 933, 815, 749, 700, 617, 518, 417. **HRMS (ESI) m/z:** Calculated for [M] **$C_{47}H_{39}CoF_3N_2O_5S$**: 859.1864; Measured: 859.1864.

UV-Vis (DCM):

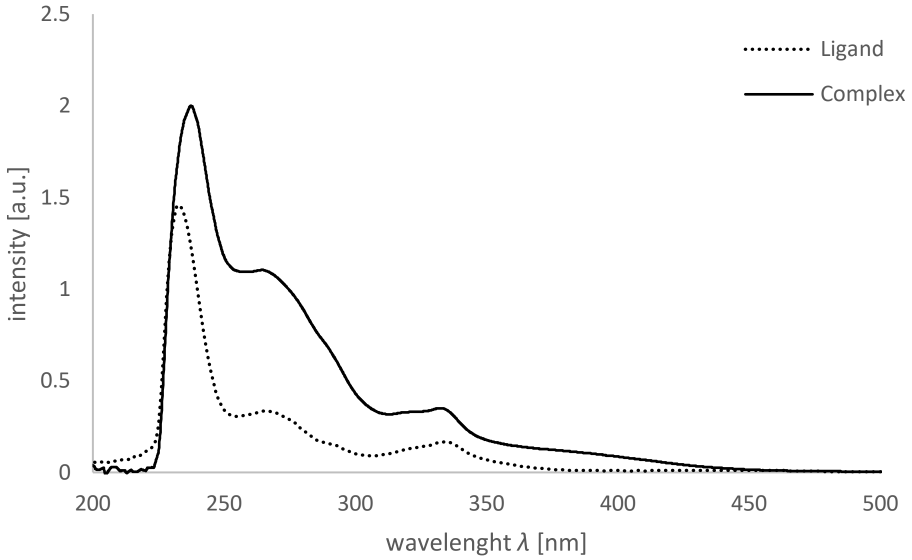

5.7 Synthesis of the Co(II)-Precatalyst 3-(5-(*tert*-Butyl)-3-((((1*R*,2*R*)-1,2-diphenyl-2-((trifluoromethyl)sulfonamido)ethyl)imino)methyl)-2-oxybenzyl)-1-(2-hydroxyphenyl)-1H-imidazol-3-ium-Co(II) Chloride (CC3*HCl)

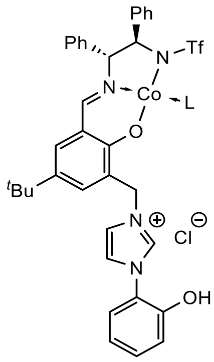

Synthesis of the Co(II)-imidazolium-phenoxyimine-complex **CC3*HCl** was performed according to **GP3**.[2] Therefore, the imidazolium-phenoxyimine-preligand (41.8 mg, 58.6 µmol, 1.0 equiv.) and $Co(acac)_2$ (15.1 mg, 58.6 µmol, 1.0 equiv.) were reacted in dry acetonitrile

(4 mL). The Co(II)-imidazolium-phenoxyimine-complex **CC3*HCl** was isolated as a green solid (14.8 mg, 19.2 µmol, 33%).

Structural analysis by NMR spectroscopy is not possible due to the resulting line broadening caused by the paramagnetic properties of the complex.

$C_{36}H_{34}ClCoF_3N_4O_4S$, MW: 770.13 g/mol. **MP**: >221 °C (Decomposition). **[α]$^{20}_{D}$ (c = 1.0 mg/mL, DCM)**: –2°. **IR ($CDCl_3$):** ṽ = 3756, 3152, 3063, 3029, 2961, 2868, 2264, 1979, 1626, 1603, 1547, 1494, 1365, 1304, 1277, 1210, 1180, 1115, 1068, 992, 966, 931, 911, 831, 799, 757, 732, 701, 618, 514. **HRMS (ESI) m/z:** Calculated for $[M-Cl]^+$ **$C_{36}H_{34}CoF_3N_4O_4S^+$**: 734.1579; Measured: 734.1572.
UV-Vis (DCM):

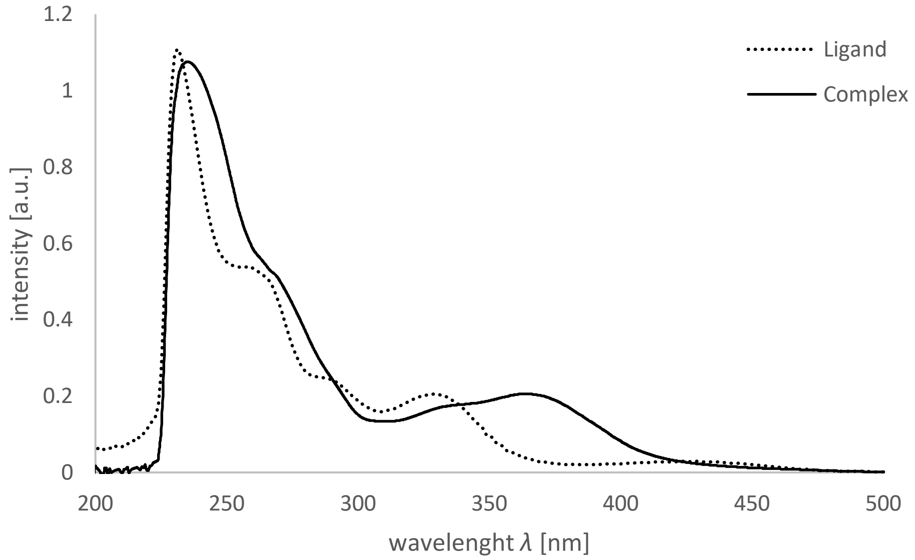

5.8 Synthesis of the Co(II)-Precatalyst 3-(5-(*tert*-Butyl)-3-((((1*R*,2*R*)-1,2-diphenyl-2-((trifluoromethyl)sulfonamido)ethyl)imino)methyl)-2-oxybenzyl)-1-phenyl-1*H*-imidazol-3-ium-Co(II) Chloride (CC4)

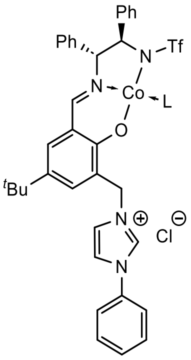

Synthesis of the Co(II)-imidazolium-phenoxyimine-complex **CC4** was performed according to **GP3**.[2] Therefore, the imidazolium-phenoxyimine-preligand **L1** (43.4 mg, 62.3 µmol,

1.0 equiv.) and Co(acac)$_2$ (16.0 mg, 62.3 µmol, 1.0 equiv.) were reacted in dry acetonitrile (4 mL). The Co(II)-imidazolium-phenoxyimine-complex **CC4** was isolated as a green solid (2.2 mg, 2.9 µmol, 5%).

Structural analysis by NMR spectroscopy is not possible due to the resulting line broadening caused by the paramagnetic properties of the complex.

$C_{36}H_{34}ClCoF_3N_4O_3S$, MW: 754.13 g/mol. **MP.:** >157 °C (Decomposition). **$[\alpha]^{20}_D$ (*c* = 1.0 mg/mL, DCM)**: −116°. **IR (CDCl$_3$):** $\tilde{\nu}$ = 3858, 3789, 3659, 3515, 3128, 2959, 2925, 2879, 2854, 2338, 2250, 2204, 2163, 2049, 1989, 1903, 1723, 1627, 1547, 1494, 1452, 1390, 1366, 1308, 1210, 1178, 1069, 1021, 931, 836, 799, 761, 730, 701, 617, 511, 476, 427. **HRMS (ESI) m/z:** Calculated for $[M\text{-}Cl]^+$ **$C_{36}H_{34}CoF_3N_4O_3S^+$**: 718.1630; Measured: 718.1619. **UV-Vis (DCM):**

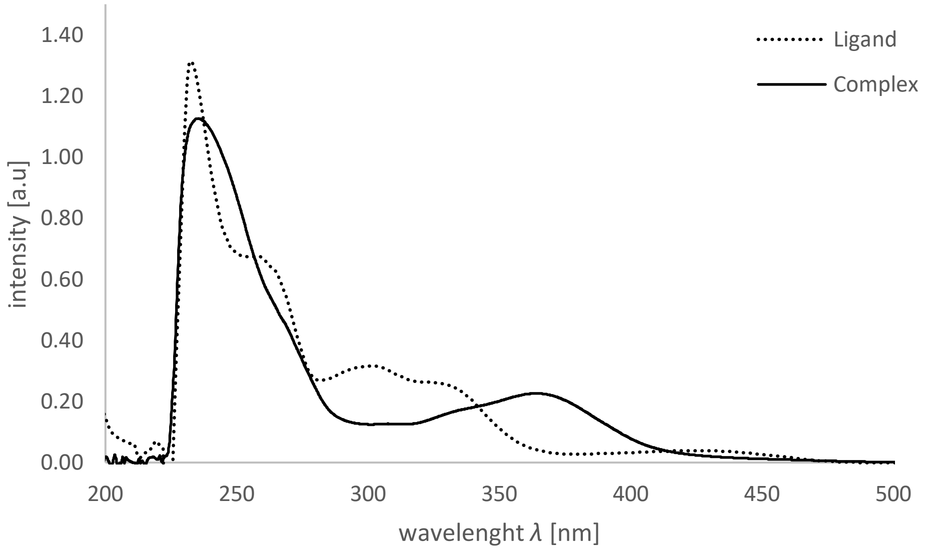

5.9 Synthesis of the Co(II)-Precatalyst ((1*R*,2*R*)-2-((5-(*tert*-Butyl)-2-oxy-3-methylbenzylidene)amino)-1,2-diphenylethyl)-1,1,1-trifluoromethane-sulfonamide-Co(II) (CC5)

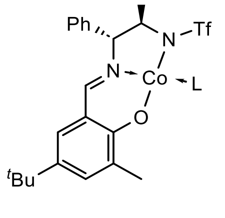

Synthesis of the Co(II)-imidazolium-phenoxyimine-complex **CC5** was performed according to **GP3**.[2] Therefore, the imidazolium-phenoxyimine-preligand (29.6 mg, 57.1 µmol, 1.0 equiv.) and Co(acac)$_2$ (14.7 mg, 57.1 µmol, 1.0 equiv.) were reacted in dry acetonitrile (3 mL). The

Co(II)-imidazolium-phenoxyimine-complex **CC5** was isolated as a brown solid (14.6 mg, 25.4 mmol, 87%).

Structural analysis by NMR spectroscopy is not possible due to the resulting line broadening caused by the paramagnetic properties of the complex.

$C_{27}H_{27}CoF_3N_2O_3S$, MW: 575.51 g/mol. **MP.**: >220 °C (Decomposition). **$[\alpha]^{20}_D$ (c = 1.0 mg/mL, DCM)**: −22°. **IR ($CDCl_3$):** ṽ = 3734, 3610, 2964, 2910, 2865, 2237, 2159, 2140, 2007, 1962, 1931, 1623, 1588, 1523, 1455, 1384, 1324, 1269, 1184, 1069, 925, 763, 698, 610. **HRMS (ESI) m/z:** Calculated for [M] **$C_{27}H_{27}CoF_3N_2O_3S$**: 575.1026; Measured: 575.1019.
UV-Vis (DCM):

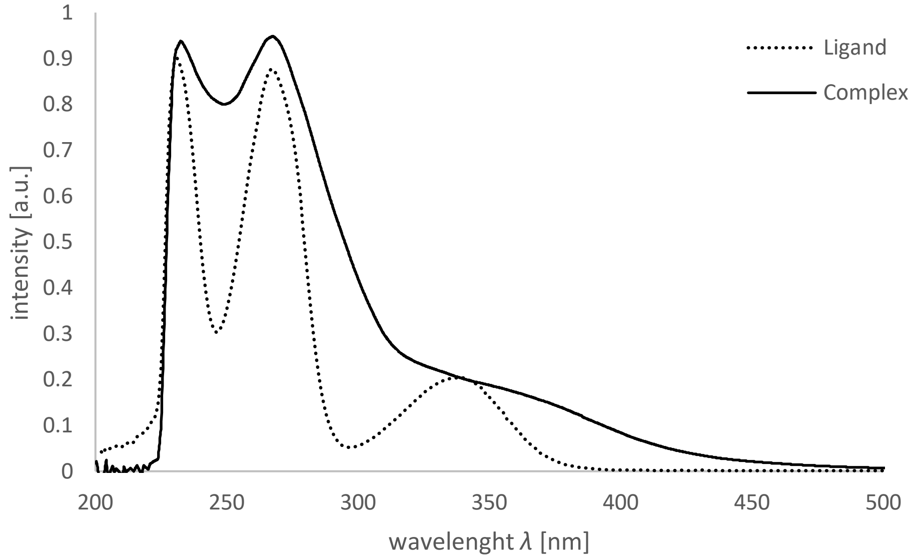

5.10 Synthesis of the Ni(II)-Precatalyst 1-(5-(*tert*-Butyl)-3-((((1*S*,2*S*)-1,2-diphenyl-2-((trifluoromethyl)sulfonamido)ethyl)imino)methyl)-2-oxybenzyl)-3-ethyl-4-((*R*)-2'-hydroxy-[1,1'-binaphthalen]-2-yl)-1*H*-1,2,3-triazol-3-ium-Ni(II) Hexafluorophosphate(V) (C7*HPF6)

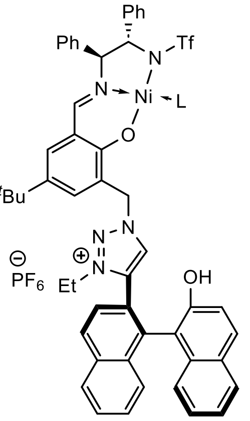

Synthesis of the Ni(II)-triazolium-phenoxyimine-complex **C7*HPF$_6$** was performed according to **GP3**.[2] Therefore, the triazolium-phenoxyimin-preligand (20.1 mg, 20 µmol, 1.0 equiv.) and Ni(acac)$_2$ (5.0 mg, 20 µmol, 1.0 equiv.) were reacted in dry acetonitrile (3 mL). The Ni(II)-triazolium-phenoxyimine-complex **C7*HPF$_6$** was isolated as a brown solid (21.4 mg, 20 µmol, >99%).

The largest scale experiment gave 125.8 mg (0.116 mmol, 92% yield).

Structural analysis by NMR spectroscopy is not possible due to the resulting line broadening caused by the paramagnetic properties of the complex.

$C_{51}H_{45}NiF_9N_5O_4PS$, MW: 1084.90 g/mol. **Mp:** >200 °C (Decomposition). **$[\alpha]^{20}_D$ (c = 0.1 g/dL, DCM):** −11°. IR (CDCl$_3$): $\tilde{\nu}$ = 2966, 1622, 1556, 1453, 1323, 1274, 1186, 1072, 845. **HRMS (ESI) m/z:** Calculated for $[M\text{-}PF_6]^+$ **$C_{51}H_{45}NiF_3N_5O_4S^+$**: 938.2492. Measured: 938.2494.

UV-Vis (DCM):

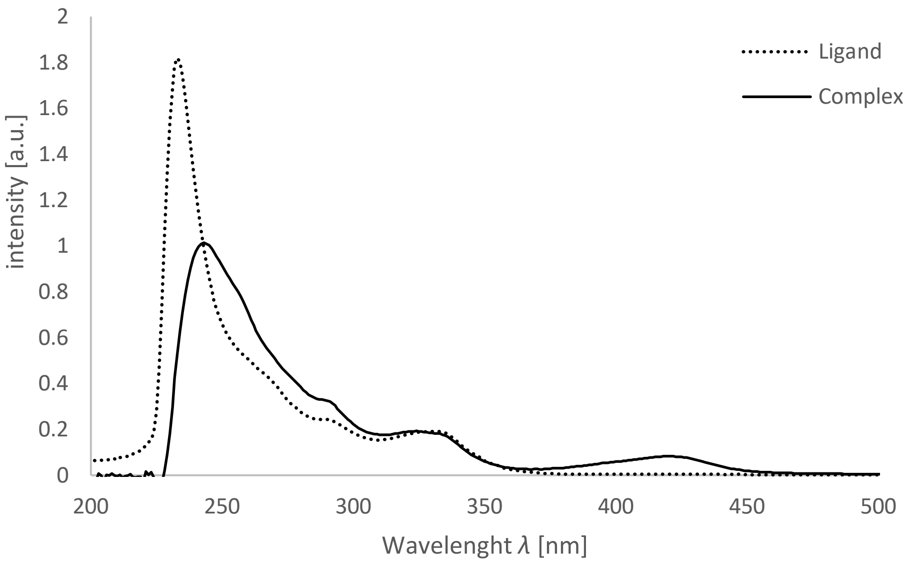

5.11 Synthesis of the Ni(II)-Precatalyst (*R*)-2'-(1-(5-(*tert*-Butyl)-3-((*E*)-(((1*S*,2*S*)-1,2-diphenyl-2-((trifluoromethyl)sulfonamido)ethyl)imino)methyl)-2-oxybenzyl)-3-ethyl-1*H*-1,2,3-triazol-3-ium-4-yl)-[1,1'-binaphthalen]-2-olate-nickel(II) (C7)

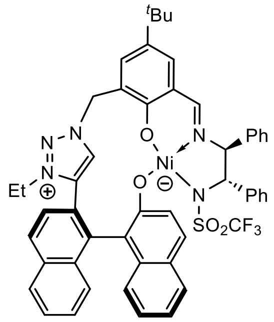

Synthesis of the Ni(II)triazolium-phenoxyimine-complex **C7** was performed according to **GP4**.[2,5] Therefore, the non-activated complex **C7*HPF$_6$** (20.0 mg, 0.0184 mmol.) was activated using the activator solvent of DCM:THF:Et$_3$N (66:33:1, 20 mL). The activated complex **C7** was isolated as a brown solid (14.9 mg, 0.0159 mmol, 86%).

Structural analysis by NMR spectroscopy is not possible due to the resulting line broadening caused by the paramagnetic properties of the complex.

$C_{51}H_{44}F_3N_5NiO_4S$, MW: 938.69 g/mol. **MP.**: >204 °C (Decomposition). **$[\alpha]^{20}_D$ (*c* = 1.0 mg/mL, DCM)**: −36. **IR (CDCl$_3$):** $\tilde{\nu}$ = 3499, 3026, 2961, 2872, 1622, 1602, 1550, 1453, 1325, 1276, 1210, 1180, 1072, 1008, 955, 844, 771, 753, 704, 613, 587, 516. **HRMS (ESI) m/z:** Calculated for $[M+H]^+$ **$C_{51}H_{45}F_3N_5NiO_4S^+$**: 938.2492; Measured: 938.2487.

UV-Vis (DCM):

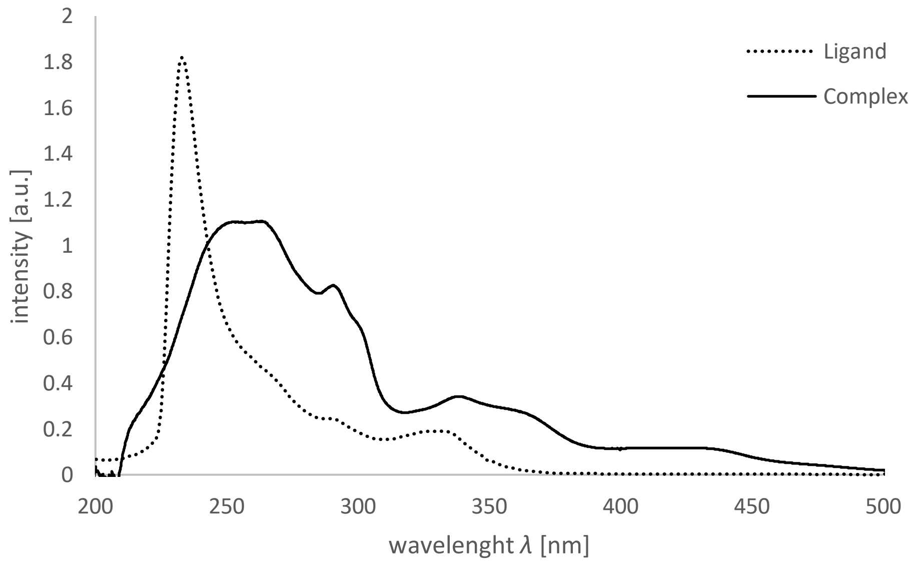

5.12 Synthesis of the Ni(II)-Precatalyst 1-(5-(*tert*-Butyl)-3-((((1*R*,2*R*)-1,2-diphenyl-2-((trifluoromethyl)sulfonamido)ethyl)imino)methyl)-2-oxybenzyl)-3-ethyl-4-((*R*)-2'-hydroxy-[1,1'-binaphthalen]-2-yl)-1*H*-1,2,3-triazol-3-ium-Ni(II) Hexafluorophosphate(V) (C6*HPF$_6$)

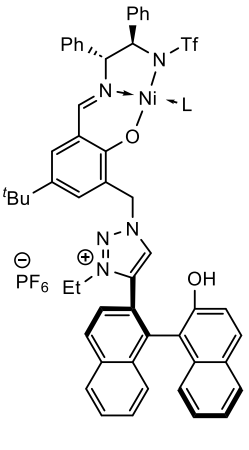

Synthesis of the Ni(II)-triazolium-phenoxyimine-complex **C6*HPF$_6$** was performed according to **GP3**.[2] Therefore, the triazolium-phenoxyimin-preligand (22.1 mg, 20 µmol, 1.0 equiv.) and Ni(acac)$_2$ (5.5 mg, 20 µmol, 1.0 equiv.) were reacted in dry acetonitrile (2 mL). The Ni(II)-triazolium-phenoxyimine-complex **C6*HPF$_6$** was isolated as a brown solid (23.1 mg, 20 µmol, >99%).

Structural analysis by NMR spectroscopy is not possible due to the resulting line broadening caused by the paramagnetic properties of the complex.

$C_{51}H_{45}NiF_9N_5O_4PS$, MW: 1084.90 g/mol. **Mp:** >197 °C (Decomposition). **$[\alpha]^{20}_D$ (c = 0.1 g/dL, DCM):** +63°. **IR ($CDCl_3$):** $\tilde{\nu}$ = 3450, 2955, 20221623, 1553, 1452, 1326, 1273, 1185, 1147, 547, 559. **HRMS (ESI) m/z:** Calculated for $[M\text{-}PF_6]^+$ **$C_{51}H_{45}NiF_3N_5O_4S^+$**: 938.2492. Measured: 938.2492.

UV-Vis (DCM):

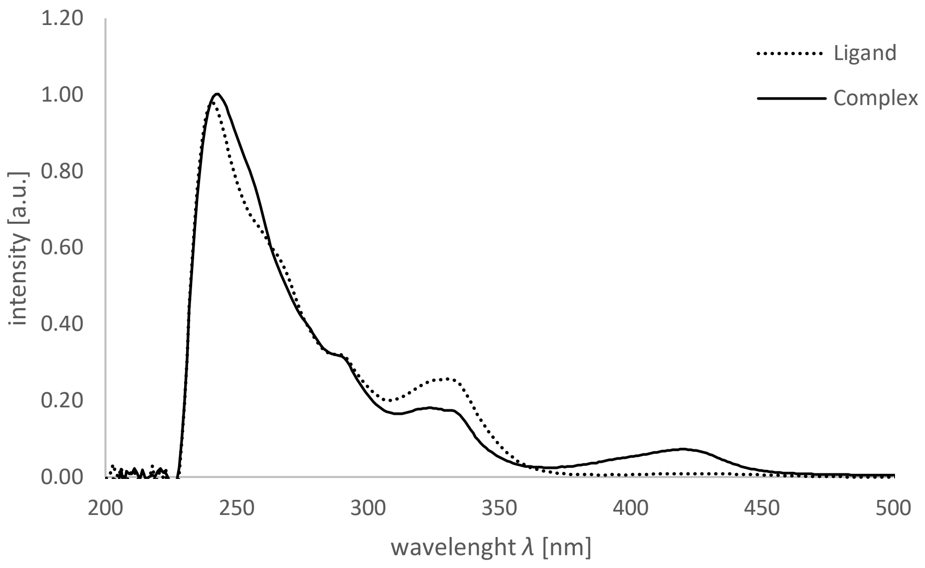

5.13 Synthesis of the Co(II)-Precatalyst 1-(5-(*tert*-Butyl)-3-((((1*S*,2*S*)-1,2-diphenyl-2-((trifluoromethyl)sulfonamido)ethyl)imino)methyl)-2-oxybenzyl)-3-ethyl-4-((*R*)-2'-hydroxy-[1,1'-binaphthalen]-2-yl)-1*H*-1,2,3-triazol-3-ium-Co(II) Hexafluorophosphate(V) (CC1*HPF$_6$)

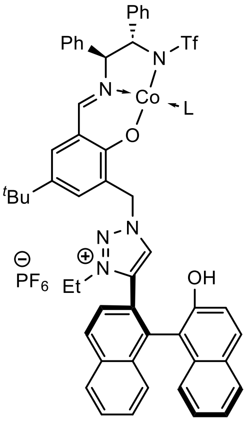

Synthesis of the Co(II)-imidazolium-phenoxyimine-complex **CC1*HPF$_6$** was performed according to **GP3**.[2] Therefore, the imidazolium-phenoxyimine-preligand (100.0 mg, 0.10 mmol, 1.0 equiv.) and Co(acac)$_2$ (17.2 mg, 0.10 mmol, 1.0 equiv.) were reacted in dry acetonitrile (10 mL). The Co(II)-imidazolium-phenoxyimine-complex **CC1*HPF$_6$** was isolated as a brown solid (105.0 mg, 0.10 mmol, >99%).

Structural analysis by NMR spectroscopy is not possible due to the resulting line broadening caused by the paramagnetic properties of the complex.

$C_{51}H_{45}CoF_9N_5O_4PS$, MW: 1084.90 g/mol. **MP.**: >235 °C (Decomposition). **[α]$^{20}_D$ (*c* = 1.0 mg/mL, DCM)**: +31°. **IR (CDCl$_3$):** $\tilde{\nu}$ = 3525, 2956, 1627, 1602, 1455, 1377, 1228, 1188, 1145, 1059, 844, 699, 559. **HRMS (ESI) m/z:** Calculated for [M-PF$_6$]$^+$

$C_{51}H_{45}CoF_3N_5O_4S^+$: 939.2471; Measured: 939.2476. Calculated for PF_6^-: 144.96. Measured: 144.96.

UV-Vis (DCM):

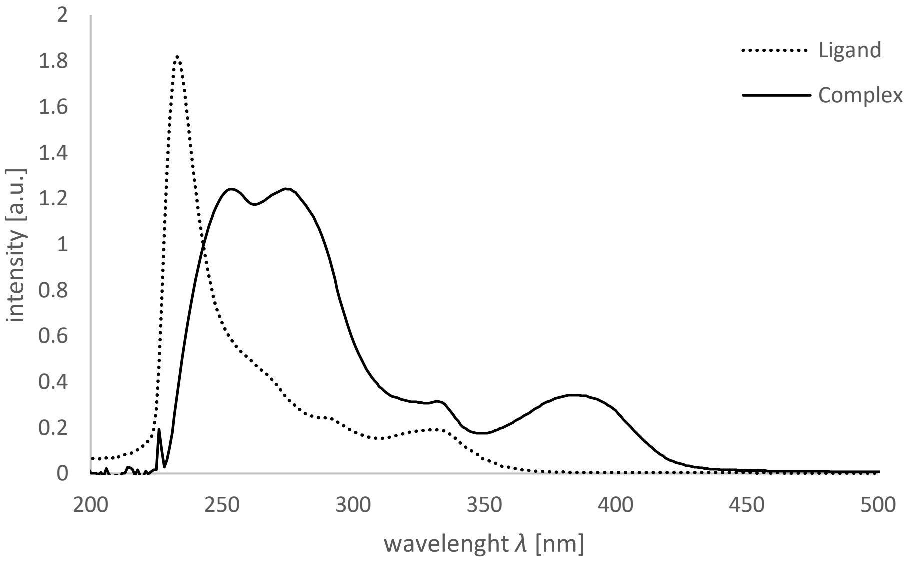

5.14 Synthesis of the Ni(II)-Precatalyst 1-(5-(*tert*-Butyl)-3-((((1*R*,2*R*)-1,2-diphenyl-2-((trifluoromethyl)sulfonamido)ethyl)imino)methyl)-2-oxybenzyl)-3-ethyl-4-((*S*)-2'-hydroxy-[1,1'-binaphthalen]-2-yl)-1*H*-1,2,3-triazol-3-ium-Ni(II) Hexafluorophosphate(V) (*ent*-C7*HPF$_6$)

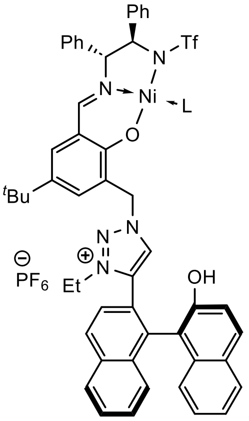

Synthesis of the Ni(II)-triazolium-phenoxyimine-complex ***ent*-C7*HPF$_6$** was performed according to **GP3**.[2] Therefore, the triazolium-phenoxyimine-preligand (62.3 mg, 60.6 µmol, 1.0 equiv.) and Ni(acac)$_2$ (15.6 mg, 60.6 µmol, 1.0 equiv.) were reacted in dry acetonitrile (6 mL). The Ni(II)-triazolium-phenoxyimine-complex ***ent*-C7*HPF$_6$** was isolated as a yellow solid (23.4 mg, 21.6 µmol, 36%).

Structural analysis by NMR spectroscopy is not possible due to the resulting line broadening caused by the paramagnetic properties of the complex.

$C_{51}H_{45}F_9N_5NiO_4PS$, MW: 1084.66 g/mol. **Mp.**: >186 °C (Decomposition). **[α]$^{20}_{D}$ (c = 1.0 mg/mL, DCM)**: +162°. **IR ($CDCl_3$):** $\tilde{\nu}$ = 3515, 3059, 3029, 2960, 2929, 2851, 2240, 2033, 2019, 1975, 1622, 1603, 1554, 1506, 1453, 1395, 1365, 1323, 1274, 1211, 1187, 1147, 1073, 1009, 965, 848, 772, 753, 698, 646, 558, 520, 467, 428. **HRMS (ESI) m/z:** Calculated for $[M\text{-}PF_6]^+$ **$C_{51}H_{45}F_3N_5NiO_4S^+$**: 938.2492; Measured: 938.2484.

UV-Vis (DCM):

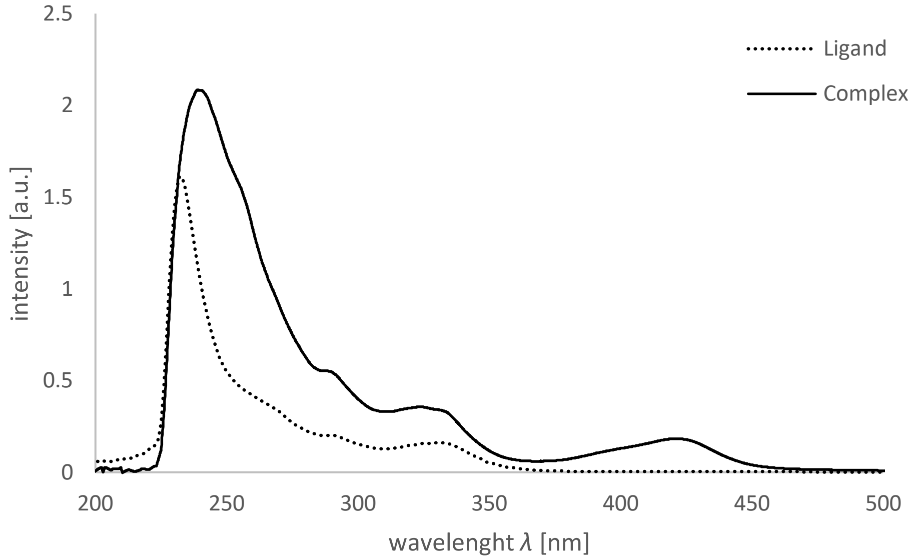

5.15 Synthesis of the Ni(II)-Precatalyst 1-(5-(*tert*-Butyl)-3-((((1*R*,2*R*)-1,2-diphenyl-2-(methylsulfonamido)ethyl)imino)methyl)-2-oxybenzyl)-3-ethyl-4-((*R*)-2'-hydroxy-[1,1'-binaphthalen]-2-yl)-1*H*-1,2,3-triazol-3-ium-Ni(II) Hexafluorophosphate(V) (CC8*HPF6)

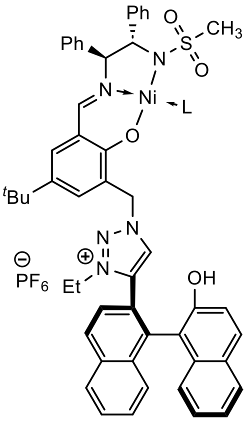

Synthesis of the Ni(II)-triazolium-phenoxyimine-complex **CC8*HPF6** was performed according to **GP3**.[2] Therefore, the triazolium-phenoxyimine-preligand (13.6 mg, 139.9 µmol, 1.0 equiv.) and $Ni(acac)_2$ (3.6 mg, 139.9 µmol, 1.0 equiv.) were reacted in dry acetonitrile (2 mL). The Ni(II)-triazolium-phenoxyimine-complex **CC8*HPF6** was isolated as a green solid (2.9 mg, 28.1 µmol, 20%).

Structural analysis by NMR spectroscopy is not possible due to the resulting line broadening caused by the paramagnetic properties of the complex.

$C_{51}H_{48}F_6N_5NiO_4PS$, MW: 1030.69 g/mol. **Mp.**: >256 °C (Decomposition). **[α]$^{20}_{D}$ (c = 1.0 mg/mL, DCM)**: −10°. **IR ($CDCl_3$):** ṽ = 3915, 3854, 3762, 3687, 3632, 3471, 3367, 3275, 3207, 3147, 2954, 2917, 2849, 2356, 2271, 2174, 2031, 1979, 1747, 1622, 1556, 1458, 1435, 1345, 1275, 1226, 1115, 1011, 979, 845, 752, 701, 559, 492, 440. **HRMS (ESI) m/z:** Calculated for [M-PF_6] **$C_{51}H_{48}N_5NiO_4S^+$**: 884.2775; Measured: 884.2784.
UV-Vis (DCM):

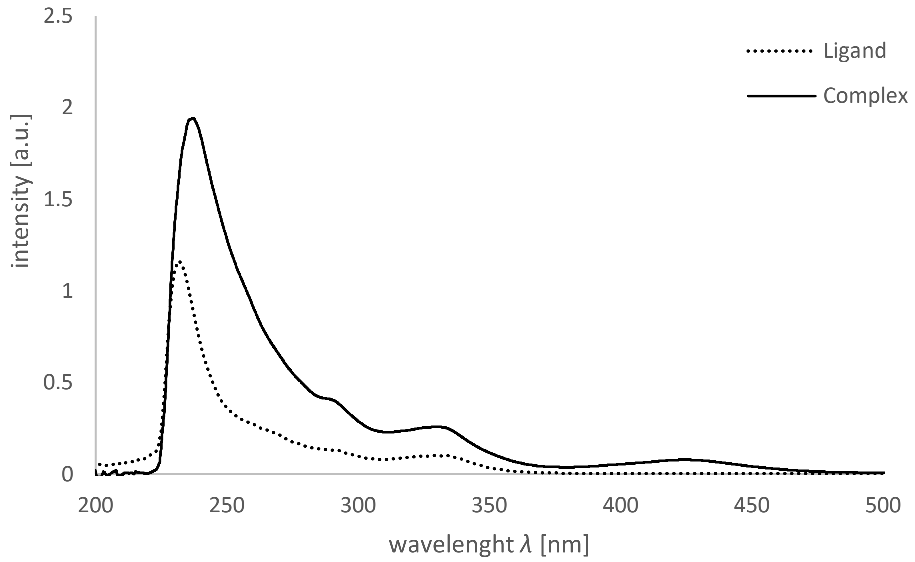

5.16 Synthesis of the Ni(II)-Precatalyst 1-(5-(*tert*-Butyl)-3-((((1*S*,2*S*)-1,2-diphenyl-2-((trifluoromethyl)sulfonamido)ethyl)imino)methyl)-2-oxybenzyl)-3-ethyl-4-(2'-hydroxy-[1,1'-biphenyl]-2-yl)-1*H*-1,2,3-triazol-3-ium-Ni(II) Hexafluorophosphate(V) (CC10*HPF$_6$)

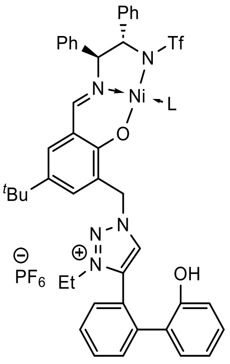

Synthesis of the Ni(II)-triazolium-phenoxyimine-complex **CC10*HPF$_6$** was performed according to **GP3**.[2] Therefore, the triazolium-phenoxyimine-preligand (13.4 mg, 14.4 µmol, 1.0 equiv.) and Ni(acac)$_2$ (3.7 mg, 14.4 µmol, 1.0 equiv.) were reacted in dry acetonitrile

(2 mL). The Ni(II)-triazolium-phenoxyimine-complex **CC10*HPF$_6$** was isolated as a yellow solid (3.7 mg, 3.8 µmol, 26%).

Due to the presence of multiple complex-species in CDCl$_3$, structural analysis by NMR-spectroscopy is impossible.

$C_{43}H_{41}F_9N_5NiO_4PS$, MW: 984.54 g/mol. **Mp.**: >226 °C (Decomposition). **[*α*]$^{20}_{D}$ (*c* = 1.0 mg/mL, DCM)**: –4°. **IR (CDCl$_3$):** $\tilde{\nu}$ = 3226, 3146, 3066, 3034, 2952, 2925, 2854, 2247, 2177, 2042, 1992, 1622, 1603, 1556, 1495, 1455, 1378, 1327, 1274, 1229, 1192, 1143, 1049, 1028, 1009, 952, 912, 815, 755, 699, 596, 515, 432. **HRMS (ESI) m/z:** Calculated for [M-PF$_6$] **$C_{43}H_{41}F_3N_5NiO_4S^+$**: 838.2179; Measured: 838.2184.
UV-Vis (DCM):

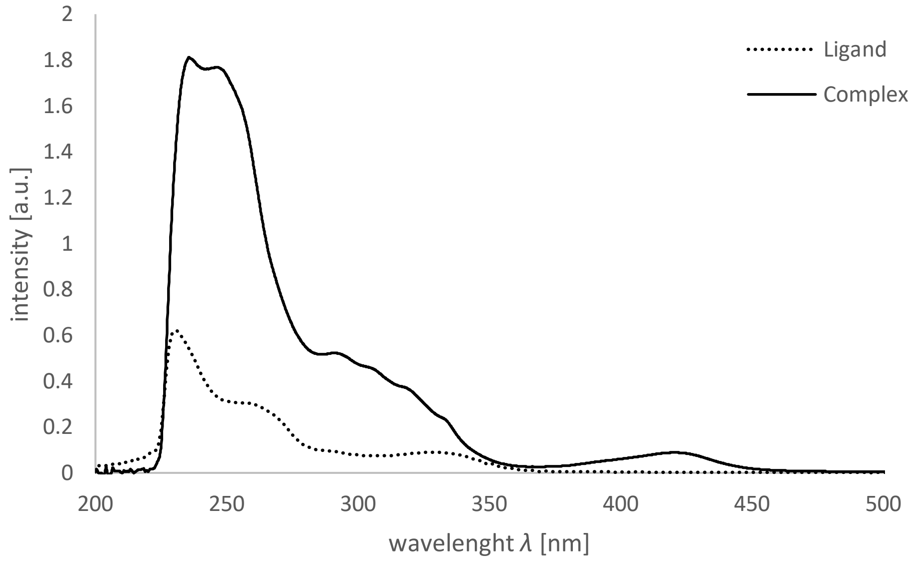

5.17 Synthesis of the Ni(II)-Precatalyst 1-(5-(*tert*-Butyl)-3-((((1*S*,2*S*)-1,2-diphenyl-2-((trifluoromethyl)sulfonamido)ethyl)imino)methyl)-2-oxybenzyl)-3-ethyl-4-(2-hydroxyphenyl)-1*H*-1,2,3-triazol-3-ium-Ni(II) Hexafluorophosphate(V) (CC9*HPF$_6$)

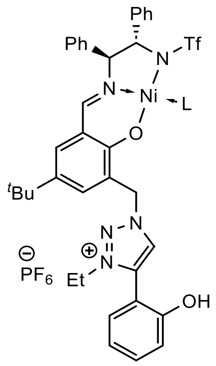

Synthesis of the Ni(II)-triazolium-phenoxyimine-complex **CC9*HPF$_6$** was performed according to **GP3**.[2] Therefore, the triazolium-phenoxyimine-preligand (25.5 mg, 29.9 μmol, 1.0 equiv.) and Ni(acac)$_2$ (7.7 mg, 29.9 μmol, 1.0 equiv.) were reacted in dry acetonitrile (2 mL). The Ni(II)-triazolium-phenoxyimine-complex **CC9*HPF$_6$** was isolated as a yellow solid (3.7 mg, 4.2 μmol, 14%).

Structural analysis by NMR spectroscopy is not possible due to the resulting line broadening caused by the paramagnetic properties of the complex.

$C_{37}H_{37}F_9N_5NiO_4PS$, MW: 908.45 g/mol. **Mp.**: >178 °C (Decomposition). **[*α*]$^{20}_{D}$ (*c* = 1.0 mg/mL, DCM)**: −188°. **IR (CDCl$_3$):** ṽ = 3528, 3491, 3062, 3031, 2962, 2868, 2182, 2159, 2037, 1962, 1621, 1553, 1496, 1451, 1394, 1366, 1321, 1275, 1210, 1182, 1147, 1090, 1008, 961, 910, 841, 760, 731, 699, 647, 613, 593, 558, 515, 429. **HRMS (ESI) m/z:** Calculation for $[M-PF_6]^+$ **$C_{37}H_{37}F_3N_5NiO_4S^+$**: 762.1866; Measured: 762.1868.

UV-Vis (DCM):

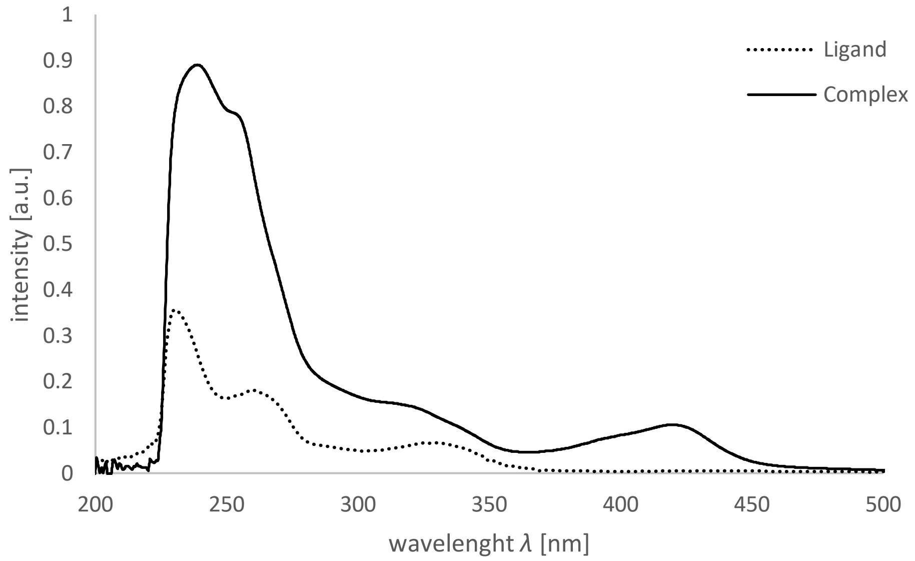

5.18 Synthesis of the Ni(II)-Precatalyst 1-(5-(*tert*-Butyl)-3-((((1*S*,2*S*)-1,2-diphenyl-2-((trifluoromethyl)sulfonamido)ethyl)imino)methyl)-2-oxybenzyl)-3-ethyl-4-phenyl-1*H*-1,2,3-triazol-3-ium-Ni(II) Hexafluorophosphate(V) (CC11)

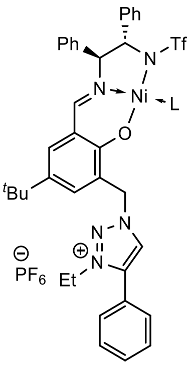

Synthesis of the Ni(II)-triazolium-phenoxyimine-complex **CC11** was performed according to **GP3**.[2] Therefore, the triazolium-phenoxyimine-preligand **L2** (21.8 mg, 26.0 µmol, 1.0 equiv.) and $Ni(acac)_2$ (6.7 mg, 26.0 µmol, 1.0 equiv.) were reacted in dry acetonitrile (2 mL). The Ni(II)-triazolium-phenoxyimin-complex **CC11** was isolated as a yellow-brown solid (11.4 mg, 12.8 µmol, 49%).

Due to the paramagnetic properties of the complex **CC11**, structural analysis by NMR-spectroscopy is not possible by the reason of paramagnetic line broadening.

$C_{37}H_{37}F_9N_5NiO_3PS$, MW: 892.45 g/mol. **Mp.**: >137 °C (Decomposition). **$[\alpha]^{20}_D$ (*c* = 1.0 mg/mL, DCM)**: +90°. **IR ($CDCl_3$):** $\tilde{\nu}$ = 3369, 3062, 3028, 2958, 2925, 2855, 2264, 2205, 2066, 1990, 1624, 1554, 1494, 1452, 1366, 1329, 1212, 1186, 1149, 1092, 1072, 1008, 960, 913, 842, 769, 699, 646, 613, 558, 516, 446, 424. **HRMS (ESI) m/z:** Measured for $[M-PF_6]^+$ **$C_{37}H_{37}F_3N_5NiO_3S^+$**: 746.1917; Measured: 746.1917.
UV-Vis (DCM):

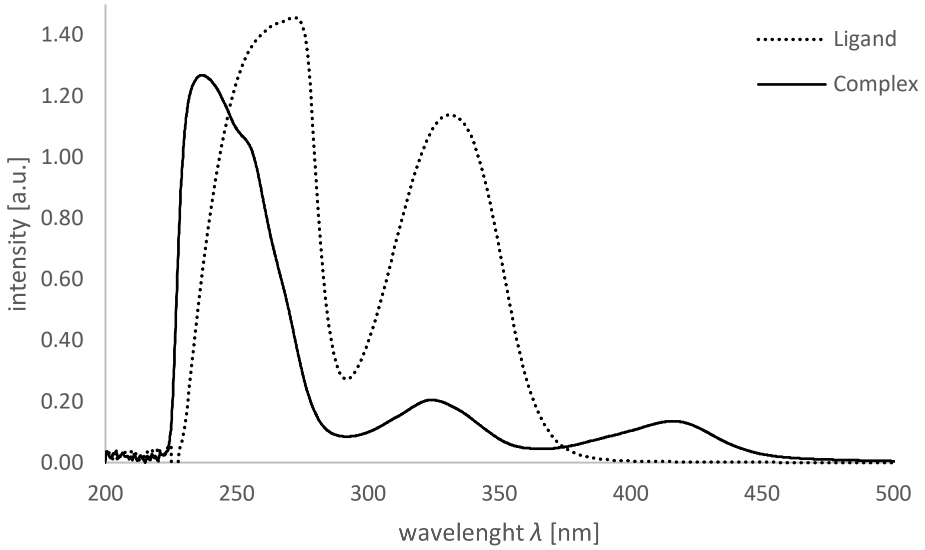

5.19 Synthesis of the Ni(II)-Precatalyst ((1*S*,2*S*)-2-((5-(*tert*-Butyl)-2-oxy-3-methylbenzylidene)amino)-1,2-diphenylethyl)-1,1,1-trifluoromethane-sulfonamide-Ni(II) (CC12)

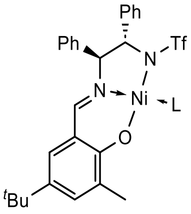

Synthesis of the Ni(II)-triazolium-phenoxyimine-complex **CC12** was performed according to **GPX**.[2] Therefore, the triazolium-phenoxyimine-preligand (11.6 mg, 22.5 µmol, 1.0 equiv.) and Ni(acac)$_2$ (5.8 mg, 22.5 µmol, 1.0 equiv.) were reacted in dry acetonitrile (2 mL). The Ni(II)-triazolium-phenoxyimine-complex **CC12** was isolated as a yellow-green solid (19.7 mg, 34.2 mmol, >99%).

Due to the paramagnetic properties of the complex, structural analysis by NMR-spectroscopy is not possible by the reason of paramagnetic line broadening.

$C_{27}H_{27}F_3N_2NiO_3S$, MW: 575.27 g/mol. **Mp.**: >176 °C (Decomposition). **[*α*]$^{20}_{D}$ (*c* = 1.0 mg/mL, DCM)**: –234°. **IR (CDCl$_3$):** $\tilde{\nu}$ = 3338, 3027, 2962, 2922, 2867, 2263, 2217, 2169, 2058, 1974, 1651, 1618, 1550, 1527, 1451, 1394, 1324, 1269, 1211, 1186, 1071, 958, 770, 699, 514. **HRMS (ESI) m/z:** Calculated for [M] **$C_{27}H_{27}F_3N_2NiO_3S$**: 574.1048; Measured: 575.1047.
UV-Vis (DCM):

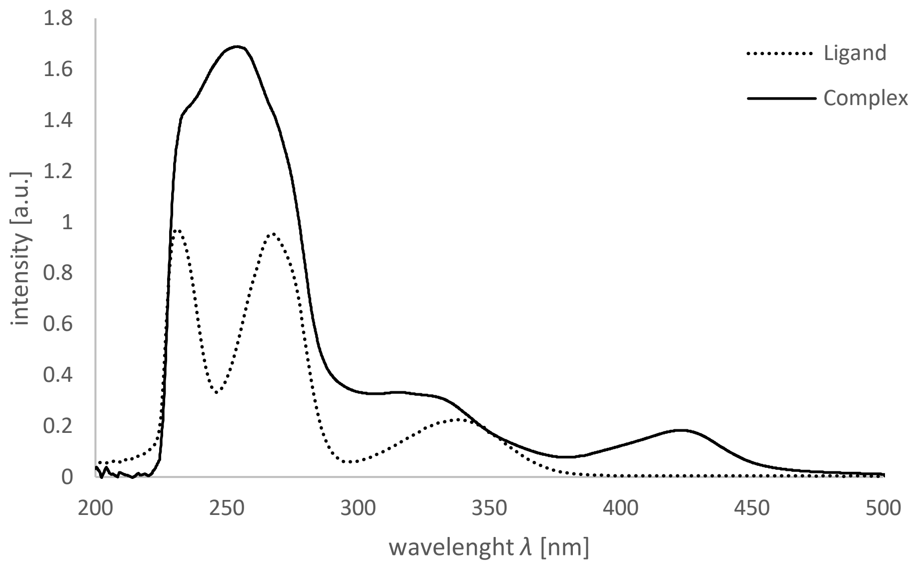

1.8
1.6
1.4
1.2
1
0.8
0.6
0.4
0.2
0
intensity [a.u.]
200
250
300
350
400
450
500
wavelenght λ [nm]
Ligand
Complex

6 Substrate Synthesis

6.1 Synthesis of Methyl-(*E*)-2-(benzylideneamino)acetate (1c)[7,8]

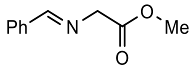

Synthesis of the iminoester **1c** was performed according to **GP5**.[7] Therefore, the hydrochloride (1000 mg, 7.96 mmol, 1.5 equiv.), triethylamine (1184 µL, 8.50 mmol, 1.6 equiv.) and benzaldehyde (542 µL, 5.31 mmol, 1.0 equiv.), were reacted in dry degassed DCM (10 mL). The reaction time for the condensation step was 20 h. After purification, the iminoester **1c** (305 mg, 1.72 mmol, 32%) was isolated as a colourless liquid.

$C_{10}H_{11}NO_2$, MW: 177.20 g/mol. **^{1}H-NMR (400 MHz, $CDCl_3$):** δ = 8.30 (*s*, 1H, C*H*N), 7.81-7.74 (*m*, 2H, Ar-*H*), 7.49-7.38 (*m*, 3H, Ar-*H*), 4.42 (*d*, *J* = 1.11 Hz, 2H, CH_2), 3.78 (*s*, 3H, OCH_3).

The analytic data of **1c** is in agreement with the literature.[8]

6.2 Synthesis of Ethyl-(*E*)-2-(benzylideneamino)acetate (1a)[7]

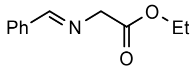

Synthesis of the iminoester **1a** was performed according to **GP5**.[7] Therefore, the hydrochloride (1000 mg, 7.16 mmol, 1.5 equiv.), triethylamine (1065 µL, 7.64 mmol, 1.6 equiv.) and benzaldehyde (487 µL, 4.78 mmol, 1.0 equiv.), were reacted in dry degassed DCM (10 mL). The reaction time for the condensation step was 20 h. After purification, the iminoester **1a** (305 mg, 1.59 mmol, 33%) was isolated as a colourless liquid.

$C_{11}H_{13}NO_2$, MW: 191.23 g/mol. **^{1}H-NMR (400 MHz, $CDCl_3$):** δ = 8.30 (*s*, 1H, C*H*N), 7.81-7.76 (*m*, 2H, Ar-*H*), 7.48-7.38 (*m*, 3H, Ar-*H*), 4.40 (*d*, *J* = 1.15 Hz, 2H, CH_2), 4.42 (*q*, *J* = 7.17 Hz, 2H, OCH_2-CH_3), 1.31 (*t*, *J* = 7.10 Hz, 3H, OCH_2-CH_3).

The analytic data of **1a** is in agreement with the literature.[7]

6.3 Synthesis of Benzyl-(*E*)-2-(benzylideneamino)acetate (1d)

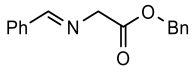

Synthesis of the iminoester **1d** was performed according to **GP5**.[7] Therefore, the hydrochloride (500.0 mg, 2.48 mmol, 1.5 equiv.), triethylamine (367 µL, 2.64 mmol, 1.6 equiv.) and benzaldehyde (268 µL, 1.65 mmol, 1.0 equiv.), were reacted in dry degassed DCM (10 mL). The reaction time for the condensation step was 20 h. After purification, the iminoester **1d** (339 mg, 1.34 mmol, 81 %) was isolated as a colourless solid.

$C_{16}H_{13}NO_3$, MW: 267.28 g/mol. **^{1}H-NMR (500 MHz, $CDCl_3$):** δ = 8.30 (*s*, 1*H*, CN*H*), 7.80-7.76 (*m*, 2H, Ar-*H*), 7.46-7.32 (*m*, 8H, Ar-*H*), 5.32 (*s*, 2H, OC*H*$_2$Ph) 4.31 (*s*, 2H, PhNC*H*$_2$), 4.46 (*d*, *J* = 1.3 Hz, PhNC*H*$_2$). **^{13}C-NMR (176 MHz,$CDCl_3$):** 170.1, 165.7, 135.7, 135.7, 131.4, 128.8, 128.6, 128.5, 128.5, 66.92, 62.09 **IR ($CDCl_3$):** ṽ = 2967, 2877, 1723, 1417, 1215, 956, 714. **HRMS (ESI) m/z:** Calculated for $[M+Na]^+$: $C_{17}H_{21}NO_4Na^+$: 276.0995; Measured: 276.0998.

6.4 Synthesis of Ethyl-(*E*)-2-((4-methoxybenzylidene)amino)acetate (1e)[7]

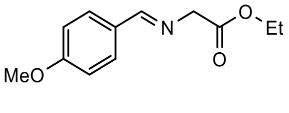

Synthesis of the iminoester **1e** was performed according to **GP5**.[7] Therefore, the hydrochloride (1000 mg, 7.16 mmol, 1.5 equiv.), triethylamine (1065 µL, 7.64 mmol, 1.6 equiv.) and 4-anisaldehyde (581 µL, 4.78 mmol, 1.0 equiv.), were reacted in dry degassed DCM (5 mL). The reaction time for the condensation step was 20 h. After purification, the iminoester **1e** (416 mg, 1.88 mmol, 39%) was isolated as a colourless liquid.

$C_{12}H_{15}NO_3$, MW: 221.26 g/mol. **^{1}H-NMR (400 MHz, $CDCl_3$):** δ = 8.21 (*s*, 1H, C*H*N), 7.76-7.70 (*m*, 2H, Ar-*H*), 6.95-6.90 (*m*, 2H, Ar-*H*), 4.36 (*d*, *J* = 1.1 Hz, 2H, C*H*$_2$), 4.23 (*q*, *J* = 7.1 Hz, 2H, OC*H*$_2$-CH$_3$), 3.84 (*s*, 3H, O-C*H*$_3$), 1.30 (*t*, *J* = 7.1 Hz, 3H, OCH$_2$-C*H*$_3$).

The analytic data of **1e** is in agreement with the literature.[7]

6.5 Synthesis of Ethyl-(*E*)-2-((4-methylbenzylidene)amino)acetate (1f)[7,9]

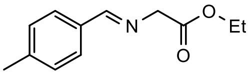

Synthesis of the iminoester **1f** was performed according to **GP5**.[7] Therefore, the hydrochloride (600 mg, 4.30 mmol, 1.5 equiv.), triethylamine (639 µL, 4.59 mmol, 1.6 equiv.) and 4-tolualdehyde (338 µL, 2.87 mmol, 1.0 equiv.), were reacted in dry degassed DCM (5 mL). The reaction time for the condensation step was 20 h. After purification, the iminoester **1f** (220 mg, 1.07 mmol, 37%) was isolated as a colourless liquid.

$C_{12}H_{15}NO_2$, MW: 205.26 g/mol. **^{1}H-NMR (400 MHz, $CDCl_3$):** δ = 8.21 (*s*, 1H, C*H*N), 7.70-7.64 (*m*, 2H, Ar-*H*), 7.25-7.19 (*m*, 2H, Ar-*H*), 4.38 (*d*, *J* = 0.9 Hz, 2H, C*H*$_2$), 4.24 (*q*, *J* = 7.1 Hz, 2H, OC*H*$_2$-CH$_3$), 2.39 (*s*, 3H, Ph-C*H*$_3$), 1.30 (*t*, *J* = 7.2 Hz, 3H, OCH$_2$-C*H*$_3$).

The analytic data of **1f** is in agreement with the literature.[9]

6.6 Synthesis of Ethyl-(*E*)-2-((4-chlorobenzylidene)amino)acetate (1g)[7,10]

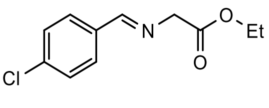

Synthesis of the iminoester **1g** was performed according to **GP5**.[7] Therefore, the hydrochloride (1000 mg, 7.16 mmol, 1.5 equiv.), triethylamine (1065 µL, 7.64 mmol, 1.6 equiv.) and 4-chloroaldehyde (564 µL, 4.78 mmol, 1.0 equiv.), were reacted in dry degassed DCM (5 mL). The reaction time for the condensation step was 20 h. After purification, the iminoester **1g** (455 mg, 2.01 mmol, 42%) was isolated as a colourless liquid.

$C_{11}H_{12}ClNO_2$, MW: 225.67 g/mol. **^{1}H-NMR (400 MHz, $CDCl_3$):** δ = 8.25 (*s*, 1H, C*H*N), 7.74-7.69 (*m*, 2H, Ar-*H*), 7.42-7.36 (*m*, 2H, Ar-*H*), 4.39 (*d*, *J* = 1.2 Hz, 2H, C*H*$_2$), 4.24 (*q*, *J* = 7.1 Hz, 2H, OC*H*$_2$-CH$_3$), 1.30 (*t*, *J* = 7.1 Hz, 3H, OCH$_2$-C*H*$_3$).

The analytic data of **1g** is in agreement with the literature.[10]

6.7 Synthesis of Ethyl-(*E*)-2-((3-chlorobenzylidene)amino)acetate (1h)[7,11]

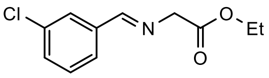

Synthesis of the iminoester **1h** was performed according to **GP5**.[7] Therefore, the hydrochloride (600 mg, 4.30 mmol, 1.5 equiv.), triethylamine (639 µL, 4.59 mmol, 1.6 equiv.) and 3-chloroaldehyde (325 µL, 2.87 mmol, 1.0 equiv.), were reacted in dry degassed DCM (5 mL). The reaction time for the condensation step was 20 h. After purification, the iminoester **1h** (244 mg, 1.08 mmol, 38%) was isolated as a colourless liquid.

$C_{11}H_{12}ClNO_2$, MW: 225.67 g/mol. **^{1}H-NMR (400 MHz, $CDCl_3$):** δ = 8.25 (*s*, 1H, C*H*N), 7.84-7.80 (*m*, 1H, Ar-*H*), 7.65-7.59 (*m*, 1H, Ar-*H*),7.45-7.32 (*m*, 2H, Ar-*H*), 4.40 (*d*, *J* = 1.1 Hz, 2H, C*H*$_2$), 4.25 (*q*, *J* = 7.2 Hz, 2H, OC*H*$_2$-CH$_3$), 1.30 (*t*, *J* = 7.2 Hz, 3H, OCH$_2$-C*H*$_3$).

The analytic data of **1h** is in agreement with the literature.[11]

6.8 Synthesis of Ethyl-(*E*)-2-((2-chlorobenzylidene)amino)acetate (1i)[7,12]

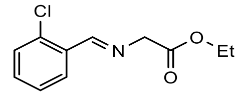

Synthesis of the iminoester **1i** was performed according to **GP5**.[7] Therefore, the hydrochloride (600 mg, 4.30 mmol, 1.5 equiv.), triethylamine (639 µL, 4.59 mmol, 1.6 equiv.) and 2-chloroaldehyde (323 µL, 2.87 mmol, 1.0 equiv.), were reacted in dry degassed DCM (5 mL). The reaction time for the condensation step was 20 h. After purification, the iminoester **1i** (293 mg, 1.43 mmol, 50%) was isolated as a colourless liquid.

$C_{11}H_{12}ClNO_2$, MW: 225.67 g/mol. **^{1}H-NMR (400 MHz, $CDCl_3$):** δ = 8.74 (*s*, 1H, C*H*N), 8.14-8.08 (*m*, 1H, Ar-*H*), 7.43-7.27 (*m*, 3H, Ar-*H*), 4.45 (*d*, *J* = 1.2 Hz, 2H, C*H*$_2$), 4.25 (*q*, *J* = 7.1 Hz, 2H, OC*H*$_2$-CH$_3$), 1.31 (*t*, *J* = 7.2 Hz, 3H, OCH$_2$-C*H*$_3$).

The analytic data of **1i** is in agreement with the literature.[12]

6.9 Synthesis of Ethyl-(*E*)-2-((4-nitrobenzylidene)amino)acetate (1j)[7,10]

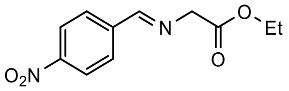

Synthesis of the iminoester **1j** was performed according to **GP5**.[7] Therefore, the hydrochloride (350 mg, 2.51 mmol, 1.5 equiv.), triethylamine (373 µL, 2.67 mmol, 1.6 equiv.) and 4-nitrobenzaldehyde (163 µL, 1.67 mmol, 1.0 equiv.), were reacted in dry degassed DCM (5 mL). The reaction time for the condensation step was 3 h. After purification, the iminoester **1j** (119 mg, 0.50 mmol, 30%) was isolated as a yellow liquid.

$C_{11}H_{12}N_2O_4$, MW: 236.23 g/mol. **^{1}H-NMR (400 MHz, $CDCl_3$):** δ = 8.39 (*s*, 1H, C*H*N), 8.31-8.26 (*m*, 2H, Ar-*H*), 7.99-7.94 (*m*, 2H, Ar-*H*), 4.47 (*d*, *J* = 1.2 Hz, 2H, C*H*$_2$), 4.26 (*q*, *J* = 7.1 Hz, 2H, OC*H*$_2$-CH$_3$), 1.32 (*t*, *J* = 7.1 Hz, 3H, OCH$_2$-C*H*$_3$).

The analytic data of **1j** is in agreement with the literature[7,10].

6.10 Synthesis of *tert*-Butyl-(*E*)-2-(benzylideneamino)acetate (1b)[7,13]

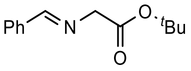

Synthesis of the iminoester **1b** was performed according to **GP5**.[7] Therefore, the hydrochloride (1000 mg, 5.97 mmol, 1.5 equiv.), triethylamine (887 µL, 6.36 mmol, 1.6 equiv.) and benzaldehyde (406 µL, 3.98 mmol, 1.0 equiv.), were reacted in dry degassed DCM (6 mL). The reaction time for the condensation step was 20 h. After purification, the iminoester **1b** (363 mg, 1.66 mmol, 42%) was isolated as a colourless liquid.

$C_{13}H_{17}NO_2$, MW: 219.28 g/mol. **^{1}H-NMR (300 MHz, $CDCl_3$):** δ = 8.27 (*s*, 1H, C*H*N), 7.83-7.73 (*m*, 2H, Ar-*H*), 7.48-7.36 (*m*, 3H, Ar-*H*), 4.32 (*d*, *J* = 1.2 Hz, 2H, C*H*$_2$), 1.50 (*s*, 9H, C(C*H*$_3$)$_3$).

The analytic data of **1b** is in agreement with the literature.[13]

6.11 Synthesis of *tert*-Butyl-(*E*)-2-((4-methoxybenzylidene)amino)acetate (1k)[7,14]

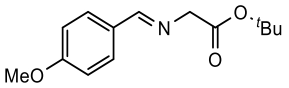

Synthesis of the iminoester **1k** was performed according to **GP5**.[7] Therefore, the hydrochloride (500 mg, 2.98 mmol, 1.5 equiv.), triethylamine (443 µL, 3.18 mmol, 1.6 equiv.) and anisaldehyde (242 µL, 1.99 mmol, 1.0 equiv.), were reacted in dry degassed DCM (5 mL). The reaction time for the condensation step was 20 h. After purification, the iminoester **1k** (347 mg, 1.39 mmol, 70%) was isolated as a yellowish liquid.

$C_{14}H_{19}NO_3$, **MW:** 249.31 g/mol. **^{1}H-NMR (300 MHz, $CDCl_3$):** δ = 8.18 (*s*, 1H, C*H*N), 7.76-7.67 (*m*, 2H, Ar-*H*), 6.96-6.87 (*m*, 2H, Ar-*H*), 4.27 (*d*, *J* = 0.9 Hz, 2H, CH_2), 3.84 (*s*, 3H, OCH_3), 1.49 (*s*, 9H, C-(CH_3)$_3$).

The analytic data of **1k** is in agreement with the literature.[14]

6.12 Synthesis of *tert*-Butyl-(*E*)-2-((4-chlorobenzylidene)amino)acetate (1l)[7,15]

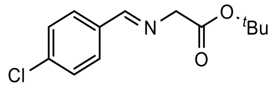

Synthesis of the iminoester **1l** was performed according to **GP5**.[7] Therefore, the hydrochloride (1000 mg, 5.97 mmol, 1.5 equiv.), triethylamine (887 µL, 6.36 mmol, 1.6 equiv.) and 4-chloroaldehyde (470 µL, 3.98 mmol, 1.0 equiv.), were reacted in dry degassed DCM (5 mL). The reaction time for the condensation step was 20 h. After purification, the iminoester **1l** (195mg, 0.77 mmol, 19%) was isolated as a colourless liquid.

$C_{13}H_{16}ClNO_2$, **MW:** 253.73 g/mol. **^{1}H-NMR (300 MHz, $CDCl_3$):** δ = 8.23 (*s*, 1H, C*H*N), 7.76-7.67 (*m*, 2H, Ar-*H*), 7.43-7.34 (*m*, 2H, Ar-*H*), 4.31 (*d*, *J* = 1.1 Hz, 2H, CH_2), 1.49 (*s*, 9H, C-(CH_3)$_3$).

The analytic data of **1l** is in agreement with the literature.[15]

6.13 Synthesis of *tert*-Butyl-(*E*)-2-((4-methylbenzyliden)amino)acetat (1m)[7,16]

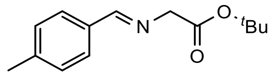

Synthesis of the iminoester **1m** was performed according to **GP5**.[7] Therefore, the hydrochloride (1000 mg, 5.97 mmol, 1.5 equiv.), triethylamine (887 µL, 6.36 mmol, 1.6 equiv.) and 4-tolualdehyde (469 µL, 3.98 mmol, 1.0 equiv.), were reacted in dry degassed DCM (5 mL). The reaction time for the condensation step was 20 h. After purification, the iminoester **1m** (455mg, 1.95 mmol, 49%) was isolated as a colourless liquid.

$C_{14}H_{19}NO_2$, **MW:** 233.31 g/mol. **^{1}H-NMR (300 MHz, $CDCl_3$):** δ = 8.22 (*s*, 1H, C*H*N), 7.67 (*d*, *J* = 8.1 Hz, 2H, Ar-*H*), 7.21 (*d*, *J* = 7.9 Hz, 2H, Ar-*H*), 4.29 (*d*, *J* = 0.9 Hz, 2H, CH_2), 2.38 (*s*, 3H, Ph-CH_3), 1.49 (*s*, 9H, C-(CH_3)$_3$).

The analytic data of **1m** is in agreement with the literature.[7,16]

6.14 Synthesis of *tert*-Butyl-(*E*)-2-((4-nitrobenzylidene)amino)acetate (1n)[7,17]

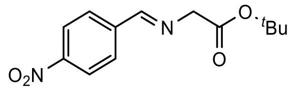

Synthesis of the iminoester **1n** was performed according to **GP5**.[7] Therefore, the hydrochloride (1000 mg, 5.97 mmol, 1.5 equiv.), triethylamine (887 µL, 6.36 mmol, 1.6 equiv.) and 4-nitrobenzaldehyde (389 µL, 3.98 mmol, 1.0 equiv.), were reacted in dry degassed DCM (5 mL). The reaction time for the condensation step was 3 h. After purification, the iminoester **1n** (260 mg, 0.98 mmol, 25%) was isolated as a yellow liquid.

$C_{13}H_{16}N_2O_4$, MW: 264.28 g/mol. **^{1}H-NMR (400 MHz, $CDCl_3$):** δ = 8.36 (*s*, 1H, C*H*N), 8.30-8.24 (*m*, 2H, Ar-*H*), 7.99-7.92 (*m*, 2H, Ar-*H*), 4.38 (*d*, *J* = 1.2 Hz, 2H, CH_2), 1.51 (*s*, 9H, C-$(CH_3)_3$).

The analytic data of **1n** is in agreement with the literature.[17]

7 Synthesis of asymmetric cycloaddition products

7.1 Synthesis of Methyl-(1*S*,3*R*,3a*S*,6a*R*)-5-methyl-4,6-dioxo-3-phenyloctahydropyrrolo[3,4-*c*]pyrrole-1-carboxylate (3cA)[2,18–20]

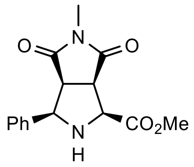

For the synthesis of **3cA** according to **GP7**,[2,18] the iminoester **1c** (42.5 mg, 0.24 mmol, 1.2 equiv.) and maleimide (22.22 mg, 0.20 mmol, 1.0 equiv.) are reacted under utilization of the catalyst **C4** (0.09 mg, 0.1 µmol, 0.05 mol%) in THF (1000 µl) at a temperature of 40 °C for 20 h. After workup of the reaction, using ^{1}H-NMR spectroscopy with mesitylene (25 µL) as an internal standard, the yield (96%) and diastereomeric ratio of *endo*:*exo* (93:7) was determined. After further purification using column chromatography with PE:ethylacetate (2:1) the product was obtained as a diastereomeric mixture (48.1 mg, 0.167 mmol, 83%). For further analysis, starting from a smaller portion of the product, the diastereomers were separated using preparative thin layer chromatography with PE:ethylacetate (2:1) and methyl-(1*S*,3*R*,3a*S*,6a*R*)-5-methyl-4,6-dioxo-3-phenyloctahydropyrrolo[3,4-*c*]pyrrole-1-carboxylate **3cA** was isolated diastereomerically pure as a colourless solid. The *ee*-value (+94%) was determined by HPLC on chiral stationary phase: Chiracel IH, cyclohexane/iPrOH (50:50), 1.0 mL/min, 220 nm, t_R = 5.883 min (minor), t_R = 6.973 min (major).

$C_{15}H_{16}N_2O_4$, MW: 288,30 g/mol. **$[\alpha]^{20}_D$ (c = 1.0 mg/mL, DCM, Sample with 94% *ee*):** +50. **^{1}H-NMR (400 MHz, $CDCl_3$):** δ = 7.39-7.29 (*m*, 5H, Ar-*H*), δ = 4.51 (*d*, *J* = 8.6 Hz, 1H, NH-C*H*-Ph), δ = 4.06 (*d*, *J* = 6.8 Hz, 1H, NH-C*H*-CO_2Et), δ =3.89 (*s*, 3H, CO_2CH_3), δ = 3.57 (*t*, *J* = 7.2 Hz, 1H, C*H*- $CHCO_2$Me), δ = 3.44 (*t*, *J* = 8.0 Hz, 1H, C*H*-CH-Ph), δ = 2.87 (*s*, 3H, N-C*H*$_3$), δ = 2.46 (*br*, 1H, N*H*).

The analytic data of **3cA** is in agreement with the literature.[19,20]

7.2 Synthesis of ethyl-(1*S*,3*R*,3a*S*,6a*R*)-5-methyl-4,6-dioxo-3-phenyloctahydropyrrolo[3,4-*c*]pyrrol-1-carboxylate (3aA)[2,5,21]

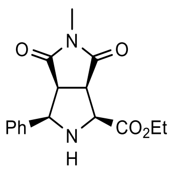

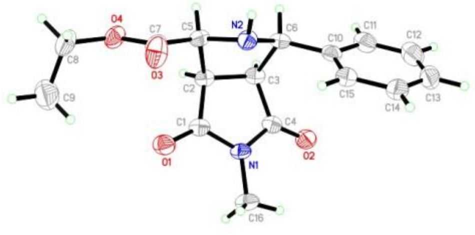

For the synthesis of **3aA** according to **GP7**,[2,18] the iminoester **1a** (45.9 mg, 0.24 mmol, 1.2 equiv.) and *N*-methylmaleimide (22.22 mg, 0.20 mmol, 1.0 equiv.) are reacted under utilization of the catalyst **C4** (0.09 mg, 0.1 µmol, 0.05 mol%) in THF (1000 µl) at a temperature of 40 °C for 20 h. After workup of the reaction, using ^{1}H-NMR spectroscopy with mesitylene (25 µL) as an internal standard, the yield (93%) and diastereomeric ratio of *endo*:*exo* (93:7) was determined. After further purification using column chromatography with PE:ethylacetate (2:1) the product was obtained as a diastereomeric mixture (50.6 mg, 0.167 mmol, 84%). For further analysis, starting from a smaller portion of the product, the diastereomers were separated using preparative thin layer chromatography with PE:ethylacetate (2:1) and ethyl-(1*S*,3*R*,3a*S*,6a*R*)-5-methyl-4,6-dioxo-3-phenyloctahydropyrrolo[3,4-*c*]pyrrol-1-carboxylate **3aA** was isolated diastereomerically pure as a colourless solid. The *ee*-value (+97%) was determined by HPLC on chiral stationary phase: Chiracel IH, cyclohexane/iPrOH (50:50), 1.0 mL/min, 220 nm, t_R = 7.307 min (minor), t_R = 11.967 min (major).

$C_{16}H_{18}N_2O_4$, MW: 302.33 g/mol. **$[\alpha]^{20}_D$ (*c* = 1.0 mg/mL, DCM, Sample with 97% *ee*):** +12. **^{1}H-NMR (300 MHz, $CDCl_3$):** δ = 7.40-7.29 (*m*, 5H, Ar-*H*), δ = 4.51 (*d*, *J* = 8.6 Hz, 1H NH-C*H*-Ph), δ = 4.44-4.27 (*m*, 2H, $CO_2CH_2CH_3$), δ = 4.05 (*d*, *J* = 6.8 Hz, 1H, NH-C*H*-CO_2Et), δ = 3.59 (*t*, *J* = 7.1 Hz, 1H, C*H*- CHCO_2Et), δ = 3.44 (*t*, *J* = 8.0 Hz, 1H, C*H*-CH-Ph), δ = 2.87 (*s*, 3H, N-CH_3), δ = 1.39 (*t*, *J* = 7.2 Hz, 3H, $CO_2CH_2CH_3$).

The analytic data of **3aA** is in agreement with the literature.[21]

7.3 Synthesis of benzyl-(1*S*,3*R*,3a*S*,6a*R*)-5-methyl-4,6-dioxo-3-phenyloctahydropyrrolo[3,4-c]pyrrole-1-carboxylate (3dA)

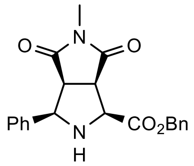

For the synthesis of **3dA** according to **GP7**,[2,18] the iminoester **1d** (60.8 mg, 0.24 mmol, 1.2 equiv.) and *N*-methylmaleimide (22.22 mg, 0.20 mmol, 1.0 equiv.) are reacted under utilization of the catalyst **C4** (0.09 mg, 0.1 µmol, 0.05 mol%) in THF (1000 µl) at a temperature of 40 °C for 20 h. After workup of the reaction, using ^{1}H-NMR spectroscopy with mesitylene (25 µL) as an internal standard, the yield (95%) and diastereomeric ratio of *endo*:*exo* (95:5) was determined. After further purification using column chromatography with PE:ethylacetate (2:1) the product was obtained as a diastereomeric mixture (59.4 mg, 0.163 mmol, 82%). For further analysis, starting from a smaller portion of the product, the diastereomers were separated using preparative thin layer chromatography with PE:ethylacetate (2:1) and benzyl-(1*S*,3*R*,3a*S*,6a*R*)-5-methyl-4,6-dioxo-3-phenyloctahydropyrrolo[3,4-c]pyrrole-1-carboxylate **3dA** was isolated diastereomerically pure as a colourless solid. The *ee*-value (+95%) was

determined by HPLC on chiral stationary phase: Chiracel IA, cyclohexane/iPrOH (52:48), 0.7 mL/min, 220 nm, t_R = 9.763 min (major), t_R = 11.000 min (minor).

$C_{21}H_{20}N_2O_4$, MW: 364.40 g/mol. **Mp.**: 177-184 °C. **$[\alpha]^{20}_D$ (*c* = 1.0 mg/mL, DCM, Sample with 95% *ee*):** −14. **^{1}H-NMR (700 MHz, $CDCl_3$):** δ = 7.49-7.45 (*m*, 2H, Ar-*H*), δ = 7.42-7.30 (*m*, 8H, Ar-*H*), δ = 5.34 (*d*, *J* = 12.1 Hz, 1H, CO_2-C*H*$_2$-Ph), δ = 5.28 (*d*, *J* = 12.0 Hz, 1H, CO_2-C*H*$_2$-Ph), δ = 4.50 (*dd*, *J* = 4.7-, 8.7 Hz, 1H NH-C*H*-Ph), δ = 4.11-4.06 (*m*, 1H, NH-C*H*-CO_2Et), δ = 3.59 (*t*, *J* = 7.2 Hz, 1H, C*H*-CHCO_2Et), δ = 3.43 (*t*, *J* = 8.3 Hz, 1H, C*H*-CH-Ph), δ = 2.89 (*s*, 3H, N-C*H*$_3$), δ = 2.43 (*br*, 1H, N*H*). **^{13}C-NMR (176 MHz, $CDCl_3$):** δ = 176.3, 175.0, 170.0, 136.9, 135.7, 129.1, 128.9, 128.8, 128.8, 128.7, 127.3, 67.8, 64.4, 62.1, 49.8, 48.5, 25.4. **IR ($CDCl_3$):** ṽ = 3326, 3027, 2997, 2929, 2848, 2252, 2002, 1756, 1698, 1497, 1439, 1385, 1350, 1313, 1285, 1245, 1189, 1119, 1097, 1072, 1028, 1008, 978, 941, 915, 899, 857, 838, 786, 755, 729, 700, 649, 631, 588, 553, 518, 487, 453. **HRMS (ESI) m/z:** Calculated for $[M-H]^+$ **$C_{21}H_{21}N_2O_4^+$**: 365.1496; Measured: 365.1492.

7.4 Synthesis of ethyl-(1*S*,3*R*,3*aS*,6*aR*)-3-(4-methoxyphenyl)-5-methyl-4,6-dioxooctahydropyrrolo[3,4-c]pyrrole-1-carboxylate (3eA)[2,22]

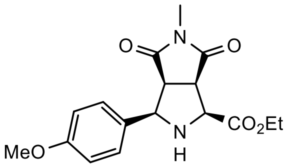

For the synthesis of **3eA** according to **GP6**,[2] the iminoester **1e** (54.2 mg, 0.24 mmol, 1.2 equiv.) and *N*-methylmaleimide (22.22 mg, 0.20 mmol, 1.0 equiv.) are reacted under utilization of the catalyst **C4** (0.18 mg, 0.2 µmol, 0.1 mol%) in THF (1000 µl) at a temperature of 40 °C for 20 h. After workup of the reaction, using ^{1}H-NMR spectroscopy with mesitylene (25 µL) as an internal standard, the yield (81%) and diastereomeric ratio of *endo*:*exo* (94:6) was determined. After further purification using column chromatography with PE:ethylacetate (1.5:1) the product was obtained diastereomerically pure (55.4 mg, 0.167 mmol, 83%, sum of the yields of both diastereomers). Ethyl-(1*S*,3*R*,3*aS*,6*aR*)-3-(4-methoxyphenyl)-5-methyl-4,6-dioxooctahydropyrrolo[3,4-c]pyrrole-1-carboxylate **3eA** was isolated diastereomerically pure as a colourless solid. The *ee*-value (+94%) was determined by HPLC on chiral stationary phase: Chiracel IH, cyclohexane/iPrOH (50:50), 1.0 mL/min, 220 nm, t_R = 11.823 min (major), t_R = 15.737 min (minor).

$C_{17}H_{20}N_2O_5$, MW: 332.36 g/mol. **$[\alpha]^{20}_D$ (*c* = 1.0 mg/mL, DCM, Sample with 94% *ee*):** +78. **^{1}H-NMR (400 MHz, $CDCl_3$):** δ = 7.28-7.22 (*m*, 2H, Ar-*H*), δ = 6.91-7.84 (*m*, 2H, Ar-*H*), δ = 4.45 (*d*, *J* = 8.6 Hz, 1H NH-C*H*-Ph), δ = 4.40-4.29 (*m*, 2H, CO_2C*H*$_2$$CH_3$), δ = 4.01 (*d*, *J* = 6.8 Hz, 1H, NH-C*H*-CO_2Et), δ = 3.80 (*s*, 3H, OC*H*$_3$), δ = 3.56 (*t*, *J* = 7.2 Hz, 1H, C*H*- CHCO_2Et), δ = 3.38 (*t*,

J = 8.1 Hz, 1H, C*H*-CH-Ph), δ = 2.88 (*s*, 3H, N-CH_3), δ = 2.42 (*br*, 1H, N*H*), δ = 1.38 (*t*, J = 7.2 Hz, 3H, $CO_2CH_2CH_3$).

The analytic data of **3eA** is in agreement with the literature.[22]

7.5 Synthesis of ethyl-(1*S*,3*R*,3*aS*,6*aR*)-5-methyl-4,6-dioxo-3-(*p*-tolyl)octahydropyrrolo[3,4-c]pyrrole-1-carboxylate (3fA)

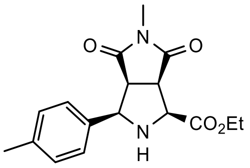

For the synthesis of **3fA** according to **GP6**,[2] the iminoester **1f** (49.3 mg, 0.24 mmol, 1.2 equiv.) and *N*-methylmaleimide (22.22 mg, 0.20 mmol, 1.0 equiv.) are reacted under utilization of the catalyst **C4** (0.18 mg, 0.2 µmol, 0.1 mol%) in THF (1000 µl) at a temperature of 40 °C for 20 h. After workup of the reaction, using ^{1}H-NMR spectroscopy with mesitylene (25 µL) as an internal standard, the yield (96%) and diastereomeric ratio of *endo*:*exo* (93:7) was determined. After further purification using column chromatography with PE:ethylacetate (1.5:1) the product was obtained as a diastereomeric mixture (62.9 mg, 0.199 mmol, 99%). For further analysis, starting from a smaller portion of the product, the diastereomers were separated using preparative thin layer chromatography with PE:ethylacetate (2:1) and ethyl-(1*S*,3*R*,3*aS*,6*aR*)-5-methyl-4,6-dioxo-3-(*p*-tolyl)octahydropyrrolo[3,4-c]pyrrole-1-carboxylate **3fA** was isolated diastereomerically pure as a colourless solid. The *ee*-value (+96%) was determined by HPLC on chiral stationary phase: Chiracel IH, cyclohexane/iPrOH (50:50), 1.0 mL/min, 220 nm, t_R = 7.903 min (major), t_R = 13.460 min (minor).

$C_{17}H_{20}N_2O_4$, MW: 316.36 g/mol. **Mp.**: 176-181 °C. **$[\alpha]^{20}_D$ (*c* = 1.0 mg/mL, DCM, Sample with 96% *ee*):** +32. **^{1}H-NMR (700 MHz, CDCl$_3$):** δ = 7.22 (*d*, J = 8.1 Hz, 2H, Ar-*H*), δ = 7.15 (*d*, J = 7.9 Hz, 2H, Ar-*H*), δ = 4.46 (*d*, J = 8.7 Hz, 1H NH-C*H*-Ph), δ = 4.40-4.30 (*m*, 2H, $CO_2CH_2CH_3$), δ = 4.02 (*d*, J = 6.9 Hz, 1H, NH-C*H*-CO_2Et), δ = 3.56 (*t*, J = 7.1 Hz, 1H, C*H*-CH CO_2Et), δ = 3.41 (*t*, J = 8.1 Hz, 1H, C*H*-CH-Ph), δ = 2.88 (*s*, 3H, N-CH_3), δ = 2.34 (*s*, 3H, Ph-CH_3), δ = 1.38 (*t*, J = 7.2 Hz, 3H, $CO_2CH_2CH_3$). **^{13}C-NMR (176 MHz, CDCl$_3$):** δ = 176.2, 175.2, 170.0, 138.3, 133.9, 129.5, 127.22, 64.3, 62.2, 61.8, 50.0, 48.7, 25.3, 21.6, 14.5. **IR (CDCl$_3$):** $\tilde{\nu}$ = 3319, 2987, 2960, 2931, 2858, 2253, 2169, 1767, 1749, 1695, 1513, 1438, 1381, 1351, 1286, 1246, 1191, 1121, 1093, 1036, 994, 973, 944, 915, 897, 850, 809, 727, 684, 649, 590, 561, 515, 476. **HRMS (ESI) m/z:** Calculated for $[M-H]^+$ **$C_{17}H_{21}N_2O_4^+$**: 317.1496; Measured: 317.1487.

7.6 Synthesis of ethyl-(1*S*,3*R*,3a*S*,6a*R*)-3-(4-chlorophenyl)-5-methyl-4,6-dioxooctahydropyrrolo[3,4-*c*]pyrrole-1-carboxylate (3gA)[2,5,23]

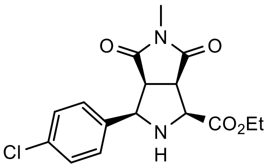

For the synthesis of **3gA** according to **GP7**,[2,5] the iminoester **1g** (54.2 mg, 0.24 mmol, 1.2 equiv.) and *N*-methylmaleimide (22.22 mg, 0.20 mmol, 1.0 equiv.) are reacted under utilization of the catalyst **C4** (45.49 µg, 0.05 µmol, 0.025 mol%) in THF (1000 µl) at a temperature of 40 °C for 20 h. After workup of the reaction, using ^{1}H-NMR spectroscopy with mesitylene (25 µL) as an internal standard, the yield (99%) and diastereomeric ratio of *endo*:*exo* (92:8) was determined. After further purification using column chromatography with PE:ethylacetate (1.5:1) the product was obtained as a diastereomeric mixture (66.1 mg, 0.196 mmol, 98%). For further analysis, starting from a smaller portion of the product, the diastereomers were separated using preparative thin layer chromatography with PE:ethylacetate (1.5:1) and ethyl-(1*S*,3*R*,3a*S*,6a*R*)-3-(4-chlorophenyl)-5-methyl-4,6-dioxooctahydropyrrolo[3,4-*c*]pyrrole-1-carboxylate **3gA** was isolated diastereomerically pure as a colourless solid. The *ee*-value (+93%) was determined by HPLC on chiral stationary phase: Chiracel IH, cyclohexane/iPrOH (50:50), 1.0 mL/min, 220 nm, t_R = 8.020 min (major), t_R = 13.007 min (minor).

$C_{16}H_{17}ClN_2O_4$, MW: 336.09 g/mol. **$[\alpha]^{20}_D$ (*c* = 1.0 mg/mL, DCM, Sample with 93% *ee*):** +92. **^{1}H-NMR (400 MHz, $CDCl_3$):** δ = 7.35-7.27 (*m*, 2H, Ar-*H*), δ = 4.45 (*d*, *J* = 8.6 Hz, 1H NH-C*H*-Ph), δ = 4.42-4.28 (*m*, 2H, $CO_2CH_2CH_3$), δ = 4.02 (*d*, *J* = 6.8 Hz, 1H, NH-C*H*-CO_2Et), δ = 3.56 (*t*, *J* = 7.2 Hz, 1H, C*H*- $CHCO_2$Et), δ = 3.41 (*t*, *J* = 8.0 Hz, 1H, C*H*-CH-Ph), δ = 2.87 (*s*, 3H, N-CH_3), δ = 1.39 (*t*, *J* = 7.2 Hz, 3H, $CO_2CH_2CH_3$).

The analytic data of **3gA** is in agreement with the literature.[23]

7.7 Synthesis of ethyl-(1*S*,3*R*,3a*S*,6a*R*)-3-(3-chlorophenyl)-5-methyl-4,6-dioxooctahydropyrrolo[3,4- c]pyrrole-1-carboxylate (3hA)

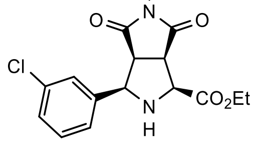

For the synthesis of **3hA** according to **GP7**,[2,5] the iminoester **1h** (54.2 mg, 0.24 mmol, 1.2 equiv.) and *N*-methylmaleimide (22.22 mg, 0.20 mmol, 1.0 equiv.) are reacted under utilization of the catalyst **C4** (0.09 mg, 0.1 µmol, 0.05 mol%) in THF (1000 µl) at a temperature

of 40 °C for 20 h. After workup of the reaction, using ^{1}H-NMR spectroscopy with mesitylene (25 µL) as an internal standard, the yield (99%) and diastereomeric ratio of *endo*:*exo* (92:8) was determined. After further purification using column chromatography with PE:ethylacetate (1.5:1) the product was obtained diastereomerically pure (68.9 mg, 0.205 mmol, >99%, sum of the yields of both diastereomers). Ethyl-(1*S*,3*R*,3*aS*,6*aR*)-3-(3-chlorophenyl)-5-methyl-4,6-dioxooctahydropyrrolo[3,4- c]pyrrole-1-carboxylate **3hA** was isolated diastereomerically pure as a colourless solid. The *ee*-value (+90%) was determined by HPLC on chiral stationary phase: Chiracel IH, cyclohexane/iPrOH (50:50), 1.0 mL/min, 220 nm, t_R = 7.930 min (major), t_R = 13.063 min (minor).

$C_{16}H_{17}ClN_2O_4$, MW: 336.77 g/mol. **Mp.**: 200-211 °C. **$[\alpha]^{20}_D$ (*c* = 1.0 mg/mL, DCM, Sample with 90% *ee*):** +38. **^{1}H-NMR (700 MHz, $CDCl_3$):** δ = 7.36 (*s*, 1H, Ar-*H*), δ = 7.29-7.27 (*m*, 2H, Ar-*H*), δ = 7.25-7.22 (*m*, 1H, Ar-*H*), δ = 4.46 (*d*, *J* = 8.8 Hz, 1H NH-C*H*-Ph), δ = 4.40-4.30 (*m*, 2H, $CO_2CH_2CH_3$), δ = 4.03 (*d*, *J* = 6.8 Hz, 1H, NH-C*H*-CO_2Et), δ = 3.57 (*t*, *J* = 7.1 Hz, 1H, C*H*-CHCO_2Et), δ = 3.43 (*t*, *J* = 8.1 Hz, 1H, C*H*-CH-Ph), δ = 2.88 (*s*, 3H, N-CH_3), δ = 2.39 (*br*, 1H, N*H*), δ = 1.39 (*t*, *J* = 7.1 Hz, 3H, $CO_2CH_2CH_3$). **^{13}C-NMR (176 MHz, $CDCl_3$):** δ = 176.0, 174.8, 169.8, 139.2, 134.8, 130.0, 128.8, 127.5, 125.68, 63.6, 62.0, 61.92, 49.6, 48.2, 25.4, 14.5. **IR ($CDCl_3$):** $\tilde{\nu}$ = 3344, 2992, 2958, 2926, 2852, 2251, 2158, 2016, 1773, 1702, 1597, 1574, 1476, 1434, 1382, 1347, 1311, 1285, 1244, 1195, 1099, 1010, 974, 861, 792, 687, 654, 597, 445. **HRMS (ESI) m/z:** Calculated for $[M-H]^+$ **$C_{16}H_{18}ClN_2O_4^+$**: 337.0950; Measured: 337.0946.

7.8 Synthesis of Ethyl-3-(2-chlorophenyl)-5-methyl-4,6-dioxooctahydropyrrolo[3,4-*c*]pyrrole-1-carboxylate (3iA)

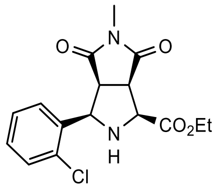

For the synthesis of **3iA** according to **GP6**,[2] the iminoester **1i** (54.2 mg, 0.24 mmol, 1.2 equiv.) and *N*-methylmaleimide (22.22 mg, 0.20 mmol, 1.0 equiv.) are reacted under utilization of the catalyst **C4** (0.36 mg, 0.4 µmol, 0.2 mol%) in THF (1000 µl) at a temperature of 40 °C for 20 h. After workup of the reaction, using ^{1}H-NMR spectroscopy with mesitylene (25 µL) as an internal standard, the yield (99%) and diastereomeric ratio of *endo*:*exo* (97:3) was determined. After further purification using column chromatography with PE:ethylacetate (1.5:1) the product was obtained as a diastereomeric mixture (68.9 mg, 0.205 mmol, >99%). The diastereomers could not be separated using preparative thin layer chromatography with PE:ethylacetate (1.5:1). The diastereomeric mixture of ethyl-3-(2-chlorophenyl)-5-methyl-4,6-dioxooctahydropyrrolo[3,4-*c*]pyrrole-1-carboxylate was obtained as a colourless solid. The *ee*-value (+93%) was determined by HPLC on chiral stationary phase: Chiracel IH,

cyclohexane/iPrOH (50:50), 1.0 mL/min, 220 nm, t_R = 6.540 min (major), t_R = 10.747 min (minor).

$C_{16}H_{17}ClN_2O_4$, MW: 336.77 g/mol. **Mp.**: 95-105 °C. **$[\alpha]^{20}_D$ (c = 1.0 mg/mL, DCM, Sample with 93% *ee*):** +80. **^{1}H-NMR (700 MHz, $CDCl_3$):** δ = 7.53-7.50 (*m*, 1H, Ar-*H*), δ = 7.42-7.38 (*m*, 1H, Ar-*H*), δ = 7.26-7.22 (*m*, 2H, Ar-*H*), δ = 4.74 (*d*, *J* = 8.3 Hz, 1H NH-C*H*-Ph), δ = 4.40-4.29 (*m*, 2H, $CO_2CH_2CH_3$), δ = 4.05 (*d*, *J* = 7.0 Hz, 1H, NH-C*H*-CO_2Et), δ = 3.72 (*t*, *J* = 8.1 Hz, 1H, C*H*-CH-Ph), δ = 3.57 (*t*, *J* = 7.4 Hz, 1H, NH-C*H*-CO_2Et), δ = 2.81 (*s*, 3H, N-CH_3), δ = 2.32 (*br*, 1H, N*H*), δ = 1.38 (*t*, *J* = 7.1 Hz, 3H, $CO_2CH_2CH_3$). **^{13}C-NMR (176 MHz, $CDCl_3$):** δ = 176.2, 174.7, 169.9, 135.3, 133.6, 129.5, 129.4, 127.5, 127.3, 61.8, 61.7, 60.7, 47.9, 47.0, 25.3, 14.5. **IR ($CDCl_3$):** ṽ = 3468, 3333, 2983, 2940, 2854, 2253, 1777, 1742, 1696, 1573, 1473, 1435, 1380, 1347, 1311, 1285, 1241, 1193, 1117, 1096, 1052, 1034, 997, 941, 912, 855, 757, 727, 684, 648, 593, 534, 458. **HRMS (ESI) m/z:** Calculated for $[M-H]^+$ **$C_{16}H_{17}ClN_2O_4^+$**: 337.0950; Measured: 337.0947.

7.9 Synthesis of ethyl-(1*S*,3*R*,3a*S*,6a*R*)-5-methyl-3-(4-nitrophenyl)-4,6-dioxooctahydropyrrolo[3,4-c]pyrrole-1-carboxylate (3jA)

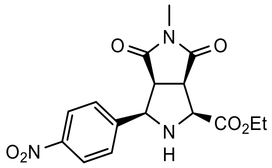

For the synthesis of **3jA** according to **GP6**,[2] the iminoester **1j** (56.7 mg, 0.24 mmol, 1.2 equiv.) and *N*-methylmaleimide (22.22 mg, 0.20 mmol, 1.0 equiv.) are reacted under utilization of the catalyst **C4** (0.18 mg, 0.2 µmol, 0.1 mol%) in THF (1000 µl) at a temperature of 40 °C for 20 h. After workup of the reaction, using ^{1}H-NMR spectroscopy with mesitylene (25 µL) as an internal standard, the yield (99%) and diastereomeric ratio of *endo*:*exo* (88:12) was determined. After further purification using column chromatography with PE:ethylacetate (1:1) the product was obtained diastereomerically pure (67.1 mg, 0.193 mmol, 97%, sum of the yields of both diastereomers). Ethyl-(1*S*,3*R*,3a*S*,6a*R*)-5-methyl-3-(4-nitrophenyl)-4,6-dioxooctahydropyrrolo[3,4-c]pyrrole-1-carboxylate **3jA** was isolated diastereomerically pure as a yellowish solid. The *ee*-value (+75%) was determined by HPLC on chiral stationary phase: Chiracel IH, cyclohexane/iPrOH (50:50), 1.0 mL/min, 220 nm, t_R = 12.547 min (major), t_R = 16.870 min (minor).

$C_{16}H_{17}N_3O_6$, MW: 347.33 g/mol. **Mp.**: 163-170 °C. **$[\alpha]^{20}_D$ (c = 1.0 mg/mL, DCM, Sample with 75% *ee*):** +92. **^{1}H-NMR (400 MHz, $CDCl_3$):** δ = 8.20 (*d*, *J* = 8.3 Hz, 2H, Ar-*H*), δ = 7.56 (*d*, *J* = 8.3 Hz, 2H, Ar-*H*), δ = 4.58 (*d*, *J* = 7.4 Hz, 1H NH-C*H*-Ph), δ = 4.42-4.24 (*m*, 2H, $CO_2CH_2CH_3$), δ = 4.07 (*d*, *J* = 5.3 Hz, 1H, NH-C*H*-CO_2Et), δ = 3.61 (*t*, *J* = 6.7 Hz, 1H, C*H*-

$CHCO_2Et$), δ = 3.50 (*t*, *J* = 7.5 Hz, 1H, C*H*-CH-Ph), δ = 2.86 (*s*, 3H, N-C*H*$_3$), δ = 2.43 (*br*, 1H, N*H*), δ = 1.39 (*t*, *J* = 7.0 Hz, 3H, $CO_2CH_2CH_3$). **^{13}C-NMR (176 MHz, $CDCl_3$):** δ = 175.7, 174.6, 169.6, 148.1, 144.7, 128.3, 124.0, 63.2, 62.0, 49.6, 47.9, 25.4, 14.5. **IR ($CDCl_3$):** ṽ = 3330, 2984, 2926, 2855, 2256, 1778, 1741, 1698, 1602, 1518, 1435, 1382, 1347, 1313, 1287, 1207, 1093, 1036, 999, 914, 861, 827, 750, 731, 695, 648, 589, 463. **HRMS (ESI) m/z:** Calculated for $[M\text{-}H]^+$ **$C_{16}H_{18}N_3O_6^+$**: 348.1190; Measured: 348.1186.

7.10 Synthesis of ethyl-(1*S*,3*R*,3*aS*,6*aR*)-4,6-dioxo-3-phenyloctahydropyrrolo[3,4-c]pyrrole-1-carboxylate (3aB)

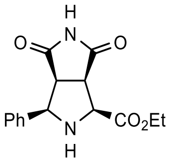

For the synthesis of **3aB** according to **GP6**,[2] the iminoester **1a** (45.9 mg, 0.24 mmol, 1.2 equiv.) and *N*-methylmaleimide (22.22 mg, 0.20 mmol, 1.0 equiv.) are reacted under utilization of the catalyst **C4** (0.55 mg, 0.6 µmol, 0.3 mol%) in THF (1000 µl) at a temperature of 40 °C for 20 h. After workup of the reaction, using ^{1}H-NMR spectroscopy with mesitylene (25 µL) as an internal standard, the yield (99%) and diastereomeric ratio of *endo*:*exo* (94:6) was determined. After further purification using column chromatography with PE:ethylacetate:acetic acid (70:29:1) the product was obtained as a diastereomeric mixture (56.3 mg, 0.195 mmol, 98%). For further analysis, starting from a smaller portion of the product, the diastereomers were separated using preparative thin layer chromatography with PE:ethylacetate:acetic acid (70:29:1) and ethyl-(1*S*,3*R*,3*aS*,6*aR*)-4,6-dioxo-3-phenyloctahydropyrrolo[3,4-c]pyrrole-1-carboxylate **3aB** was isolated diastereomerically pure as a colourless solid. The *ee*-value (+94%) was determined by HPLC on chiral stationary phase: Chiracel IH, cyclohexane/iPrOH (50:50), 1.0 mL/min, 220 nm, t_R = 7.443min (major), t_R = 9.917 min (minor).

$C_{15}H_{16}N_2O_4$, MW: 288.30 g/mol. **Mp.**: 142-153 °C. **$[\alpha]^{20}_D$ (*c* = 1.0 mg/mL, DCM, Sample with 94% *ee*):** +24. **^{1}H-NMR (700 MHz, $CDCl_3$):** δ = 7.84 (*br*, 1H, CO_2-NH-CO_2), δ = 7.41 (*d*, *J* = 7.3 Hz, 2H, Ar-*H*), δ = 7.36 (*t*, *J* = 7.6 Hz, 2H, Ar-*H*), δ = 7.32 (*t*, *J* = 7.2 Hz, 2H, Ar-*H*), δ = 4.50 (*d*, *J* = 8.4 Hz, 1H NH-C*H*-Ph), δ = 4.38-4.30 (*m*, 2H, $CO_2CH_2CH_3$), δ = 4.03 (*d*, *J* = 6.1 Hz, 1H, NH-C*H*-CO_2Et), δ = 3.62 (*t*, *J* = 6.7 Hz, 1H, C*H*- $CHCO_2Et$), δ = 3.44 (*t*, *J* = 7.8 Hz, 1H, C*H*-CH-Ph), δ = 2.50 (*br*, 1H, Ph-CH-N*H*), δ = 1.37 (*t*, *J* = 7.2 Hz, 3H, $CO_2CH_2CH_3$). **^{13}C-NMR (176 MHz, $CDCl_3$):** δ = 175.8, 174.6, 169.8, 136.7, 128.8, 128.8, 127.4, 64.5, 62.2, 61.9, 51.1, 49.7, 14.5. **IR ($CDCl_3$):** ṽ = 3237, 3083, 2957, 2925, 2853, 2254, 1781, 1713, 1456, 1379, 1348, 1311, 1199, 1124, 1099, 1070, 1009, 910, 857, 810, 763, 730,

699, 634, 570. **HRMS (ESI) m/z:** Calculated for $[M-H]^+$ $C_{15}H_{17}N_2O_4^+$: 289.1183; Measured: 289.1174.

7.11 Synthesis of ethyl-(1*S*,3*R*,3a*S*,6a*R*)-4,6-dioxo-3,5-diphenyloctahydropyrrolo[3,4-*c*]pyrrole-1-carboxylate (3aC)[2,5,24]

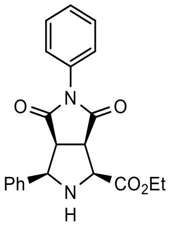

For the synthesis of **3aC** according to **GP7**,[2,5] the iminoester **1a** (45.9 mg, 0.24 mmol, 1.2 equiv.) and *N*-phenylmaleimide (34.6 mg, 0.20 mmol, 1.0 euqiv.) are reacted under utilization of the catalyst **C4** (0.046 mg, 0.05 µmol, 0.025 mol%) in THF (1000 µl) at a temperature of 40 °C for 20 h. After workup of the reaction, using ^{1}H-NMR spectroscopy with mesitylene (25 µL) as an internal standard, the yield (99%) and diastereomeric ratio of *endo*:*exo* (93:7) was determined. After further purification using column chromatography with PE:ethylacetate (1.5:1) the product was obtained as a diastereomeric mixture (62.9 mg, 0.173 mmol, 86%). For further analysis, starting from a smaller portion of the product, the diastereomers were separated using preparative thin layer chromatography with PE:ethylacetate (1.5:1) and ethyl-(1*S*,3*R*,3a*S*,6a*R*)-4,6-dioxo-3,5-diphenyloctahydropyrrolo[3,4-*c*]pyrrole-1-carboxylate **3aC** was isolated diastereomerically pure as a colourless solid. The *ee*-value (+86%) was determined by HPLC on chiral stationary phase: Chiracel IH, cyclohexane/iPrOH (50:50), 1.0 mL/min, 220 nm, t_R = 8.357 min (major), t_R = 16.833 min (minor).

$C_{21}H_{20}N_2O_4$, MW: 364.40 g/mol. **$[\alpha]^{20}_D$ (*c* = 1.0 mg/mL, DCM, Sample with 86% *ee*):** +50. **^{1}H-NMR (300 MHz, $CDCl_3$):** δ = 7.49-7.27 (*m*, 8H, Ar-*H*), δ = 7.18-7.10 (*m*, 2H, Ar-*H*), δ = 4.62 (*d*, *J* = 8.8 Hz, 1H NH-C*H*-Ph), δ = 4.42-4.26 (*m*, 2H, $CO_2CH_2CH_3$), δ = 4.13 (*d*, *J* = 6.4 Hz, 1H, NH-C*H*-CO_2Et), δ = 3.74 (*t*, *J* = 6.8 Hz, 1H, C*H*- $CHCO_2$Et), δ = 3.57 (*t*, *J* = 8.0 Hz, 1H, C*H*-CH-Ph), δ = 1.25 (*t*, *J* = 7.2 Hz, 3H, $CO_2CH_2CH_3$).

The analytic data of **3aC** is in agreement with the literature.[24]

7.12 Synthesis of ethyl-(1*S*,3*R*,3a*S*,6a*R*)-5-benzyl-4,6-dioxo-3-phenyloctahydropyrrolo[3,4-*c*]pyrrole-1-carboxylate (3aD)

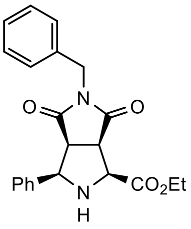

For the synthesis of **3aD** according to **GP6**,[2] the iminoester **1a** (45.9 mg, 0.24 mmol, 1.2 equiv.) and *N*-benzylmaleimide (37.4 mg, 0.20 mmol, 1.0 equiv.) are reacted under utilization of the catalyst **C4** (0.18 mg, 0.2 µmol, 0.1 mol%) in THF (1000 µl) at a temperature of 40 °C for 20 h. After workup of the reaction, using ^{1}H-NMR spectroscopy with mesitylene (25 µL) as an internal standard, the yield (99%) and diastereomeric ratio of *endo*:*exo* (92:8) was determined. After further purification using column chromatography with PE:ethylacetate (1:1) the product was obtained diastereomerically pure (73.4 mg, 0.194 mmol, 97%, sum of the yields of both diastereomers). ethyl-(1*S*,3*R*,3a*S*,6a*R*)-5-benzyl-4,6-dioxo-3-phenyloctahydropyrrolo[3,4-*c*]pyrrole-1-carboxylate **3aD** was isolated diastereomerically pure as a colourless solid. The *ee*-value (+95%) was determined by HPLC on chiral stationary phase: Chiracel IH, cyclohexane/iPrOH (50:50), 1.0 mL/min, 220 nm, t_R = 7.097 min (major), t_R = 17.990 min (minor).

$C_{22}H_{22}N_2O_4$, MW: 378.43 g/mol. **$[\alpha]^{20}_D$ (*c* = 1.0 mg/mL, DCM, Sample with 95% *ee*):** +20. **^{1}H-NMR (400 MHz, $CDCl_3$):** δ = 7.35-7.17 (*m*, 8H, Ar-*H*), δ = 7.16-7.10 (*m*, 2H, Ar-*H*), δ = 4.61-4.41 (*m*, 3H, NH-C*H*-Ph, CH_2-Ph), δ = 4.36 (*q*, *J* = 7.1 Hz, 2H, $CO_2CH_2CH_3$), δ = 4.02 (*d*, *J* = 7.0 Hz, 1H, NH-C*H*-CO_2Et), δ = 3.56 (*t*, *J* = 7.2 Hz, 1H, C*H*- CHCO_2Et), δ = 3.37 (*t*, *J* = 8.1 Hz, 1H, C*H*-CH-Ph), δ = 2.26 (*br*, 1H, N*H*), δ = 1.38 (*t*, *J* = 7.2 Hz, 3H, $CO_2CH_2CH_3$). **^{13}C-NMR (176 MHz, $CDCl_3$):** δ = 175.6, 174.4, 169.7, 136.4, 135.8, 129.2, 128.7, 128.5, 128.3, 128.0, 127.2, 64.4, 62.2, 61.6, 49.6, 48.5, 42.7, 14.3. **IR ($CDCl_3$):** ṽ = 3335, 3032, 2983, 2934, 2851, 1743, 1703, 1455, 1430, 1397, 1347, 1313, 1196, 1130, 1033, 952, 856, 753, 700, 635, 589. **HRMS (ESI) m/z:** Calculated for $[M-H]^+$ **$C_{22}H_{23}N_2O_4^+$**: 379.1652; Measured: 379.1648.

7.13 Synthesis of *tert*-butyl-(1*R*,3*S*,3a*S*,6a*R*)-5-methyl-4,6-dioxo-3-phenyloctahydropyrrolo[3,4-*c*]pyrrole-1-carboxylate (3bA)

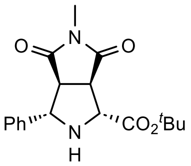

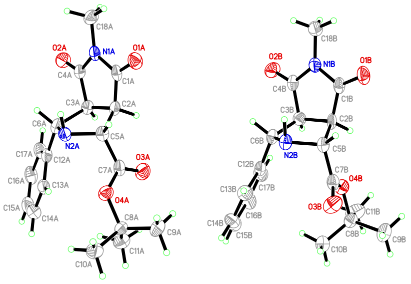

For the synthesis of **3bA** according to **GP6**,[2] the iminoester **1b** (52.6 mg, 0.24 mmol, 1.2 equiv.) and *N*-methylmaleimide (22.2 mg, 0.20 mmol, 1.0 equiv.) are reacted under utilization of the catalyst **C7** (0.94 mg, 1.0 µmol, 0.5 mol%) in THF (100 µl) and methanol (4 µl, 0.10 mmol, 0.5 equiv.) as an additive at a temperature of 10 °C for 20 h. After workup of the reaction, using ^{1}H-NMR spectroscopy with mesitylene (25 µL) as an internal standard, the yield (96%) and diastereomeric ratio of *exo*:*endo* (89:11) was determined. After further purification using column chromatography with PE:ethylacetate (1.5:1) the product was obtained as a diastereomeric mixture (62.7 mg, 0.190 mmol, 95%). For further analysis, starting from a smaller portion of the product, the diastereomers were separated using preparative thin layer chromatography with PE:ethylacetate (1.5:1) and *tert*-butyl-(1*R*,3*S*,3a*S*,6a*R*)-5-methyl-4,6-dioxo-3-phenyloctahydropyrrolo[3,4-*c*]pyrrole-1-carboxylate **3bA** was isolated diastereomerically pure as a colourless solid. The *ee*-value (94%) was determined by HPLC on chiral stationary phase: Chiracel IA, cyclohexane/iPrOH (70:30), 0.7 mL/min, 220 nm, t_R = 16.940 min (minor), t_R = 28.263 min (major).

$C_{18}H_{22}N_2O_4$, MW: 330.38 g/mol. **Mp.**: 91-93 °C. **$[\alpha]^{20}_D$ (*c* = 1.0 mg/mL, DCM, Sample with 94% *ee*):** +4. **^{1}H-NMR (700 MHz, $CDCl_3$):** δ = 7.41 (*d*, *J* = 7.5 Hz, 2H, Ar-*H*), 7.35 (*t*, *J* = 7.7 Hz, 2H, Ar-*H*), 7.29 (*t*, *J* = 7.3 Hz, 1H, Ar-*H*), δ = 4.49 (*d*, *J* = 5.2 Hz, 1H, NH-C*H*-Ph), δ = 3.94 (*d*, *J* = 4.5 Hz, 1H, NH-C*H*-CO_2Et), δ = 3.81 (*dd*, J = 4.6, 8.9 Hz, 1H, C*H*-CH CO_2Et), δ = 3.46 (*dd*, *J* = 5.2, 8.8 Hz, 1H, C*H*-CH-Ph), δ = 3.04 (*s*, 3H, N-CH_3), δ = 2.60 (*br*, 1H, N*H*), δ = 1.39 (*s*, 9H, C(CH_3)$_3$). **^{13}C-NMR (176 MHz, $CDCl_3$):** δ = 177.6, 177.4, 170.8, 141.1, 129.1, 128.3, 127.0, 82.9, 65.5, 63.6, 52.4, 49.5, 28.2, 25.5. **IR ($CDCl_3$):** ṽ = 3336, 2979, 2932, 1778, 1698, 1604, 1434, 1382, 1369, 1281, 1250, 1156, 1130, 959, 879, 844, 753, 701, 670, 638, 572, 509. **HRMS (ESI) m/z:** Calculated for $[M-H]^+$ **$C_{18}H_{23}N_2O_4^+$**: 331.1652; Measured: 331.1642.

7.14 Synthesis of *tert*-butyl-(1*R*,3*S*,3*aS*,6*aR*)-3-(4-methoxyphenyl)-5-methyl-4,6-dioxooctahydropyrrolo[3,4-*c*]pyrrole-1-carboxylate (3kA)

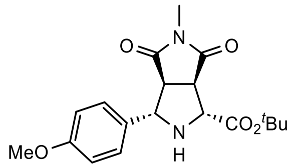

For the synthesis of **3kA** according to **GP6**,[2] the iminoester **1k** (59.8 mg, 0.24 mmol, 1.2 equiv.) and *N*-methylmaleimide (22.2 mg, 0.20 mmol, 1.0 equiv.) are reacted under utilization of the catalyst **C7** (3.75 mg, 4.0 µmol, 2.0 mol%) in THF (100 µl) and methanol (4 µl, 0.10 mmol, 0.5 equiv.) as an additive at a temperature of 10 °C for 20 h. After workup of the reaction, using ^{1}H-NMR spectroscopy with mesitylene (25 µL) as an internal standard, the yield (99%) and diastereomeric ratio of *exo*:*endo* (80:20) was determined. After further purification using column chromatography with PE:ethylacetate (70:30) the product was obtained as a diastereomeric mixture (67.1 mg, 0.186 mmol, 93%). For further analysis, starting from a smaller portion of the product, the diastereomers were separated using preparative thin layer chromatography with PE:ethylacetate (1.5:1) and *tert*-butyl-(1*R*,3*S*,3*aS*,6*aR*)-3-(4-methoxyphenyl)-5-methyl-4,6-dioxooctahydropyrrolo[3,4-*c*]pyrrole-1-carboxylate **3kA** was isolated diastereomerically pure as a colourless solid. The *ee*-value (90%) was determined by HPLC on chiral stationary phase: Chiracel IA, cyclohexane/iPrOH (50:50), 1.0 mL/min, 220 nm, t_R = 11.907 min (minor), t_R = 15.797 min (minor).

$C_{19}H_{24}N_2O_5$, MW: 360.41 g/mol. **Mp.**: 57-70 °C. **$[\alpha]^{20}_D$ (*c* = 1.0 mg/mL, DCM, Sample with 90% *ee*):** +8. **^{1}H-NMR (700 MHz, $CDCl_3$):** δ = 7.33 (*d*, *J* = 8.7 Hz, 2H, Ar-*H*), δ = 6.88 (*d*, *J* = 8.7 Hz, 2H, Ar-*H*), δ = 4.41 (*d*, *J* = 5.4 Hz, 1H, NH-C*H*-Ph), δ = 3.92 (*d*, *J* = 4.7 Hz, 1H, NH-C*H*-CO_2Et), δ = 3.81-3.77 (*m*, 4H, C*H*-CH CO_2Et/OC*H*$_3$), δ = 3.41 (*dd*, *J* = 5.5, 8.9 Hz, 1H, C*H*-CH-Ph), δ = 3.03 (*s*, 3H, N-C*H*$_3$), δ = 2.64 (*br*, 1H, N*H*), δ = 1.41 (*s*, 9H, C(C*H*$_3$)$_3$). **^{13}C-NMR (176 MHz, $CDCl_3$):** δ = 177.5, 177.4, 170.9, 159.7, 133.0, 128.2, 114.5, 82.9, 65.2, 63.5, 55.7, 52.6, 49.6, 28.2, 25.5. **IR ($CDCl_3$):** $\tilde{\nu}$ = 3338, 2978, 2933, 2840, 1983, 1778, 1703, 1612, 1585, 1514, 1434, 1382, 1369, 1285, 1250, 1157, 1034, 959, 833, 627, 530. **HRMS (ESI) m/z:** Calculated for $[M-H]^+$ **$C_{19}H_{25}N_2O_5{}^+$**: 361.1758; Measured: 361.1753.

7.15 Synthesis of *tert*-butyl-(1*R*,3*S*,3*aS*,6*aR*)-3-(4-chlorophenyl)-5-methyl-4,6-dioxooctahydropyrrolo[3,4-*c*]pyrrole-1-carboxylate (3lA)

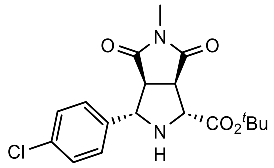

For the synthesis of **3lA** according to **GP6**,[2] the iminoester **1l** (60.9 mg, 0.24 mmol, 1.2 equiv.) and *N*-methylmaleimide (22.2 mg, 0.20 mmol, 1.0 equiv.) are reacted under utilization of the catalyst **C7** (0.94 mg, 1.0 µmol, 0.5 mol%) in THF (100 µl) and methanol (4 µl, 0.10 mmol, 0.5 equiv.) as an additive at a temperature of 10 °C for 20 h. After workup of the reaction, using ^{1}H-NMR spectroscopy with mesitylene (25 µL) as an internal standard, the yield (96%) and diastereomeric ratio of *exo*:*endo* (92:8) was determined. After further purification using column chromatography with PE:ethylacetate (70:30) the product was obtained as a diastereomeric mixture (70.2 mg, 0.192 mmol, 96%). For further analysis, starting from a smaller portion of the product, the diastereomers were separated using preparative thin layer chromatography with PE:ethylacetate (1.5:1) and *tert*-butyl-(1*R*,3*S*,3a*S*,6a*R*)-3-(4-chlorophenyl)-5-methyl-4,6-dioxooctahydropyrrolo[3,4-*c*]pyrrole-1-carboxylate **3lA** was isolated diastereomerically pure as a colourless solid. The *ee*-value (91%) was determined by HPLC on chiral stationary phase: Chiracel IA, cyclohexane/iPrOH (50:50), 1.0 mL/min, 220 nm, t_R = 11.990 min (major), t_R = 18.733 min (minor).

$C_{18}H_{21}ClN_2O_4$, MW: 364.83 g/mol. **Mp.**: 100-106 °C. **$[\alpha]^{20}_D$ (*c* = 1.0 mg/mL, DCM, Sample with 91% *ee*):** +12. **^{1}H-NMR (700 MHz, $CDCl_3$):** δ = 7.39 (*d*, *J* = 8.5 Hz, 2H, Ar-*H*), δ = 7.32 (*d*, *J* = 8.5 Hz, 2H, Ar-*H*), δ = 4.42 (*d*, *J* = 5.5 Hz, 1H, NH-C*H*-Ph), δ = 3.95 (*d*, *J* = 4.8 Hz, 1H, NH-C*H*-CO_2Et), δ = 3.79 (*dd*, J = 4.8, 8.9 Hz, 1H, C*H*-CH CO_2Et), δ = 3.37 (*dd*, *J* = 5.6, 9.0 Hz, 1H, C*H*-CH-Ph), δ = 3.03 (*s*, 3H, N-CH_3), δ = 2.63 (*br*, 1H, N*H*), δ = 1.43 (*s*, 9H, C(CH_3)$_3$). **^{13}C-NMR (176 MHz, $CDCl_3$):** δ = 177.3, 177.1, 170.6, 139.6, 134.1,129.2, 128.4, 83.0, 64.9, 63.5, 52.7, 49.5, 28.2, 25.6. **IR ($CDCl_3$):** ṽ = 3338, 2979, 2933, 1778, 1699, 1492, 1434, 1382, 1369, 1281, 1250, 1156, 1130, 1091, 1015, 959, 831, 755, 734, 650, 617, 517, 449. **HRMS (ESI) m/z:** Calculated for $[M-H]^+$ **$C_{18}H_{22}ClN_2O_4^+$**: 365.1263; Measured: 365.1260.

7.16 Synthesis of *tert*-butyl-(1*R*,3*S*,3a*S*,6a*R*)-5-methyl-4,6-dioxo-3-(*p*-tolyl)octahydropyrrolo[3,4-*c*]pyrrole-1-carboxylate (3mA)

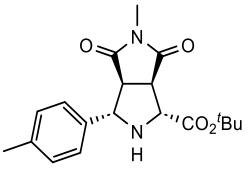

For the synthesis of **3mA** according to **GP6**,[2] the iminoester **1m** (56.0 mg, 0.24 mmol, 1.2 equiv.) and *N*-methylmaleimide (22.2 mg, 0.20 mmol, 1.0 equiv.) are reacted under utilization of the catalyst **C7** (0.94 mg, 1.0 µmol, 0.5 mol%) in THF (100 µl) and methanol (4 µl, 0.10 mmol, 0.5 equiv.) as an additive at a temperature of 10 °C for 20 h. After workup of the reaction, using ^{1}H-NMR spectroscopy with mesitylene (25 µL) as an internal standard, the yield (91%) and diastereomeric ratio of *exo*:*endo* (82:18) was determined. After further purification using column chromatography with PE:ethylacetate (70:30) the product was obtained as a

diastereomeric mixture (60.5 mg, 0.183 mmol, 92%). For further analysis, starting from a smaller portion of the product, the diastereomers were separated using preparative thin layer chromatography with PE:ethylacetate (1.5:1) and *tert*-butyl-(1*R*,3*S*,3*aS*,6*aR*)-5-methyl-4,6-dioxo-3-(*p*-tolyl)octahydropyrrolo[3,4-*c*]pyrrole-1-carboxylate **3mA** was isolated diastereomerically pure as a colourless solid. The *ee*-value (89%) was determined by HPLC on chiral stationary phase: Chiracel IA, cyclohexane/iPrOH (50:50), 0.7 mL/min, 220 nm, t_R = 12.833 min (minor), t_R = 18.333 min (major).

$C_{19}H_{24}N_2O_4$, MW: 344.41 g/mol. **Mp.**: 64-71 °C. **$[\alpha]^{20}_D$ (*c* = 1.0 mg/mL, DCM, Sample with 89% *ee*):** –6. **^{1}H-NMR (700 MHz, $CDCl_3$):** δ = 7.28 (*d*, *J* = 8.0 Hz, 2H, Ar-*H*), δ = 7.15 (*d*, *J* = 7.7 Hz, 2H, Ar-*H*), δ = 4.44 (*d*, *J* = 5.1 Hz, 1H, NH-C*H*-Ph), δ = 3.93 (*d*, *J* = 4.5 Hz, 1H, NH-C*H*-CO_2Et), δ = 3.80 (*dd*, J = 4.5, 8.9 Hz, 1H, C*H*-CH-CO_2Et), δ = 3.43 (*dd*, *J* = 5.2, 8.8 Hz, 1H, C*H*-CH-Ph), δ = 3.03 (*s*, 3H, N-CH_3), δ = 2.33 (*s*, 3H, Ph-CH_3), δ = 1.40 (*s*, 9H, C(CH_3)$_3$). **^{13}C-NMR (176 MHz, $CDCl_3$):** δ = 177.5, 177.3, 170.8, 138.0, 137.9, 129.8, 126.9, 82.9, 64.4, 63.6, 52.5, 49.6, 28.2, 25.5, 21.4. **IR ($CDCl_3$):** ṽ = 3338, 2978, 2929, 1778, 1702, 1515, 1434, 1382, 1369, 1281, 1250, 1157, 1130, 1022, 959, 844, 813, 740, 628, 515. **HRMS (ESI) m/z:** Calculated for $[M-H]^+$ **$C_{19}H_{25}N_2O_4^+$**: 345.1809; Measured: 345.1805.

7.17 Synthesis of *tert*-butyl-(1*R*,3*S*,3*aS*,6*aR*)-5-methyl-3-(4-nitrophenyl)-4,6-dioxooctahydropyrrolo[3,4-*c*]pyrrole-1-carboxylate (3nA)

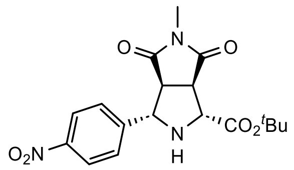

For the synthesis of **3nA** according to **GP6**,[2] the iminoester **1n** (63.4 mg, 0.24 mmol, 1.2 equiv.) and *N*-methylmaleimide (22.2 mg, 0.20 mmol, 1.0 equiv.) are reacted under utilization of the catalyst **C7** (1.88 mg, 2.0 µmol, 1.0 mol%) in THF (100 µl) and methanol (4 µl, 0.10 mmol, 0.5 equiv.) as an additive at a temperature of 10 °C for 20 h. After workup of the reaction, using ^{1}H-NMR spectroscopy with mesitylene (25 µL) as an internal standard, the yield (77%) and diastereomeric ratio of *exo*:*endo* (69:39) was determined. After further purification using column chromatography with PE:ethylacetate (1.5:1) the product was obtained as a diastereomeric mixture (55.9 mg, 0.149 mmol, 75%). For further analysis, starting from a smaller portion of the product, the diastereomers were separated using preparative thin layer chromatography with PE:ethylacetate (1.5:1) and *tert*-butyl-(1*R*,3*S*,3*aS*,6*aR*)-5-methyl-3-(4-nitrophenyl)-4,6-dioxooctahydropyrrolo[3,4-*c*]pyrrole-1-carboxylate **3nA** was isolated diastereomerically pure as a colourless solid. The *ee*-value (87%) was determined by HPLC on chiral stationary phase: Chiracel IA, cyclohexane/iPrOH (50:50), 1.0 mL/min, 220 nm, t_R = 19.217 min (minor), t_R = 44.873 min (major).

$C_{18}H_{21}N_3O_6$, MW: 375.38 g/mol. **Mp.**: 93-104 °C. **[α]$^{20}_{D}$ (*c* = 1.0 mg/mL, DCM, Sample with 87% *ee*):** –12. **^{1}H-NMR (700 MHz, $CDCl_3$):** δ = 8.22 (*d*, *J* = 8.7 Hz, 2H, Ar-*H*), δ = 7.70 (*d*, *J* = 8.7 Hz, 2H, Ar-*H*), δ = 4.55 (*d*, *J* = 5.7 Hz, 1H, NH-C*H*-Ph), δ = 4.03 (*d*, *J* = 4.9 Hz, 1H, NH-C*H*-CO_2Et), δ = 3.80 (*dd*, J = 4.8, 8.9 Hz, 1H, C*H*-CH-CO_2Et), δ = 3.39 (*dd*, *J* = 5.9, 9.0 Hz, 1H, C*H*-CH-Ph), δ = 3.05 (*s*, 3H, N-CH_3), δ = 2.70 (*br*, 1H, N*H*), δ = 1.46 (*s*, 9H, C(CH_3)$_3$). **^{13}C-NMR (176 MHz, $CDCl_3$):** δ = 177.0, 176.7, 170.4, 148.6, 147.9, 128.0, 124.2, 83.2, 64.9, 63.5, 53.1, 49.5, 28.3, 25.6. **IR ($CDCl_3$):** ṽ = 3344, 2980, 2930, 2857, 1779, 1699, 1601, 1520, 1435, 1382, 1369, 1347, 1281, 1247, 1156, 1129, 1015, 959, 916, 844, 734, 700, 649, 517. **HRMS (ESI) m/z:** Calculated for [M-H]$^+$ **$C_{18}H_{22}N_3O_6^+$**: 376.1503; Measured: 376.1499.

7.18 Synthesis of *tert*-butyl-(1*R*,3*S*,3a*S*,6a*R*)-4,6-dioxo-3,5-diphenyloctahydropyrrolo[3,4-*c*]pyrrole-1-carboxylate (3bC)

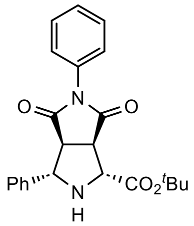

For the synthesis of **3bC** according to **GP6**,[2] the iminoester **1b** (52.6 mg, 0.24 mmol, 1.2 equiv.) and *N*-phenylmaleimide (34.6 mg, 0.20 mmol, 1.0 equiv.) are reacted under utilization of the catalyst **C7** (1.88 mg, 2.0 µmol, 1.0 mol%) in THF (100 µl) and methanol (4 µl, 0.10 mmol, 0.5 equiv.) as an additive at a temperature of 10 °C for 20 h. After workup of the reaction, using ^{1}H-NMR spectroscopy with mesitylene (25 µL) as an internal standard, the yield (83%) and diastereomeric ratio of *exo*:*endo* (90:10) was determined. After further purification using column chromatography with PE:ethylacetate (85:15) the product was obtained as a diastereomeric mixture (68.0 mg, 0.173 mmol, 87%). For further analysis, starting from a smaller portion of the product, the diastereomers were separated using preparative thin layer chromatography with PE:ethylacetate (1.5:1) and *tert*-butyl-(1*R*,3*S*,3a*S*,6a*R*)-4,6-dioxo-3,5-diphenyloctahydropyrrolo[3,4-*c*]pyrrole-1-carboxylate **3bC** was isolated diastereomerically pure as a colourless solid. The *ee*-value (91%) was determined by HPLC on chiral stationary phase: Chiracel IA, cyclohexane/iPrOH (50:50), 0.7 mL/min, 220 nm, t_R = 13.507 min (minor), t_R = 15.203 min (major).

$C_{23}H_{24}N_2O_4$, MW: 392.46 g/mol. **Mp.**: 141-148 °C. **[α]$^{20}_{D}$ (*c* = 1.0 mg/mL, DCM, Sample with 91% *ee*):** –24. **^{1}H-NMR (300 MHz, $CDCl_3$):** δ = 7.53-7.28 (*m*, 10H, Ar-*H*), δ = 4.64 (*d*, *J* = 4.7 Hz, 1H, NH-C*H*-Ph), δ = 4.10 (*d*, *J* = 4.0 Hz, 1H, NH-C*H*-CO_2Et), δ = 3.97 (*dd*, J = 4.2, 8.7 Hz, 1H, C*H*-CH-CO_2Et), δ = 3.62 (*dd*, *J* = 5.0, 8.7 Hz, 1H, C*H*-CH-Ph), δ = 2.82 (*br*, 1H, N*H*), δ = 1.41 (*s*, 9H, C(CH_3)$_3$). **^{13}C-NMR (176 MHz, $CDCl_3$):** δ = 176.4, 176.2, 170.8, 140.9, 132.0, 129.5, 129.2, 129.1, 128.4, 127.1, 126.7, 83.0, 66.1, 64.0, 52.4, 49.7, 28.2. **IR ($CDCl_3$):**

ṽ = 3340, 3064, 2978, 2928, 2853, 2185, 2013, 1715, 1599, 1497, 1456, 1370, 1237, 1157, 913, 844, 747, 694, 620, 517, 470. **HRMS (ESI) m/z:** Calculated for $[M-H]^+$ $\mathbf{C_{23}H_{25}N_2O_4^+}$: 393.1809; Measured: 393.1804.

7.19 Synthesis of *tert*-butyl-(1*R*,3*S*,3a*S*,6a*R*)-5-benzyl-4,6-dioxo-3-phenyloctahydropyrrolo[3,4-*c*]pyrrole-1-carboxylate (3bD)

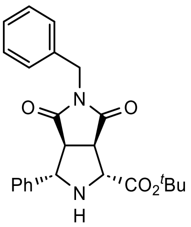

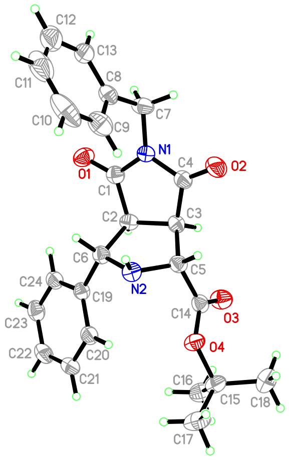

For the synthesis of **3bD** according to **GP6**,[2] the iminoester **1b** (52.6 mg, 0.24 mmol, 1.2 equiv.) and *N*-benzylmaleimide (37.4 mg, 0.20 mmol, 1.0 Äq.) are reacted under utilization of the catalyst **C7** (1.88 mg, 2.0 µmol, 1.0 mol%) in THF (100 µl) and methanol (4 µl, 0.10 mmol, 0.5 equiv.) as an additive at a temperature of 10 °C for 20 h. After workup of the reaction, using ^{1}H-NMR spectroscopy with mesitylene (25 µL) as an internal standard, the yield (99%) and diastereomeric ratio of *exo*:*endo* (91:9) was determined. After further purification using column chromatography with PE:ethylacetate (70:30) the product was obtained as a diastereomeric mixture (80.9 mg, 0.199 mmol, >99%). For further analysis, starting from a smaller portion of the product, the diastereomers were separated using preparative thin layer chromatography with PE:ethylacetate (1.5:1) and *tert*-butyl-(1*R*,3*S*,3a*S*,6a*R*)-5-benzyl-4,6-dioxo-3-phenyloctahydropyrrolo[3,4-*c*]pyrrole-1-carboxylate **3bD** was isolated diastereomerically pure as a colourless solid. The *ee*-value (91%) was determined by HPLC on chiral stationary phase: Chiracel IA, cyclohexane/iPrOH (50:50), 1.0 mL/min, 220 nm, t_R = 9.507 min (minor), t_R = 13.487 min (major).

$\mathbf{C_{24}H_{26}N_2O_4}$**, MW:** 406.48 g/mol. **Mp.**: 129-133 °C. $[\alpha]^{20}_D$ **(*c* = 1.0 mg/mL, DCM, Sample with 91% *ee*):** –26. **^{1}H-NMR (300 MHz, CDCl$_3$):** δ = 7.47-7.27 (*m*, 10H, Ar-*H*), δ = 4.69 (*s*, 2H, C*H*$_2$-Ph), δ = 4.44 (*d*, *J* = 5.1 Hz, 1H, NH-C*H*-Ph), δ = 3.93 (*d*, *J* = 4.4 Hz, 1H, NH-C*H*-CO$_2$Et), δ = 3.79 (*dd*, J = 4.3, 8.9 Hz, 1H, C*H*-CH-CO$_2$Et), δ = 3.45 (*dd*, *J* = 5.3, 8.7 Hz, 1H, C*H*-CH-Ph), δ = 2.64 (*br*, 1H, N*H*), δ = 1.39 (*s*, 9H, C(C*H*$_3$)$_3$). **^{13}C-NMR (176 MHz, CDCl$_3$):** δ = 177.0, 176.8, 170.7, 140.8, 136.0, 129.1, 129.1, 129.0, 128.4, 128.3, 127.0, 82.9, 65.6, 63.6, 52.5, 49.6, 43.0, 28.2. **IR (CDCl$_3$):** ṽ = 3338, 3064, 3033, 2978, 2931, 1776, 1702, 1604, 1496, 1455,

1431, 1393, 1368, 1343, 1314, 1249, 1155, 1085, 1030, 984, 897, 843, 751, 734, 700, 626, 540, 471. **HRMS (ESI) m/z:** Calculated for [M-H]$^+$ $\mathbf{C_{24}H_{27}N_2O_4^+}$: 407.1965; Measured: 407.1955.

8 Determination of the relative and absolute configuration of pyrrolidines

For the pyrrolidines unknown to literature, the relative configuration was determined using 2D NMR spectroscopy. The utilized procedure will be explained below for the *endo*- and *exo*-configured products **3fA** and **3mA**.

The spectrum in Figure S1 shows an overlay of the HSQC (blue signals) and HMBC (red signals) for the **3fA**. In the ^{1}H-NMR spectrum, the four ring protons split into two doublets and two triplets. In the Figure S1, a coupling can be seen in the HMBC spectrum between the downfield-shifted doublet and the aromatic signals in the ^{13}C-NMR spectrum. This indicates that this signal can be assigned to the proton at the 3R carbon atom. The upfield-shifted doublet in the ^{1}H spectrum exhibits two couplings with the carbonyl carbon atoms and can therefore be assigned to the proton at the 1S carbon atom.

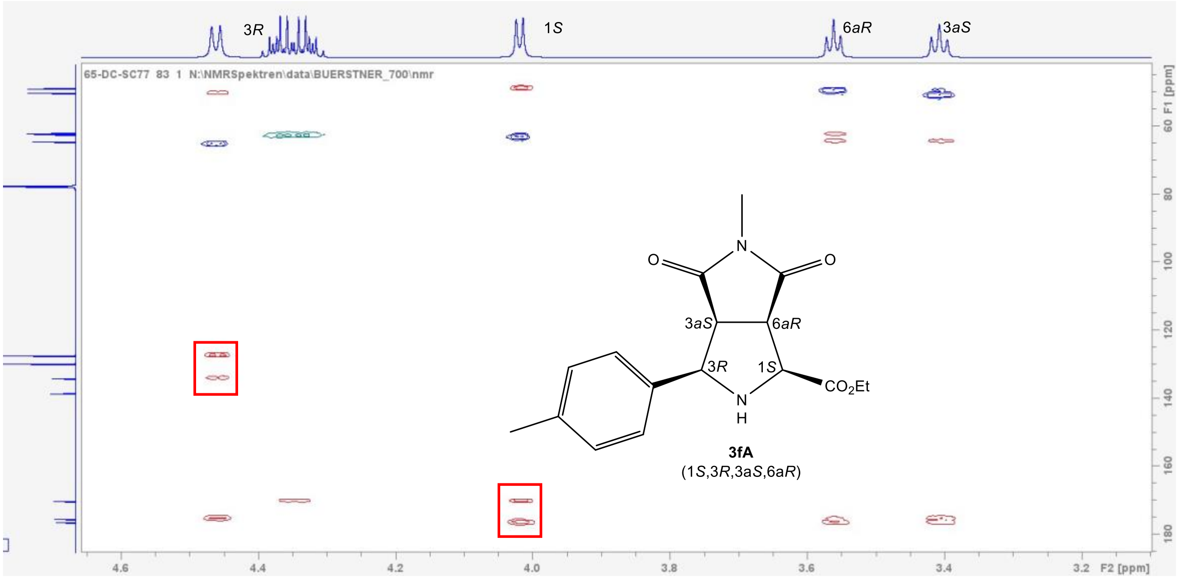

Figure S1: Overlay of the HSQC (blue signals) and HMBC spectra (red signals) of the endo-configured 3fA product.

As the two ring protons can be assigned to the 3R and 1S carbon atoms, the two triplet signals can be assigned via proton coupling in the COSY spectrum. As shown in Figure S2, the downfield-shifted triplet can be assigned to the proton at the 6aR carbon atom. The upfield-shifted triplet can be assigned to the proton at the 3aS carbon atom.

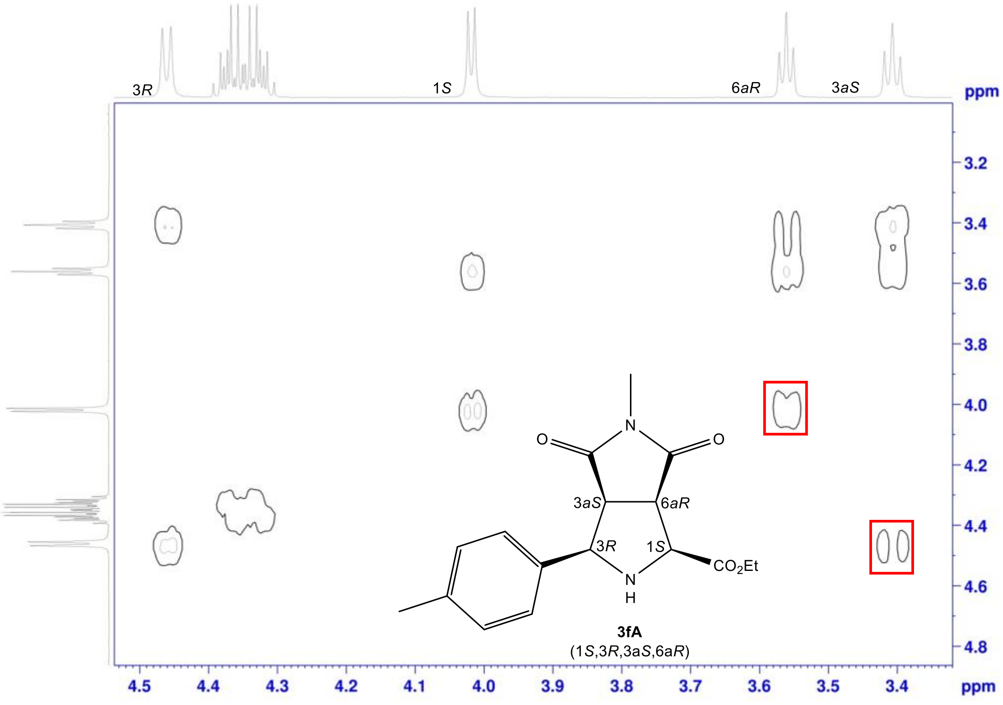

Figure S2: The COSY spectrum of the endo-configured 3fA product.

The relative configuration of the product was determined via spatial coupling in the NOESY spectrum (Figure S3). Each doublet signal couples to a triplet signal, and each triplet signal couples to the other triplet and a doublet signal, which means that all protons are located on one side of the ring, indicating an endo-configuration of **3fA**.

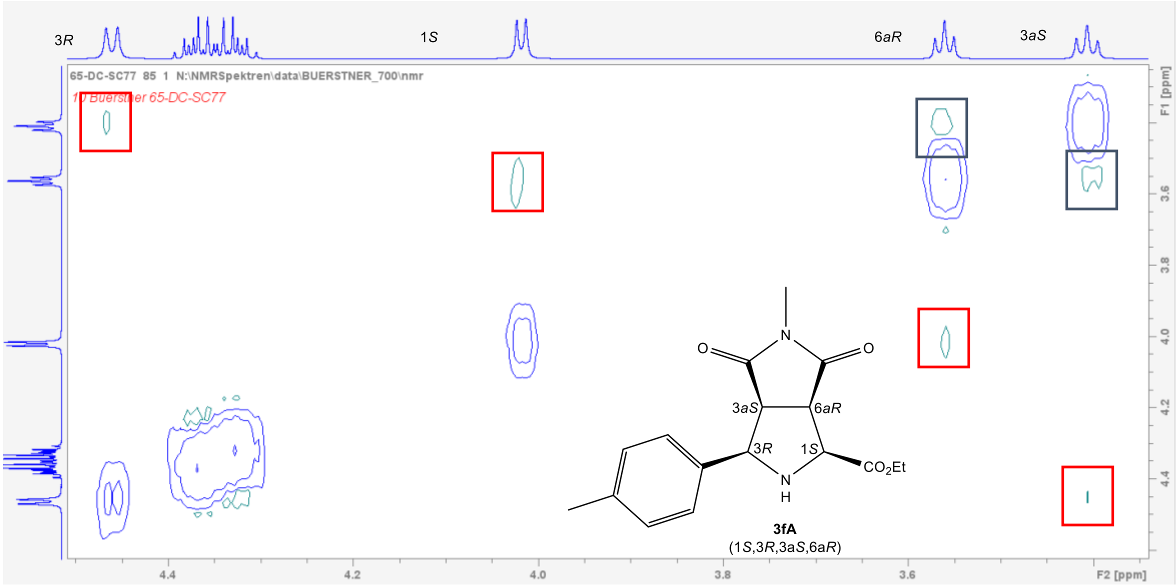

Figure S3: The NOESY spectrum of the endo-configured 3fA product.

For **3mA** the characterization is shown below. For this product the ring protons of the pyrrolidine moiety split into two doublets and two doublets of doublets. By the HMBC spectrum, the downfield-shifted proton can be assigned to the 3S carbon atom, and the upfield-shifted proton to the 1R proton.

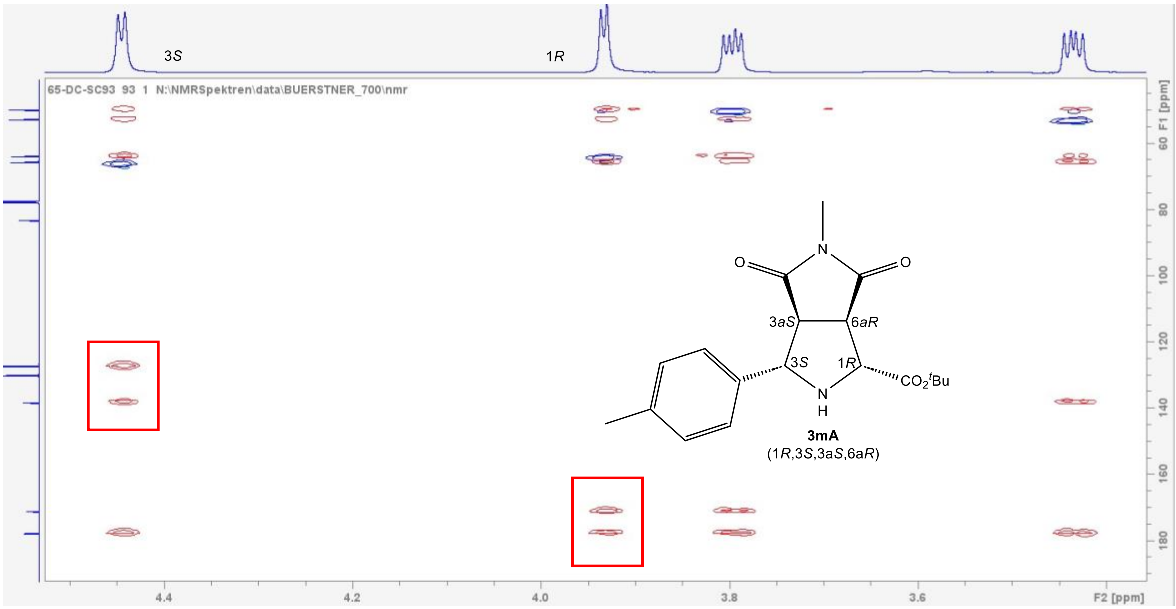

Figure S4: Overlay of the HSQC (blue signals) and HMBC spectra (red signals) of the exo-configured 250-D2-E2 product.

Via proton/proton coupling in the COSY spectrum in Figure S5, the two doublet of doublet signals were assigned. As marked in the figure, upfield-shifted doublet of doublet is the proton at the 3aS carbon atom. The downfield-shifted doublet of doublet is the proton at the 6aR carbon atom.

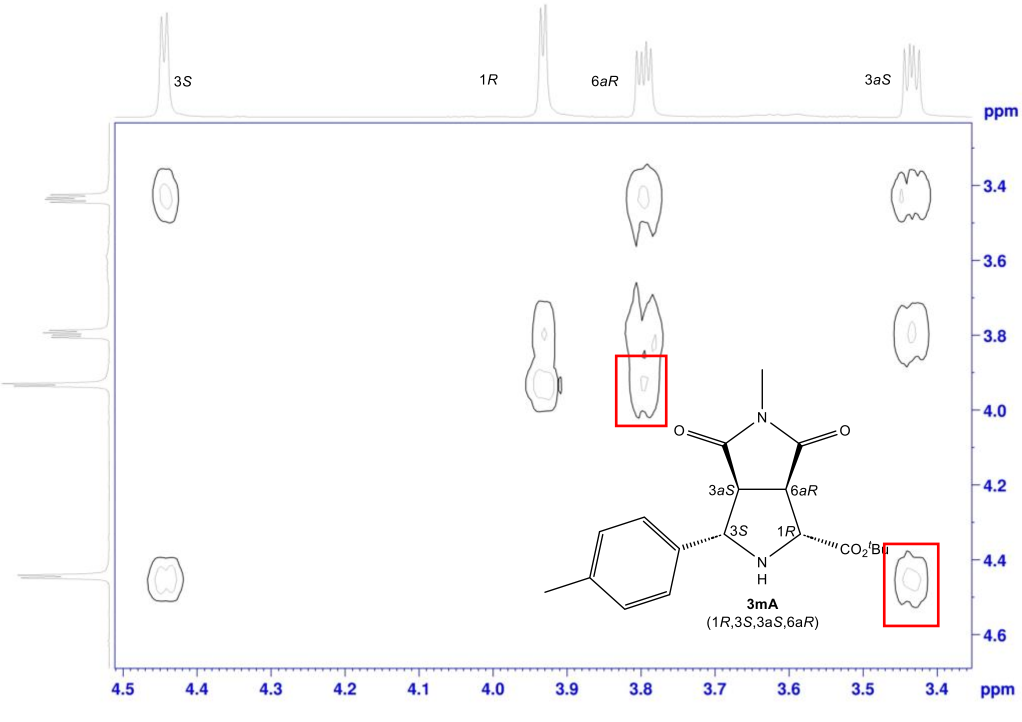

Figure S5: The COSY spectrum of the exo-configured 3mA product.

The relative configuration is determined in the NOESY spectrum based on the spatial coupling in Figure S6. As marked in the figure, for the product **3mA**, coupling is exclusively found between the two doublets of doublets. The two doublets of doublet signals do not couple with the doublet signals. This means that they are located on opposite sides of the ring. From this, it can be concluded that the product **3mA** obtains an *exo*-configuration.

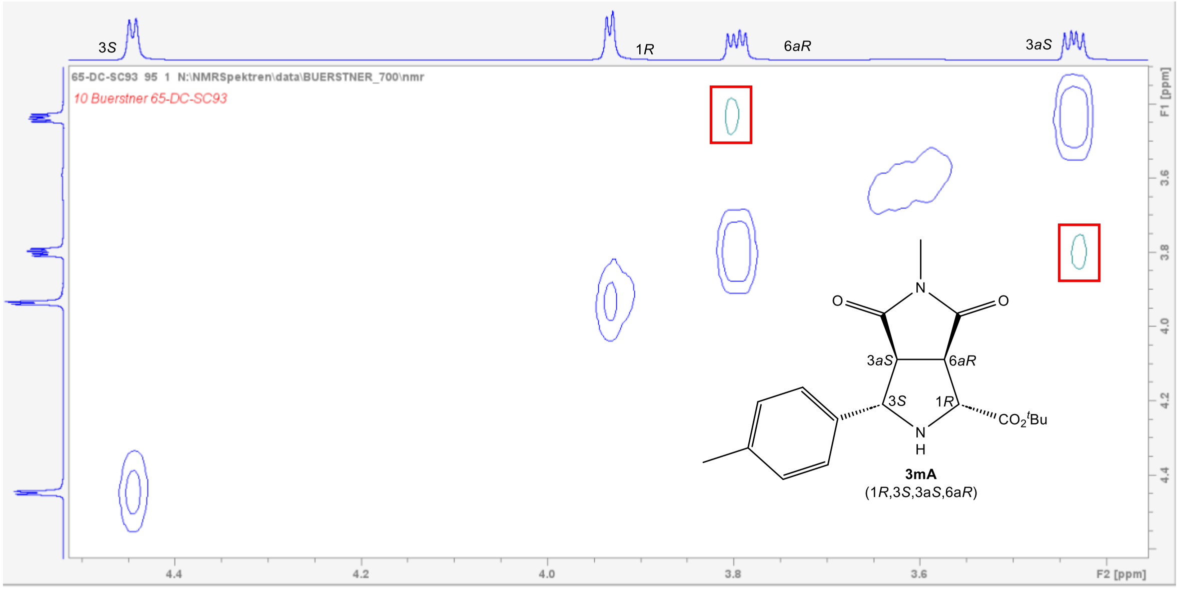

Figure S6: The NOESY spectrum of the exo-configured 3mA product.

To determine the absolute configuration of products not known in the literature, single crystals of a highly enantiomerically enriched compound were measured by X-ray diffraction. To obtain single crystals for the pyrrolidines, recrystallization was performed from a mixture of *n*-hexane and dichloroethane. The crystal structures were determined by Dr. Wolfgang Frey of the Institute of Organic Chemistry at the University of Stuttgart.

In the endo-selective model reaction using the Co(II) imidazolium phenoxyimine complex **C4**, a single crystal was obtained for the endo-configured product **3aA**. By X-ray diffraction the crystal structure in Figure S7 was determined with an absolute configuration of (1S,3R,3aS,6aR). Due to the well-defined binding pocket of the Co(II)-imidazolium-phenoxyimine catalyst and the specific polyfunctional activation principle of both reaction partners, it was assumed that all endo-configured cycloaddition products formed by **C4** have the same absolute configuration of (1S,3R,3aS,6aR).

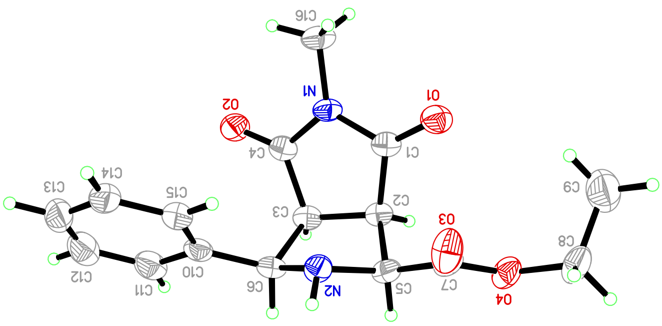

Figure S7: Crystal structure of the endo-configured product 3mA with an absolute configuration of (1S,3R,3aS,6aR). Measured and determined by Dr. W. Frey.

For the exo-selective reaction using the Ni(II) triazolium phenoxyimine complex **C7**, a crystal structure of **3bA** (Figure S8) could be determined with an absolute configuration of (1R,3S,3aS,6aR) was determined. This product is not known to the literature.

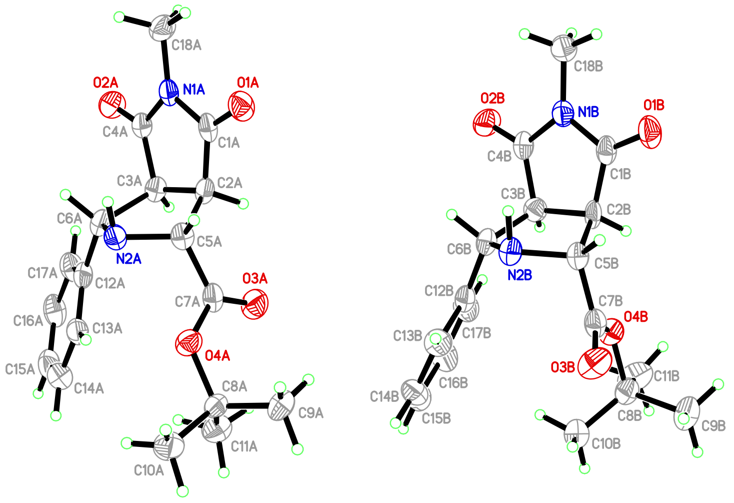

Figure S8: Crystal structure of the exo-configured product 3bA with an absolute configuration of (1R,3S,3aS,6aR). Measured and determined by Dr. W. Frey.

Another crystal structure was obtained for **3bD** (Figure S9). Also in this case, an absolute configuration (1R,3S,3aS,6aR) was determined.

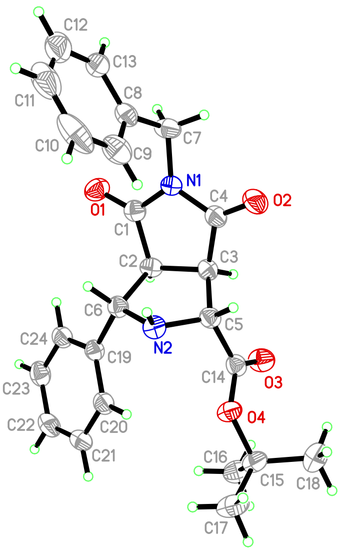

Figure S9: The crystal structure of the exo-configured product 3bA with an absolute configuration of (1R,3S,3aS,6aR) measured by W. Frey using X-ray diffraction.

Due to the well-defined binding pocket of the Ni(II)-imidazolium-phenoxyimine catalyst and the specific polyfunctional activation principle of both reaction partners, it was assumed that all exo-configured cycloaddition products formed by **C7** have the same absolute configuration of (1R,3S,3aS,6aR).

9 Kinetic studies

9.1 VTNA

For the kinetic investigations the method of *Visual Kinetic Analysis* was used [25], which allows to obtain mechanistic information from the visual comparison of reaction profiles.

In this work, the *Variable Time Normalization Analysis* (VTNA) method developed by *J. Bures*[25–27] was applied. The principle of this method is based on the fact that the concentration profiles from different experiments in which the concentration of a component is varied can only be superimposed if their time axis is replaced by the integral of the concentration of component A, which carries the kinetic order of component γ in the exponent.[25,27]:

$$\sum [A]^{\gamma} \Delta t = \sum_{i=1}^{n} \left(\frac{[A]_i + [A]_{i-1}}{2}\right)^{\gamma} (t_i - t_{i-1}) \tag{1}$$

If all *n* components of the reaction that influence the reaction rate are taken into account simultaneously in the normalization of the time scale for each reaction profile, all *n*+1 reaction profiles superimpose along a straight line. The slope of the straight corresponds to the experimental rate constant k_{obs}.[25,27]

9.1.1 Exo-selective model reaction

All concentration profiles required for this method were measured by ^1H NMR spectroscopy. The concentrations of individual components were determined by adding a defined amount of mesitylene (25 µL) as an internal standard. The procedure of the kinetic measurements using the VTNA method developed by J. Bures[25–27] will first be described by the *exo*-selective model reaction using the Ni(II) triazolium phenoxyimine complex **C7**. A substrate concentration of 0.5 M was used for the measurements.

In Table S1, the reaction conditions for kinetic measurements are listed. The VTNA analysis consisted of a total of five measurements. First, a reference measurement (KN045) was performed. In the subsequent measurements (KN46-49), the starting concentration was then changed for each reaction component (catalyst, maleimide, imino ester, and product) in each measurement.

Table S1: Concentrations and molar amounts of the catalyst C7, N-methylmaleimide 2A and the tert-butyl imino ester 1b in the VTNA experiments of the exo-selective model reaction.

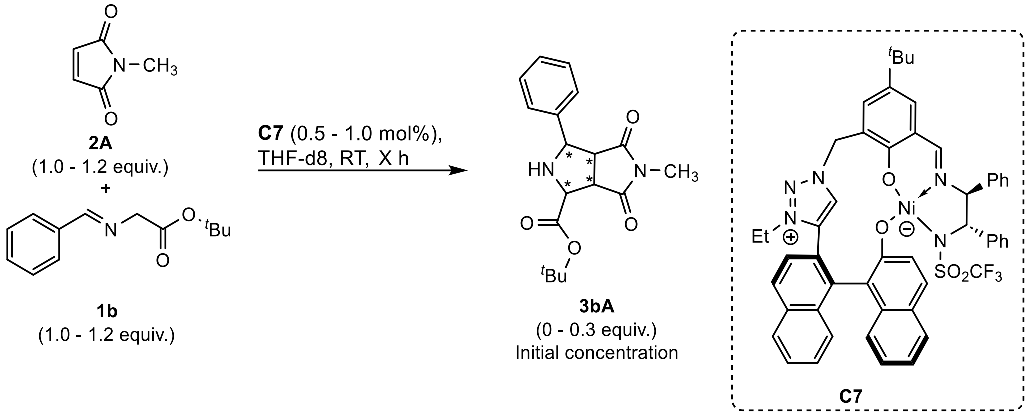

Nr.	[2A] mol/l	[2A] mmol	[1b] mol/l	[1b] mmol	[C7] mol/l	[C7] mmol	[3bA] mol/l	[3bA] mmol
KN045	**0.5**	0.25	**0.5**	0.25	**0.005**	0.0025	**0**	0
KN046	0.5	0.25	0.5	0.25	**0.0025**	0.00125	0	0
KN047	**0.6**	0.30	0.5	0.25	0.005	0.0025	0	0
KN048	0.5	0.25	**0.6**	0.30	0.005	0.0025	0	0
KN049	0.5	0.25	0.5	0.25	0.005	0.0025	**0.15**	0.075

As an example, Figure S10 shows the concentration profiles of the maleimide **2A**, the iminoester **1b** and the product **3bA** for the measurement KN045.

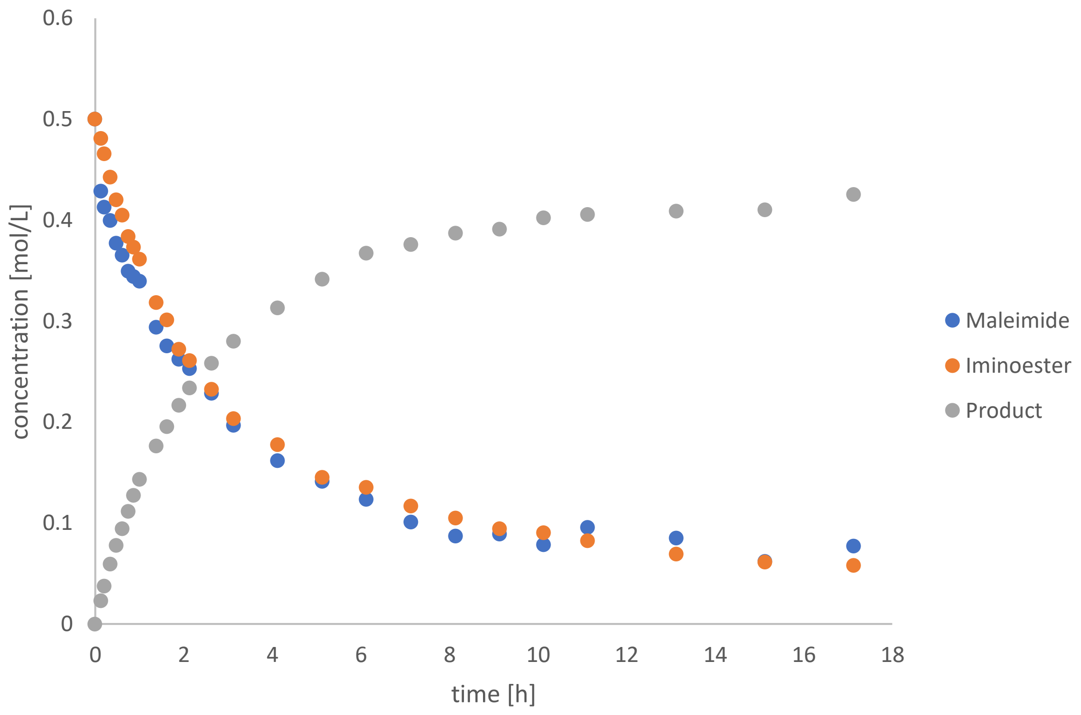

Figure S10: Concentration profiles for the N-methylmaleimide 2A, the tert-butyl imino ester 1b and the product 3bA for the VTNA experiment KN045 of the exo-selective model reaction.

We used the automatic integration program *intser* from *Bruker Topspin* to determine the concentrations of the reaction components. During the evaluation of the measurements, we encountered the problem, that the proton signal used to determine the concentration of maleimide **2A** overlaps with the proton signal of the *exo*-configured side product. To prevent falsification, the deconvolution tool (*dcon*) from *Bruker Topspin* was used to separate the two overlapping signals. As shown in Figure S11, the overlapping signals were approximated and separated using a *Lorentzian* function.

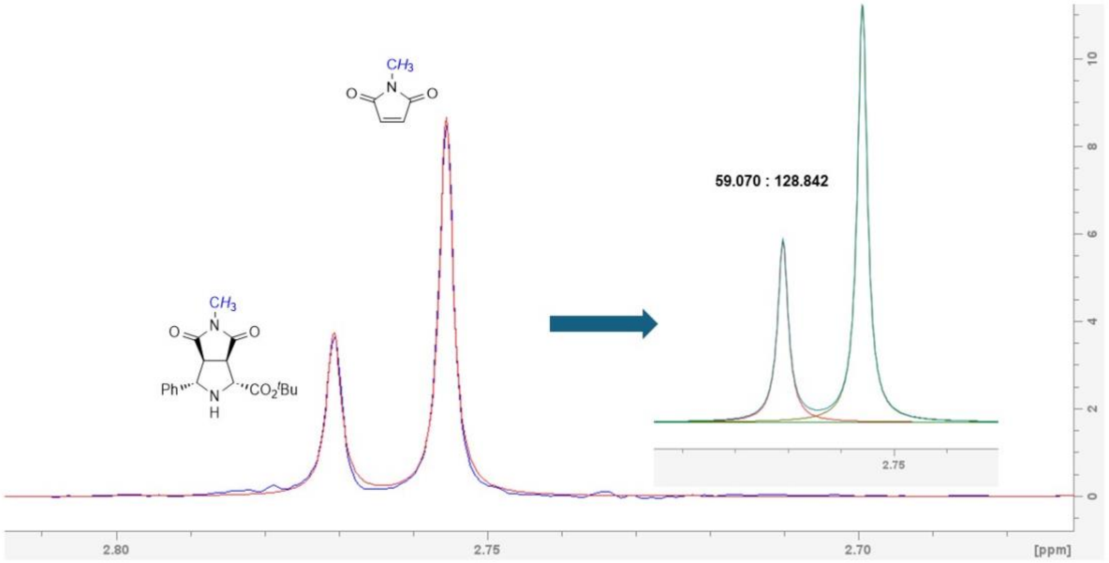

Figure S11: Separation of the superimposed signals in the ^{1}H NMR of the methyl groups of N-methylmaleimide 2A and the exo-configured product 3bA with the deconvolution tool (dcon) from Bruker topspin by an approximation with a Lorentzian function.

The described procedure was repeated for all measurement points of the KN045-49 measurement series. The resulting concentration curves of KN045 are shown in Figure S12.

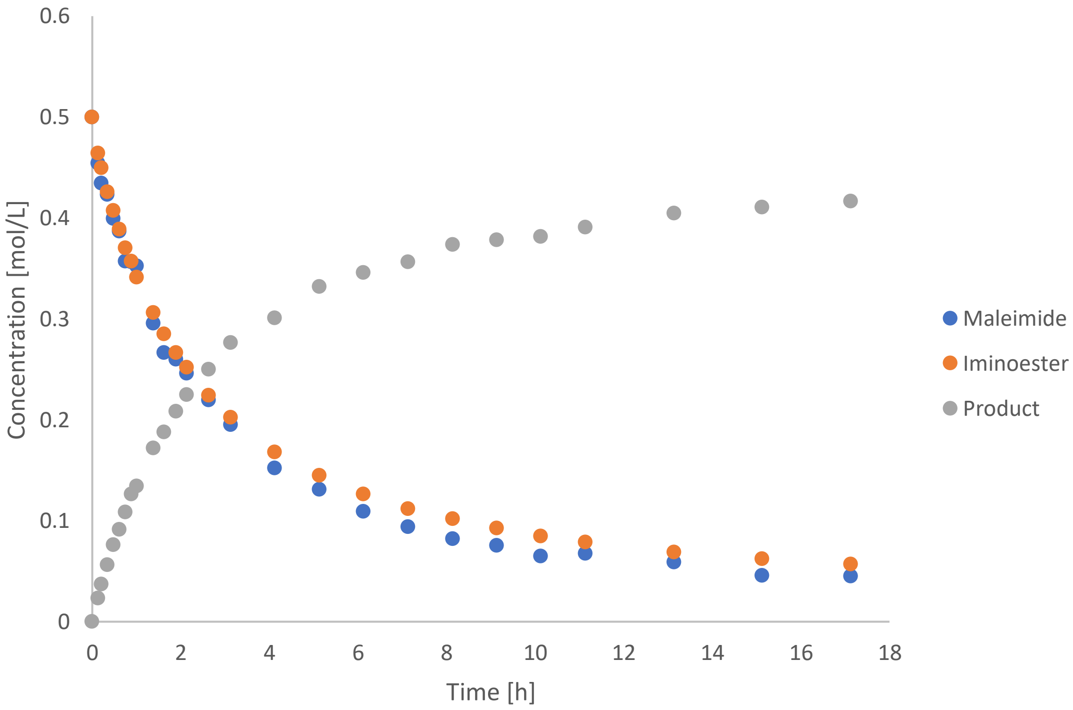

Figure S12: Concentration profiles for the N-methylmaleimide 2A, the tert-butyl imino ester 1b and the product 3bA for the VTNA experiment KN045 of the exo-selective model reaction.

The determination of the partial reaction orders γ should be exemplified for the iminoester **1b** for the experiments KN045 and KN048, in which the iminoester concentration is varied. For the evalueation, the imino ester concentration curves were used. As depicted in Figure S13, the time axes were normalized by formula (1) for different partial reaction orders of the imino ester until an overlap of the curves was observed. As shown in Figure S13, this procedure was conducted for partial reaction orders of γ = 0, γ = 0.9, and γ = 2.0. The best overlap of the imino ester concentration curves was achieved for a partial reaction order of γ = 0.9.

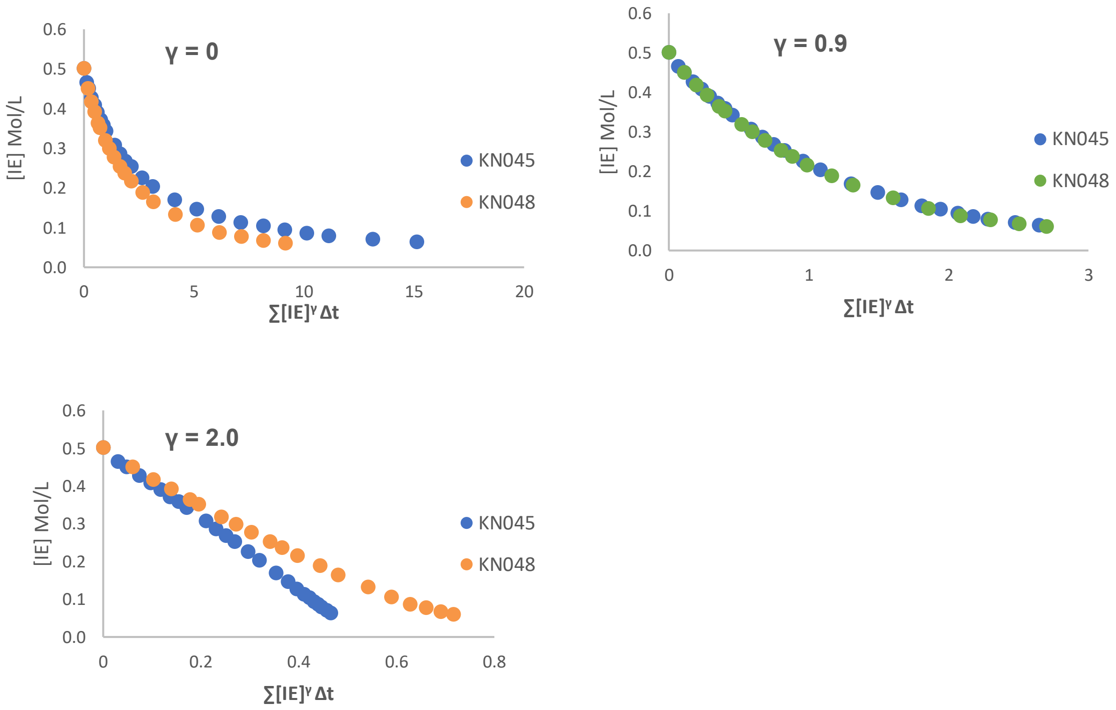

Figure S13: Determination of a partial reaction order γ for the imino ester 1b using VTNA, for which the best superposition of the concentration curves of the imino ester is obtained (KN045 vs. KN048).

The same procedure was performed for the maleimide **2A** (Figure S14, top right), the iminoester **1b** (Figure S14, top left), the catalyst **C7** (Figure S14, bottom left), and the product **3bA** (Figure S14, bottom right). The partial reaction orders of the components, which resulted in the best superposition of the curves, are shown in Figure S14.

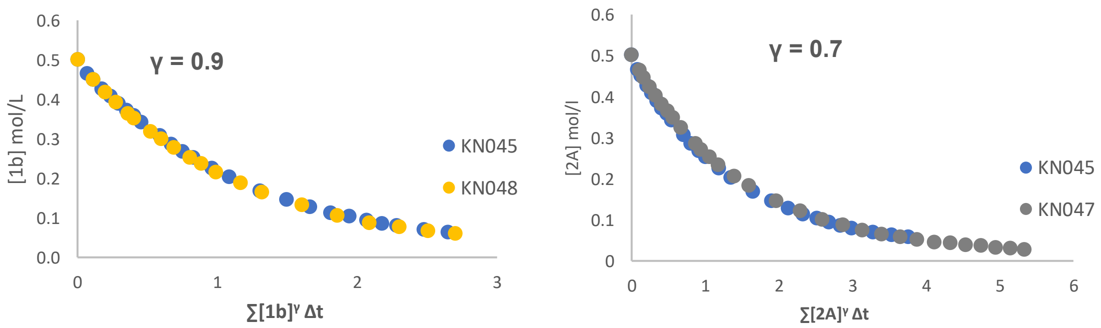

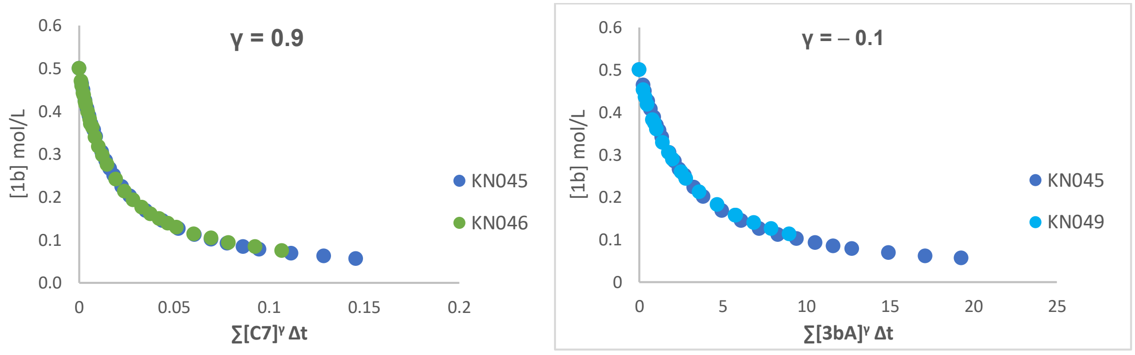

Figure S14: The partial reaction orders γ for the imino ester 1b (top left), the maleimide 2A (top right), the catalyst C7 (bottom left) and the 3bA (bottom right), for which the best superposition of the concentration curves was obtained using VTNA.

For the final linearization the previously determined partial reaction orders were used. For the evaluation, the concentration curves of the imino ester for experiment KN045-49 were used. The slope of the resulting straight corresponds to the experimental rate constant k_{obs}. Due to the visual evaluation, the VTNA method does not allow for mathematical error analysis.[25,27]

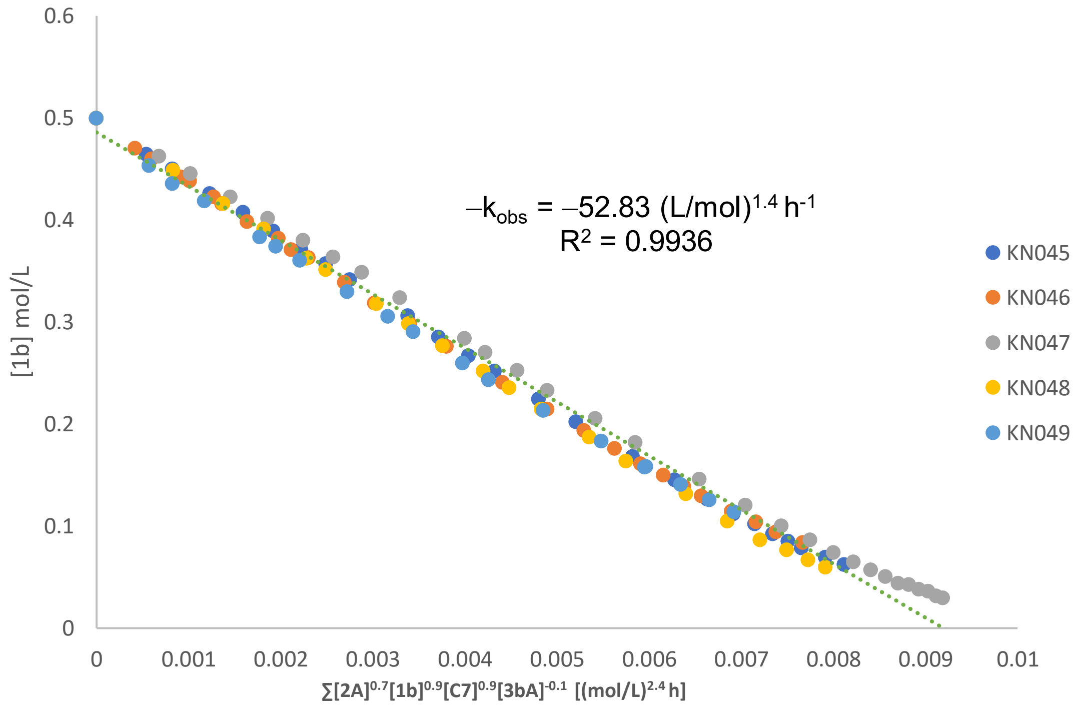

Figure S15: Linearization of the concentration curves of the imino ester from the measurements KN045-49 by means of VTNA using the partial reaction orders determined in Figure S14.

The experimental rate law for the exo-selective model reaction using **C7** can be determined as:

$$r = 52.83\,[C7]^{0.9}[1b]^{0.9}[2A]^{0.7}[3bA]^{-0.1}$$

9.1.2 Endo-selective model reaction

A similar measurement procedure was performed for the *endo*-selective cycloaddition using the Co(II)-imidazolium-phenoxyimine catalyst **C4**. An overview of the experiments performed and reaction conditions is given in Table S2.

Table S2: Concentrations and molar amounts of the catalyst C4, N-methylmaleimide 2A and the ethyl imino ester 1a in the VTNA experiments of the endo-selective model reaction.

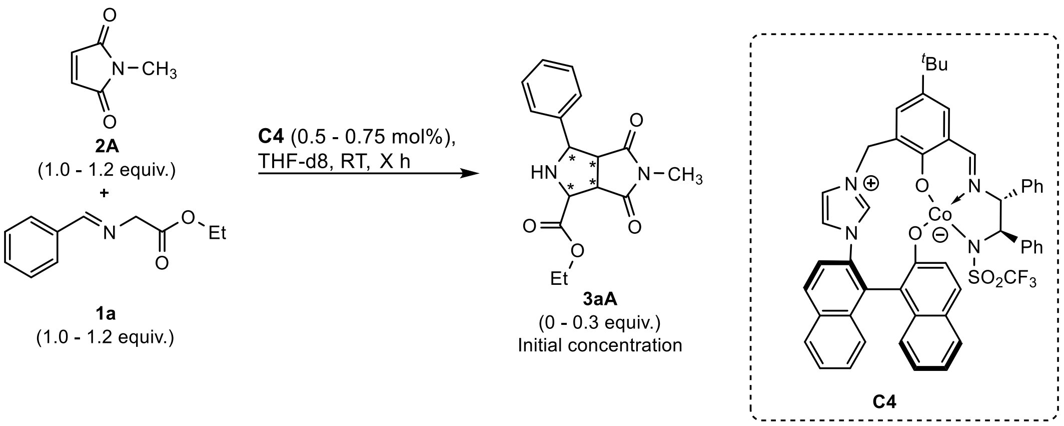

Nr.	[2A] mol/l	[2A] mmol	[1a] mol/l	[1a] mmol	[C4] mol/l	[C4] mmol	[3aA] mol/l	[3aA] mmol
KN040	**0.2**	0.15	**0.2**	0.15	**0.001**	0.00075	**0**	0
KN039	0.2	0.15	0.2	0.15	**0.0014**	0.00105	0	0
KN041	**0.24**	0.18	0.2	0.15	0.001	0.00075	0	0
KN042	0.2	0.15	**0.26**	0.19	0.001	0.00075	0	0
KN043	0.2	0.15	0.2	0.15	0.001	0.00075	**0.06**	0.045

As an example, Figure S16 shows the concentration profiles of the maleimide **2A**, the iminoester **1a** and the product **3aA** for the measurement KN040.

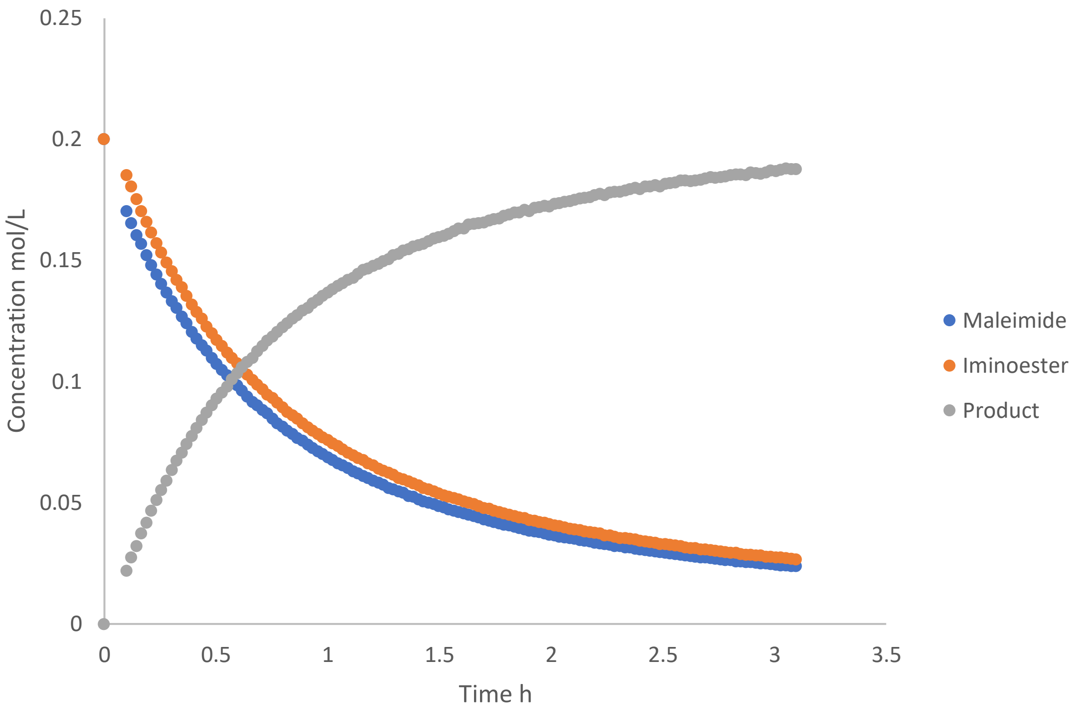

Figure S16: Concentration profiles for the N-methylmaleimide 2A, the ethyl imino ester 1a and the product 3aA for the VTNA experiment KN040 of the endo-selective model reaction.

As previously described for the endo-selective reaction using the Co(II)-imidazolium-phenoxyimine complex **C4**, for the exo-selective reaction using the Ni-triazolium-phenoxyimine complex, the ^{1}H-NMR signal for determining the concentration of the maleimide is superimposed by a proton signal on the exo-configured product **3aA**. For this reason, the *Bruker Topspin* deconvolution tool (*dcon*) was also used for the endo-selective reaction to separate the superimposed signals. Due to the the large number of measurement points with very little scatter, measurement points were selected at regular time intervals for further analysis. The obtained concentration profiles are shown in Figure S17.

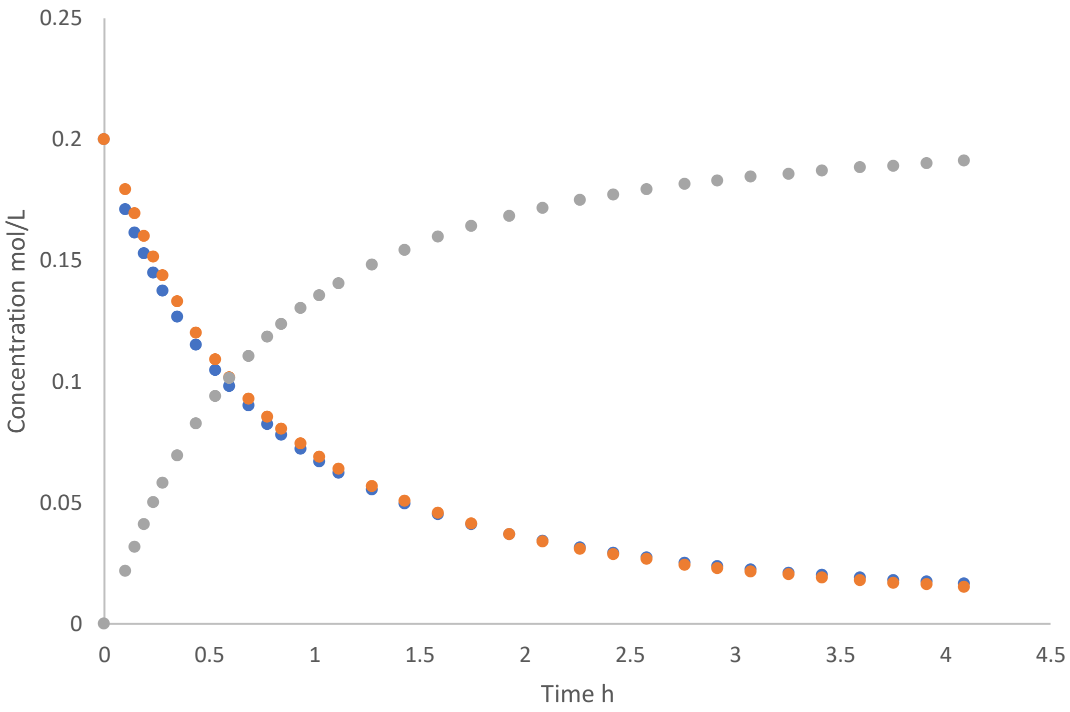

Figure S17: Concentration profiles for the N-methylmaleimide 2A, the ethyl imino ester 1a and the product 3aA for the VTNA experiment KN040 of the endo-selective model reaction.

Using the concentration curves of the maleimide, the time scale was normalized analogously to the procedure described for the Ni(II) catalyst **C7**. The resulting partial reaction orders of N-methylmaleimide, the tert-butylimino ester, the catalyst, and the product, for which the best superposition of the concentration profiles from the measurements was obtained are shown in Figure S18.

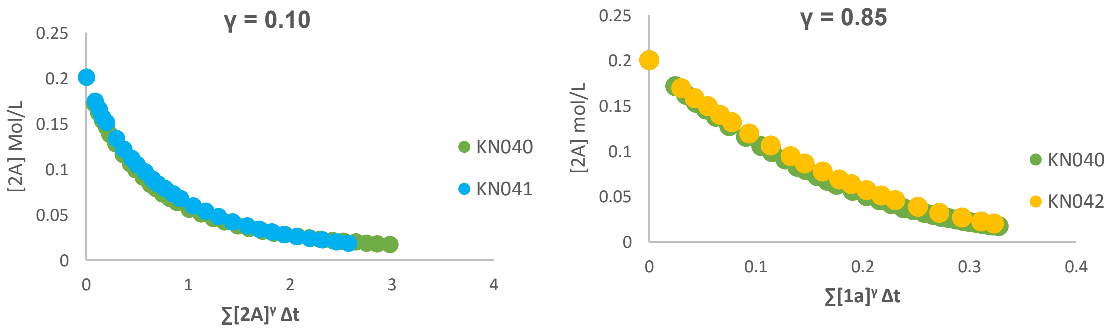

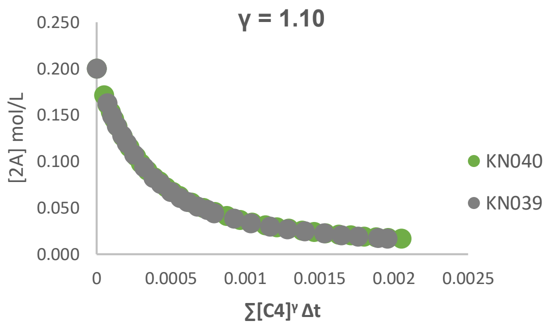

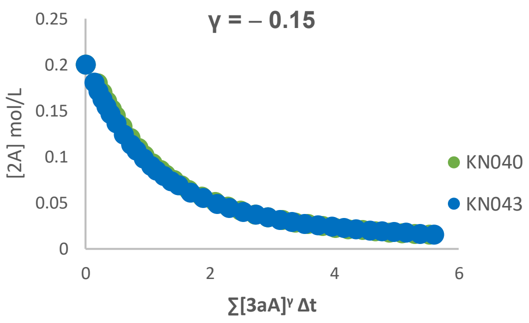

Figure S18: The partial reaction orders γ for the maleimide 2A (top left), the imino ester 1a (top right), the catalyst C4 (bottom left) and the product 3aA (bottom right), for which the best superposition of the concentration curves was obtained using VTNA.

For the final linearization the previously determined partial reaction orders were used. For the evaluation, the concentration curves of the imino ester for experiment KN039-43 were used. The slope of the straight corresponds to the experimental rate constant k_{obs}. Due to the visual evaluation, the VTNA method does not allow for mathematical error analysis.[25,27]

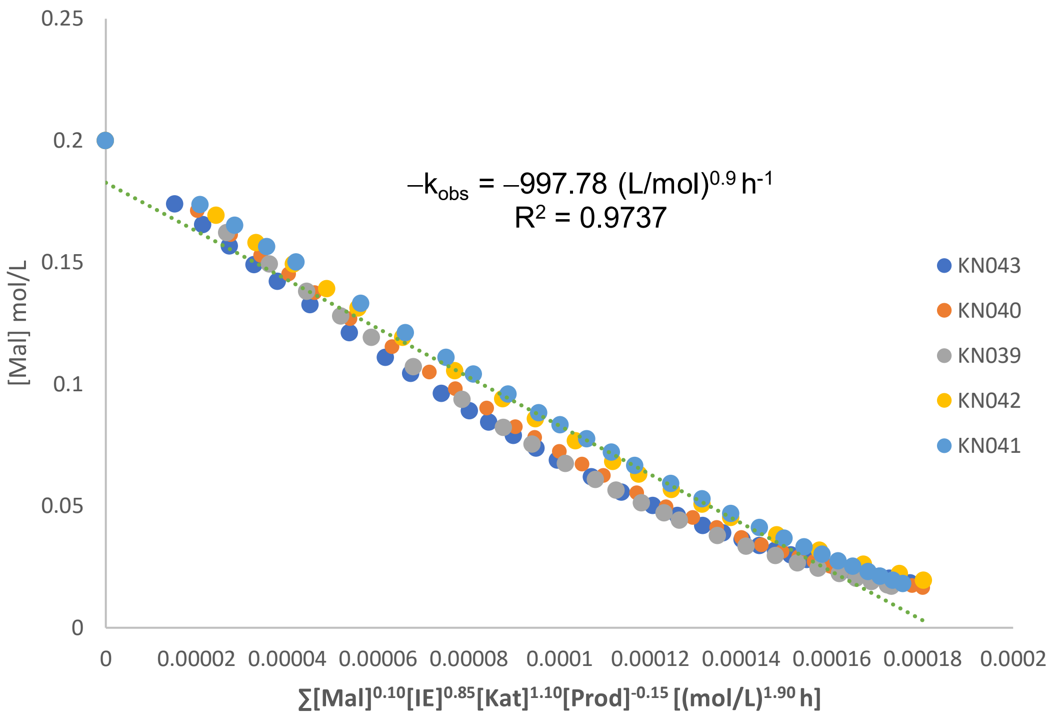

Figure S19: Linearization of the maleimide concentration curves from the measurements KN039-43 by VTNA using the partial reaction orders determined in Figure S18.

The experimental rate law for the exo-selective model reaction using **C4** can be determined as:

$$r = 997.78\ [C4]^{1.10}[1a]^{0.85}[2A]^{0.1}[3aA]^{-0.15}$$

The activation barrier can be calculated from the determined rate constant k_{obs}:[4,28]

$$\Delta^{\ddagger}G_{exp} = -RTln\left(\frac{k_{obs} \cdot h}{k_B \cdot T}\right) \tag{1}$$

with the universal gas constant R, the temperature T = 298 K, the *Plank* constant h, the *Boltzmann* constant k_B und k_{obs} = 0.28 1/s.

The determined rate constant k_{obs} for the *endo*-selective model reaction using **C4** results in an experimental activation barrier of $\Delta^{\ddagger}G_{exp}$ = 76.14 kJ/mol for the overall reaction.

9.2 NMR Data for VTNA

Exo-Cycloaddition (KN045)

Nr.	t h	[2A] mol/L	[1b] mol/L	[3bA] mol/L
1	0.000	0.500	0.500	0.000
2	0.133	0.455	0.464	0.023
3	0.216	0.435	0.450	0.037
4	0.349	0.424	0.426	0.057
5	0.483	0.400	0.407	0.076
6	0.616	0.387	0.389	0.091
7	0.749	0.357	0.371	0.109
8	0.883	0.356	0.357	0.126
9	1.016	0.353	0.341	0.134
10	1.389	0.296	0.306	0.172
11	1.630	0.267	0.285	0.188
12	1.894	0.260	0.267	0.208
13	2.144	0.246	0.252	0.225
14	2.637	0.220	0.224	0.250
15	3.135	0.196	0.202	0.276
16	4.133	0.152	0.168	0.301
17	5.133	0.131	0.145	0.332
18	6.133	0.109	0.127	0.346
19	7.135	0.094	0.112	0.356
20	8.148	0.082	0.102	0.374
21	9.137	0.076	0.093	0.378
22	10.136	0.065	0.085	0.382
23	11.136	0.068	0.079	0.391
24	13.140	0.059	0.069	0.405
25	15.135	0.046	0.062	0.411
26	17.139	0.045	0.057	0.417

Exo-Cycloaddition (KN046)

Nr.	t h	[2A] mol/L	[1b] mol/L	[3bA] mol/L
1	0.000	0.500	0.500	0.000
2	0.183	0.452	0.470	0.016
3	0.278	0.474	0.460	0.027
4	0.451	0.442	0.443	0.043
5	0.511	0.407	0.438	0.050
6	0.683	0.417	0.423	0.066
7	0.745	0.412	0.416	0.068
8	0.940	0.413	0.399	0.080
9	1.201	0.380	0.382	0.097
10	1.311	0.380	0.371	0.109
11	1.479	0.364	0.363	0.121
12	1.848	0.347	0.339	0.140
13	2.199	0.317	0.319	0.160
14	2.662	0.300	0.298	0.177
15	3.199	0.279	0.276	0.199
16	4.202	0.239	0.241	0.234
17	5.201	0.207	0.214	0.258
18	6.203	0.181	0.194	0.278
19	7.203	0.171	0.176	0.291
20	8.203	0.145	0.161	0.303
21	9.200	0.144	0.150	0.322
22	10.201	0.126	0.139	0.328
23	11.201	0.116	0.130	0.333
24	13.239	0.100	0.115	0.348
25	15.238	0.088	0.104	0.363
26	17.241	0.077	0.095	0.372
27	20.344	0.072	0.084	0.380
28	23.414	0.059	0.075	0.387

Exo-Cycloaddition (KN047)

Nr.	t h	[2A] mol/L	[1b] mol/L	[3bA] mol/L
1	0.000	0.600	0.500	0.000
2	0.150	0.549	0.463	0.029
3	0.239	0.538	0.445	0.045
4	0.364	0.527	0.423	0.066
5	0.498	0.483	0.402	0.090
6	0.637	0.482	0.380	0.108
7	0.764	0.463	0.364	0.122
8	0.898	0.424	0.349	0.144
9	1.098	0.399	0.324	0.166
10	1.499	0.363	0.284	0.202
11	1.649	0.349	0.271	0.214
12	1.899	0.330	0.253	0.233
13	2.161	0.316	0.233	0.251
14	2.649	0.282	0.205	0.280
15	3.150	0.255	0.182	0.303
16	4.162	0.222	0.146	0.336
17	5.150	0.192	0.120	0.366
18	6.148	0.171	0.100	0.378
19	7.151	0.152	0.086	0.392
20	8.149	0.149	0.074	0.407
21	9.146	0.139	0.065	0.414
22	10.161	0.135	0.057	0.422
23	11.151	0.119	0.051	0.423
24	12.184	0.122	0.044	0.431
25	13.163	0.109	0.043	0.436
26	14.151	0.111	0.038	0.444
27	15.149	0.102	0.036	0.447
28	16.149	0.095	0.032	0.446
29	17.154	0.094	0.029	0.451
30	18.154	0.094	0.026	0.450

Exo-Cycloaddition (KN048)

Nr.	t h	[2A] mol/L	[1b] mol/L	[3bA] mol/L
1	0.000	0.500	0.600	0.000
2	0.183	0.444	0.549	0.037
3	0.334	0.423	0.516	0.065
4	0.475	0.380	0.491	0.090
5	0.644	0.360	0.462	0.113
6	0.731	0.343	0.451	0.129
7	0.978	0.312	0.418	0.158
8	1.154	0.288	0.398	0.179
9	1.364	0.270	0.377	0.197
10	1.654	0.241	0.352	0.228
11	1.864	0.217	0.336	0.240
12	2.154	0.209	0.315	0.260
13	2.665	0.167	0.287	0.290
14	3.150	0.148	0.264	0.310
15	4.165	0.105	0.232	0.344
16	5.154	0.083	0.205	0.357
17	6.154	0.069	0.186	0.375
18	7.153	0.055	0.177	0.394
19	8.152	0.045	0.167	0.404
20	9.152	0.032	0.160	0.408
21	10.152	0.031	0.153	0.414

Exo-Cycloaddition (KN049)

Nr.	t h	[2A] mol/L	[1b] mol/L	[3bA] mol/L
1	0.000	0.500	0.500	0.150
2	0.183	0.453	0.453	0.168
3	0.274	0.464	0.436	0.184
4	0.407	0.410	0.419	0.199
5	0.671	0.384	0.383	0.237
6	0.757	0.370	0.374	0.242
7	0.890	0.343	0.361	0.256
8	1.194	0.311	0.330	0.289
9	1.496	0.283	0.306	0.317
10	1.707	0.270	0.290	0.332
11	2.174	0.246	0.260	0.359
12	2.457	0.233	0.244	0.367
13	3.167	0.197	0.213	0.408
14	4.166	0.148	0.183	0.436
15	5.162	0.133	0.158	0.456
16	5.190	0.135	0.158	0.458
17	6.168	0.112	0.141	0.474
18	7.165	0.105	0.126	0.488
19	8.164	0.096	0.114	0.499

Endo-Cycloaddition (KN040)

Nr.	t h	[2A] mol/L	[1a] mol/L	[3aA] mol/L
1	0.000	0.200	0.200	0.000
2	0.100	0.171	0.180	0.022
3	0.145	0.161	0.169	0.032
4	0.190	0.153	0.160	0.041
5	0.235	0.145	0.152	0.050
6	0.280	0.138	0.144	0.058
7	0.348	0.127	0.133	0.070
8	0.438	0.115	0.120	0.083
9	0.528	0.105	0.109	0.094
10	0.596	0.098	0.102	0.102
11	0.686	0.090	0.093	0.110
12	0.777	0.083	0.086	0.119
13	0.844	0.078	0.081	0.124
14	0.934	0.072	0.075	0.130
15	1.024	0.067	0.069	0.136
16	1.115	0.062	0.064	0.141
17	1.273	0.055	0.057	0.148
18	1.431	0.050	0.051	0.154
19	1.589	0.045	0.046	0.160
20	1.746	0.041	0.041	0.164
21	1.927	0.037	0.037	0.169
22	2.085	0.034	0.034	0.172
23	2.265	0.031	0.031	0.175
24	2.423	0.029	0.029	0.177
25	2.580	0.027	0.027	0.179
26	2.761	0.025	0.025	0.182
27	2.918	0.024	0.023	0.183
28	3.076	0.022	0.022	0.185
29	3.256	0.021	0.021	0.186
30	3.416	0.020	0.019	0.187
31	3.596	0.019	0.018	0.188
32	3.754	0.018	0.017	0.189
33	3.912	0.017	0.016	0.190
34	4.092	0.017	0.015	0.191

Endo-Cycloaddition (KN039)

Nr.	t h	[2A] mol/L	[1a] mol/L	[3aA] mol/L
1	0.000	0.200	0.200	0.000
2	0.100	0.162	0.169	0.034
3	0.145	0.149	0.156	0.048
4	0.190	0.138	0.144	0.060
5	0.235	0.128	0.133	0.072
6	0.280	0.119	0.123	0.081
7	0.348	0.107	0.111	0.095
8	0.438	0.094	0.097	0.109
9	0.528	0.082	0.085	0.121
10	0.596	0.075	0.078	0.129
11	0.687	0.067	0.069	0.138
12	0.777	0.061	0.062	0.145
13	0.845	0.057	0.058	0.149
14	0.935	0.051	0.052	0.155
15	1.025	0.047	0.048	0.159
16	1.093	0.044	0.045	0.163
17	1.273	0.038	0.038	0.170
18	1.431	0.034	0.034	0.174
19	1.612	0.030	0.029	0.179
20	1.770	0.027	0.026	0.182
21	1.929	0.024	0.024	0.184
22	2.111	0.022	0.021	0.187
23	2.269	0.020	0.020	0.189
24	2.427	0.019	0.018	0.190
25	2.609	0.018	0.017	0.192
26	2.699	0.017	0.016	0.193

Endo-Cycloaddition (KN041)

Nr.	t h	[2A] mol/L	[1a] mol/L	[3aA] mol/L
1	0.000	0.240	0.200	0.000
2	0.100	0.214	0.179	0.020
3	0.145	0.205	0.170	0.030
4	0.190	0.196	0.162	0.038
5	0.236	0.190	0.154	0.046
6	0.349	0.173	0.136	0.064
7	0.439	0.161	0.124	0.077
8	0.529	0.151	0.113	0.089
9	0.596	0.144	0.106	0.096
10	0.687	0.136	0.097	0.105
11	0.777	0.128	0.089	0.113
12	0.844	0.123	0.084	0.119
13	0.934	0.118	0.077	0.125
14	1.025	0.112	0.071	0.132
15	1.115	0.107	0.066	0.137
16	1.273	0.099	0.058	0.146
17	1.430	0.093	0.051	0.153
18	1.588	0.087	0.045	0.159
19	1.768	0.081	0.040	0.165
20	1.948	0.077	0.035	0.170
21	2.106	0.073	0.031	0.174
22	2.263	0.070	0.028	0.177
23	2.421	0.067	0.025	0.180
24	2.579	0.065	0.022	0.182
25	2.759	0.063	0.020	0.185
26	2.917	0.061	0.018	0.187
27	3.097	0.059	0.016	0.189
28	3.255	0.058	0.015	0.190

Endo-Cycloaddition (KN042)

Nr.	t h	[2A] mol/L	[1a] mol/L	[3aA] mol/L
1	0.000	0.200	0.257	0.000
2	0.100	0.169	0.231	0.027
3	0.145	0.158	0.219	0.038
4	0.190	0.149	0.209	0.049
5	0.235	0.139	0.199	0.059
6	0.280	0.131	0.190	0.068
7	0.349	0.119	0.178	0.082
8	0.439	0.106	0.163	0.096
9	0.529	0.094	0.151	0.109
10	0.596	0.086	0.142	0.118
11	0.686	0.077	0.132	0.129
12	0.776	0.068	0.124	0.137
13	0.844	0.063	0.118	0.144
14	0.935	0.057	0.111	0.151
15	1.025	0.051	0.105	0.157
16	1.115	0.045	0.099	0.162
17	1.273	0.038	0.091	0.171
18	1.430	0.032	0.085	0.178
19	1.610	0.026	0.079	0.184
20	1.768	0.022	0.074	0.188
21	1.881	0.020	0.072	0.191

Endo-Cycloaddition (KN043)

Nr.	t h	[2A] mol/L	[1a] mol/L	[3aA] mol/L
1	0.000	0.200	0.200	0.060
2	0.100	0.174	0.180	0.085
3	0.145	0.166	0.171	0.095
4	0.190	0.157	0.162	0.104
5	0.235	0.149	0.154	0.113
6	0.280	0.142	0.147	0.120
7	0.348	0.133	0.136	0.131
8	0.438	0.121	0.124	0.144
9	0.528	0.111	0.113	0.155
10	0.596	0.104	0.107	0.162
11	0.686	0.096	0.098	0.171
12	0.777	0.089	0.090	0.179
13	0.844	0.085	0.085	0.185
14	0.934	0.079	0.079	0.191
15	1.024	0.074	0.074	0.197
16	1.114	0.069	0.069	0.202
17	1.272	0.062	0.061	0.210
18	1.429	0.056	0.055	0.216
19	1.610	0.050	0.049	0.222
20	1.768	0.046	0.044	0.226
21	1.949	0.042	0.040	0.231
22	2.107	0.039	0.037	0.235
23	2.265	0.036	0.034	0.238
24	2.423	0.034	0.032	0.241
25	2.580	0.032	0.029	0.243
26	2.738	0.030	0.027	0.245
27	2.918	0.028	0.025	0.247
28	3.099	0.027	0.024	0.249
29	3.257	0.025	0.023	0.250
30	3.415	0.024	0.021	0.251
31	3.595	0.023	0.020	0.252
32	3.753	0.022	0.019	0.254
33	3.910	0.021	0.018	0.255
34	4.069	0.020	0.017	0.256

35	4.250	0.019	0.016	0.256
36	4.431	0.018	0.016	0.257

10 Spectroscopy

10.1 Co(II)-imidazolium-phenoxyimine complex C4

After complexation and activation, **C4*HCl** and **C4** were investigated by ^{1}H and ^{19}F-NMR spectroscopy.In Figure S20, the ^{1}H-NMR spectra of the imidazolium phenolimine preligand (Figure S20, green spectrum), the non-activated Co(II) imidazolium phenoxyimine complex **C4*HCl** (Figure S20, red spectrum), and the activated Co(II) imidazolium phenoxyimine complex **C4** (Figure S20, blue spectrum) are shown.

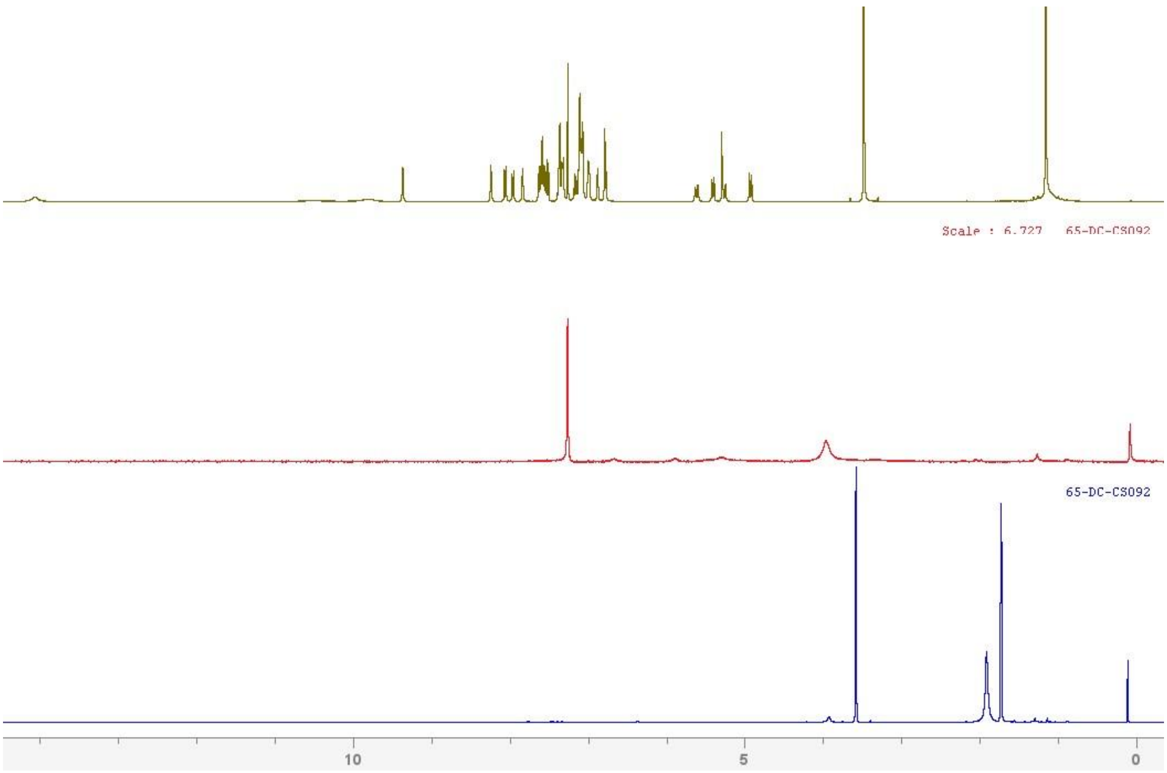

Figure S20: Comparison of the ^{1}H NMR spectra of the preligand (green spectrum) and the complexes C4*HCl (red spectrum) and C4 (blue spectrum).

For both **C4*HCl** and **C4**, no characteristic ligand signals can be observed, which suggests that paramagnetic complex species are formed after complexation and activation.

Due to the color change during complexation and activation from yellow [Ligand] to green [**C4*HCl**] and orange [**C4**] (Figure S21), we performed UV-Vis measurements. These were conducted at a concentration of $7 \cdot 10^{-5}$ M in a wavelength range of 200 to 700 nm. A comparison of the spectra of the preligand and the complexes **C4*HCl** and **C4** shows strong absorption bands in the range from 200 to approximately 320 nm. These can presumably be assigned to the aromatic units of the different species. However, significant differences are

evident in the wavelength range from approximately 320 to 480 nm, with a significant red shift being observed for the absorption bands from the ligand via **C4*HCl** to **C4**. Comparing these absorption band with the literature, we assigned this absorption to the π/π* transition of the imine.[29,30] The red shift could be explained by the coordination of the imine to the metal center.[29] In the spectrum of the activated complex **C4**, the strong shift of the absorption band from **C4*HCl** to **C4** could be explained by a change in the coordination environment of the Co(II) center upon activation of the complex.

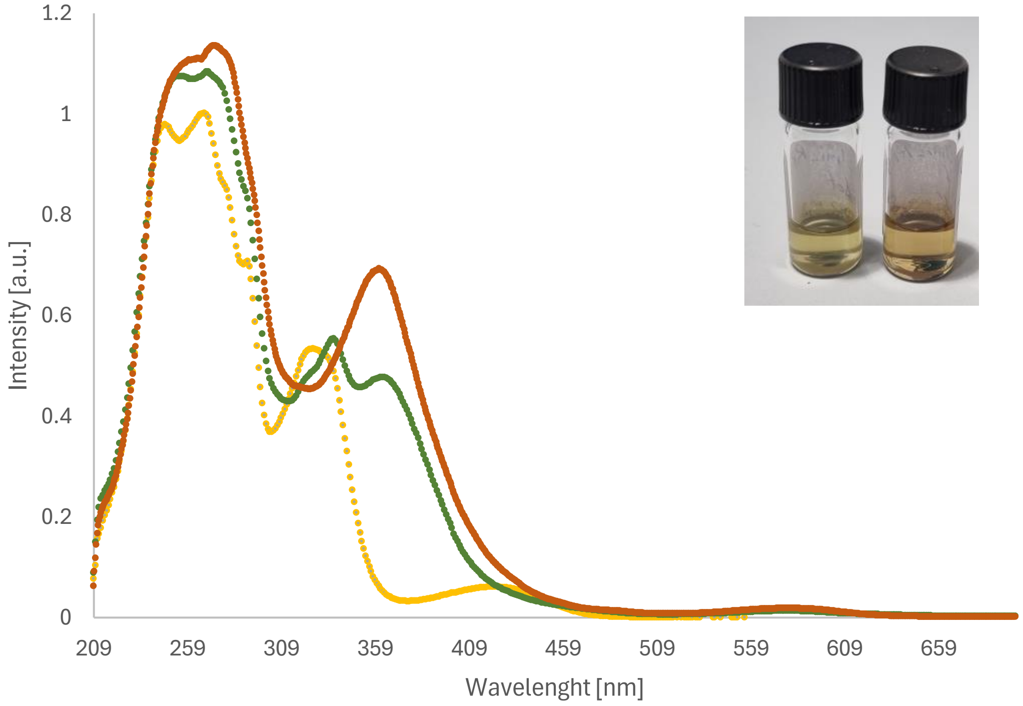

Figure S21: UV-Vis spectra of the imidazolium phenol imine preligand (yellow), the unactivated Co(II) imidazolium phenoxyimine complex C4*HCl (green), and the activated Co(II) imidazolium phenoxyimine complex C4 (orange). The solutions of the unactivated Co(II) imidazolium phenoxyimine complex C4*HCl (left) and the activated Co(II) imidazolium phenoxyimine complex C4 (right) are shown at the top right.

10.2 Ni(II)-triazolium-phenoxyimine complex C7

Figure S22 shows a comparison of the ^{1}H NMR spectra of the preligand (Figure S22, green spectrum), the unactivated Ni(II) triazolium phenoxyimine complex **C7*HPF$_6$** (Figure S22, red spectrum), and the activated Ni(II) triazolium phenoxyimine complex **C7** (Figure S22, blue spectrum). For the Ni(II) triazolium phenoxyimine complex, signals can be observed in the ^{1}H NMR spectrum for both **C7*HPF$_6$** and **C7**, which are broadened and shifted compared to the preligand. For both the non-activated and activated Ni-triazolium phenoxyimine complexes the phenolic proton characteristic of the preligand disappears at a shift of 14.2 ppm, suggesting that the observed signals could be assigned to the Ni(II)-triazolium phenoxyimine complex. Due to the strong signal broadening, further interpretation of the spectra is not possible.

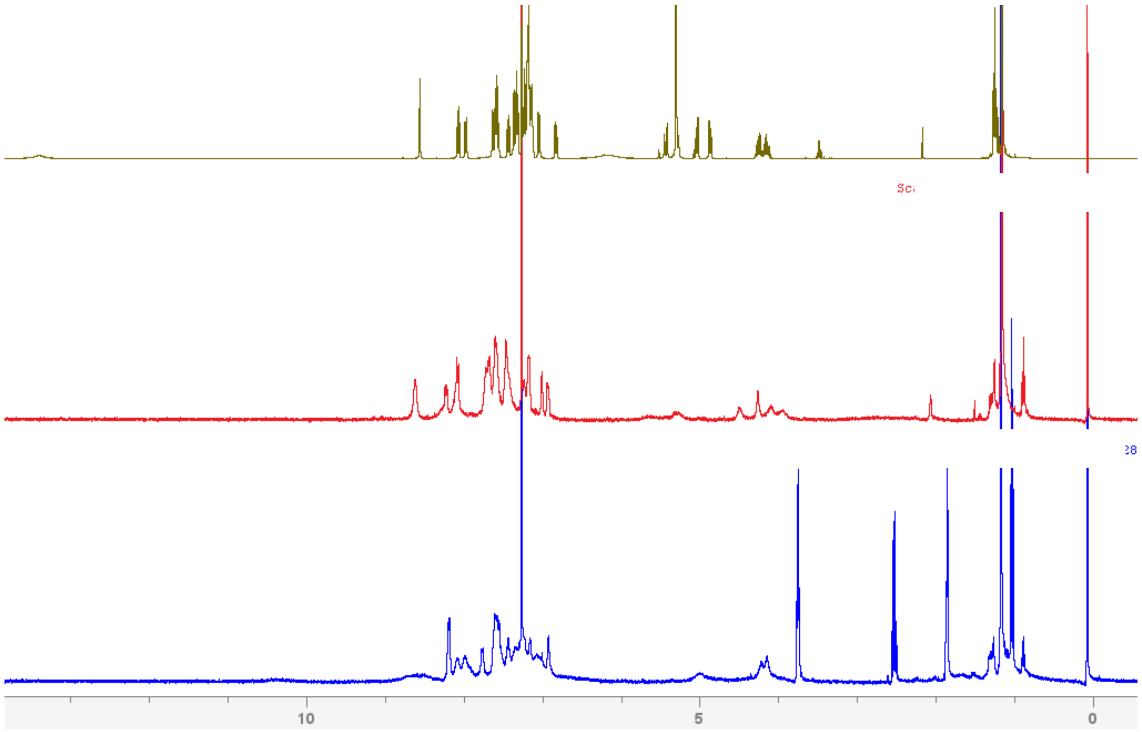

Figure S22: Comparison of the ^{1}H NMR spectra of the triazolium phenol imine preligand (green spectrum), the unactivated Ni(II) triazolium phenoxyimine complex C7*HPF$_6$ (red spectrum) and the activated Ni(II) triazolium phenoxyimine complex C7 (blue spectrum).

It can be concluded from the spectra that both the **C7*HPF$_6$** and **C7** samples contain both diamagnetic and paramagnetic components.

A significant color change can be observed from yellow ligand to orange precatalyst **C7*HPF$_6$** to golden-brown catalyst **C7**. UV-Vis measurements were performed at a concentration of $7 \cdot 10^{-5}$ M in a wavelength range of 200 to 700 nm (Figure S23). The species in Figure S23 show strong absorption bands in the range from 200 to approximately 320 nm, that can presumably be assigned to the aromatic units of the various species. Significant differences between the species can be seen in the wavelength range from approximately 307 to 560 nm, with a significant red shift in the absorption bands of the phenolimine preligand, via the unactivated complex **C7*HPF$_6$**, to the activated complex **C7**. The bands can presumably be assigned to the π/π* transition of the imine.[29,30] In the case of the Ni-triazolium complex, the strong shift of the absorption band of **C7*HPF$_6$** compared to **C7** is also striking. An explanation for this could be a change in the coordination environment of the Ni(II) center upon activation of the complex.

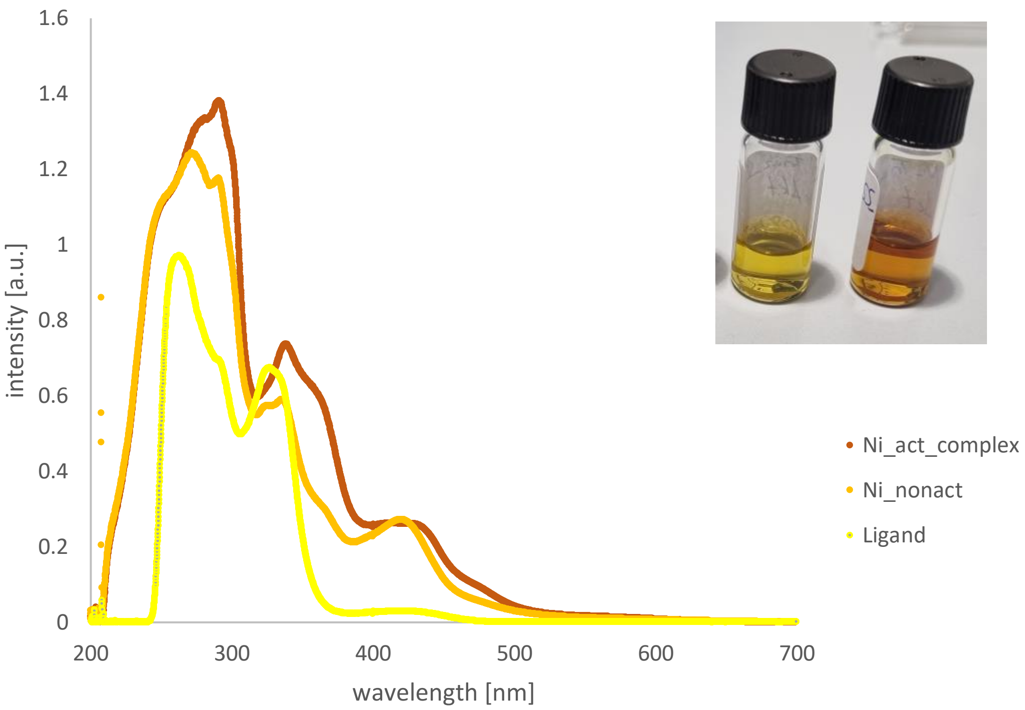

Figure S23: UV-Vis spectra of the triazolium phenol imine preligand (light yellow), the non-activated Ni(II) triazolium phenoxyimine complex C7*HPF$_6$ (yellow-orange) and the activated Ni(II) triazolium phenoxyimine complex C7 (brown). The solutions of the non-activated Ni(II) triazolium phenoxyimine complex C7*HPF$_6$ (left) and the activated Ni(II) triazolium phenoxyimine complex C7 (right) are also shown at the top right.

11 Magnetometry

SQUID measurements were conducted to get insight into the overall electronic spin in the sample as well as magnetic characteristics of the compounds. The measurements were taken in solid state powders immobilized in eicosane.

The Ni(II) centred catalyst is a d^8-system and does not change oxidation state by activation to **C7.** The overall spin of the molecule is dependent on the ligand field splitting and can either be S=0 or S=1. Susceptibility measurements reveal a low magnetic moment in both samples (Figure S24).

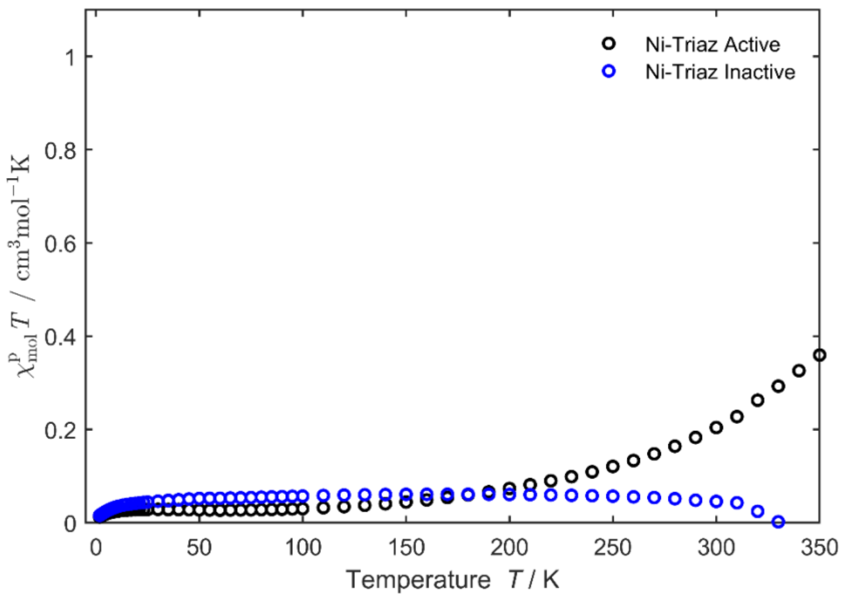

Figure S24: χT plot of magnetic susceptibility measurements on the Ni(II)Triaz catalyst **C7*HPF$_6$** and **C7**. The measurement was taken on a solid state sample in a temperature range between 4-350 K.

Below 200 K the susceptibility-temperature product $\chi_m T$ in both samples is around 0.07 cm^{3}mol^{-1}K. With increasing temperatures, the susceptibility of the inactive catalyst remains constant and drops at elevated temperatures to 0 cm^{3}mol^{-1}K, whereas the susceptibility of the active catalyst rises to 0.37 cm^{3}mol^{-1}K at 350 K. The expected susceptibility of an S = 0 system is 0 cm^{3}mol^{-1}K and ca. 1 cm^{3}mol^{-1}K for an S = 1 system with g = 2, according to the Curie-Weiss law.

The results indicate a mostly diamagnetic species in both samples in solid state with changes in the summed magnetic moment upon activation of the catalyst. The small but significant increase of $\chi_m T$ towards high temperatures for the active is consistent with small structural changes from diamagnetic square planar to paramagnetic distorted tetrahedral, or interaction with an apical group.

The Co(II) centered catalyst is a d^7-system and does not change oxidation state upon activation to **C4**. The overall spin of the molecule is dependent on the ligand field splitting and can either be S=3/2 or S=1/2. Similar to the Nickel system SQUID measurements of the solid

samples immobilized in eicosane were done on both the **C4*HCl** (Figure S25) and **C4** (Figure S26) compound.

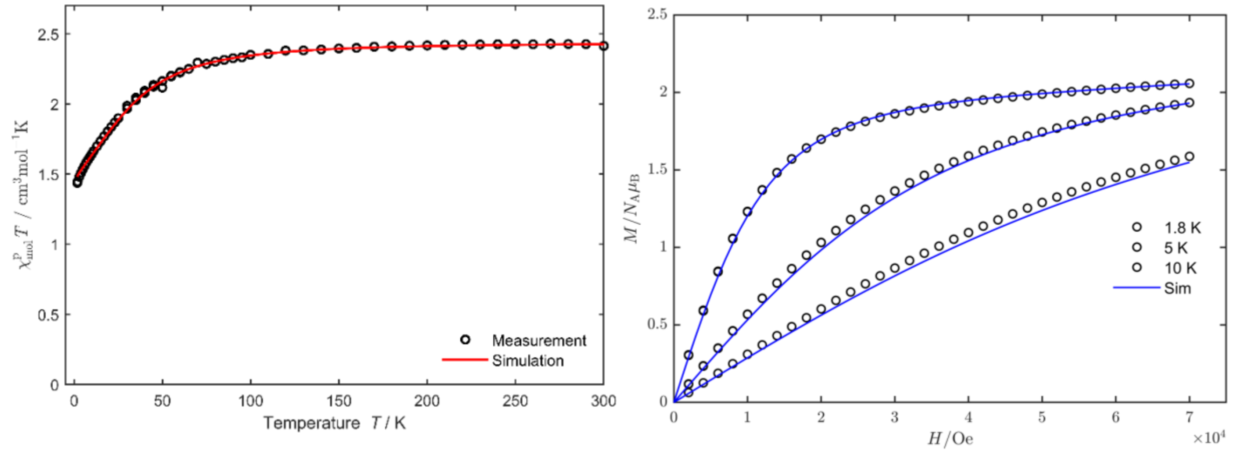

Figure S25: (left) χT plot of magnetic susceptibility measurements in a temperature range between 4-300 K; (right) *MH* measurement between 0-7 T on the **non-activated** Co(II)Imidazole catalyst **C4*HCl**. The measurement was taken on a solid-state sample.

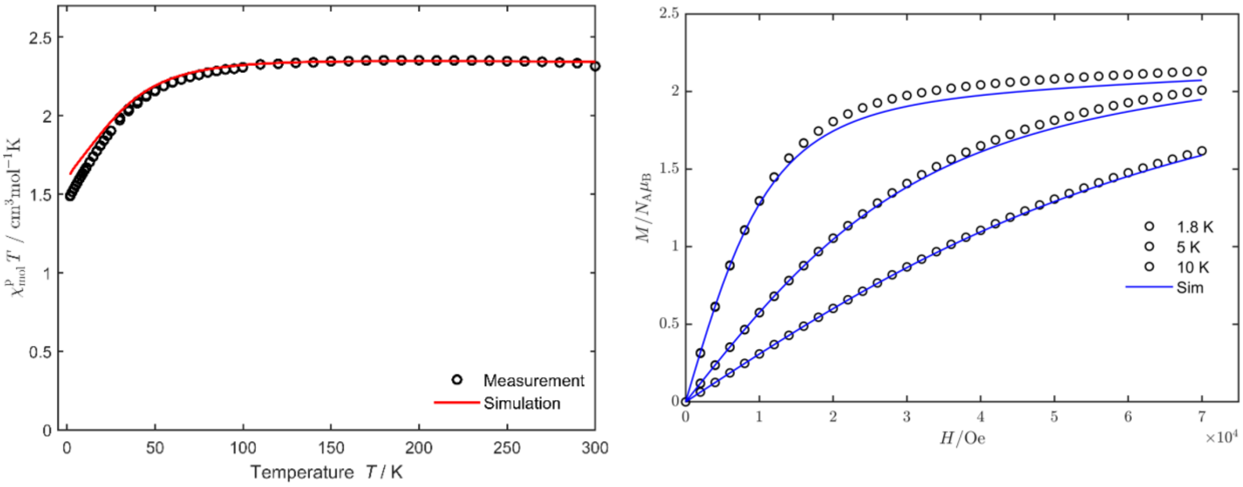

Figure S26: (left) χT plot of magnetic susceptibility measurements in a temperature range between 4-300 K; (right) *MH* measurement between 0-7 T on the **activated** Co(II)Imidazole catalyst **C4**. The measurement was taken on a solid-state sample.

In both measurements the susceptibility at high temperatures is constant at around 2.4 $cm^3mol^{-1}K$. Below 100 K the intensity of $\chi_m T$ drops to 1.5 $cm^3mol^{-1}K$ at 1.8 K in both samples. The general shape of *MH* measurements is similar in both samples and shows a saturation effect at 1.8 K but is almost linear at 10 K.

The shape of both measurements are characteristic for high spin (S=3/2) Co(II) compounds with a positive zero field splitting (ZFS). The measurements were fitted by a calculation of the spin Hamiltonian to represent the measurements.[31] The parameters of the calculations for both compounds are represented in the following table:

Table S3: Simulation Parameters for SQUID measurements of C4 and the precatalyst. Measurements and Simulation are shown in **Figure S25** and **Figure S26**.

	Co(II) non-activated	Co(II) activated **C4**
S	3/2	3/2
g_{iso}	2.28(3)	2.21(6)
D [cm^{-1}]	35(2)	38(1)
E/D	0.28(2)	0.33(1)

The results for **C4** are backed by CASSCF NEFPT2 calculations on DFT optimized structure of the active Co(II) triazole catalyst that predict a ZFS of 37.837 cm^{-1} and an E/*D* ratio of 0.309.

A second perspective on the susceptibility of each system is obtained by Evans method NMR measurements.[32] In contrast to SQUID measurements the sample is in solution and only one measurement point at room temperature is obtained. Measurements were taken on all previously mentioned samples in THF, a coordinating solvent that was also used for catalysis. For the measurement the sample is dissolved in deuterated solvent with a small impurity of non-deuterated solvent. In addition to that a capillary with a 10:1 mixture of deuterated and not deuterated solvent is added in the NMR tube. In a standard ^{1}H NMR measurement the signals of the non-deuterated solvents are tracked. The presence of the paramagnetic species in the NMR tube shifts the signals. The shift between the signals is linked to the susceptibility by the following equation:

$$\chi_{\mathrm{m}}^{p} = \frac{3\,\delta\nu\, c^{p}}{\nu_0 4\pi} - \chi_{m}^{dia}$$

In the equation $\delta\nu$ is the observed frequency shift, c^p is the concentration of the paramagnetic species in the NMR tube, ν_0 is the frequency of the NMR spectrometer and χ_m^{dia} is the diamagnetic susceptibility of the compound. The latter can be estimated accurately by the Pascal's constants.[33]

NMR Measurements on the Cobalt system were done in THF with 2 mM solutions of the non-activated and the activated catalyst. The diamagnetic corrections were done with Pascal's constants based on the compounds structure. The results of the measurements are shown in the following table.

Table S4: Measurement conditions and results of Evans method NMR measurements on the non-activated and the activated catalyst in THF-D_8. The diamagnetic correction is calculated with Pascal's constants.

	Co(II) non-activated	Co(II) activated **C4**
Frequency ν_0 [MHz]	400.100	400.100
Temperature *T* [K]	298	298

Splitting $\delta\nu$ [Hz]	20.80	21.07
Diamagnetic Correction χ_m^{dia} [emu mol^{-1}]	-3.86e-4	-3.63e-4

The splitting can be interpreted with a S = 3/2 with slightly shifted g-value between the two samples. The inactive catalyst has g_{iso}=2.23(3) and the active catalyst has g_{iso}=2.28(3).

The results of Evans NMR experiments are close to results obtained by SQUID magnetometry in solid state. Slight shifts in the g-value could be because a solid-state structure is compared to a solvated molecule. This could be geometric change or even coordination of THF to the Co(II) center.

In all measurements on the Nickel catalyst **C7** the splitting of the solvent peaks is too small to separate signals. In addition to the little portion of paramagnetic species the split of a S = 1 species is much weaker than a S = 3/2 based splitting. Based on the line broadening of the NMR signals, a paramagnetic contribution in a similar range as obtained by SQUID can also be present in the NMR data.

12 EPR-Experiments

EPR spectroscopy gives perspective on the paramagnetic center in the complexes by probing transitions between energy levels in oppose to detecting the bulk magnetic moment. By that, we aimed for a more detailed view on electronic structure and changes on the metal center by activation of the catalysts and solvation in different solvents.

Magnetometry measurements on **C7** and its precatalyst (featuring the neutral naphthol unit) showed that the compounds are mostly diamagnetic. A paramagnetic Ni(II) d^8-system is S=1 which can only be detected in high field EPR (HFEPR) in most cases, because of the integer spin and zero field splitting (ZFS). Solid state measurements at 4 K in HFEPR did not give a signal.

C4 and its precatalyst were measured in X-band EPR in frozen solutions of toluene:DCM and 2-Me-THF. The measurements were done at 4 K and 30 K.

The general shape of the signal is similar in all samples, with a signal at 1000 G with a splitting into 6 lines and further signals at 3000 G and 4500 G. The central signal shifts strongly between the solvents. Overall, all signals are very broad and not well resolved. With increasing temperature all signals become less intense.

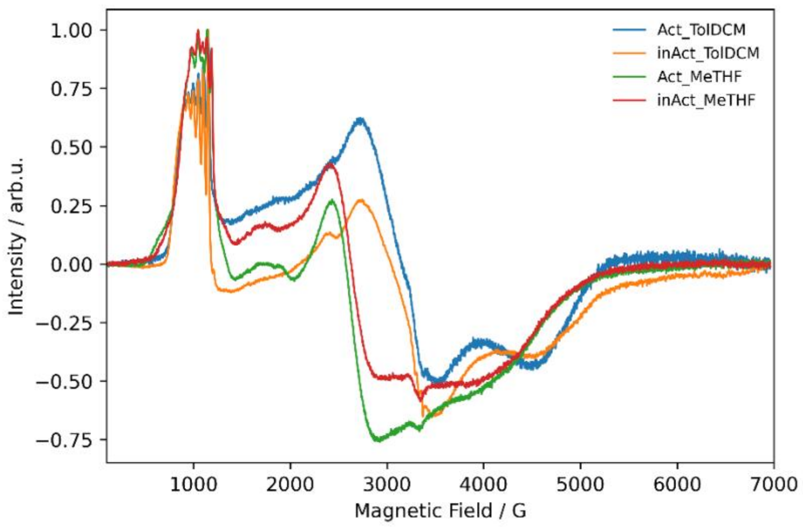

Figure S27: X-Band EPR measurements of the non-activated and activated Cobalt complex **C4*HCl** and **C4** measured at 7 K in 1 mM solutions of 2-MeTHF and a mixture of Toluene:DCM (1:1)

The splitting at low field can be assigned to hyperfine splitting based on the cobalt center and the strong shift in signals based on the solvent indicates towards coordination of the solvent to open coordination sites of the catalyst. Because of the broad signals it is hard to clarify whether signals of multiple species overlap in the spectrum. The decreasing signal intensity at higher temperatures confirm the positive sign of the ZFS.

The spectrum of the active catalyst in Toluene DCM was simulated with easyspin based on starting parameters of calculations and magnetometry.[31]

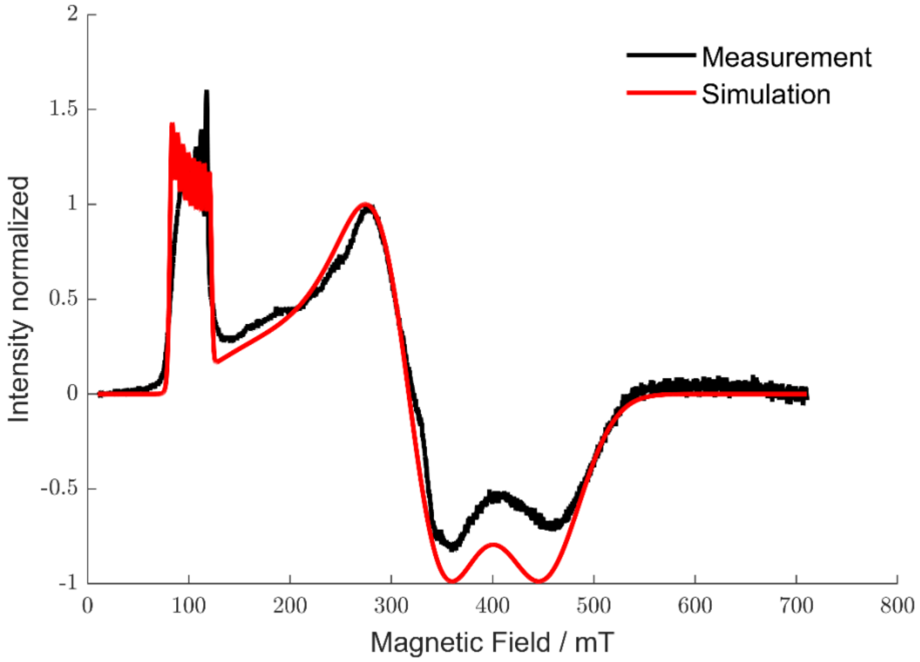

Figure S28 X-Band EPR Measurement of C4 as a 1 mM solution in Toluene:DCM (black) with a simulation according to the Spin Hamiltonian and parameters in Table S5 done with easyspin.

The simulation is shown in Figure S28 and based on the parameters in the following table.

Table S5: Simulation parameters for X-Band measurement on **C4** in frozen solution (toluene:DCM) shown in **Figure S28**.

Parameter	Value
Spin *S*	3/2
g_x; g_y; g_z	2.15(1); 2.45(1); 2.05(1)
g_{iso}	2.22(1)
Hyperfine Co A_x; A_y; A_z	0; 180(5);0
D	38(1)
E/D	0.33(1)
*g*Strain	0.52(2); 0.10(2); 0.37(2)

The large ZFS in all Co(II) based samples cannot be probed directly with sub-THz EPR techniques because the energy gap between the $m_S = \pm {}^1\!/_2$ and $m_S = \pm {}^3\!/_2$ states is too large to be probed. Because of that, EPR is relatively insensitive to the ZFS in this specific case.

13 Investigation of a Possible Non-Linear Effect

The presence of a nonlinear effect in an asymmetric catalytic reaction can serve as evidence for the involvement of dimers in the catalytic cycle. If a monomeric catalyst species is involved in the selectivity-determining step, a linear dependence of the *ee*-value of the product ($ee_{Prod.}$) and the *ee*-value of the catalyst ($ee_{Cat.}$) is expected due to the formula:[34,35]

$$ee_{Prod.} = ee_{max} ee_{Cat.} \qquad (2)$$

Deviations from this behavior can be indicative for the presence of catalyst dimers.[34,35] The following figures show the results of the investigation for the Ni(II) triazolium phenoxyimine complex **C7** and the Co(II) imidazolium phenoxyimine complex **C4**. For the experiments, the two catalyst enantiomers **C4**/ ***ent*-C4** and **C7**/ ***ent*-C7** were mixed to obtain catalysts with different *ee*-values of 0, 20, 40, 60, 80 and 100% ee.

For the *endo*-selective cycloaddition the experiments are conducted at a concentration of 0.2 M (Table S6).

Table S6: 1,3-Dipolar cycloaddition of azomethine ylides to maleimides. Investigations of the nonlinear effect in the endo-selective model reaction.

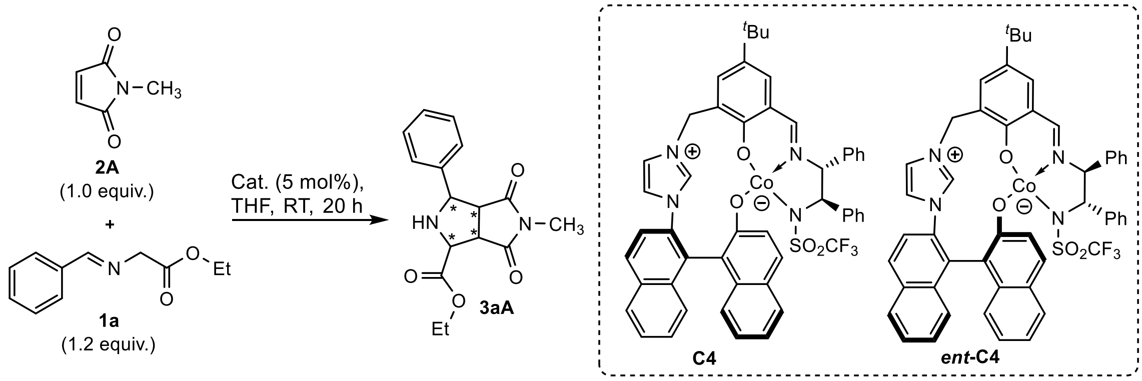

Nr.	*ee* C4 [%]	*dr*[a] (*exo*/*endo*)	*ee*[b] (*endo*)
1	0	6:94	3
2	20	6:94	21
3	40	7:93	39
4	60	6:94	55
5	80	6:94	80
6	100	6:94	98

[a]Determined by H-NMR spectroscopy.

[b] Determined by HPLC on a chiral stationary phase.

For evaluation, the *ee*-value of the *endo*-configured product **3aA** is compared with the ee value of the catalyst in Figure S29.

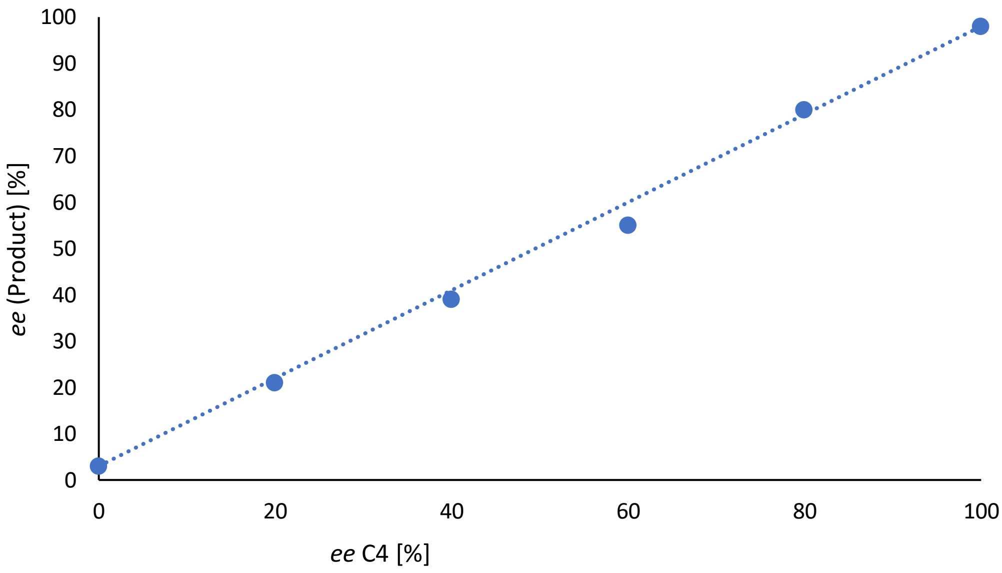

Figure S29: Evaluation of the study on the non-linear effect for the endo-selective model reaction.

No deviation from a linear dependence could be obeserved for the *endo*-selective cycloaddition using the activated Co(II) imidazolium phenoxyimine complex **C4/ *ent*-C4**.

For the *endo*-selective cycloaddition the experiments are conducted at a concentration of 0.5 M (Table S7).

Table S7: 1,3-Dipolar cycloaddition of azomethine ylides to maleimides. Investigations of the nonlinear effect in the exo-selective model reaction.

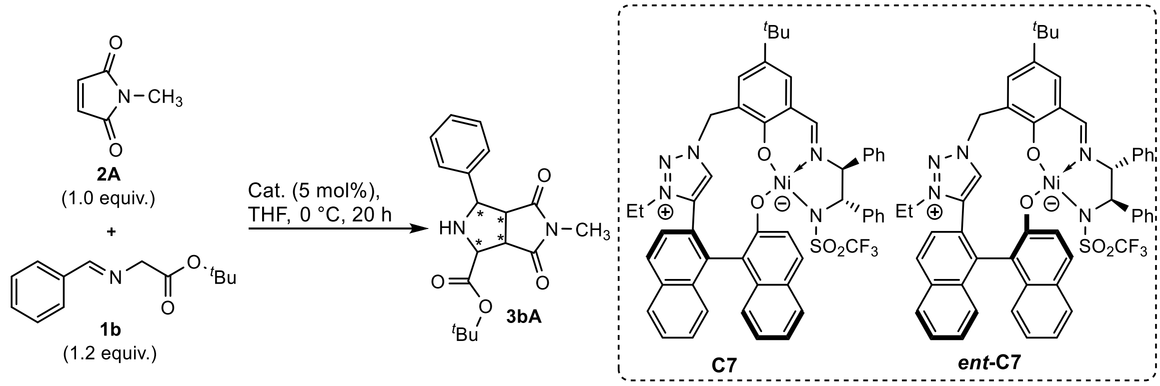

Nr.	*ee ent*-C7 [%]	*dr*[a] (*exo*/*endo*)	*ee*[b] (*exo*)
1	0	91:9	2
2	20	91:9	17
3	40	91:9	39
4	60	90:10	55
5	80	91:9	66
6	100	91:9	91

[a]Bestimmt mittels H-NMR Spektroskopie.

[b]Bestimmt mittels HPLC an chiraler stationärer Phase.

For evaluation, the ee-value of the exo-configured product **3bA** is compared with the ee value of the catalyst in Figure S30.

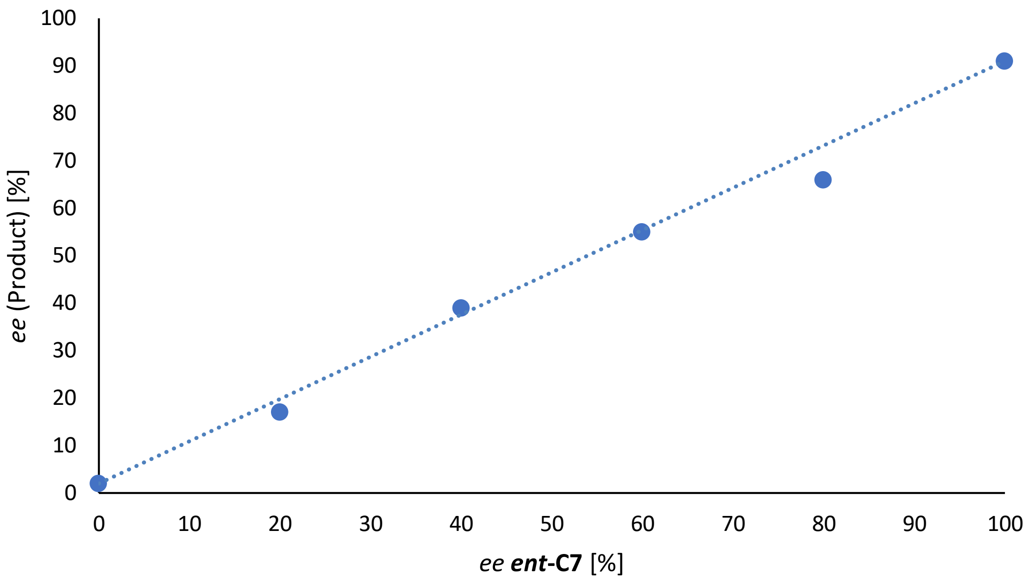

Figure S30: Evaluation of the study on the non-linear effect for the exo-selective model reaction.

No deviation from a linear dependence could be obeserved for the *exo*-selective cycloaddition using the activated Ni(II) triazolium phenoxyimine complex ***ent*-C4/C4**.

14 UV-Vis Experiments

14.1 *Beer*'s Plot

As a support for the investigations in chapter 13 (non-linear effect study), UV-Vis experiments were performed for **C4** and **C7**. In the experiments, the dependence of the absorption intensity of characteristic absorption bands on the concentration of the complexes was investigated. Deviations from the *Lambert-Beer* law in the form of a non-linear relationship between the concentration of the analyte and the absorption intensity and the can occur by aggregation, e.g. through the formation of dimers.[36]

In the experiments, the UV-Vis spectra of **C4** and **C7** were measured in concentration series (200 to 700 nm). For **C4**, a concentration range of 7.0 · 10-5 M to 1.0 · 10-7 M was prepared in dry THF (Figure S31).

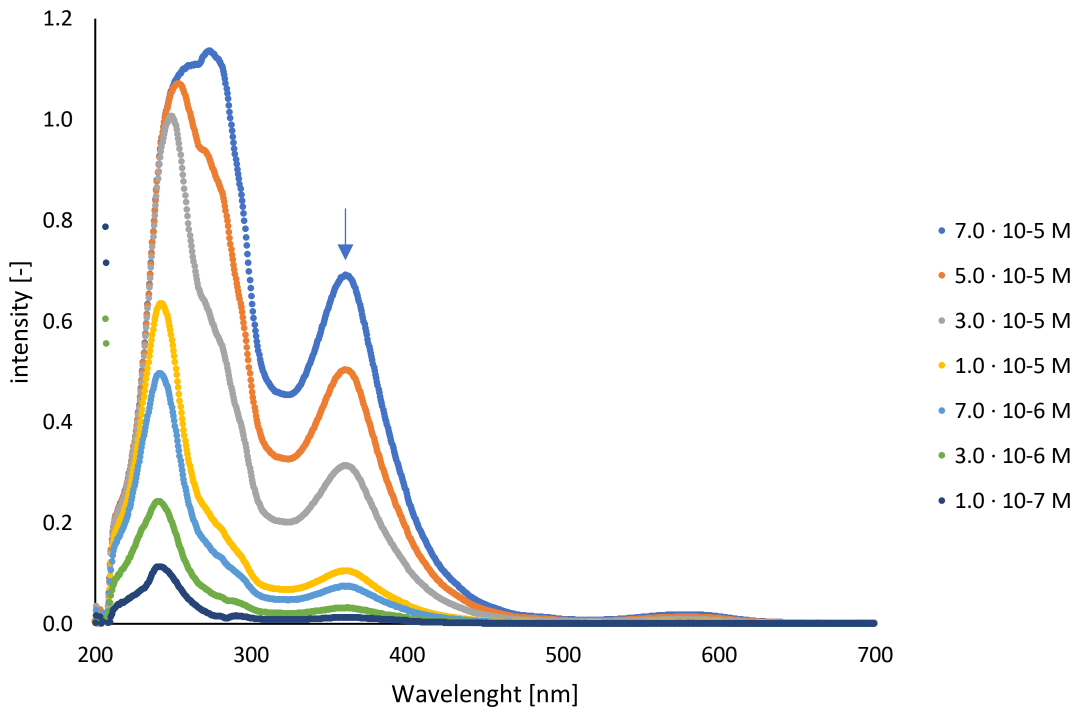

Figure S31: The UV-Vis spectra of the concentration series of C4 in dry THF in a concentration range from 7.0 · 10^{-5} M bis 1.0 · 10^{-7} M.

For the analysis, the absorption maximum at a wavelength of 361 nm was used. This absorption band can presumably be attributed to the π/π* transition of the imine.[29,30] Table S8 lists the intensity of this absorption band for the concentration series of **C4**.

Table S8: The intensity of the absorption band at a wavelength of 361 nm for the concentration series of C4.

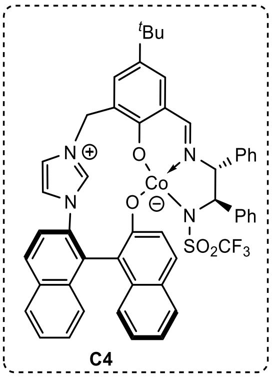

c [M]	*I* [-] 361 nm
$7.0 \cdot 10^{-5}$	0.692
$5.0 \cdot 10^{-5}$	0.503
$3.0 \cdot 10^{-5}$	0.314
$1.0 \cdot 10^{-5}$	0.105
$7.0 \cdot 10^{-6}$	0.074
$3.0 \cdot 10^{-6}$	0.031
$1.0 \cdot 10^{-7}$	0.012

Figure S32 depicts the intensity of the absorption maximum at a wavelength of 361 nm plotted against the concentration of the measurement series for **C4**.

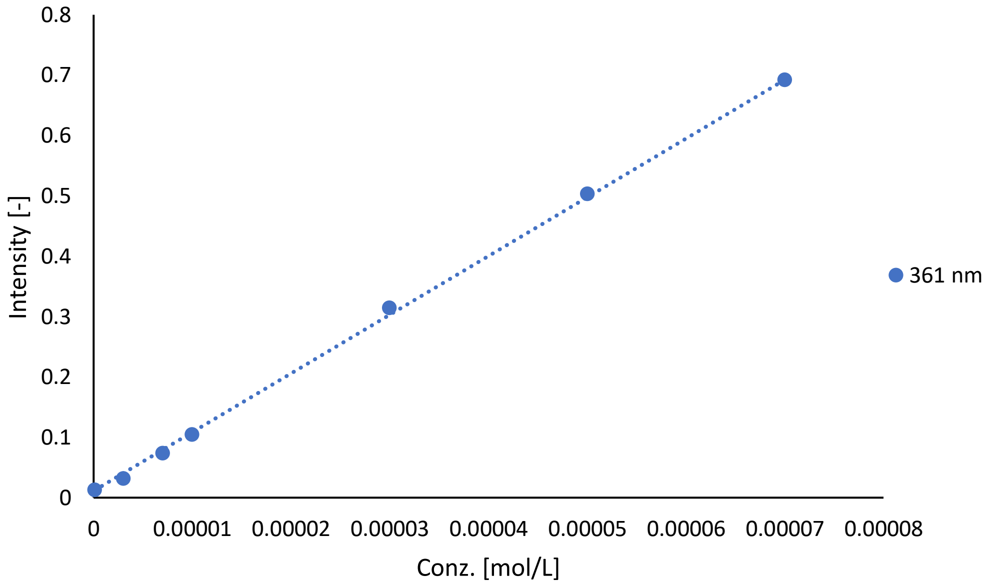

Figure S32: Plot of the intensity of the absorption band at 361 nm versus the concentration of C4.

No deviation from the *Lambert-Beer* law was found. This result was interpreted as further evidence against the presence of a catalyst dimer for the Co(II)-catalyst **C4**.

For the Ni(II)-complex **C7**, a concentration series with a range from 7.0 · 10-5 M to 1.0 · 10-7 M was prepared in dry THF (Figure S33).

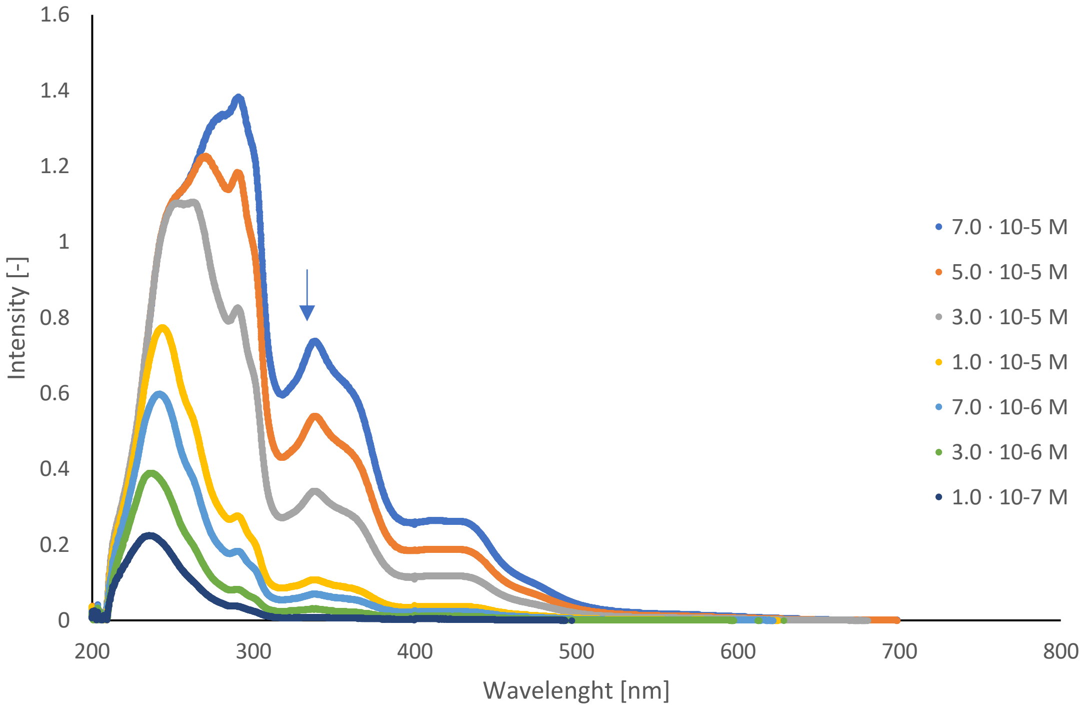

Figure S33: The UV-Vis spectra of the concentration series of C7 in dry THF in a concentration range from 7.0 · 10^{-5} M bis 1.0 · 10^{-7} M.

For the analysis, the absorption maximum at a wavelength of 339 nm was used. This absorption band can presumably be attributed to the π/π* transition of the imine.[29,30] **Table S9** lists the intensity of this absorption band for the concentration series of **C7**.

Table S9: The intensity of the absorption band at a wavelength of 339 nm for the concentration series of (R)-(1S,2S)-Ni(II)-Tri-Tf-213*.

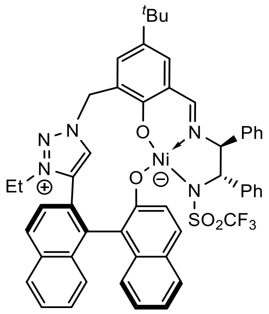

C7

c [M]	*I* [-] 339 nm

$7.0 \cdot 10^{-5}$	0.738
$5.0 \cdot 10^{-5}$	0.538
$3.0 \cdot 10^{-5}$	0.342
$1.0 \cdot 10^{-5}$	0.107
$7.0 \cdot 10^{-6}$	0.069
$3.0 \cdot 10^{-6}$	0.030
$1.0 \cdot 10^{-7}$	0.008

Figure S34 depicts the intensity of the absorption maximum at a wavelength of 339 nm plotted against the concentration of the measurement series for **C7**.

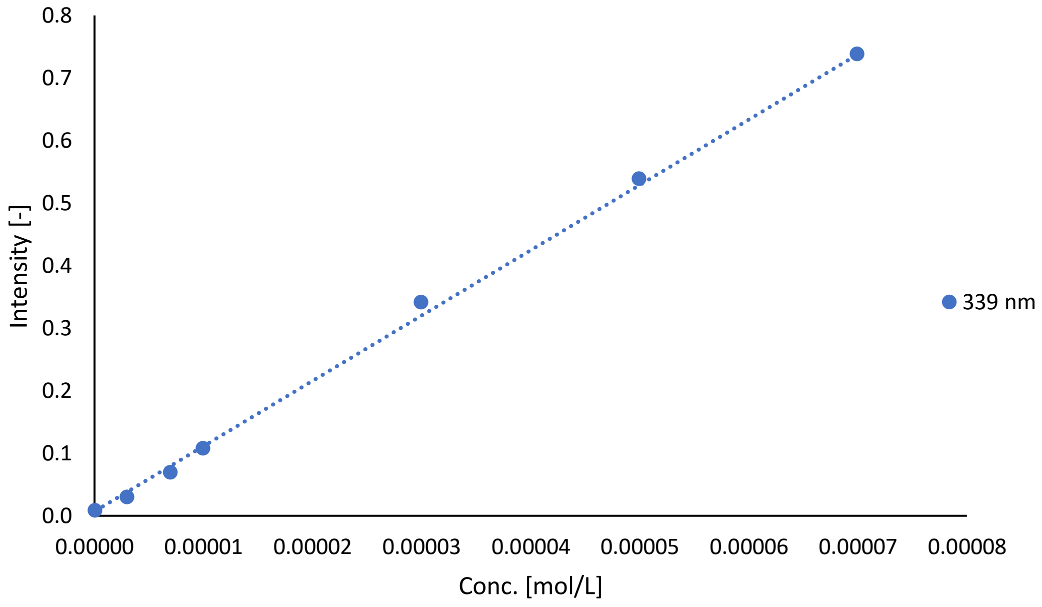

Figure S34: Plot of the intensity of the absorption band at 339 nm versus the concentration of C7.

No deviation from the *Lambert-Beer* law was found. This result was interpreted as further evidence against the presence of a catalyst dimer for the Ni(II)-catalyst **C7**.

15 Computational studies

15.1 General

The conformational space of all molecules has been initially searched using meta-dynamics simulations based on tight-binding quantum chemical calculations as implemented in CREST.[37][38]

Stationary point on the potential energy surface (PES) were optimized with the DL-FIND optimization library[39] in ChemShell.[40] All minima and transition state structures were

preoptimized using GFN2-xTB.[41] The program package Turbomole V7.4.1[42] was used for the electronic structure calculations on density functional theory (DFT) level of theory. Geometry optimization and subsequent frequency calculations were performed with DFT, PBEh-3c functional, and the def2-mSVP basis set.[43] The optimized minima structures were verified to be local minima by analytical frequency analysis. All transition state structures were confirmed to possess only a single mode with an imaginary frequency. To verify reactant and product states, internal reaction coordinate (IRC) calculations starting from the transition state structures were performed. The Gibbs free energy G was calculated at 298.15K within the rigid rotor harmonic oscillator (RRHO) approximation at a reference state of 1 atm in gas phase. Vibrational frequencies less than 100cm^{-1} were raised to that threshold.

At fixed geometries, the electronic energy was calculated using the B3LYP functional[44] with Grimme´s empirical dispersion correction with Becke-Johnson damping scheme D3(BJ).[45] For all single point calculations, the def2-TZVP basis set[46] was used and the solvent effects were accounted for with the conductor-like screening model (COSMO).[47–49] A dielectric constant of ϵ = 7.53 for THF and default values for the atomic radii in the cavity for COSMO were used.

Gibbs free energy differences for bimolecular reactions were converted from 1 atm gas phase reference state $\Delta G°_{1\ \mathrm{atm}}$ to 1 mol/L liquid reference state $\Delta G°_{1\ \mathrm{mol/L}}$ using the following equation:

$$\Delta G^{\circ}_{1\,\mathrm{mol/L}} = \Delta G^{\circ}_{1\,\mathrm{atm}} - RT\log\left(\frac{RT}{p_0}\,c_0\right)$$

where R is the ideal gas constant, T the absolute temperature in Kelvin, p_0 the reference pressure of 1 atm and c_0 = 1 mol/L.

An additional xyz-file has been provided, which contains cartesian coordinates for all calculated species.

15.2 Generating structure for C4 and C7

In two previous studies, structurally related Cu^{II} complexes were investigated as catalysts for the asymmetric 1,4-addition of various 1,3-dicarbonyl substrates to β-substituted nitroolefins and the addition of nitroolefins to pyrazolones, respectively, where the reaction mechanism was also elucidated by DFT calculations (B3LYP/cc-pVDZ/IEF-PCM (THF) using the program package Gaussian 16[50] or analogously to the methodology described in 1. In this context, a structure of the catalyst was created in the publication of Willig and Peters[2] based on the crystal structure of a related complex (for details see the work of *Willig and Peters*). This calculated structure served as the starting point for our earlier study,[4] as we replaced the imidazolium group with a triazolium unit.

In the present work, starting structures for **C4** and **C7** were built based on the two catalyst structures described above by changing the central metal ion and possibly varying the stereoinformation of the chiral backbone.

15.3 The *exo*-selective approach with C7

15.3.1 Precatalyst

15.3.1.1 Spin-State

According to solid-state EPR spectroscopy and SQUID magnetometry (chapter 11and 12), the precatalyst corresponding to **C7** (called **pC7**) consists of a large fraction of a low-spin state complex and a small fraction of a high-spin complex.

In Table S10, the relative Gibbs free energies of naked **pC7** are listed.

Table S10: Summary of relative Gibbs free energies of different spin-states for **pC7**.

spin state	Δ*G*°/kJ mol^{-1} relatively to triplet	
singlet	+34.4	
triplet	0.0	This compound is going to be called **pC7**.

15.3.1.2 Adducts

Since THF is a coordinating solvent, the potential adduct formation between **pC7** and THF must be taken into account. It turns out that increasing the coordination number of the Ni metal center from 4 to 5 for the low-spin complex leads to a significant destabilization. This is not surprising as for Ni(II) Coordination-Induced Spin-State Switching (CISSS) is a known effect.[51]

Since NEt_3 is used for activation, adduct formation with it is also investigated. The results are summarized below (Table S11).

Table S11: Summary of relative Gibbs free energies of possible adducts of **pC7** with THF and NEt_3 in different spin-states.

adduct partner	Spin-state	Δ*G*°/kJ mol^{-1} relatively to **pC7**
THF	singlet	+78.7
	triplet	+0.7
NEt_3	singlet	+29.8
	triplet	+16.2

15.3.2 Activated Catalyst

15.3.2.1 Spin-State

According to solid-state EPR spectroscopy and SQUID magnetometry (chapter 11and 12), the catalyst **C7** consists of a large fraction of a low-spin state complex and a small fraction of a high-spin complex.

In Table S12, the relative Gibbs free energies of naked **C7** are listed.

Table S12: Summary of relative Gibbs free energies of different spin-states for **C7**.

spin state	$\Delta G°$/kJ mol^{-1} relatively to triplet	
singlet	-2.7	
triplet	0.0	This compound is going to be called **C7**.

15.3.2.2 Adducts

Again, adduct formation with THF and NEt_3 is taken into account. It turned out, that in contrast to **pC7**, the THF adduct formation is exergonic for the high-spin catalyst (Table S13).

Table S13: Summary of relative Gibbs free energies of possible adducts of **C7** with THF and NEt_3 in different spin-states.

adduct partner	spin state	$\Delta G°$/kJ mol^{-1} relatively to **C7**
THF	singlet	+8.4
	triplet	-13.9
NEt_3	singlet	+27.3
	triplet	+57.4

15.3.3 Singlet vs. Triplet PES

As can be seen from the tables above in combination with the EPR spectroscopic and SQUID magnetometric data, it is not clear on which PES the reaction takes place. Furthermore, it is possible that a change of multiplicity occurs during the course of the reaction.

However, it is known that DFT has problems in predicting accurate energy differences for different multiplicities. Therefore, the singlet and triplet PES might be shifted relative to each other.

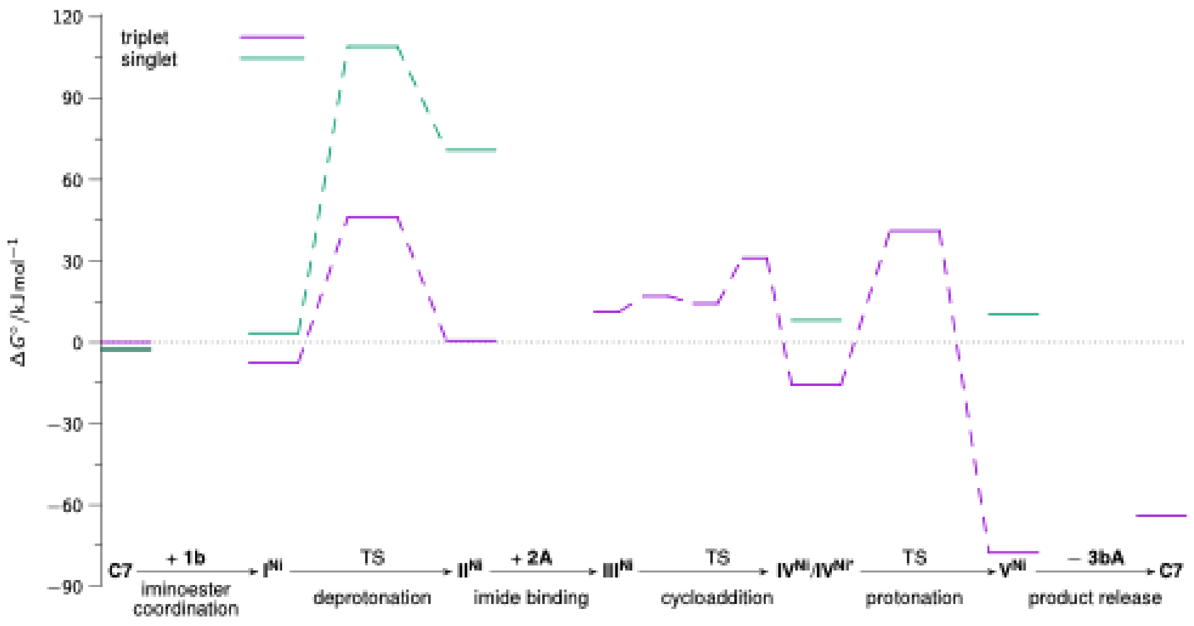

Figure S35: Gibbs free energy profile of the proposed catalytic cycle (Scheme 2 in the main text). **C7** (triplet) and the isolated substrates were chosen as reference state ($\Delta G°$ = 0 kJ mol^{-1}).

To systematically answer these two questions, almost all intermediates for the *exo*-main product were calculated on both the singlet and triplet PES.

This clearly shows that changing the coordination number from 4 to 5 for Ni(II) in the low-spin state leads to a significant increase in energy. This finding is not unexpected (see above).

The energy difference between the different intermediates on the singlet PES is so large that a reaction can be ruled out even without barriers on the singlet PES.

To further support this, the barrier to iminoester deprotonation on both PESs was also investigated. The large barrier on the singlet PES further confirms the finding that the high-spin portion is likely to be catalytically active.

15.3.4 C,C Bond Formation

The C,C bond formation step is particularly interesting for *exo*-selective catalysis with **C7**: For three of the four stereoisomers considered, C,C bond formation occurs following a concerted mechanism. Only for one of the two *exo*-enantiomers, which is the main product in the case of catalysis with **C7**, a stepwise mechanism was found.

To investigate this finding in more detail, two-dimensional PES scans were performed for both possible *exo*-enantiomers by systematically varying the two C,C bond lengths. The results are

summarized in Figure S36. They support the observation that stepwise mechanism is only possible for the *exo*-main product.

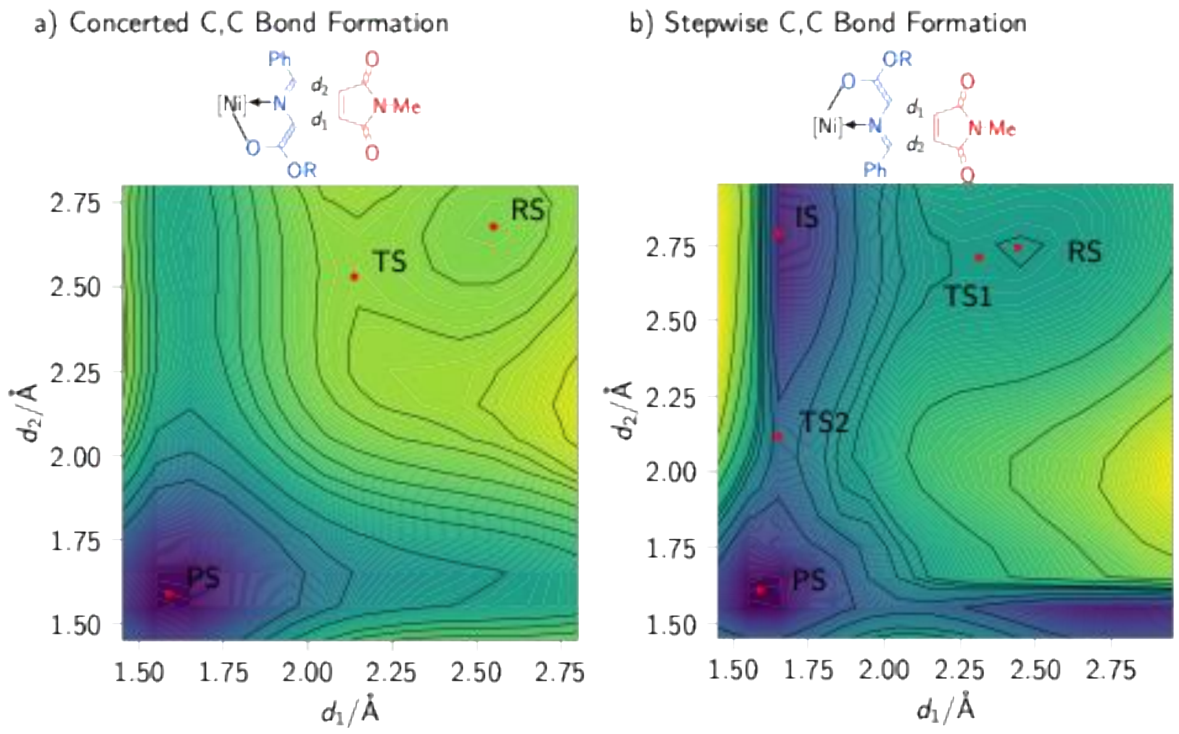

Figure S36: 2D-PES scans for concerted (a; left) and stepwise C-C bond formation (b; right). RS: reactant state, TS: transition state, IS: intermediate state, PS: product state.

Accordingly, the possibility of stabilizing the Michael intermediate, which is formed after the formation of only one C,C bond, by a hydrogen bond with the binaphthol-OH group is crucial for the existence of a stepwise reaction mechanism. For steric reasons, this arrangement is only possible for the main product.

15.3.5 Cycoladduct Protonation

According to the mechanistic model, the Gibbs free energy difference between the *exo*- and *endo*-cycloadduct protonation TSs determines the *exo*:*endo* selectivity. Since the B3LYP-D3(BJ)-COSMO value of 1.9 kJ mol^{-1} is not significant, $\Delta\Delta G$ was determined using three additional methods (PBE0,[52] M06-2X,[53] COSMO-RS)[48,49,54] (Table S14).

Table S14: Summary of ΔΔ*G* for the cycloadduct protonation TSs with different quantumchemical methods and estimated *exo*:*endo* ratio.

Method	ΔΔ*G*/kJ mol^{-1}	*exo:endo*
B3LYP-D3(BJ)-COSMO	+1.9	68:32
B3LYP-D3(BJ)-COSMO-RS	+4.9	88:12
PBE0-D3(BJ)-COSMO	+5.9	92:8
M06-2X	+11.2	99:1

Accordingly, the *exo*-selectivity is preserved when the DFT functional and the implicit solvation model are varied. The best agreement with the experimental value is obtained with the B3LYP-D3(BJ)-COSMO-RS method.

15.3.6 Kinetic Investigation

Since it is unclear how large the concentration of the catalytically active species is, an apparent experimental barrier comparable to the DFT energy span can neither be determined from the VTNA rate constant, nor from the temporal change of a substrate or product concentration, nor from a microkinetic model, since the experimental barrier according to

$$r = k_{\mathrm{rea.}}[\mathbf{C7}] \Leftrightarrow k_{\mathrm{rea.}} = \frac{r}{[\mathbf{C7}]},$$

$$\Delta G^{\ddagger}_{\mathrm{rea.}} = -RT\,\log\left(\frac{k_{\mathrm{rea.}} \cdot h}{k_{\mathrm{B}} \cdot T}\right)$$

is dependent on the concentration of the catalytically active species. Here, *r* is the experimental rate, $k_{\mathrm{rea.}}$ the experimental rate constant, *R* the universal gas constant, *h* the Planck constant, k_{B} the Boltzmann constant, *T* and the absolute temperature in Kelvin.

15.4 The *endo*-selective approach with C4

15.4.1 Precatalyst

15.4.1.1 Spin-State

According to solid-state EPR spectroscopy and SQUID magnetometry (chapter 11and 12), the precatalyst corresponding to **C4** has a high-spin state.

In contrast to that finding, DFT predicts that naked **pC4** should have a low-spin state (Table S15).

Table S15: Summary of relative Gibbs free energies of different spin-states for **pC4**.

spin state	$\Delta G°$/kJ mol^{-1} relatively to quartet	
doublet	-7.3	
quartet	0.0	This compound is referred to as **pC4**.

15.4.1.2 Adducts

Since THF is a coordinating solvent, the potential adduct formation between **pC4** and THF must be taken into account. It turns out that increasing the coordination number of the Co metal center from 4 to 5 leads to a relative stabilization of the high-spin state, which is therefore the more stable state.

Since $NEtiPr_2$ is used for activation, adduct formation with it is also investigated. The results are summarized below (Table S16).

Table S16: Summary of relative Gibbs free energies of possible adducts of **pC4** with THF and $NEtiPr_2$ in different spin-states.

adduct partner	spin state	$\Delta G°$/kJ mol^{-1} relatively to **pC4**
THF	doublet	-26.1
	quartet	-41.5
$NEtiPr_2$	doublet	+24.3
	quartet	-29.1

15.4.2 Activated Catalyst

15.4.2.1 Spin-State

According to solid-state EPR spectroscopy, SQUID magnetometry, and Evans NMR in THF (chapter 11and 12), the catalyst **C4** has a high-spin state (Table S17). This finding is in line with the DFT results.

Table S17: Summary of relative Gibbs free energies of different spin-states for **C4**.

spin state	$\Delta G°$/kJ mol^{-1} relatively to quartet	
doublet	+44.0	
quartet	0.0	This compound is referred to as **C4**.

15.4.2.2 Adducts

Again, adduct formation with THF and $NEtiPr_2$ is taken into account. It turned out, that in contrast to **C4**, adduct formation is always an endergonic process (Table S18).

Table S18: Summary of relative Gibbs free energies of possible adducts of **C4** with THF and $NEtiPr_2$ in different spin-states.

adduct patrtner	spin state	$\Delta G°$/kJ mol^{-1} relatively to **C4**
THF	doublet	-
	quartet	+4.7
$NEtiPr_2$	doublet	+49.6
	quartet	+27.8

15.4.3 C,C Bond Formation

A mechanistically interesting point is the C,C bond formation. Just as for the Ni catalyst **C7**, this step can proceed either stepwise or concertedly (Chapter 15.3.4).

It turns out that a stepwise mechanism is only found for one of the two possible *exo*-isomers. In all other cases, the double C,C bond formation step occurs concertedly in a single step. Again, the reason is that only in the case of that one *exo*-enantiomer the Michael intermediate can be stabilized by hydrogen bonding tot he binaphthol-OH to form a stable intermediate on the PES.

That result implies that if other electrophiles are used, Michael addition may well be possible instead of the 3+2 cycloaddition. The crucial point is the stabilization of this reaction pathway.

15.4.4 Estimated Selectivity

Experimental results showed that the *endo*-selective catalysis with **C4** is sensitive to the modification of the steric demand of the ester residue. Table 3 in the main text of the paper shows that Me and Bn are tolerated as ester residues in addition to Et. The use of tBu leads to a significant drop in selectivity (yield: 75%, dr = 70:30 (endo:exo), ee (endo) = 85%).

This finding can be explained by our DFT model. Figure S37 and S38 show the Gibbs energy profiles of the reaction with Et and tBu ester residue using C4. In addition to the main *endo* pathway, the TS of the C,C bond formation of the *exo* minor product is also shown. It turns out that the decrease in endo selectivity is due to the destabilization of the endo C,C bond formation TS. This destabilization can be explained by the fact that the tBu ester residue does not fit into the binding pocket spanned by the catalyst **C4** (Figure S39).

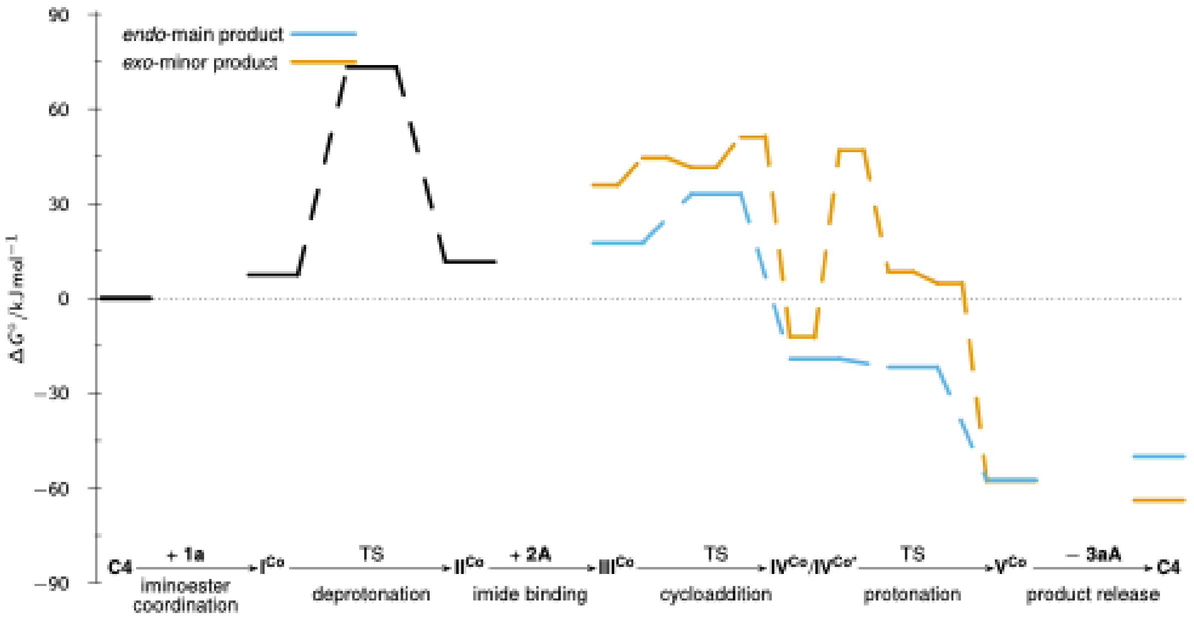

Figure S37: Gibbs free energy profile of the proposed catalytic cycle (Scheme 2 in the main text). **C4** and the isolated substrates were chosen as reference state ($\Delta G° = 0$ kJ mol^{-1}).

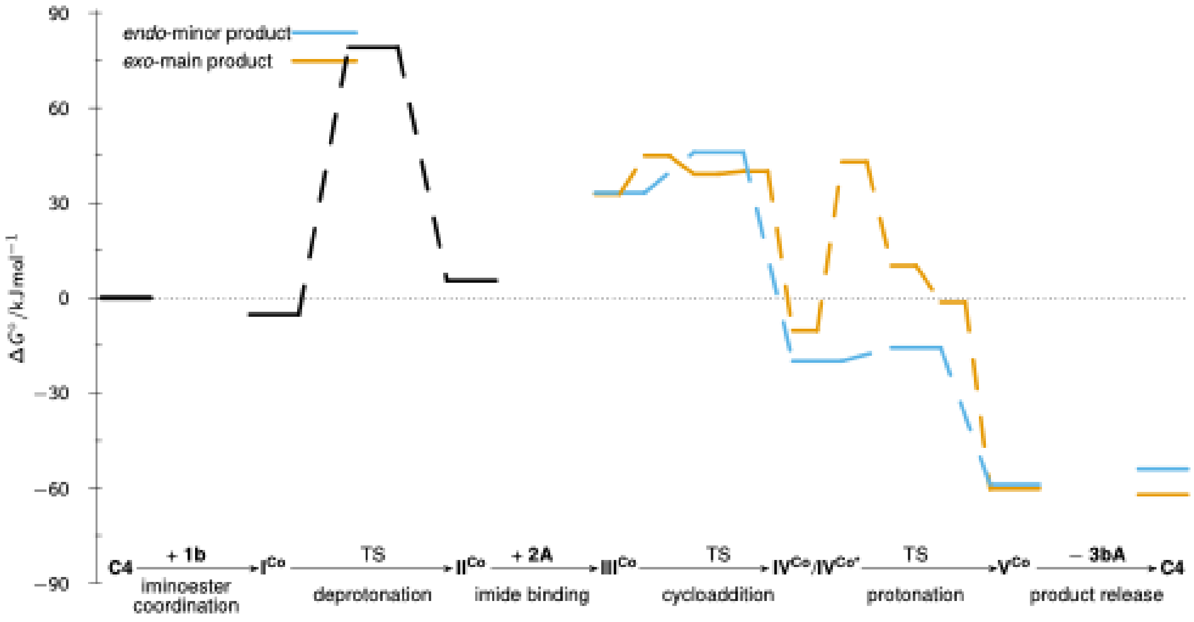

Figure S38: Gibbs free energy profile of the proposed catalytic cycle (Scheme 2 in the main text). **C4** and the isolated substrates were chosen as reference state ($\Delta G°$ = 0 kJ mol^{-1}).

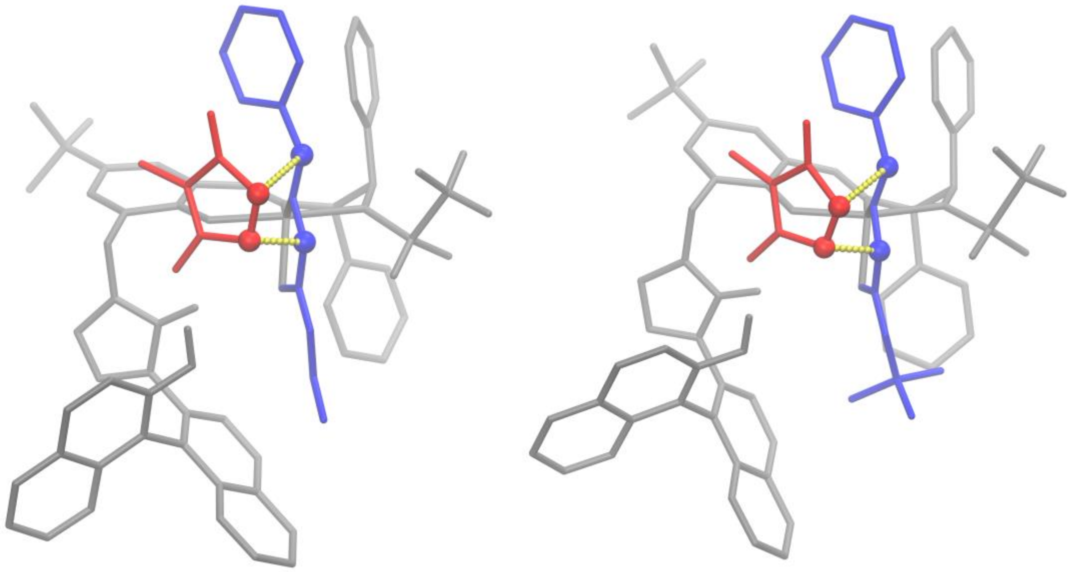

Figure S39: Comparison of the C,C bond formation TS of the endo main path with Et (left) and tBu ester residue (right).

The computational model results in a *dr* of >99:1 for the Et ester, while a value of 46:54 is obtained for the ^{t}Bu ester.

15.4.5 Kinetic Investigation

To calculate an apparent experimental barrier from the kinetic data (chapter 9.1.2) for the investigated *endo*-selective approach with **C4** the logarithmic normalized concentration of reaction product [**3aA**] can be fitted with a linear fit function of the type (Figure S40):

$$\log\left(\frac{n_0[\mathbf{1a}]-n[\mathbf{3aA}]}{\text{mol L}^{-1}}\right) = m \cdot t$$

An apparent overall reaction rate constant $k_{\text{rea.}}$ can then be estimated by using the formula:

$$k_{\text{rea.}} = -\frac{m}{[\mathbf{C4}]}$$

where m is the gradient of the linear fit function and [**C4**] = 0.05 mol% the initial concentration of the catalyst **C4**.

The apparent barrier $\Delta G^{\ddagger}_{\text{rea}}$ is given by

$$\Delta G^{\ddagger}_{\text{rea.}} = -RT\,\log\left(\frac{k_{\text{rea.}}\cdot h}{k_{\text{B}}\cdot T}\right)$$

with the apparent overall reaction rate constant $k_{\text{rea.}}$,the universal gas constant R, the Planck constant h, the Boltzmann constant k_{B} and the absolute temperature in Kelvin T.

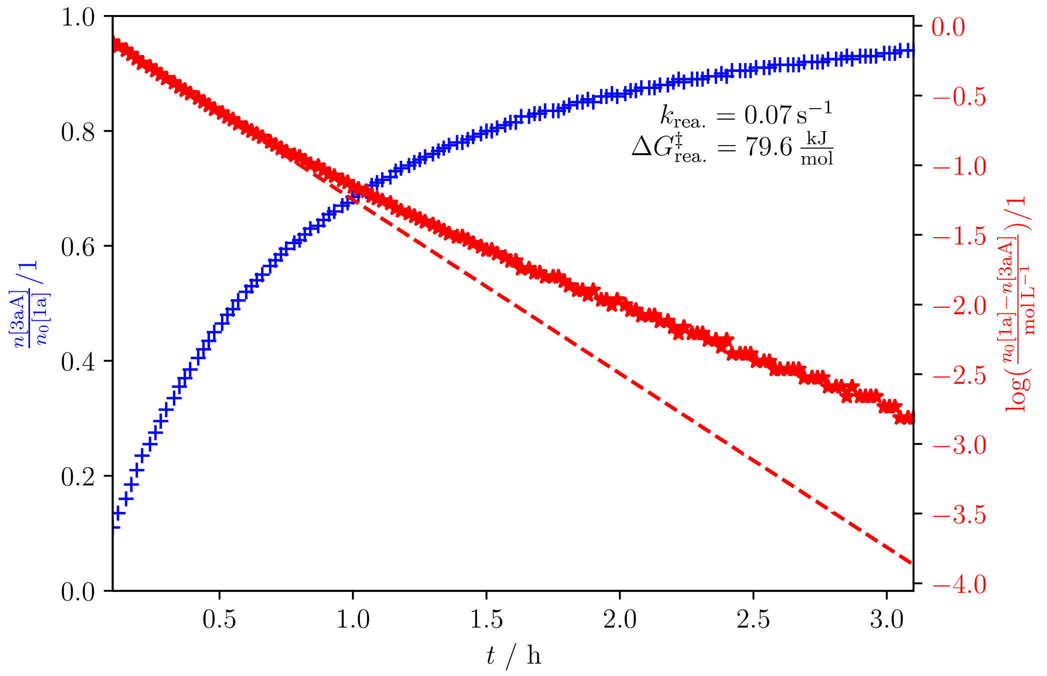

Figure S40: Concentration profile for **3aA** formation with **C4** and calculated $k_{\text{rea.}}$ and $\Delta G^{\ddagger}_{\text{rea.}}$.

15.4.6 Microkinetic Model

As a direct comparison between our Gibbs energy profile from DFT (Figure 2 in the main text) and experimental kinetic data (chapter 9.1.2) is possible via a microkinetic model, we decided to fit such a microkinetic model using methods similar to those previously applied to an asymmetric hydroboration by our groups.[55] For this purpose, we separated the catalytic

reaction according to Scheme 2 in the main text into eight elementary steps and optimized their barriers based on the DFT-derived barriers, if available (Table S19). The results of our kinetic model and the measured concentration profiles over time are compared in Figure S41, which indicates good agreement.

Table S19: Elementary reactions (R1 – R8) used to model the catalytic reaction and the corresponding Gibbs free energy barriers obtained from the microkinetic model ($\Delta G_{MM}^{‡}$) and from DFT calculations ($\Delta G_{DFT}^{‡}$) in kJ mol^{-1}, if available.

label	reaction	$\Delta G_{MM}^{‡}$	$\Delta G_{DFT}^{‡}$
iminoester coordination			
R1	**1a** + **C4** -> **I**^{Co}	53.9	–
R2	**I**^{Co} -> **1a** + **C4**	52.9	–
deprotonation			
R3	**I**^{Co} -> **II**^{Co}	73.8	66.0
R4	**II**^{Co} -> **I**^{Co}	62.8	61.7
imide binding			
R5	**2A** + **II**^{Co} -> **III**^{Co}	55.0	–
cycloaddition and protonation			
R6	**III**^{Co} -> **V**^{Co}	15.5	15.5
product release			
R7	**V**^{Co} -> **3aA** + **C4**	54.0	–
R8	**3aA** + **C4** -> **V**^{Co}	53.2	–

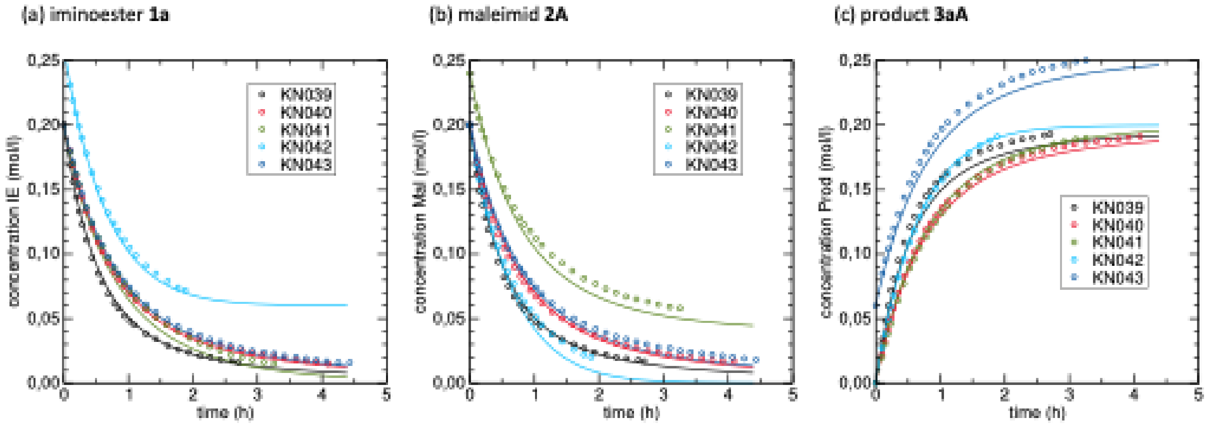

Figure S41: Measured concentration profiles (dots) vs. results from our microkinetic model (lines). The initial concentrations are summarized in chapter 9.1.2. The reaction was carried out at 298 K in THF as solvent.

Overall, the apparent barrier for the catalytic reaction **1a** + **2A** -> **3aA** from the microkinetic model is found to be +75.6 kJ mol^{-1}, which again agrees well with the calculated energetic span

of +80.8 kJ mol^{-1} that can be deducted from the Gibbs free energy profile (Figure 2 in the main text).

Overall, the apparent barrier for the catalytic reaction **1a** + **2A** -> **3aA** from the microkinetic model is found to be +75.6 kJ mol^{-1}, which again agrees well with the calculated energetic span of +80.8 kJ mol^{-1} that can be deducted from the Gibbs free energy profile (Figure 2 in the main text).

15.5 Comparison of the *endo*- and the *exo*-selective approach

In order to examine the differences between the *endo*-selective approach with **C4** and the *exo*-selective approach with **C7**, the reaction pathways to the respective main products were each analyzed under different aspects:

- Coordination Vicinity of the Metal Centers
- Hydrogen-Bonds
- Steric Interactions
- Conformational Changes
- Entropic and Enthalpic Contributions for every Reaction Step

15.5.1 Coordination Vicinity of the Metal Centers

The coordination of the metal centers is usually an important and metal-specific parameter. For this reason, all minima and transition states along the reaction pathways to the respective main product with **C4** and **C7** were investigated with regard to the coordination environments of the metal centers. The results are summarized in Table S20.

Table S20: Comparison of the coordination vicinities of the respective metal centers along the reaction pathways to the respective main product. M=Minimum, TS=transition state.

State	*endo*-selective approach with **C4**	*exo*-selective approach with **C7**
pure catalyst (M)	CN = 4, tetrahedral	CN = 4, tetrahedral
iminoester coordination (M)	CN = 5, distorted square-pyramidal	CN = 5, tetrahedral + one additional elongated bond to iminoester
deprotonation (TS)	CN = 5, distorted square-pyramidal	CN = 5, distorted square-pyramidal
azomethine ylide complex (M)	CN = 5, distorted square-pyramidal	CN = 5, distorted square-pyramidal
imide coordination (M)	CN = 5, trigonal-bipyramidal	CN = 5, distorted square-pyramidal
bond formation (TS)	CN = 5, distorted square-pyramidal	CN = 5, distorted square-pyramidal
cycloadduct (M)	CN = 5, distorted square-pyramidal	CN = 5, distorted square-pyramidal
cycloadduct rearrangement (M)	CN = 5, trigonal-bipyramidal	CN = 5, distorted square-pyramidal
protonation (TS)	CN = 5, trigonal-bipyramidal	CN = 5, distorted square-pyramidal
product-catalyst complex (M)	CN = 5, trigonal-bipyramidal	CN = 6, octahedral

By comparing the reaction pathways with **C4** and **C7** to their respective main products with regard to the coordination environments of the metal centers, it can be seen that both Co^{II} and Ni^{II} have a coordination number of five in almost all states during catalysis. The only exception is the product-catalyst complex in the case of *exo*-selective approach with **C7**, which is characterized by an octahedral coordination environment of Ni^{II} center.

Apart from that observation, a clear correlation can be drawn between the coordination environments of the Co metal centers and the relative energies of the respective states: If a trigonal-bipyramidal coordination environment is possible, the structure is clearly preferred over others without this arrangement.

15.5.2 Hydrogen-Bonds

Hydrogen bonds are known to have a stabilizing effect on the one hand and to force systems into certain configurations on the other. Therefore, the two approaches with **C4** and **C7** were investigated with regard to possible hydrogen bonds (Table S21).

Table S21: Comparison of possible hydrogen bonds along the reaction pathways to the respective main product. M=Minimum, TS=transition state.

State	*endo*-selective approach with **C4**	*exo*-selective approach with **C7**
pure catalyst (M)	-	-
iminoester coordination (M)	-	-
deprotonation (TS)	-	-
azomethine ylide complex (M)	-	Binaphthol-OH…O-Sulfonamide
imide coordination (M)	Binaphthol-OH…O-Maleimide	Binaphthol-OH…O-Maleimide
bond formation (TS)	Binaphthol-OH…O-Maleimide	Binaphthol-OH…O-Maleimide
cycloadduct (M)	Binaphthol-OH…O-Maleimide	Binaphthol-OH…O-Maleimide
cycloadduct rearrangement (M)	Binaphthol-OH…N-Cycloadduct	Binaphthol-OH…O-Sulfonamide
protonation (TS)		-
product-catalyst complex	Imidazolium-H…N-Product	-

That comparison from above shows a clear similarity between the two approaches: Following the paradigm of cooperative activation of electrophile and nucleophile, the maleimide is activated via a hydrogen bond from the binaphthol group.

On the other hand, the difference between the two methods is also clearly recognizable by the hydrogen bond between the binaphthol group and the sulfonamide unit, which is only possible in the case of **C7**. That hydrogen bond leads to a folding of the catalyst. The corresponding states are clearly favored energetically through this conformation.

15.5.3 Steric Interactions

Probably the most important point in the context of asymmetric homogeneous catalysis are steric interactions, as these can significantly influence the accessibility of prochiral centers. Table S22 compares the *endo*- and *exo*-selective approaches with **C4** and **C7**, respectively, with respect to additional steric interactions that occurred despite the great similarities between the two catalysts.

Table S22: Comparison of additional steric. interactions along the reaction pathways to the respective main product. M=Minimum, TS=transition state.

State	*endo*-selective approach with **C4**	*exo*-selective approach with **C7**
pure catalyst (M)		
iminoester coordination (TS)		
deprotonation (TS)	repulsion due to short Et-Ph-distance and interaction with SO_2-group	
azomethine ylide complex (M)		
imide coordination (M)		
bond formation (M)		
cycloadduct (M)		
cycloadduct rearrangement (M)		
protonation (TS)		
product-catalyst complex (M)		

Particularly striking here is the steric repulsion of the iminoester with the chiral backbone and the SO2 group of the catalyst in the deprotonation transition state with **C4**. This additional repulsion correlates with the high deprotonation barrier found.

15.5.4 Conformational Changes

The quantumchemical investigation of the underlying reaction mechanisms reduces the mechanisms themselves to three sub-steps: Iminoester deprotonation, C,C bond formation, and cycloadduct protonation. Due to the high flexibility of the catalysts **C4** and **C7**, relatively large rearrangements are necessary between these reaction steps, for which small barriers are expected, but which may nevertheless represent a significant difference between the two approaches. At the same time, similarities between the two approaches can be identified.

Table S23: Comparison of conformational changes along the reaction pathways to the respective main product. M=Minimum, TS=transition state.

State	*endo*-selective approach with **C4**	*exo*-selective approach with **C7**
pure catalyst (M)		
iminoester coordination (M)		
deprotonation (TS)	opening the aryloxide O...Co bond	opening the aryloxide O...Ni bond
azomethine ylide complex (M)	Binaphthol-OH oriented towards the outer sphere	Folding of the catalyst structure due to hydrogen bond (Binaphthol-OH…O-Sulfonamide)
imide coordination (M)		Unfolding due to hydrogen bond (dto.) break
bond formation (TS)		
cycloadduct (M)		
cycloadduct rearrangement (M)	Restoring the trigonal bipyramidal coordination vicinity of the metal center	Re-folding of the catalyst structure due to hydrogen bond (dto.)
protonation (TS)	direct H^+-transfer	Breaking the hydrogen bond and close proximity of negative partial charges
product-catalyst complex (M)	180° rotation around the product-O…Co-bond	180° rotation around the product-O…Ni-bond

The comparison of the conformal changes shows that the catalyst in the *exo*-selective approach with **C7** performs a folding/unfolding movement due to the hydrogen bond between binaphthol and sulfonamide. The folding leads to an energetic stabilization of the corresponding minima. As a result, the zigzag profile of the Gibbs free-energy profile can occur.

Since the same folding of the catalyst for the *endo*-selective approach with **C4** is not possible for steric reasons, we expect a flatter Gibbs free-energy profile, which is also maintained.

15.5.5 Entropic and Enthalpic Contributions for every Reaction Step

Additionally, the entropic (Δ*S*°) and enthalpic (Δ*H*°) contributions to the Gibbs free-energy (Δ*G*°) were analyzed for each reaction step (Table S24).

Table S24: Comparison of entropic and enthalpic contributions for every reaction step along the reaction pathways to the respective main product. The catalyst **C*x*** (***x*** = 4,7) and the isolated substrates were chosen as reference states. M=Minimum, TS=transition state.

State	*endo*-selective approach with **C4**	*exo*-selective approach with **C7**
pure catalyst (M)	Δ*S*° = 0.0 kJ mol^{-1}<br>Δ*H*° = 0.0 kJ mol^{-1}<br>Δ*G*° = 0.0 kJ mol^{-1}	Δ*S*° = 0.0 kJ mol^{-1}<br>Δ*H*° = 0.0 kJ mol^{-1}<br>Δ*G*° = 0.0 kJ mol^{-1}
iminoester coordination (M)	Δ*S*° = -60.7 kJ mol^{-1}<br>Δ*H*° = -53.4 kJ mol^{-1}<br>Δ*G*° = +7.3 kJ mol^{-1}	Δ*S*° = -62.1 kJ mol^{-1}<br>Δ*H*° = -69.6 kJ mol^{-1}<br>Δ*G*° = -7.5 kJ mol^{-1}
deprotonation (TS)	Δ*S*° = -63.3 kJ mol^{-1}<br>Δ*H*° = +10.1 kJ mol^{-1}<br>Δ*G*° = +73.3 kJ mol^{-1}	Δ*S*° = -64.1 kJ mol^{-1}<br>Δ*H*° = -20.6 kJ mol^{-1}<br>Δ*G*° = +43.5 kJ mol^{-1}
azomethine ylide complex (M)	Δ*S*° = -62.8 kJ mol^{-1}<br>Δ*H*° = -51.1 kJ mol^{-1}<br>Δ*G*° = +11.6 kJ mol^{-1}	Δ*S*° = -62.3 kJ mol^{-1}<br>Δ*H*° = -61.8 kJ mol^{-1}<br>Δ*G*° = +0.6 kJ mol^{-1}
imide coordination (M)	Δ*S*° = -118.9 kJ mol^{-1}<br>Δ*H*° = -101.3 kJ mol^{-1}<br>Δ*G*° = +17.6 kJ mol^{-1}	Δ*S*° = -117.1 kJ mol^{-1}<br>Δ*H*° = -107.0 kJ mol^{-1}<br>Δ*G*° = +10.1 kJ mol^{-1}
bond formation (TS)	Δ*S*° = -124.6 kJ mol^{-1}<br>Δ*H*° = -91.5 kJ mol^{-1}<br>Δ*G*° = +33.1 kJ mol^{-1}	transition state 1:<br>Δ*S*° = -120.7 kJ mol^{-1}<br>Δ*H*° = -104.8 kJ mol^{-1}<br>Δ*G*° = +15.9 kJ mol^{-1}<br>intermediate:<br>Δ*S*° = -122.2 kJ mol^{-1}<br>Δ*H*° = -107.1 kJ mol^{-1}<br>Δ*G*° = +15.1 kJ mol^{-1}<br>Transition state 2:<br>Δ*S*° = -124.6 kJ mol^{-1}<br>Δ*H*° = -93.5 kJ mol^{-1}<br>Δ*G*° = +31.1 kJ mol^{-1}
cycloadduct (M)	Δ*S*° = -125.7 kJ mol^{-1}<br>Δ*H*° = -144.8 kJ mol^{-1}<br>Δ*G*° = -19.1 kJ mol^{-1}	Δ*S*° = -124.1 kJ mol^{-1}<br>Δ*H*° = -107.5 kJ mol^{-1}<br>Δ*G*° = +16.5 kJ mol^{-1}
cycloadduct rearrangement (M)	Δ*S*° = -127.04 kJ mol^{-1}<br>Δ*H*° = -146.4 kJ mol^{-1}<br>Δ*G*° = -19.1 kJ mol^{-1}	Δ*S*° = -123.3 kJ mol^{-1}<br>Δ*H*° = -139.0 kJ mol^{-1}<br>Δ*G*° = -15.7 kJ mol^{-1}
protonation (M)	Δ*S*° = -129.6 kJ mol^{-1}<br>Δ*H*° = -151.2 kJ mol^{-1}<br>Δ*G*° = -21.7 kJ mol^{-1}	Δ*S*° = -129.1 kJ mol^{-1}<br>Δ*H*° = -88.0 kJ mol^{-1}<br>Δ*G*° = +41.1 kJ mol^{-1}

product-catalyst complex (M)	$\Delta S°$ = -125.5 kJ mol^{-1}<br>$\Delta H°$ = -182.9 kJ mol^{-1}<br>$\Delta G°$ = -57.5 kJ mol^{-1}	$\Delta S°$ = -126.2 kJ mol^{-1}<br>$\Delta H°$ = -203.8 kJ mol^{-1}<br>$\Delta G°$ = -77.6 kJ mol^{-1}

The analysis shows that the entropic penalty of the two substrate coordination steps is already compensated by the enthalpic benefit of the substate binding. Consequently, the reaction barriers are essentially caused by the enthalpic contributions.

15.6 Data behind the Figures

Table S25: Raw date of the Gibbs free energy profile shown in Figure 2 in the main text.

state	$\Delta G°$/kJ mol^{-1} (*endo*)
**C4**	0.0
**I**^{C4}	+7.5
TS (**I**^{4} -> **II**^{C4})	+73.3
**II**^{C4}	+11.6
**III**^{C4}	+17.6
TS (**III**^{C4} -> **IV**^{C4})	+33.1
**IV**^{C4}	-19.1
**IV**^{C4*}	-19.1
TS (**IV**^{C4*} -> **V**^{C4})	-21.7
**V**^{C4}	-57.5
**3aA**	-50.0

Table S26: Raw date of the Gibbs free energy profile shown in Figure 2 in the main text.

state	$\Delta G°$/kJ mol^{-1} (*exo*)	$\Delta G°$/kJ mol^{-1} (*endo*)
**C7**	0.0	0.0
**I**^{C7}	-7.5	-7.5
TS (**I**^{C7} -> **II**^{C7})	+46.2	+46.2
**II**^{C7}	+0.6	+0.6
**III**^{C7}	+11.2	+3.5
TS1 (**III**^{C7} -> **IV**^{C7})	+17.0	+18.4
Inter (**III**^{C7} -> **IV**^{C7})	+14.3	
TS2 (**III**^{C7} -> **IV**^{C7})	+31.1	
**IV**^{C7}	+16.5	-17.8
**IV**^{C7*}	-15.7	-27.8
TS (**IV**^{C7*} -> **V**^{C7})	+41.1	+43.0
**V**^{C7}	-77.6	-55.0
**3bA**	-63.9	-54.0

16 NMR Data

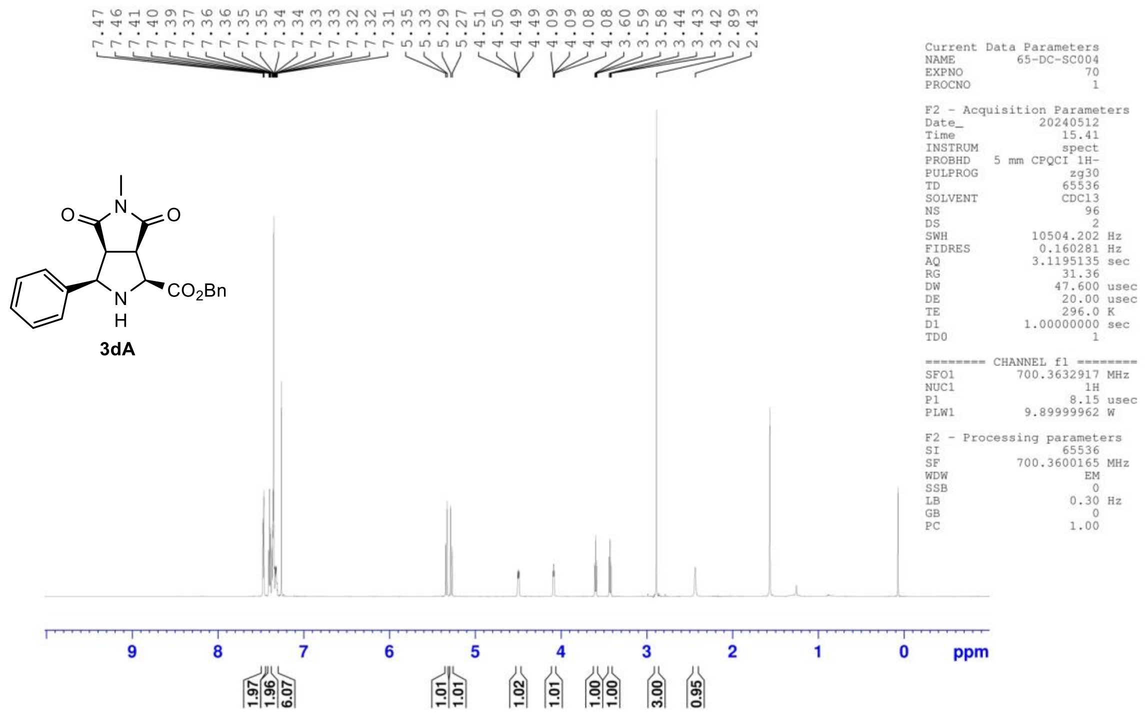
O
N
O
CO2Bn
N
H
3dA
7.47 7.46 7.41 7.40 7.39 7.37 7.36 7.36 7.35 7.35 7.34 7.34 7.33 7.33 7.33 7.32 7.32 7.31 5.35 5.33 5.29 5.27 4.51 4.50 4.49 4.49 4.09 4.09 4.08 4.08 3.60 3.59 3.58 3.44 3.43 3.42 2.89 2.43
1.97 1.96 6.07 1.01 1.01 1.02 1.01 1.00 1.00 3.00 0.95
9 8 7 6 5 4 3 2 1 0 ppm
Current Data Parameters
NAME 65-DC-SC004
EXPNO 70
PROCNO 1
F2 - Acquisition Parameters
Date_ 20240512
Time 15.41
INSTRUM spect
PROBHD 5 mm CPQCI 1H-
PULPROG zg30
TD 65536
SOLVENT CDCl3
NS 96
DS 2
SWH 10504.202 Hz
FIDRES 0.160281 Hz
AQ 3.1195135 sec
RG 31.36
DW 47.600 usec
DE 20.00 usec
TE 296.0 K
D1 1.00000000 sec
TD0 1
======== CHANNEL f1 ========
SFO1 700.3632917 MHz
NUC1 1H
P1 8.15 usec
PLW1 9.89999962 W
F2 - Processing parameters
SI 65536
SF 700.3600165 MHz
WDW EM
SSB 0
LB 0.30 Hz
GB 0
PC 1.00

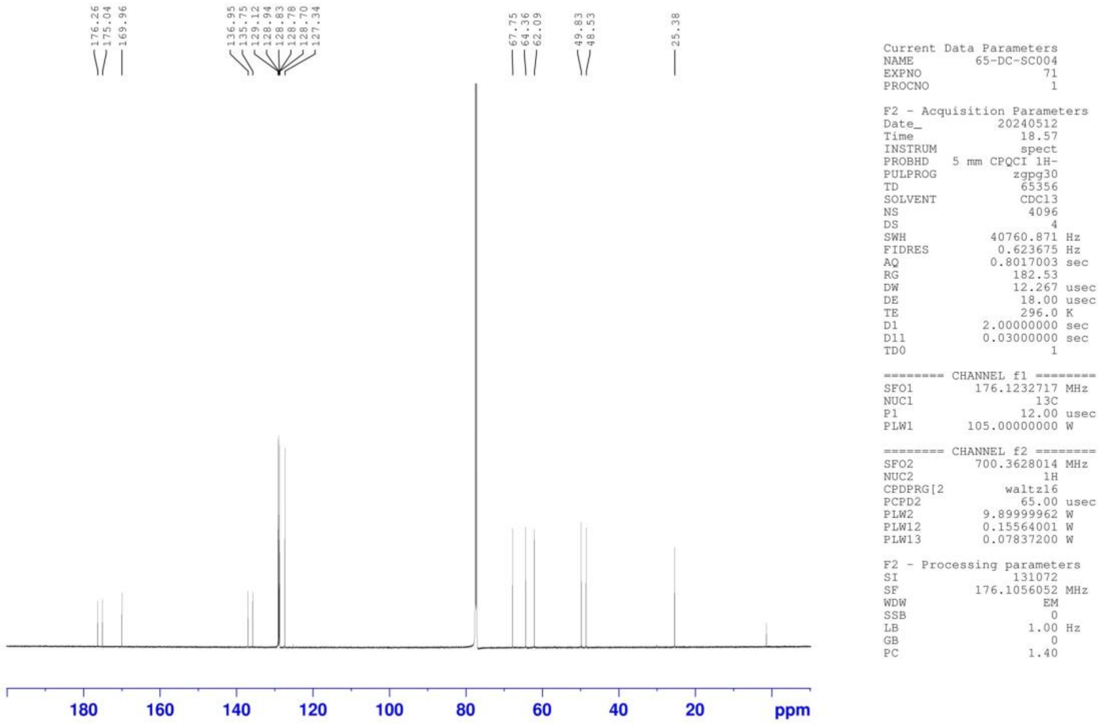
176.26 175.04 169.96 136.95 135.75 129.12 128.94 128.83 128.78 128.70 127.34 67.75 64.36 62.09 49.83 48.53 25.38
180 160 140 120 100 80 60 40 20 ppm
Current Data Parameters
NAME 65-DC-SC004
EXPNO 71
PROCNO 1
F2 - Acquisition Parameters
Date_ 20240512
Time 18.57
INSTRUM spect
PROBHD 5 mm CPQCI 1H-
PULPROG zgpg30
TD 65356
SOLVENT CDCl3
NS 4096
DS 4
SWH 40760.871 Hz
FIDRES 0.623675 Hz
AQ 0.8017003 sec
RG 182.53
DW 12.267 usec
DE 18.00 usec
TE 296.0 K
D1 2.00000000 sec
D11 0.03000000 sec
TD0 1
======== CHANNEL f1 ========
SFO1 176.1232717 MHz
NUC1 13C
P1 12.00 usec
PLW1 105.00000000 W
======== CHANNEL f2 ========
SFO2 700.3628014 MHz
NUC2 1H
CPDPRG[2 waltz16
PCPD2 65.00 usec
PLW2 9.89999962 W
PLW12 0.15564001 W
PLW13 0.07837200 W
F2 - Processing parameters
SI 131072
SF 176.1056052 MHz
WDW EM
SSB 0
LB 1.00 Hz
GB 0
PC 1.40

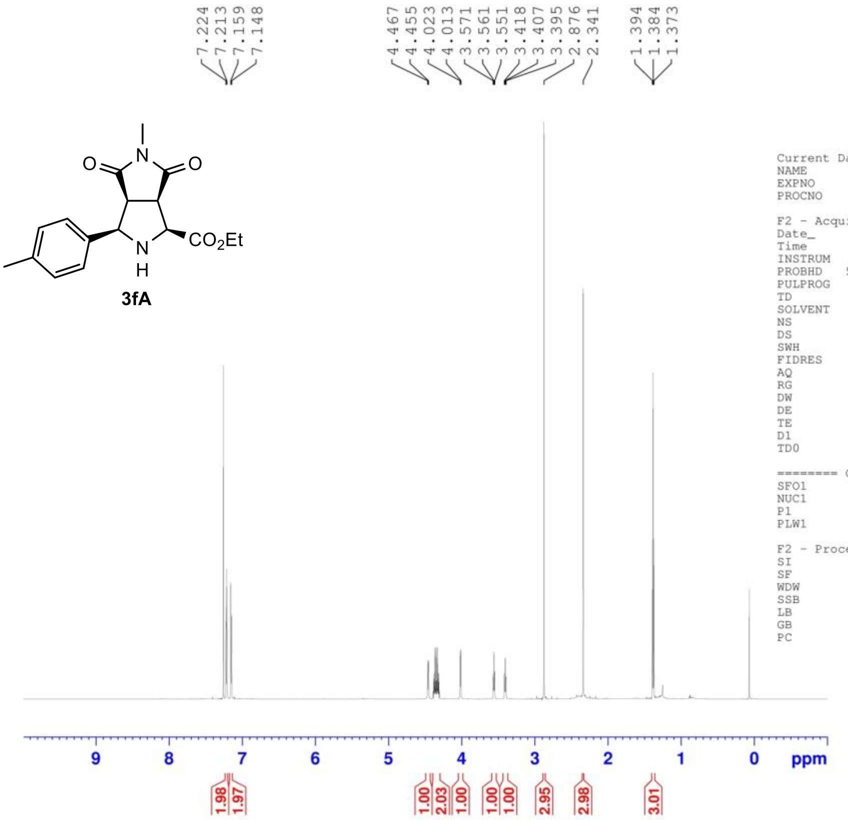

Current Data Parameters
NAME 65-DC-SC77
EXPNO 80
PROCNO 1

F2 - Acquisition Parameters
Date_ 20231129
Time 0.05
INSTRUM spect
PROBHD 5 mm CPQCI 1H-
PULPROG zg30
TD 65536
SOLVENT CDCl3
NS 120
DS 2
SWH 10504.202 Hz
FIDRES 0.160281 Hz
AQ 3.1195135 sec
RG 24.82
DW 47.600 usec
DE 20.00 usec
TE 298.0 K
D1 1.00000000 sec
TD0 1

======== CHANNEL f1 ========
SFO1 700.3632917 MHz
NUC1 1H
P1 8.15 usec
PLW1 9.89999962 W

F2 - Processing parameters
SI 65536
SF 700.3600157 MHz
WDW EM
SSB 0
LB 0.30 Hz
GB 0
PC 1.00

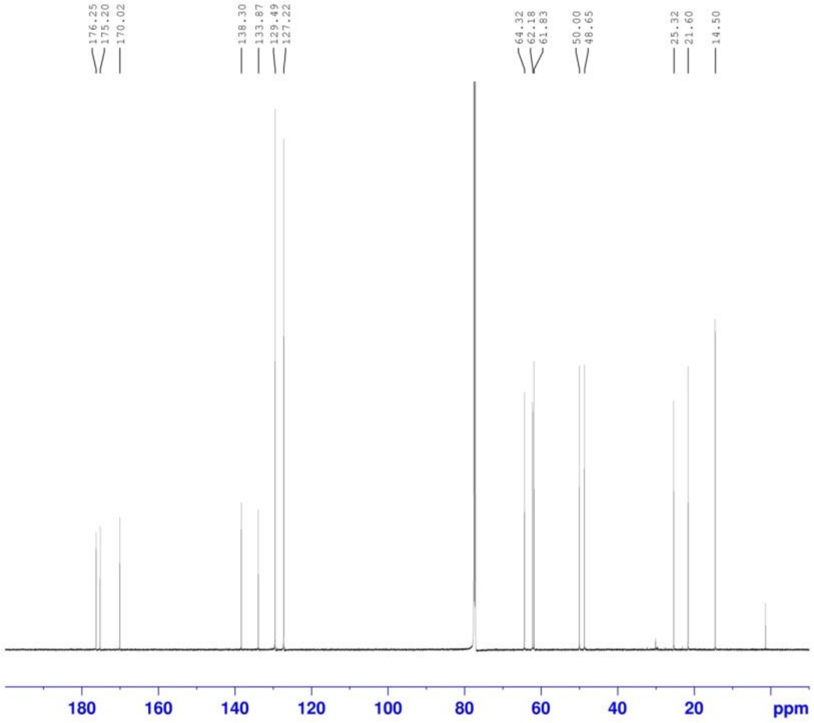

Current Data Parameters
NAME 65-DC-SC77
EXPNO 81
PROCNO 1

F2 - Acquisition Parameters
Date_ 20231129
Time 3.22
INSTRUM spect
PROBHD 5 mm CPQCI 1H-
PULPROG zgpg30
TD 65356
SOLVENT CDCl3
NS 4096
DS 4
SWH 40760.871 Hz
FIDRES 0.623675 Hz
AQ 0.8017003 sec
RG 182.53
DW 12.267 usec
DE 18.00 usec
TE 298.0 K
D1 2.00000000 sec
D11 0.03000000 sec
TD0 1

======== CHANNEL f1 ========
SFO1 176.1232717 MHz
NUC1 13C
P1 12.00 usec
PLW1 105.00000000 W

======== CHANNEL f2 ========
SFO2 700.3628014 MHz
NUC2 1H
CPDPRG[2 waltz16
PCPD2 65.00 usec
PLW2 9.89999962 W
PLW12 0.15564001 W
PLW13 0.07837200 W

F2 - Processing parameters
SI 131072
SF 176.1056045 MHz
WDW EM
SSB 0
LB 1.00 Hz
GB 0
PC 1.40

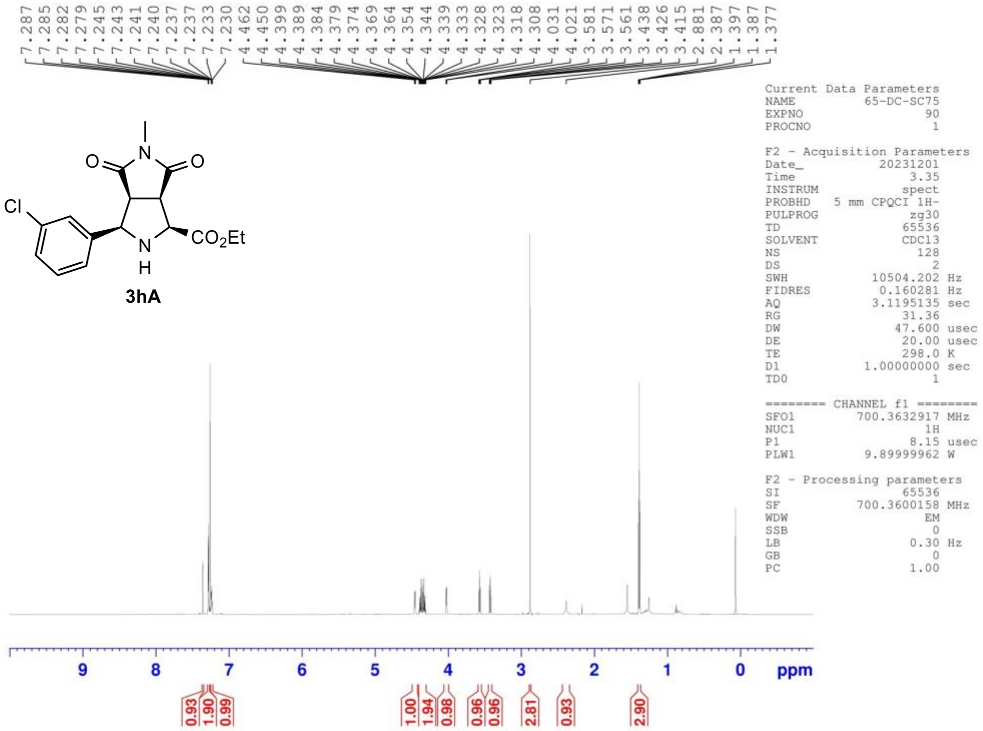
7.287 7.285 7.282 7.279 7.245 7.243 7.241 7.240 7.237 7.237 7.233 7.230 4.462 4.450 4.399 4.389 4.384 4.379 4.374 4.369 4.364 4.354 4.344 4.339 4.333 4.328 4.323 4.318 4.308 4.031 4.021 3.581 3.571 3.561 3.438 3.426 3.415 2.881 2.387 1.397 1.387 1.377
O
N
O
Cl
N
H
CO_2Et
3hA
Current Data Parameters
NAME 65-DC-SC75
EXPNO 90
PROCNO 1
F2 - Acquisition Parameters
Date_ 20231201
Time 3.35
INSTRUM spect
PROBHD 5 mm CPQCI 1H-
PULPROG zg30
TD 65536
SOLVENT CDCl3
NS 128
DS 2
SWH 10504.202 Hz
FIDRES 0.160281 Hz
AQ 3.1195135 sec
RG 31.36
DW 47.600 usec
DE 20.00 usec
TE 298.0 K
D1 1.00000000 sec
TD0 1
======== CHANNEL f1 ========
SFO1 700.3632917 MHz
NUC1 1H
P1 8.15 usec
PLW1 9.89999962 W
F2 - Processing parameters
SI 65536
SF 700.3600158 MHz
WDW EM
SSB 0
LB 0.30 Hz
GB 0
PC 1.00
9 8 7 6 5 4 3 2 1 0 ppm
0.93 1.90 0.99 1.00 1.94 0.98 0.96 0.96 2.81 0.93 2.90

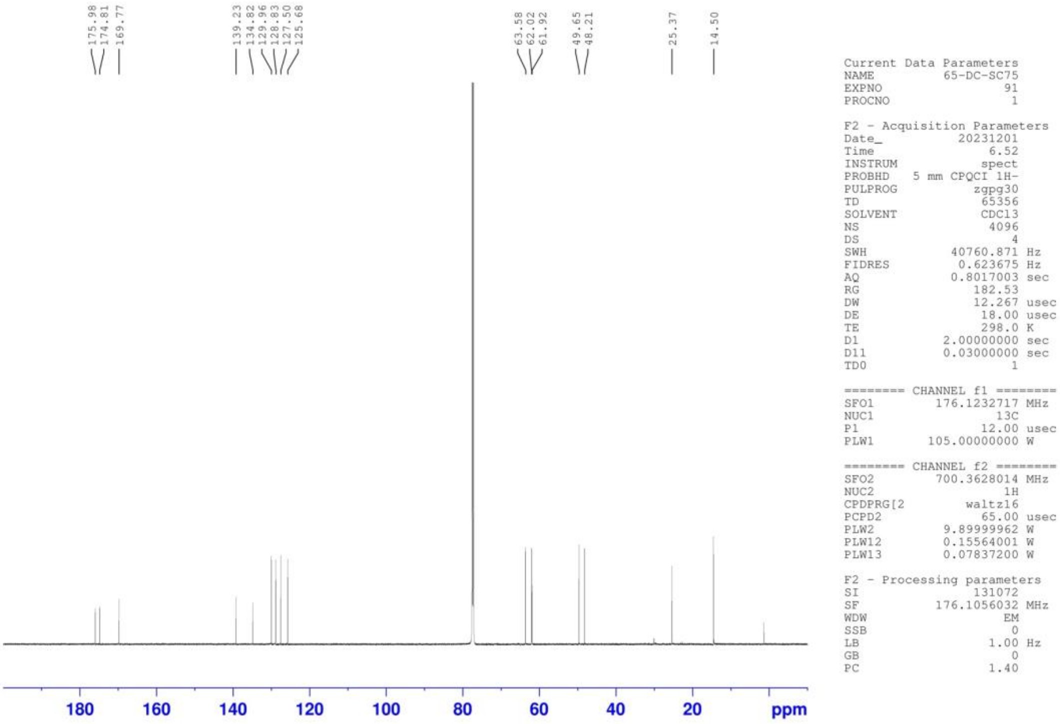
175.98 174.81 169.77 139.23 134.82 129.96 128.83 127.50 125.68 63.58 62.02 61.92 49.65 48.21 25.37 14.50
Current Data Parameters
NAME 65-DC-SC75
EXPNO 91
PROCNO 1
F2 - Acquisition Parameters
Date_ 20231201
Time 6.52
INSTRUM spect
PROBHD 5 mm CPQCI 1H-
PULPROG zgpg30
TD 65356
SOLVENT CDCl3
NS 4096
DS 4
SWH 40760.871 Hz
FIDRES 0.623675 Hz
AQ 0.8017003 sec
RG 182.53
DW 12.267 usec
DE 18.00 usec
TE 298.0 K
D1 2.00000000 sec
D11 0.03000000 sec
TD0 1
======== CHANNEL f1 ========
SFO1 176.1232717 MHz
NUC1 13C
P1 12.00 usec
PLW1 105.00000000 W
======== CHANNEL f2 ========
SFO2 700.3628014 MHz
NUC2 1H
CPDPRG[2 waltz16
PCPD2 65.00 usec
PLW2 9.89999962 W
PLW12 0.15564001 W
PLW13 0.07837200 W
F2 - Processing parameters
SI 131072
SF 176.1056032 MHz
WDW EM
SSB 0
LB 1.00 Hz
GB 0
PC 1.40
180 160 140 120 100 80 60 40 20 ppm

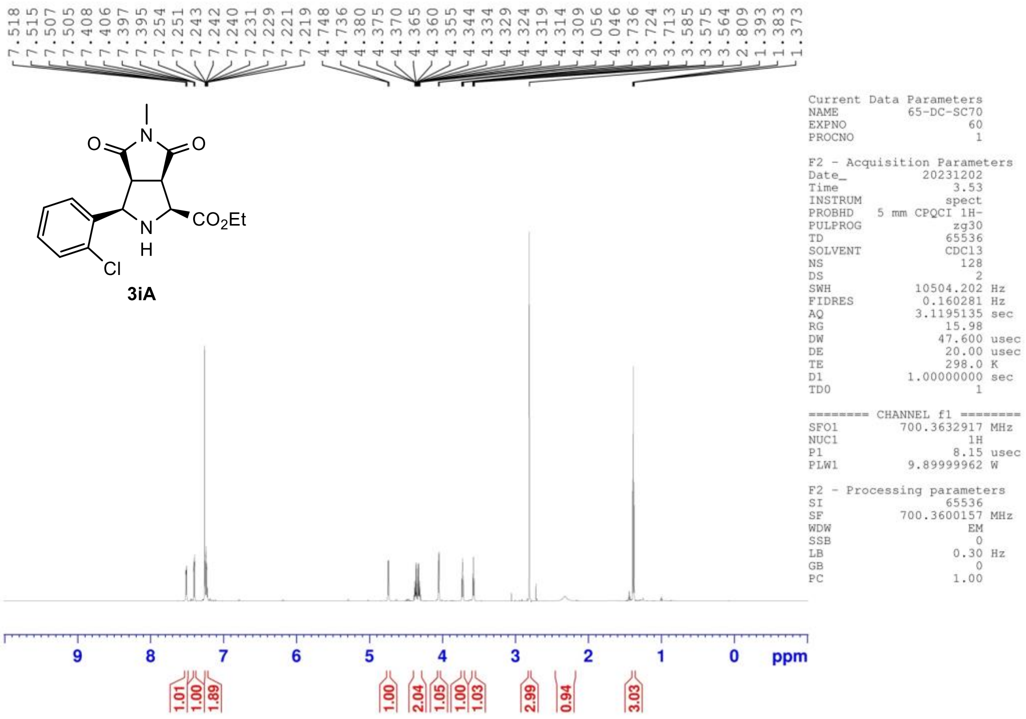
7.518
7.515
7.507
7.505
7.408
7.406
7.397
7.395
7.254
7.251
7.243
7.242
7.240
7.231
7.229
7.221
7.219
4.748
4.736
4.380
4.375
4.370
4.365
4.360
4.355
4.344
4.334
4.329
4.324
4.319
4.314
4.309
4.056
4.046
3.736
3.724
3.713
3.585
3.575
3.564
2.809
1.393
1.383
1.373
O
N
O
CO_2Et
N
H
Cl
3iA
Current Data Parameters
NAME 65-DC-SC70
EXPNO 60
PROCNO 1
F2 - Acquisition Parameters
Date_ 20231202
Time 3.53
INSTRUM spect
PROBHD 5 mm CPQCI 1H-
PULPROG zg30
TD 65536
SOLVENT CDCl3
NS 128
DS 2
SWH 10504.202 Hz
FIDRES 0.160281 Hz
AQ 3.1195135 sec
RG 15.98
DW 47.600 usec
DE 20.00 usec
TE 298.0 K
D1 1.00000000 sec
TD0 1
======== CHANNEL f1 ========
SFO1 700.3632917 MHz
NUC1 1H
P1 8.15 usec
PLW1 9.89999962 W
F2 - Processing parameters
SI 65536
SF 700.3600157 MHz
WDW EM
SSB 0
LB 0.30 Hz
GB 0
PC 1.00
9
8
7
6
5
4
3
2
1
0
ppm
1.01
1.00
1.89
1.00
2.04
1.05
1.00
1.03
2.99
0.94
3.03

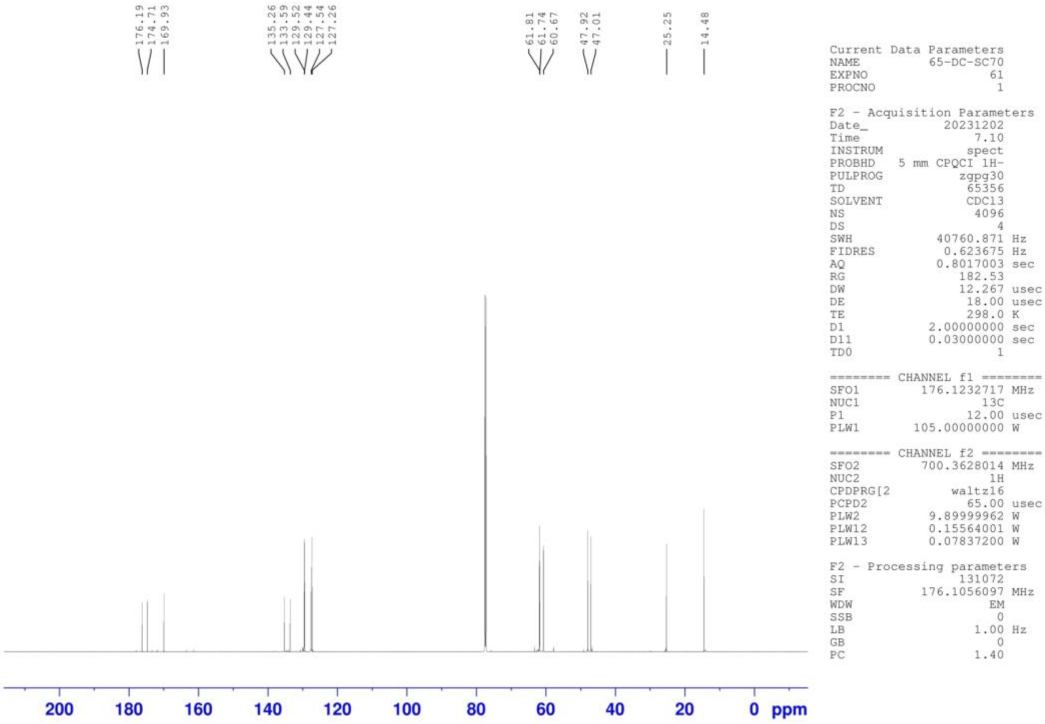
176.19
174.71
169.93
135.26
133.59
129.52
129.44
127.54
127.26
61.81
61.74
60.67
47.92
47.01
25.25
14.48
Current Data Parameters
NAME 65-DC-SC70
EXPNO 61
PROCNO 1
F2 - Acquisition Parameters
Date_ 20231202
Time 7.10
INSTRUM spect
PROBHD 5 mm CPQCI 1H-
PULPROG zgpg30
TD 65356
SOLVENT CDCl3
NS 4096
DS 4
SWH 40760.871 Hz
FIDRES 0.623675 Hz
AQ 0.8017003 sec
RG 182.53
DW 12.267 usec
DE 18.00 usec
TE 298.0 K
D1 2.00000000 sec
D11 0.03000000 sec
TD0 1
======== CHANNEL f1 ========
SFO1 176.1232717 MHz
NUC1 13C
P1 12.00 usec
PLW1 105.00000000 W
======== CHANNEL f2 ========
SFO2 700.3628014 MHz
NUC2 1H
CPDPRG[2 waltz16
PCPD2 65.00 usec
PLW2 9.89999962 W
PLW12 0.15564001 W
PLW13 0.07837200 W
F2 - Processing parameters
SI 131072
SF 176.1056097 MHz
WDW EM
SSB 0
LB 1.00 Hz
GB 0
PC 1.40
200
180
160
140
120
100
80
60
40
20
0
ppm

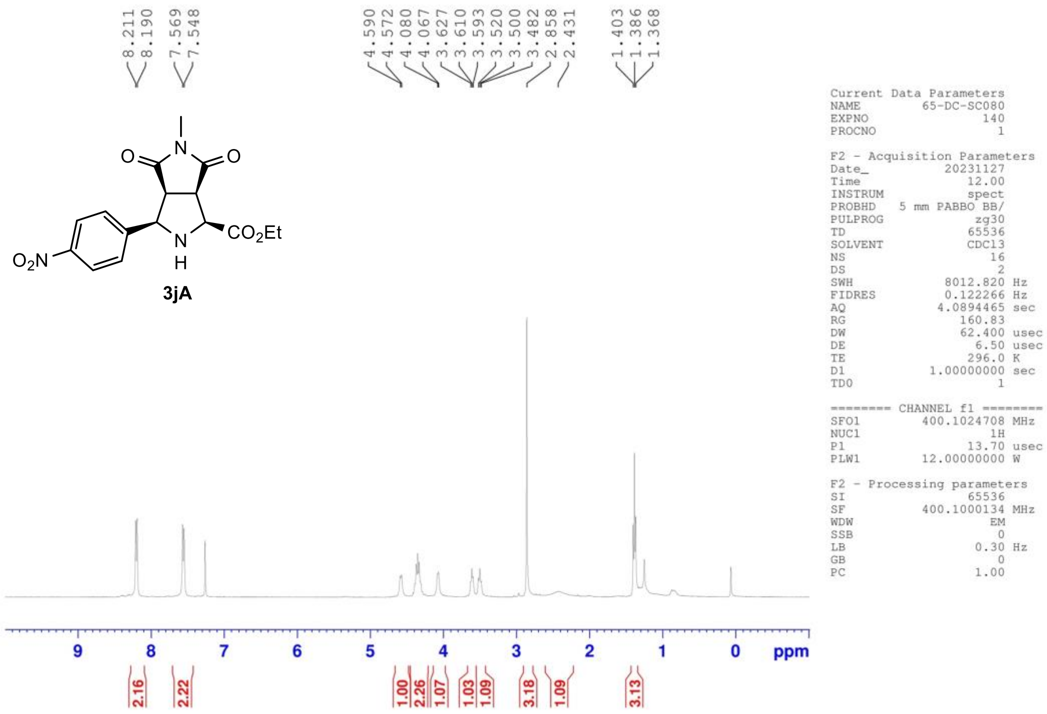
8.211
8.190
7.569
7.548
4.590
4.572
4.080
4.067
3.627
3.610
3.593
3.520
3.500
3.482
2.858
2.431
1.403
1.386
1.368
O
N
O
O_2N
N
H
CO_2Et
3jA
Current Data Parameters
NAME 65-DC-SC080
EXPNO 140
PROCNO 1
F2 - Acquisition Parameters
Date_ 20231127
Time 12.00
INSTRUM spect
PROBHD 5 mm PABBO BB/
PULPROG zg30
TD 65536
SOLVENT CDCl3
NS 16
DS 2
SWH 8012.820 Hz
FIDRES 0.122266 Hz
AQ 4.0894465 sec
RG 160.83
DW 62.400 usec
DE 6.50 usec
TE 296.0 K
D1 1.00000000 sec
TD0 1
======== CHANNEL f1 ========
SFO1 400.1024708 MHz
NUC1 1H
P1 13.70 usec
PLW1 12.00000000 W
F2 - Processing parameters
SI 65536
SF 400.1000134 MHz
WDW EM
SSB 0
LB 0.30 Hz
GB 0
PC 1.00
9 8 7 6 5 4 3 2 1 0 ppm
2.16
2.22
1.00
2.26
1.07
1.03
1.09
3.18
1.09
3.13

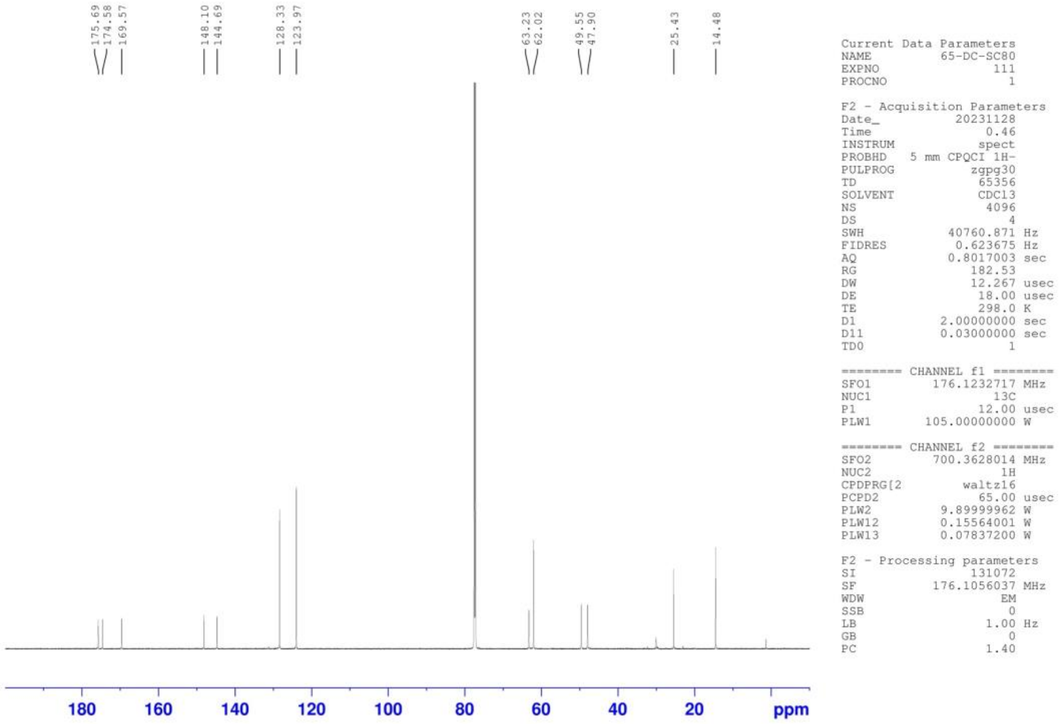
175.69
174.58
169.57
148.10
144.69
128.33
123.97
63.23
62.02
49.55
47.90
25.43
14.48
Current Data Parameters
NAME 65-DC-SC80
EXPNO 111
PROCNO 1
F2 - Acquisition Parameters
Date_ 20231128
Time 0.46
INSTRUM spect
PROBHD 5 mm CPQCI 1H-
PULPROG zgpg30
TD 65356
SOLVENT CDCl3
NS 4096
DS 4
SWH 40760.871 Hz
FIDRES 0.623675 Hz
AQ 0.8017003 sec
RG 182.53
DW 12.267 usec
DE 18.00 usec
TE 298.0 K
D1 2.00000000 sec
D11 0.03000000 sec
TD0 1
======== CHANNEL f1 ========
SFO1 176.1232717 MHz
NUC1 13C
P1 12.00 usec
PLW1 105.00000000 W
======== CHANNEL f2 ========
SFO2 700.3628014 MHz
NUC2 1H
CPDPRG[2 waltz16
PCPD2 65.00 usec
PLW2 9.89999962 W
PLW12 0.15564001 W
PLW13 0.07837200 W
F2 - Processing parameters
SI 131072
SF 176.1056037 MHz
WDW EM
SSB 0
LB 1.00 Hz
GB 0
PC 1.40
180 160 140 120 100 80 60 40 20 ppm

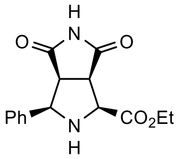
H
O
N
O
Ph
N
H
CO2Et

3aB

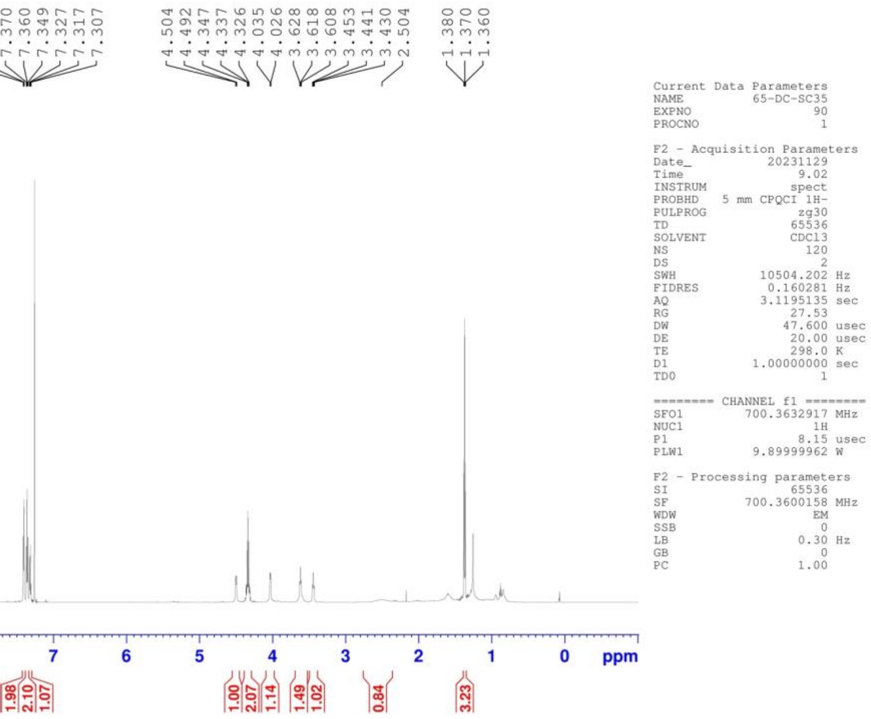
7.843
7.413
7.402
7.370
7.360
7.349
7.327
7.317
7.307
4.504
4.492
4.347
4.337
4.326
4.035
4.026
3.628
3.618
3.608
3.453
3.441
3.430
2.504
1.380
1.370
1.360
Current Data Parameters
NAME 65-DC-SC35
EXPNO 90
PROCNO 1
F2 - Acquisition Parameters
Date_ 20231129
Time 9.02
INSTRUM spect
PROBHD 5 mm CPQCI 1H-
PULPROG zg30
TD 65536
SOLVENT CDCl3
NS 120
DS 2
SWH 10504.202 Hz
FIDRES 0.160281 Hz
AQ 3.1195135 sec
RG 27.53
DW 47.600 usec
DE 20.00 usec
TE 298.0 K
D1 1.00000000 sec
TD0 1
======== CHANNEL f1 ========
SFO1 700.3632917 MHz
NUC1 1H
P1 8.15 usec
PLW1 9.89999962 W
F2 - Processing parameters
SI 65536
SF 700.3600158 MHz
WDW EM
SSB 0
LB 0.30 Hz
GB 0
PC 1.00
9
8
7
6
5
4
3
2
1
0
ppm
0.95
1.98
2.10
1.07
1.00
2.07
1.14
1.49
1.02
0.84
3.23

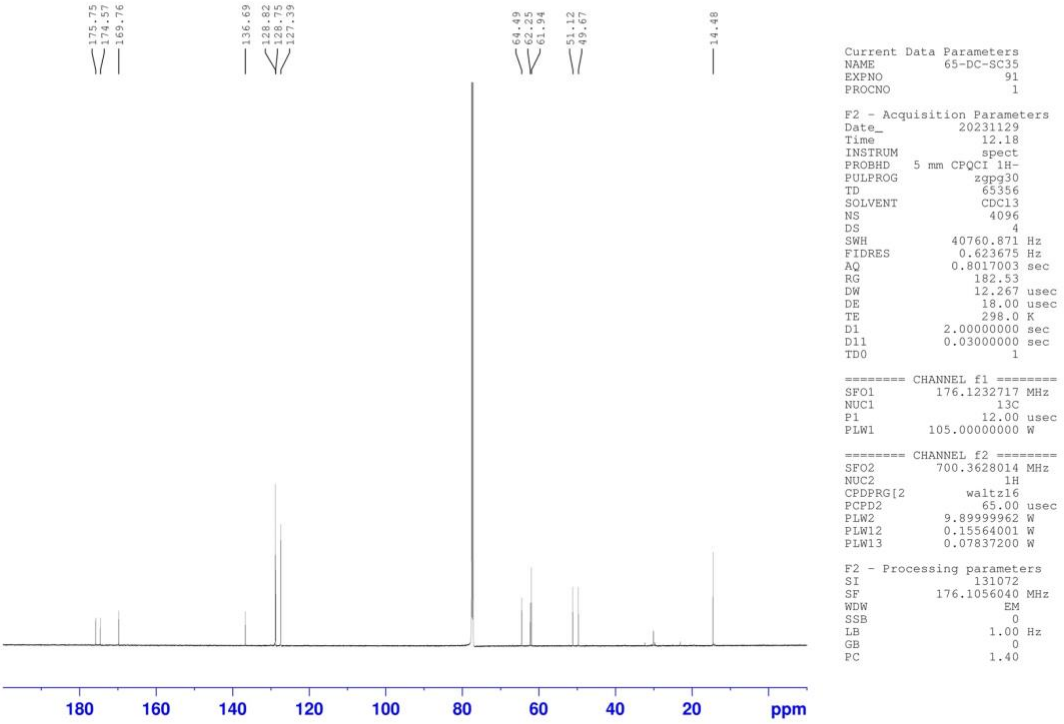
175.75
174.57
169.76
136.69
128.82
128.75
127.39
64.49
62.25
61.94
51.12
49.67
14.48
Current Data Parameters
NAME 65-DC-SC35
EXPNO 91
PROCNO 1
F2 - Acquisition Parameters
Date_ 20231129
Time 12.18
INSTRUM spect
PROBHD 5 mm CPQCI 1H-
PULPROG zgpg30
TD 65356
SOLVENT CDCl3
NS 4096
DS 4
SWH 40760.871 Hz
FIDRES 0.623675 Hz
AQ 0.8017003 sec
RG 182.53
DW 12.267 usec
DE 18.00 usec
TE 298.0 K
D1 2.00000000 sec
D11 0.03000000 sec
TD0 1
======== CHANNEL f1 ========
SFO1 176.1232717 MHz
NUC1 13C
P1 12.00 usec
PLW1 105.00000000 W
======== CHANNEL f2 ========
SFO2 700.3628014 MHz
NUC2 1H
CPDPRG[2 waltz16
PCPD2 65.00 usec
PLW2 9.89999962 W
PLW12 0.15564001 W
PLW13 0.07837200 W
F2 - Processing parameters
SI 131072
SF 176.1056040 MHz
WDW EM
SSB 0
LB 1.00 Hz
GB 0
PC 1.40
180
160
140
120
100
80
60
40
20
ppm

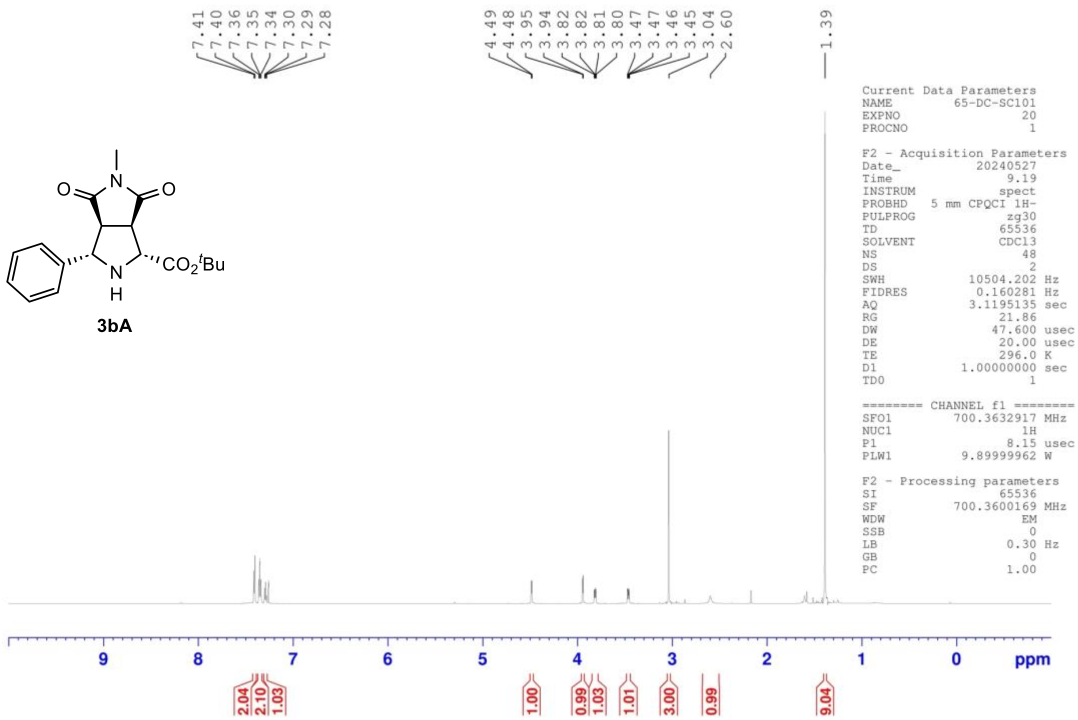
O
N
O
N
H
CO2tBu
3bA
7.41
7.40
7.36
7.35
7.34
7.30
7.29
7.28
4.49
4.48
3.95
3.94
3.82
3.82
3.81
3.80
3.47
3.47
3.46
3.45
3.04
2.60
1.39
Current Data Parameters
NAME 65-DC-SC101
EXPNO 20
PROCNO 1
F2 - Acquisition Parameters
Date_ 20240527
Time 9.19
INSTRUM spect
PROBHD 5 mm CPQCI 1H-
PULPROG zg30
TD 65536
SOLVENT CDCl3
NS 48
DS 2
SWH 10504.202 Hz
FIDRES 0.160281 Hz
AQ 3.1195135 sec
RG 21.86
DW 47.600 usec
DE 20.00 usec
TE 296.0 K
D1 1.00000000 sec
TD0 1
======== CHANNEL f1 ========
SFO1 700.3632917 MHz
NUC1 1H
P1 8.15 usec
PLW1 9.89999962 W
F2 - Processing parameters
SI 65536
SF 700.3600169 MHz
WDW EM
SSB 0
LB 0.30 Hz
GB 0
PC 1.00
9
8
7
6
5
4
3
2
1
0
ppm
2.04
2.10
1.03
1.00
0.99
1.03
1.01
3.00
0.99
9.04

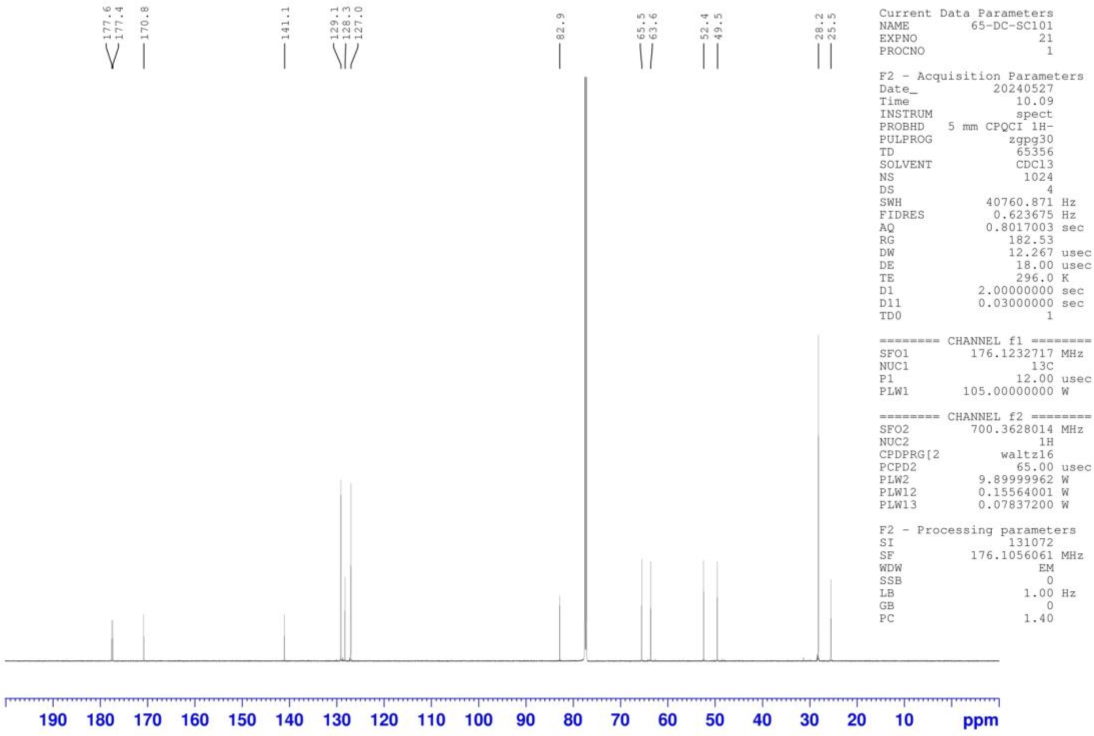
177.6
177.4
170.8
141.1
129.1
128.3
127.0
82.9
65.5
63.6
52.4
49.5
28.2
25.5
Current Data Parameters
NAME 65-DC-SC101
EXPNO 21
PROCNO 1
F2 - Acquisition Parameters
Date_ 20240527
Time 10.09
INSTRUM spect
PROBHD 5 mm CPQCI 1H-
PULPROG zgpg30
TD 65356
SOLVENT CDCl3
NS 1024
DS 4
SWH 40760.871 Hz
FIDRES 0.623675 Hz
AQ 0.8017003 sec
RG 182.53
DW 12.267 usec
DE 18.00 usec
TE 296.0 K
D1 2.00000000 sec
D11 0.03000000 sec
TD0 1
======== CHANNEL f1 ========
SFO1 176.1232717 MHz
NUC1 13C
P1 12.00 usec
PLW1 105.00000000 W
======== CHANNEL f2 ========
SFO2 700.3628014 MHz
NUC2 1H
CPDPRG[2 waltz16
PCPD2 65.00 usec
PLW2 9.89999962 W
PLW12 0.15564001 W
PLW13 0.07837200 W
F2 - Processing parameters
SI 131072
SF 176.1056061 MHz
WDW EM
SSB 0
LB 1.00 Hz
GB 0
PC 1.40
190
180
170
160
150
140
130
120
110
100
90
80
70
60
50
40
30
20
10
ppm

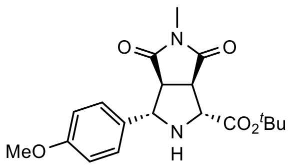

3kA

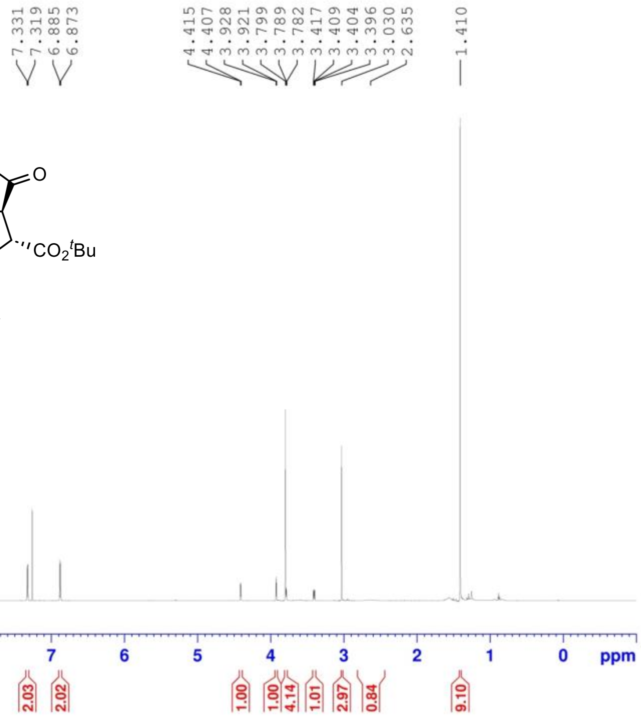


```
Current Data Parameters
NAME              65-DC-SC99
EXPNO                     70
PROCNO                     1

F2 - Acquisition Parameters
Date_               20231202
Time                   12.50
INSTRUM                spect
PROBHD   5 mm CPQCI 1H-
PULPROG                 zg30
TD                     65536
SOLVENT                CDCl3
NS                       128
DS                         2
SWH            10504.202 Hz
FIDRES          0.160281 Hz
AQ             3.1195135 sec
RG                     27.53
DW                47.600 usec
DE                 20.00 usec
TE                 298.0 K
D1            1.00000000 sec
TD0                        1

======== CHANNEL f1 ========
SFO1         700.3632917 MHz
NUC1                      1H
P1                  8.15 usec
PLW1           9.89999962 W

F2 - Processing parameters
SI                     65536
SF           700.3600158 MHz
WDW                       EM
SSB                        0
LB                   0.30 Hz
GB                         0
PC                      1.00
```

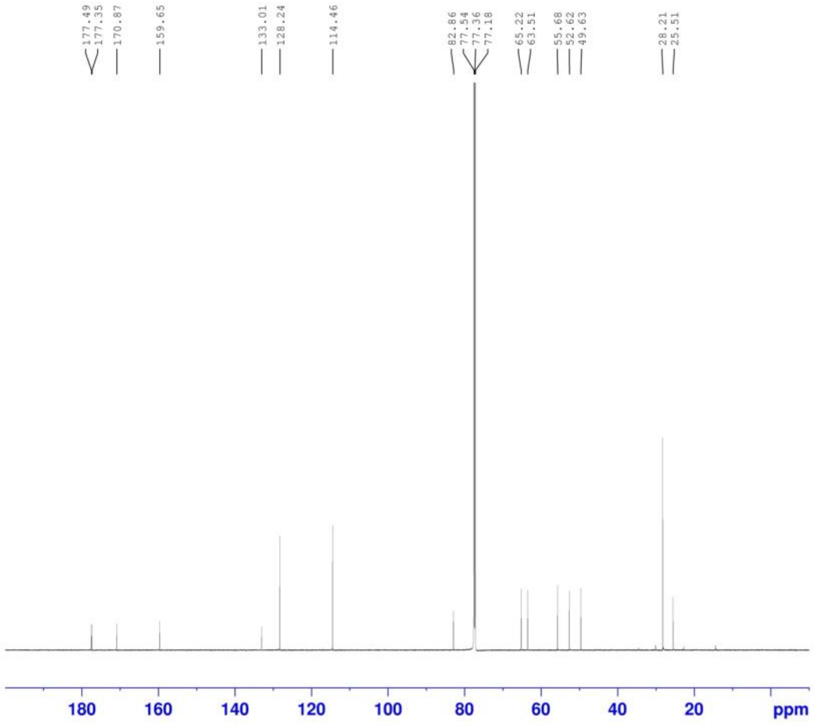


```
Current Data Parameters
NAME              65-DC-SC99
EXPNO                     71
PROCNO                     1

F2 - Acquisition Parameters
Date_               20231202
Time                   16.07
INSTRUM                spect
PROBHD   5 mm CPQCI 1H-
PULPROG              zgpg30
TD                     65356
SOLVENT                CDCl3
NS                      4096
DS                         4
SWH            40760.871 Hz
FIDRES          0.623675 Hz
AQ             0.8017003 sec
RG                    182.53
DW                12.267 usec
DE                 18.00 usec
TE                 298.0 K
D1            2.00000000 sec
D11           0.03000000 sec
TD0                        1

======== CHANNEL f1 ========
SFO1         176.1232717 MHz
NUC1                     13C
P1                 12.00 usec
PLW1        105.00000000 W

======== CHANNEL f2 ========
SFO2         700.3628014 MHz
NUC2                      1H
CPDPRG[2             waltz16
PCPD2              65.00 usec
PLW2           9.89999962 W
PLW12          0.15564001 W
PLW13          0.07837200 W

F2 - Processing parameters
SI                    131072
SF           176.1056030 MHz
WDW                       EM
SSB                        0
LB                   1.00 Hz
GB                         0
PC                      1.40
```

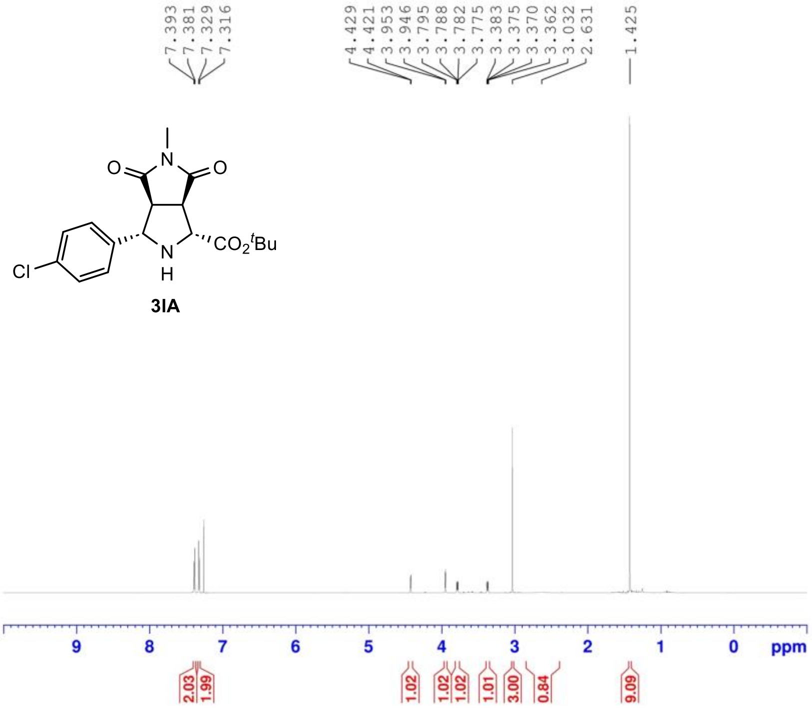


```
Current Data Parameters
NAME            65-DC-SC88
EXPNO                   80
PROCNO                   1

F2 - Acquisition Parameters
Date_             20231202
Time                 21.47
INSTRUM              spect
PROBHD   5 mm CPQCI 1H-
PULPROG               zg30
TD                   65536
SOLVENT              CDCl3
NS                     128
DS                       2
SWH              10504.202 Hz
FIDRES            0.160281 Hz
AQ               3.1195135 sec
RG                   21.86
DW                  47.600 usec
DE                   20.00 usec
TE                   298.0 K
D1              1.00000000 sec
TD0                      1

======== CHANNEL f1 ========
SFO1           700.3632917 MHz
NUC1                    1H
P1                    8.15 usec
PLW1            9.89999962 W

F2 - Processing parameters
SI                   65536
SF             700.3600157 MHz
WDW                     EM
SSB                      0
LB                    0.30 Hz
GB                       0
PC                    1.00
```

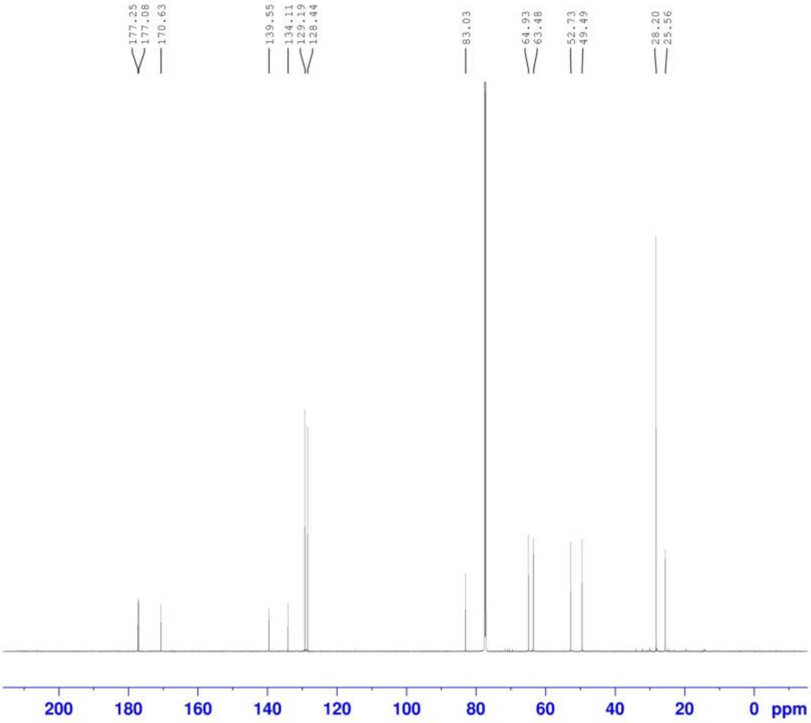


```
Current Data Parameters
NAME            65-DC-SC88
EXPNO                   81
PROCNO                   1

F2 - Acquisition Parameters
Date_             20231203
Time                  1.04
INSTRUM              spect
PROBHD   5 mm CPQCI 1H-
PULPROG             zgpg30
TD                   65356
SOLVENT              CDCl3
NS                    4096
DS                       4
SWH              40760.871 Hz
FIDRES            0.623675 Hz
AQ               0.8017003 sec
RG                  182.53
DW                  12.267 usec
DE                   18.00 usec
TE                   298.0 K
D1              2.00000000 sec
D11             0.03000000 sec
TD0                      1

======== CHANNEL f1 ========
SFO1           176.1232717 MHz
NUC1                   13C
P1                   12.00 usec
PLW1          105.00000000 W

======== CHANNEL f2 ========
SFO2           700.3628014 MHz
NUC2                    1H
CPDPRG[2           waltz16
PCPD2                65.00 usec
PLW2            9.89999962 W
PLW12           0.15564001 W
PLW13           0.07837200 W

F2 - Processing parameters
SI                  131072
SF             176.1056041 MHz
WDW                     EM
SSB                      0
LB                    1.00 Hz
GB                       0
PC                    1.40
```

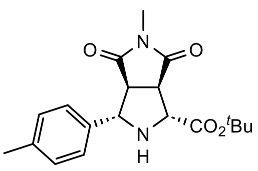

3mA

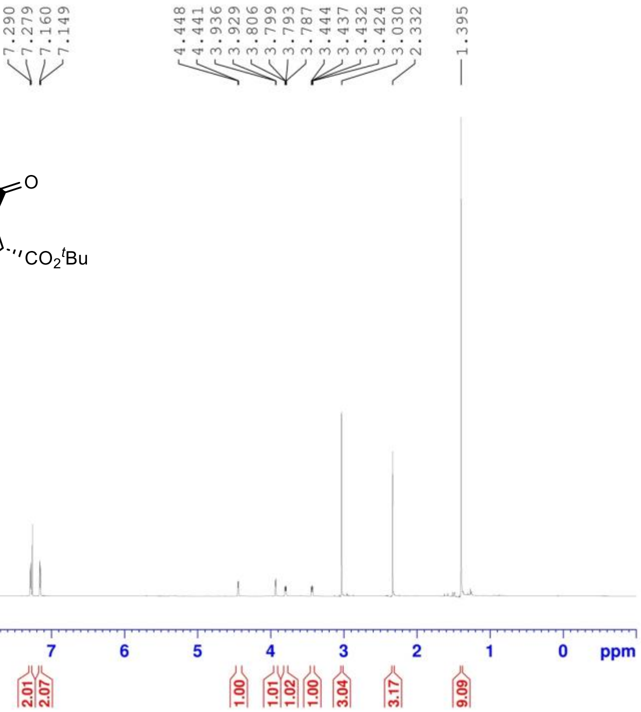


```
Current Data Parameters
NAME          65-DC-SC93
EXPNO                 90
PROCNO                 1

F2 - Acquisition Parameters
Date_           20231203
Time                6.44
INSTRUM            spect
PROBHD   5 mm CPQCI 1H-
PULPROG             zg30
TD                 65536
SOLVENT            CDCl3
NS                   128
DS                     2
SWH            10504.202 Hz
FIDRES          0.160281 Hz
AQ             3.1195135 sec
RG                  20.5
DW                47.600 usec
DE                 20.00 usec
TE                 298.0 K
D1            1.00000000 sec
TD0                    1

======== CHANNEL f1 ========
SFO1         700.3632917 MHz
NUC1                  1H
P1                  8.15 usec
PLW1          9.89999962 W

F2 - Processing parameters
SI                 65536
SF           700.3600157 MHz
WDW                   EM
SSB                    0
LB                  0.30 Hz
GB                     0
PC                  1.00
```

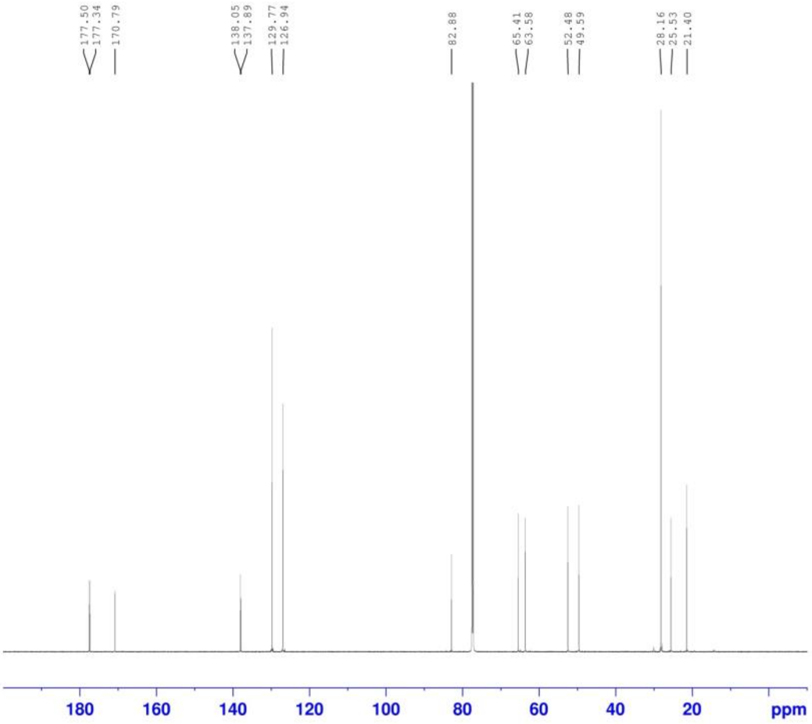


```
Current Data Parameters
NAME          65-DC-SC93
EXPNO                 91
PROCNO                 1

F2 - Acquisition Parameters
Date_           20231203
Time               10.01
INSTRUM            spect
PROBHD   5 mm CPQCI 1H-
PULPROG          zgpg30
TD                 65356
SOLVENT            CDCl3
NS                  4096
DS                     4
SWH            40760.871 Hz
FIDRES          0.623675 Hz
AQ             0.8017003 sec
RG                182.53
DW                12.267 usec
DE                 18.00 usec
TE                 298.0 K
D1            2.00000000 sec
D11           0.03000000 sec
TD0                    1

======== CHANNEL f1 ========
SFO1         176.1232717 MHz
NUC1                 13C
P1                 12.00 usec
PLW1        105.00000000 W

======== CHANNEL f2 ========
SFO2         700.3628014 MHz
NUC2                  1H
CPDPRG[2          waltz16
PCPD2              65.00 usec
PLW2          9.89999962 W
PLW12         0.15564001 W
PLW13         0.07837200 W

F2 - Processing parameters
SI                131072
SF           176.1056048 MHz
WDW                   EM
SSB                    0
LB                  1.00 Hz
GB                     0
PC                  1.40
```

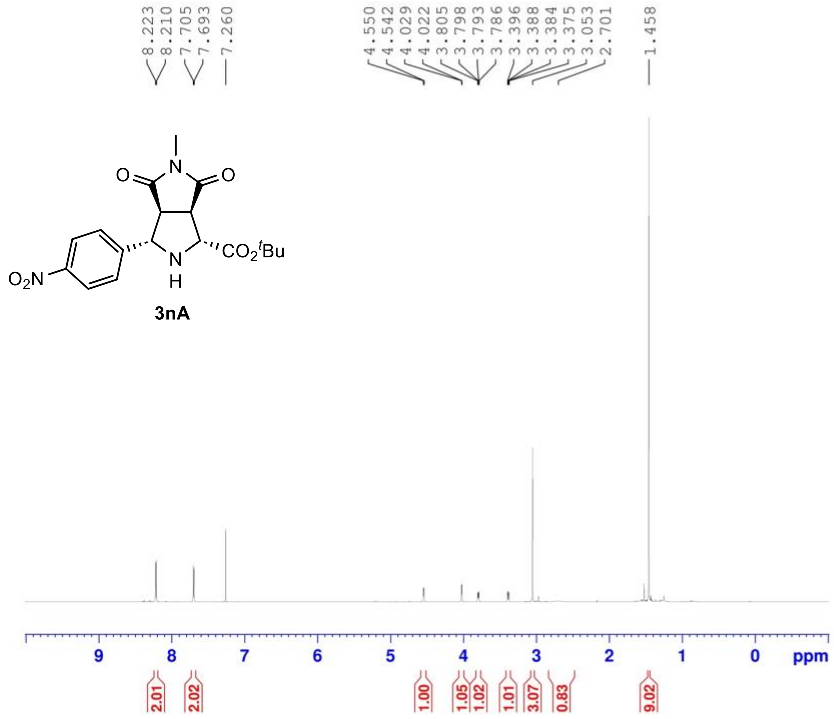


```
Current Data Parameters
NAME            65-DC-SC97
EXPNO                   70
PROCNO                   1

F2 - Acquisition Parameters
Date_             20231130
Time                 18.10
INSTRUM              spect
PROBHD   5 mm CPQCI 1H-
PULPROG               zg30
TD                   65536
SOLVENT              CDCl3
NS                     128
DS                       2
SWH              10504.202 Hz
FIDRES            0.160281 Hz
AQ               3.1195135 sec
RG                   21.86
DW                  47.600 usec
DE                   20.00 usec
TE                   298.0 K
D1              1.00000000 sec
TD0                      1

======== CHANNEL f1 ========
SFO1           700.3632917 MHz
NUC1                    1H
P1                    8.15 usec
PLW1            9.89999962 W

F2 - Processing parameters
SI                   65536
SF             700.3600157 MHz
WDW                     EM
SSB                      0
LB                    0.30 Hz
GB                       0
PC                    1.00
```

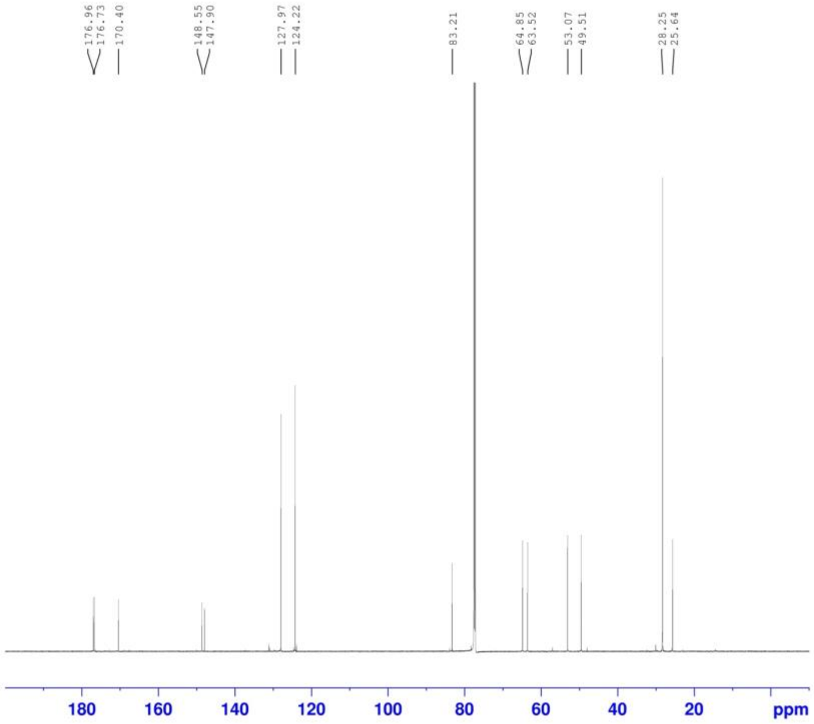


```
Current Data Parameters
NAME            65-DC-SC97
EXPNO                   71
PROCNO                   1

F2 - Acquisition Parameters
Date_             20231130
Time                 21.27
INSTRUM              spect
PROBHD   5 mm CPQCI 1H-
PULPROG             zgpg30
TD                   65356
SOLVENT              CDCl3
NS                    4096
DS                       4
SWH              40760.871 Hz
FIDRES            0.623675 Hz
AQ               0.8017003 sec
RG                  182.53
DW                  12.267 usec
DE                   18.00 usec
TE                   298.0 K
D1              2.00000000 sec
D11             0.03000000 sec
TD0                      1

======== CHANNEL f1 ========
SFO1           176.1232717 MHz
NUC1                    13C
P1                   12.00 usec
PLW1          105.00000000 W

======== CHANNEL f2 ========
SFO2           700.3628014 MHz
NUC2                    1H
CPDPRG[2            waltz16
PCPD2                65.00 usec
PLW2            9.89999962 W
PLW12           0.15564001 W
PLW13           0.07837200 W

F2 - Processing parameters
SI                  131072
SF             176.1056043 MHz
WDW                     EM
SSB                      0
LB                    1.00 Hz
GB                       0
PC                    1.40
```

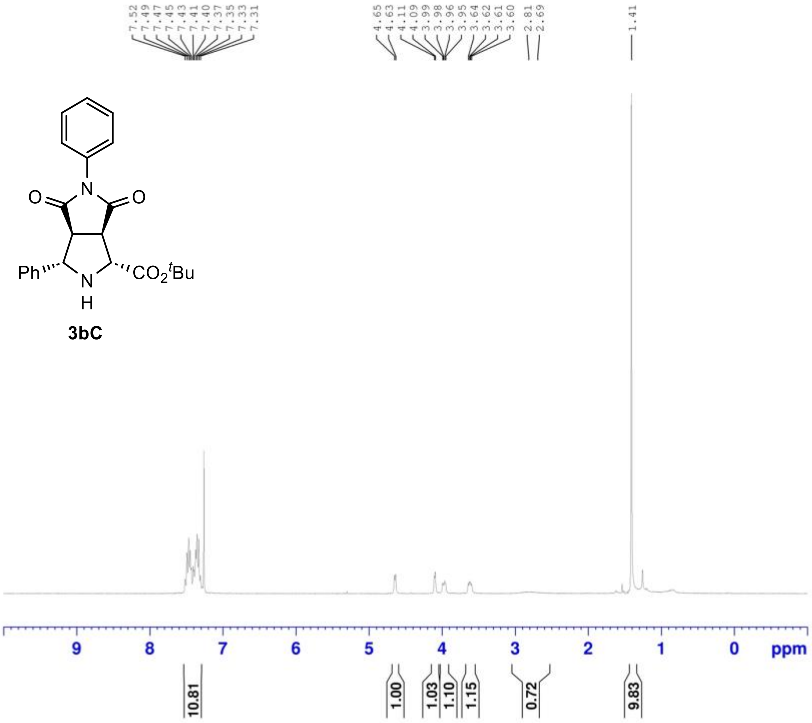


```
Current Data Parameters
NAME            65-DC-SC094
EXPNO                   200
PROCNO                    1

F2 - Acquisition Parameters
Date_              20231129
Time                  13.47
INSTRUM               spect
PROBHD   5 mm PABBO BB-
PULPROG                zg30
TD                    32768
SOLVENT               CDCl3
NS                       16
DS                        2
SWH                6188.119 Hz
FIDRES             0.188846 Hz
AQ                2.6476543 sec
RG                      456
DW                   80.800 usec
DE                     8.00 usec
TE                    296.0 K
D1               1.00000000 sec
TD0                       1

======== CHANNEL f1 ========
NUC1                     1H
P1                    11.05 usec
PL1                   -2.00 dB
PL1W            37.02396774 W
SFO1            300.1318534 MHz

F2 - Processing parameters
SI                    16384
SF              300.1300061 MHz
WDW                      EM
SSB                       0
LB                     0.30 Hz
GB                        0
PC                     1.00
```

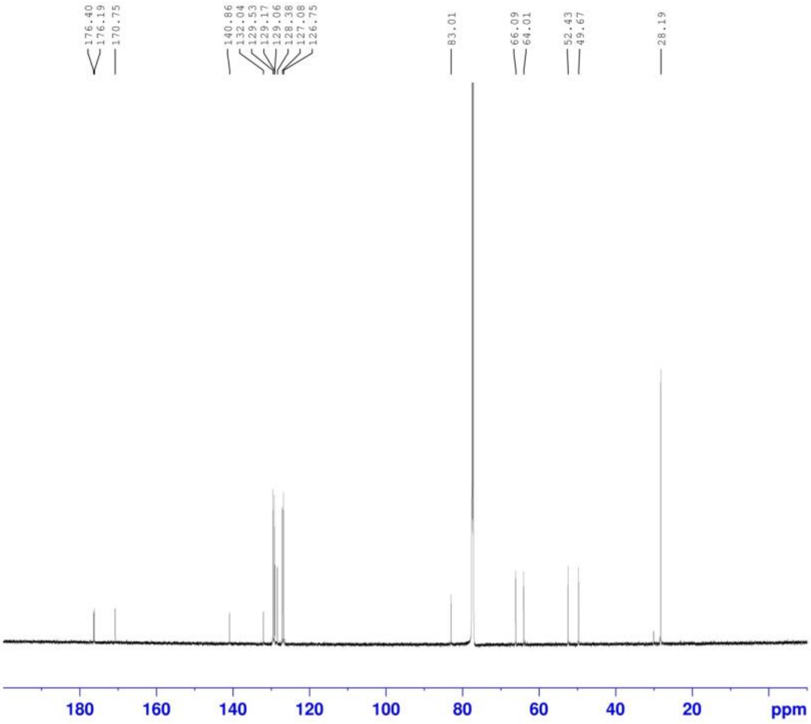


```
Current Data Parameters
NAME             65-DC-SC94
EXPNO                   101
PROCNO                    1

F2 - Acquisition Parameters
Date_              20231203
Time                  18.55
INSTRUM               spect
PROBHD   5 mm CPQCI 1H-
PULPROG             zgpg30
TD                    65356
SOLVENT               CDCl3
NS                     4096
DS                        4
SWH               40760.871 Hz
FIDRES             0.623675 Hz
AQ                0.8017003 sec
RG                   182.53
DW                   12.267 usec
DE                    18.00 usec
TE                    298.0 K
D1               2.00000000 sec
D11              0.03000000 sec
TD0                       1

======== CHANNEL f1 ========
SFO1            176.1232717 MHz
NUC1                    13C
P1                    12.00 usec
PLW1           105.00000000 W

======== CHANNEL f2 ========
SFO2            700.3628014 MHz
NUC2                     1H
CPDPRG[2            waltz16
PCPD2                 65.00 usec
PLW2             9.89999962 W
PLW12            0.15564001 W
PLW13            0.07837200 W

F2 - Processing parameters
SI                   131072
SF              176.1056023 MHz
WDW                      EM
SSB                       0
LB                     1.00 Hz
GB                        0
PC                     1.40
```

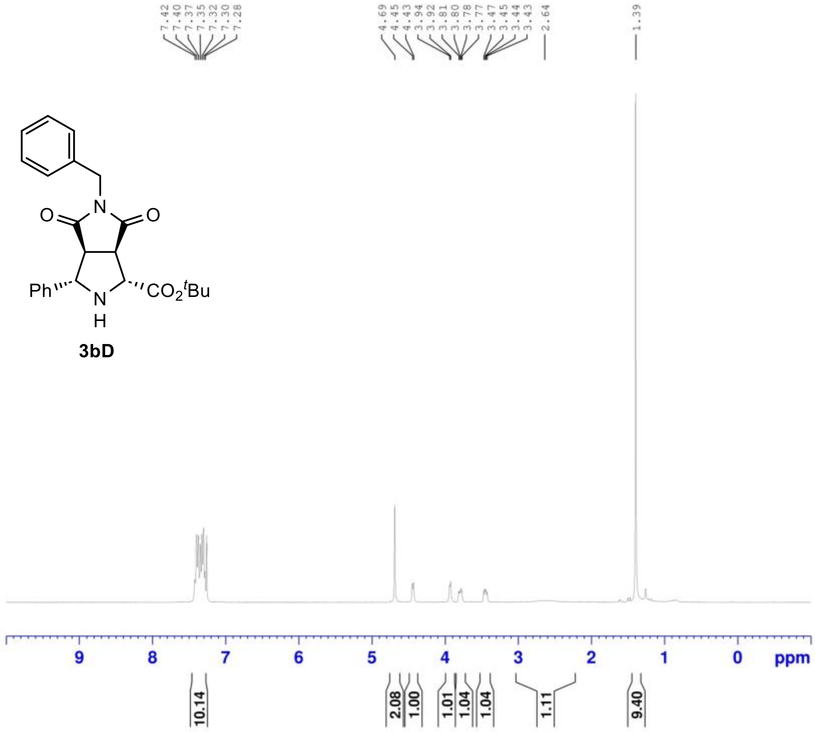

Current Data Parameters
NAME 65-DC-SC096
EXPNO 230
PROCNO 1

F2 - Acquisition Parameters
Date_ 20231127
Time 15.12
INSTRUM spect
PROBHD 5 mm PABBO BB-
PULPROG zg30
TD 32768
SOLVENT CDCl3
NS 16
DS 2
SWH 6188.119 Hz
FIDRES 0.188846 Hz
AQ 2.6476543 sec
RG 287
DW 80.800 usec
DE 8.00 usec
TE 296.0 K
D1 1.00000000 sec
TD0 1

======== CHANNEL f1 ========
NUC1 1H
P1 11.05 usec
PL1 -2.00 dB
PL1W 37.02396774 W
SFO1 300.1318534 MHz

F2 - Processing parameters
SI 16384
SF 300.1300061 MHz
WDW EM
SSB 0
LB 0.30 Hz
GB 0
PC 1.00

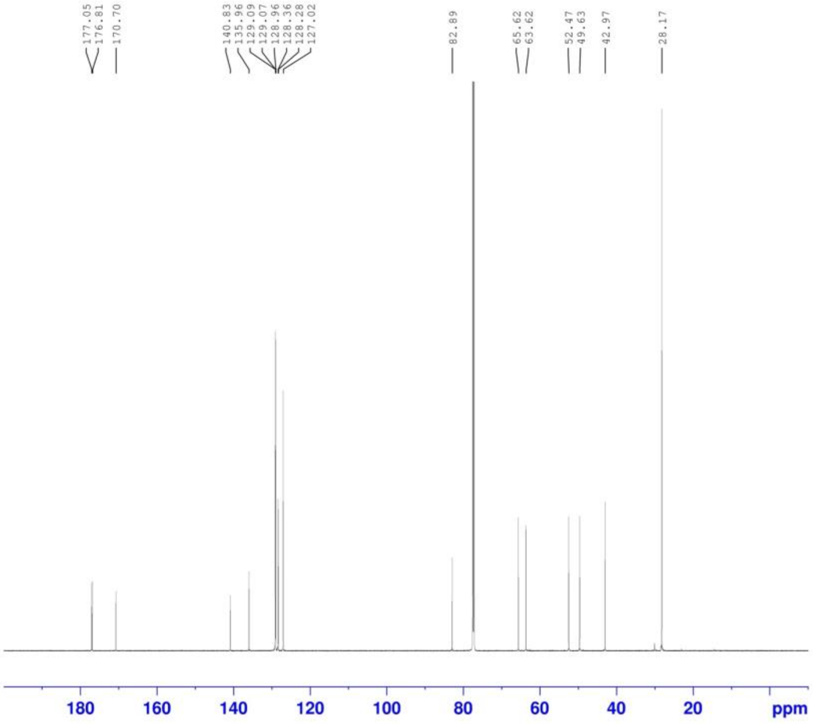

Current Data Parameters
NAME 65-DC-SC96
EXPNO 101
PROCNO 1

F2 - Acquisition Parameters
Date_ 20231129
Time 21.13
INSTRUM spect
PROBHD 5 mm CPQCI 1H-
PULPROG zgpg30
TD 65356
SOLVENT CDCl3
NS 4096
DS 4
SWH 40760.871 Hz
FIDRES 0.623675 Hz
AQ 0.8017003 sec
RG 182.53
DW 12.267 usec
DE 18.00 usec
TE 298.0 K
D1 2.00000000 sec
D11 0.03000000 sec
TD0 1

======== CHANNEL f1 ========
SFO1 176.1232717 MHz
NUC1 13C
P1 12.00 usec
PLW1 105.00000000 W

======== CHANNEL f2 ========
SFO2 700.3628014 MHz
NUC2 1H
CPDPRG[2 waltz16
PCPD2 65.00 usec
PLW2 9.89999962 W
PLW12 0.15564001 W
PLW13 0.07837200 W

F2 - Processing parameters
SI 131072
SF 176.1056062 MHz
WDW EM
SSB 0
LB 1.00 Hz
GB 0
PC 1.40

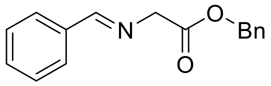
N
O
Bn
O

1d

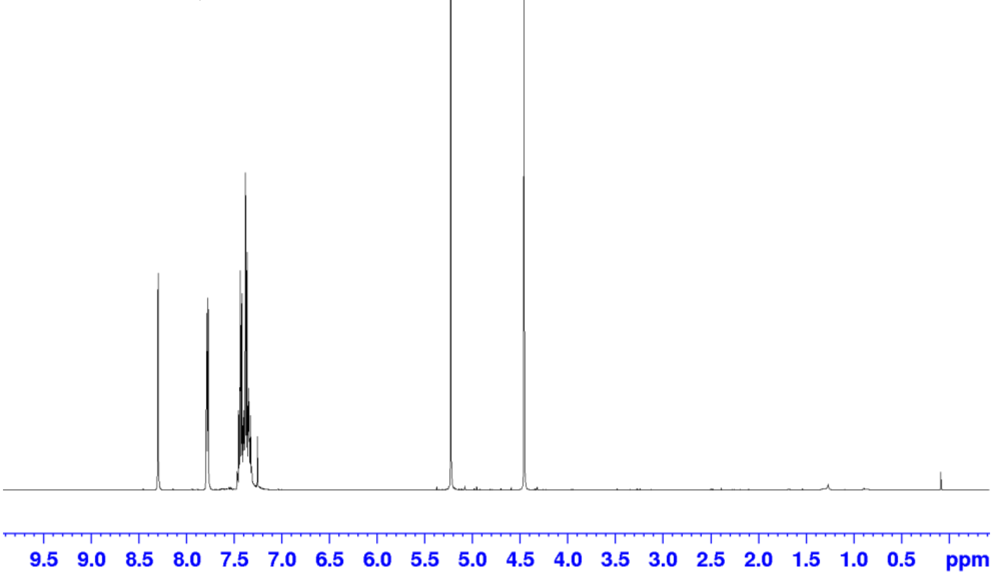
9.5 9.0 8.5 8.0 7.5 7.0 6.5 6.0 5.5 5.0 4.5 4.0 3.5 3.0 2.5 2.0 1.5 1.0 0.5 ppm

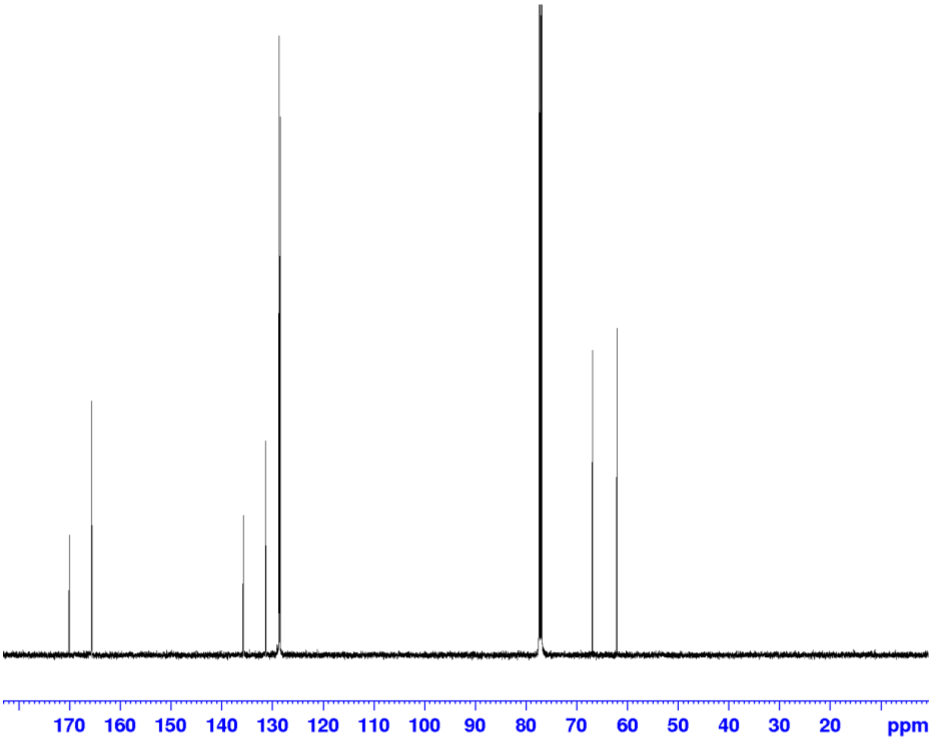
170 160 150 140 130 120 110 100 90 80 70 60 50 40 30 20 ppm

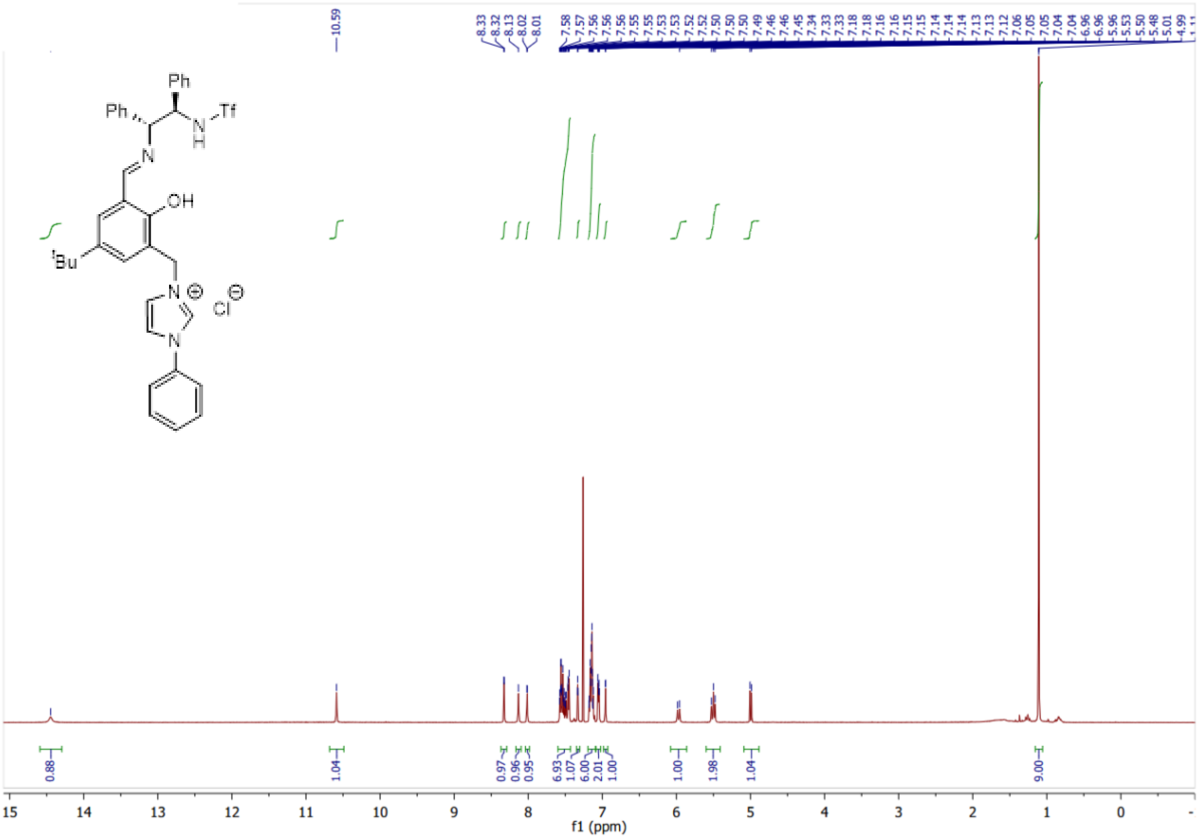

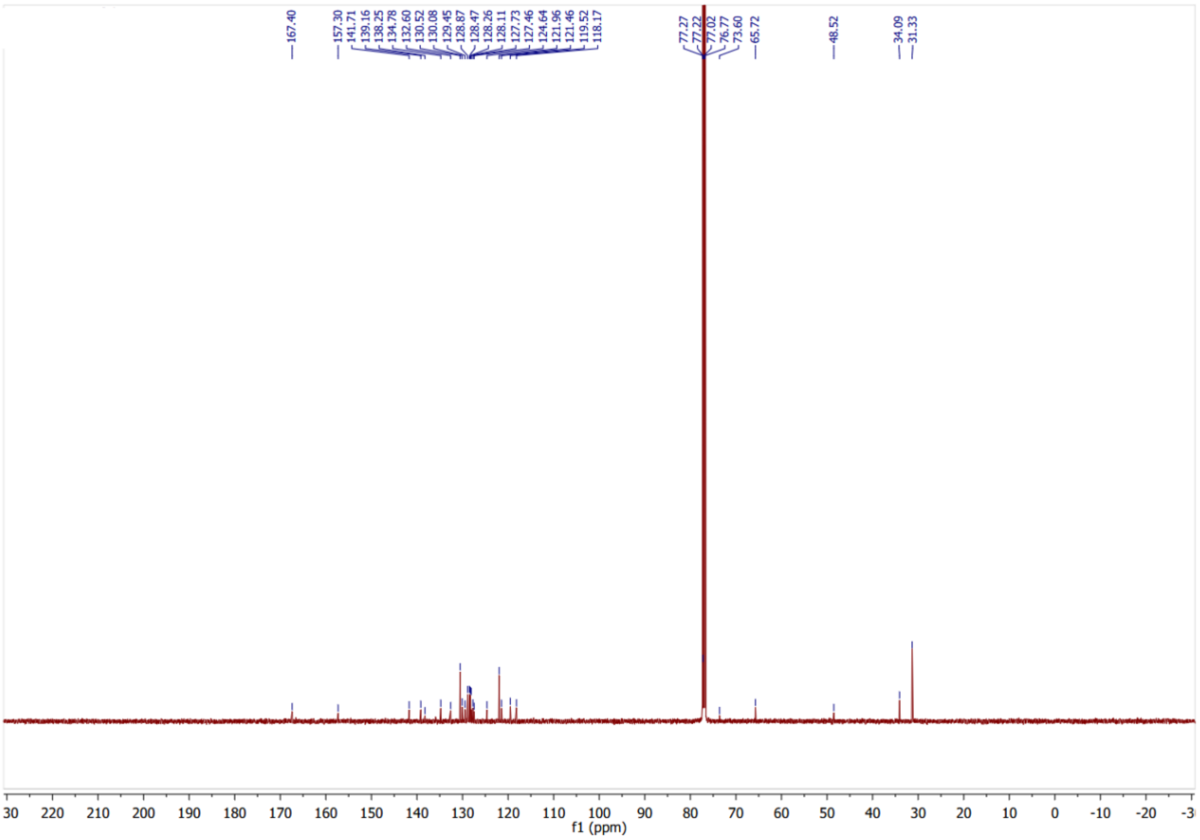

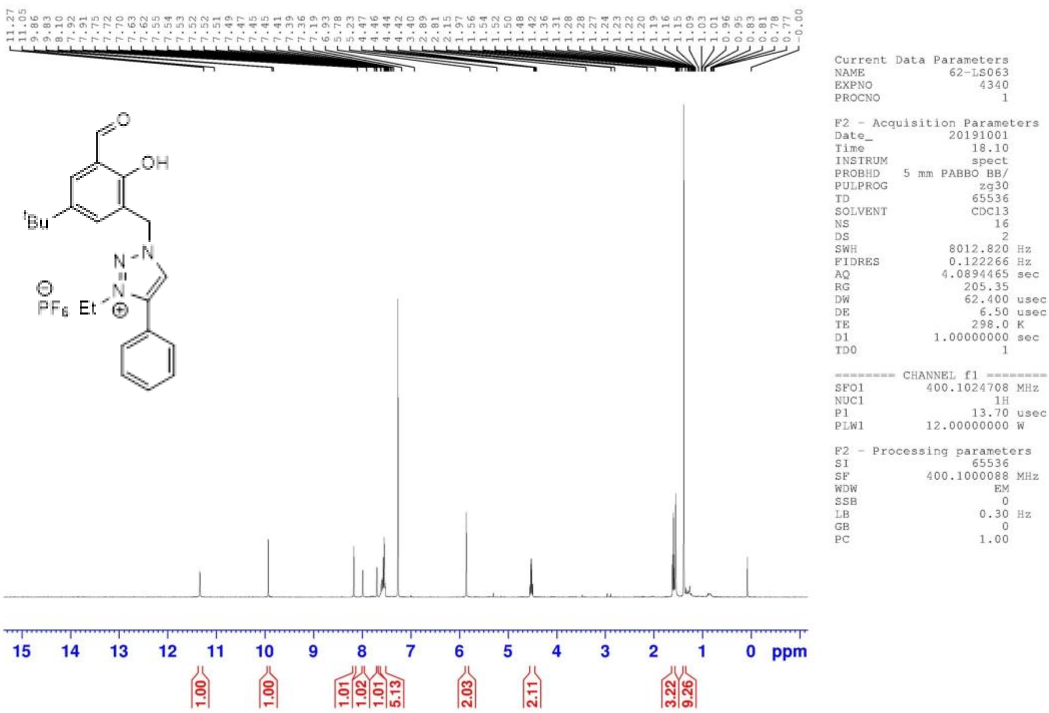
tBu
OH
N
PF6
Et
Current Data Parameters
NAME 62-LS063
EXPNO 4340
PROCNO 1
F2 - Acquisition Parameters
Date_ 20191001
Time 18.10
INSTRUM spect
PROBHD 5 mm PABBO BB/
PULPROG zg30
TD 65536
SOLVENT CDCl3
NS 16
DS 2
SWH 8012.820 Hz
FIDRES 0.122266 Hz
AQ 4.0894465 sec
RG 205.35
DW 62.400 usec
DE 6.50 usec
TE 298.0 K
D1 1.00000000 sec
TD0 1
======== CHANNEL f1 ========
SFO1 400.1024708 MHz
NUC1 1H
P1 13.70 usec
PLW1 12.00000000 W
F2 - Processing parameters
SI 65536
SF 400.1000088 MHz
WDW EM
SSB 0
LB 0.30 Hz
GB 0
PC 1.00
ppm

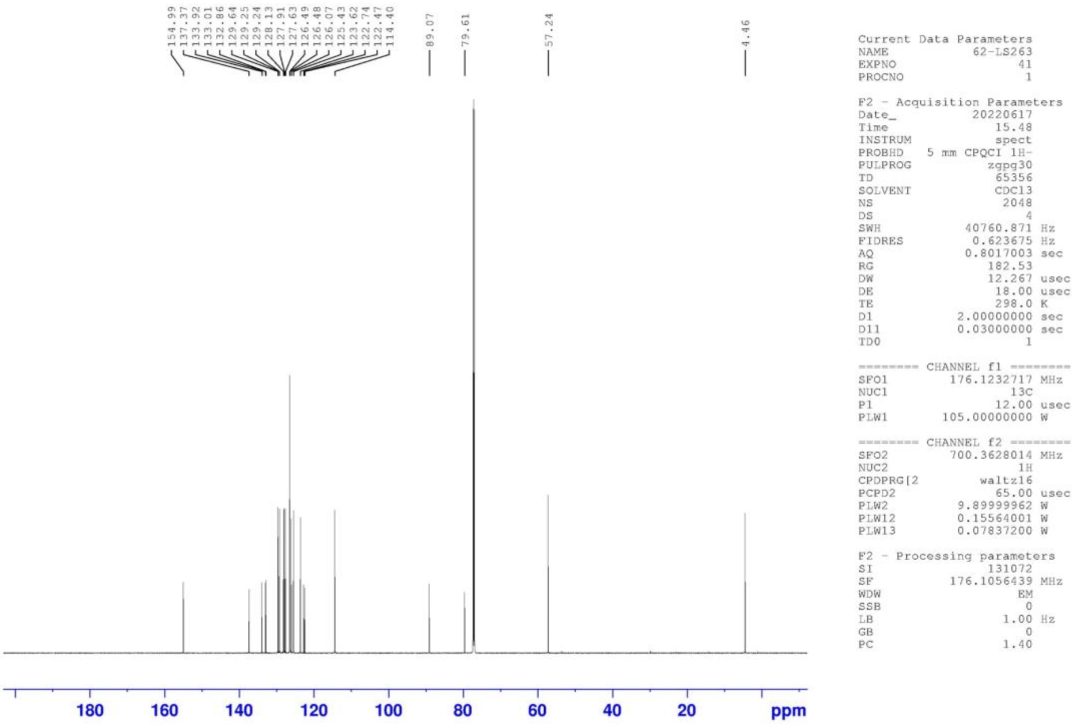
Current Data Parameters
NAME 62-LS263
EXPNO 41
PROCNO 1
F2 - Acquisition Parameters
Date_ 20220617
Time 15.48
INSTRUM spect
PROBHD 5 mm CPQCI 1H-
PULPROG zgpg30
TD 65356
SOLVENT CDCl3
NS 2048
DS 4
SWH 40760.871 Hz
FIDRES 0.623675 Hz
AQ 0.8017003 sec
RG 182.53
DW 12.267 usec
DE 18.00 usec
TE 298.0 K
D1 2.00000000 sec
D11 0.03000000 sec
TD0 1
======== CHANNEL f1 ========
SFO1 176.1232717 MHz
NUC1 13C
P1 12.00 usec
PLW1 105.00000000 W
======== CHANNEL f2 ========
SFO2 700.3628014 MHz
NUC2 1H
CPDPRG[2 waltz16
PCPD2 65.00 usec
PLW2 9.89999962 W
PLW12 0.15564001 W
PLW13 0.07837200 W
F2 - Processing parameters
SI 131072
SF 176.1056439 MHz
WDW EM
SSB 0
LB 1.00 Hz
GB 0
PC 1.40
ppm

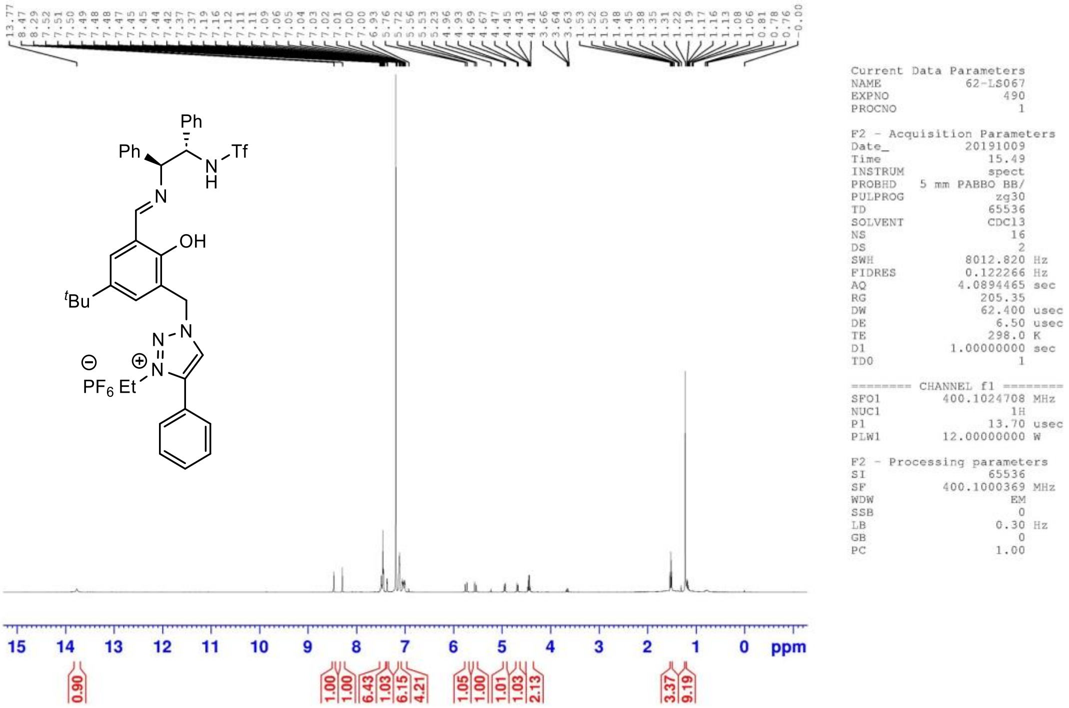

Ph
Ph
N
Tf
H
N
OH
tBu
N
N
N
PF6
Et
Current Data Parameters
NAME 62-LS067
EXPNO 490
PROCNO 1
F2 - Acquisition Parameters
Date_ 20191009
Time 15.49
INSTRUM spect
PROBHD 5 mm PABBO BB/
PULPROG zg30
TD 65536
SOLVENT CDCl3
NS 16
DS 2
SWH 8012.820 Hz
FIDRES 0.122266 Hz
AQ 4.0894465 sec
RG 205.35
DW 62.400 usec
DE 6.50 usec
TE 298.0 K
D1 1.00000000 sec
TD0 1
======== CHANNEL f1 ========
SFO1 400.1024708 MHz
NUC1 1H
P1 13.70 usec
PLW1 12.00000000 W
F2 - Processing parameters
SI 65536
SF 400.1000369 MHz
WDW EM
SSB 0
LB 0.30 Hz
GB 0
PC 1.00
15 14 13 12 11 10 9 8 7 6 5 4 3 2 1 0 ppm
0.90
1.00
1.00
6.43
1.03
6.15
4.21
1.05
1.00
1.01
1.03
2.13
3.37
9.19

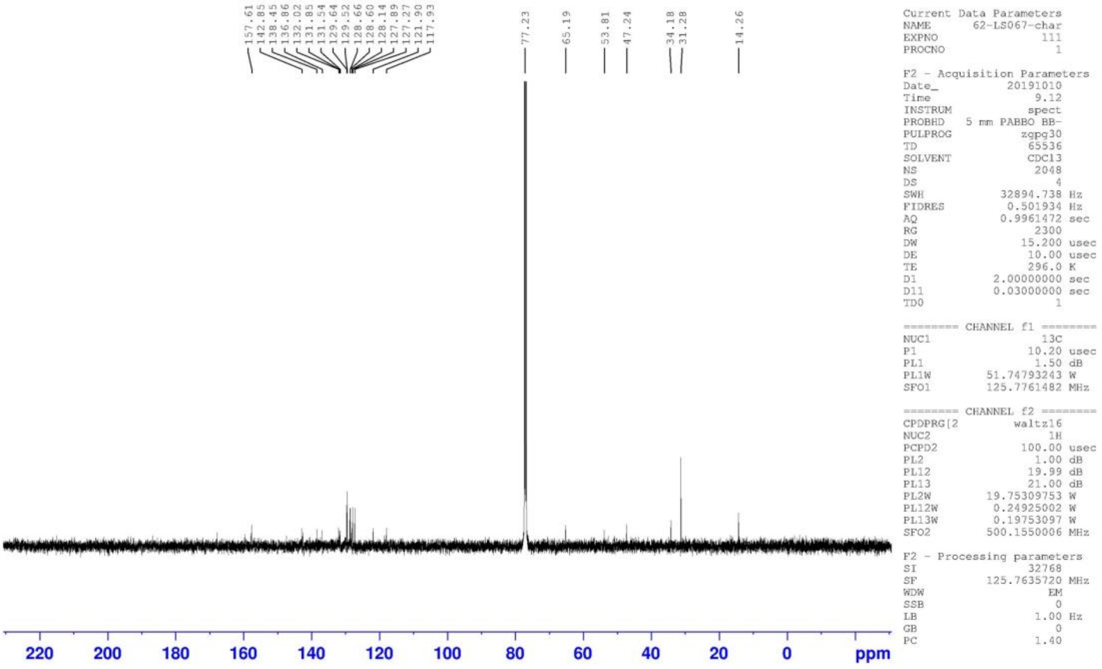

157.61
142.85
138.45
136.86
132.02
131.85
131.54
129.64
129.52
128.66
128.60
128.14
127.89
127.27
121.90
117.93
77.23
65.19
53.81
47.24
34.18
31.28
14.26
Current Data Parameters
NAME 62-LS067-char
EXPNO 111
PROCNO 1
F2 - Acquisition Parameters
Date_ 20191010
Time 9.12
INSTRUM spect
PROBHD 5 mm PABBO BB-
PULPROG zgpg30
TD 65536
SOLVENT CDCl3
NS 2048
DS 4
SWH 32894.738 Hz
FIDRES 0.501934 Hz
AQ 0.9961472 sec
RG 2300
DW 15.200 usec
DE 10.00 usec
TE 296.0 K
D1 2.00000000 sec
D11 0.03000000 sec
TD0 1
======== CHANNEL f1 ========
NUC1 13C
P1 10.20 usec
PL1 1.50 dB
PL1W 51.74793243 W
SFO1 125.7761482 MHz
======== CHANNEL f2 ========
CPDPRG[2 waltz16
NUC2 1H
PCPD2 100.00 usec
PL2 1.00 dB
PL12 19.99 dB
PL13 21.00 dB
PL2W 19.75309753 W
PL12W 0.24925002 W
PL13W 0.19753097 W
SFO2 500.1550006 MHz
F2 - Processing parameters
SI 32768
SF 125.7635720 MHz
WDW EM
SSB 0
LB 1.00 Hz
GB 0
PC 1.40
220 200 180 160 140 120 100 80 60 40 20 0 ppm

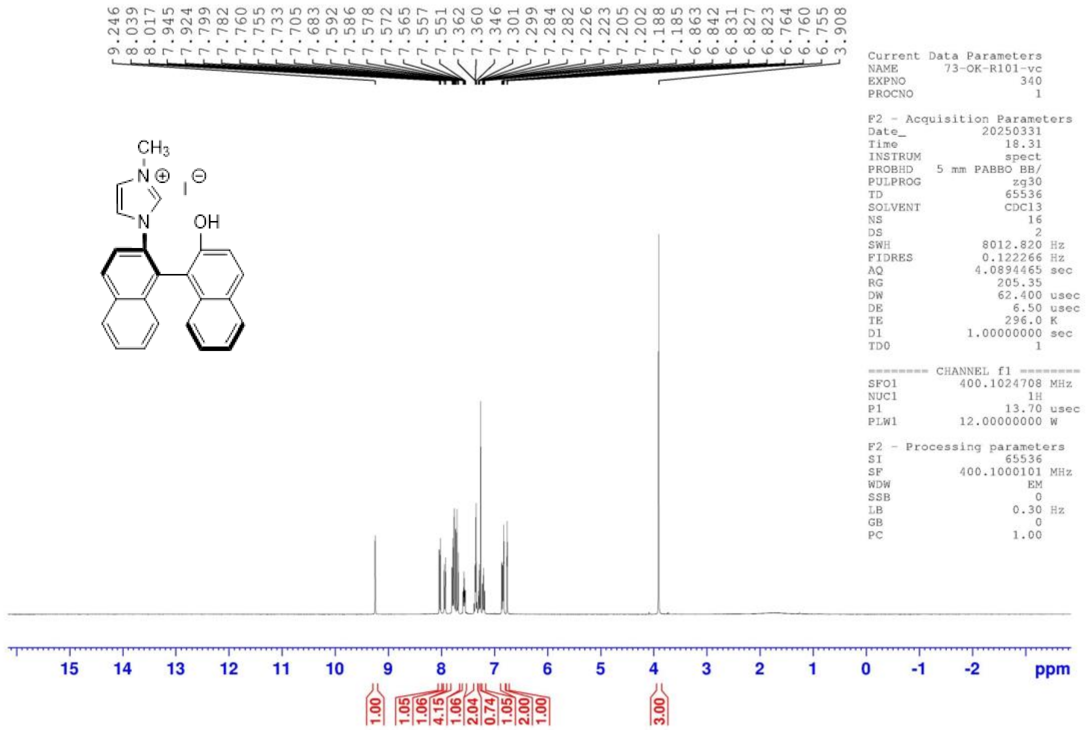
9.246 8.039 8.017 7.945 7.924 7.799 7.782 7.760 7.755 7.733 7.705 7.683 7.592 7.586 7.578 7.572 7.565 7.557 7.551 7.362 7.360 7.346 7.301 7.299 7.284 7.282 7.226 7.223 7.205 7.202 7.188 7.185 6.863 6.842 6.831 6.827 6.823 6.764 6.760 6.755 3.908
CH3
N⊕
I⊖
N
OH
Current Data Parameters
NAME 73-OK-R101-vc
EXPNO 340
PROCNO 1
F2 - Acquisition Parameters
Date_ 20250331
Time 18.31
INSTRUM spect
PROBHD 5 mm PABBO BB/
PULPROG zg30
TD 65536
SOLVENT CDCl3
NS 16
DS 2
SWH 8012.820 Hz
FIDRES 0.122266 Hz
AQ 4.0894465 sec
RG 205.35
DW 62.400 usec
DE 6.50 usec
TE 296.0 K
D1 1.00000000 sec
TD0 1
======== CHANNEL f1 ========
SFO1 400.1024708 MHz
NUC1 1H
P1 13.70 usec
PLW1 12.00000000 W
F2 - Processing parameters
SI 65536
SF 400.1000101 MHz
WDW EM
SSB 0
LB 0.30 Hz
GB 0
PC 1.00
15 14 13 12 11 10 9 8 7 6 5 4 3 2 1 0 -1 -2 ppm
1.00 1.05 1.06 4.15 1.06 2.04 0.74 1.05 2.00 1.00 3.00

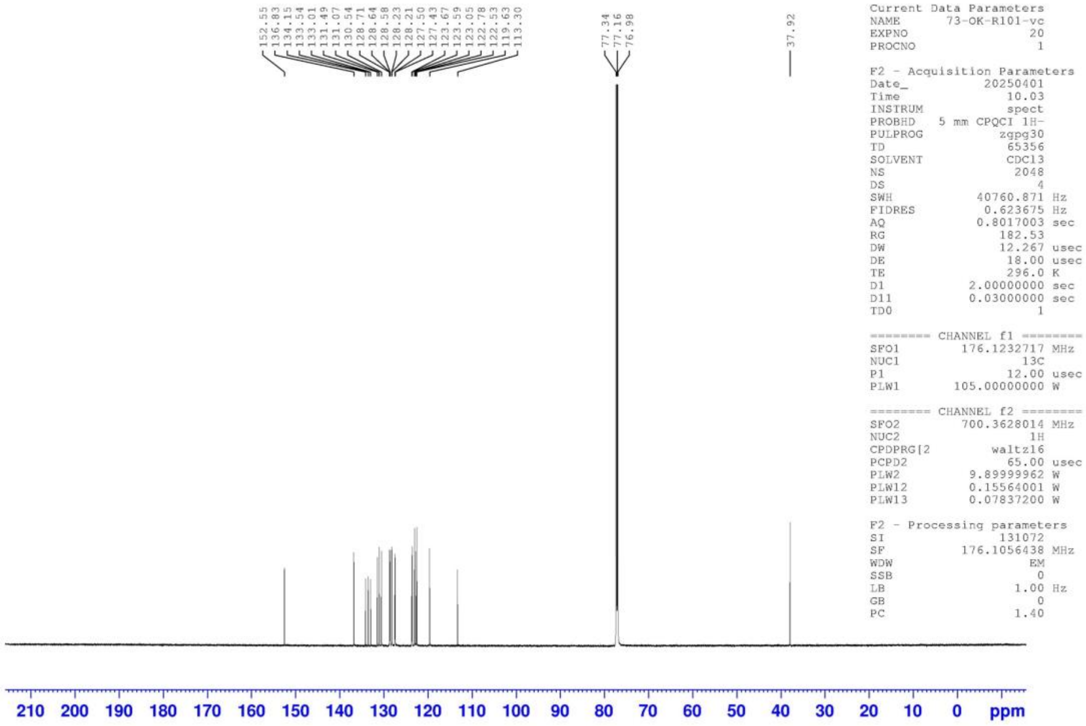
152.55 136.83 134.15 133.54 133.01 131.49 131.07 130.54 128.71 128.64 128.58 128.23 128.21 127.50 127.43 123.67 123.59 123.05 122.78 122.53 119.63 113.30
77.34 77.16 76.98
37.92
Current Data Parameters
NAME 73-OK-R101-vc
EXPNO 20
PROCNO 1
F2 - Acquisition Parameters
Date_ 20250401
Time 10.03
INSTRUM spect
PROBHD 5 mm CPQCI 1H-
PULPROG zgpg30
TD 65356
SOLVENT CDCl3
NS 2048
DS 4
SWH 40760.871 Hz
FIDRES 0.623675 Hz
AQ 0.8017003 sec
RG 182.53
DW 12.267 usec
DE 18.00 usec
TE 296.0 K
D1 2.00000000 sec
D11 0.03000000 sec
TD0 1
======== CHANNEL f1 ========
SFO1 176.1232717 MHz
NUC1 13C
P1 12.00 usec
PLW1 105.00000000 W
======== CHANNEL f2 ========
SFO2 700.3628014 MHz
NUC2 1H
CPDPRG[2 waltz16
PCPD2 65.00 usec
PLW2 9.89999962 W
PLW12 0.15564001 W
PLW13 0.07837200 W
F2 - Processing parameters
SI 131072
SF 176.1056438 MHz
WDW EM
SSB 0
LB 1.00 Hz
GB 0
PC 1.40
210 200 190 180 170 160 150 140 130 120 110 100 90 80 70 60 50 40 30 20 10 0 ppm

17 Chromatograms

3cA, IH, cyclohexane:^{i}PrOH (50:50), 1.0 mL/min, 220 nm

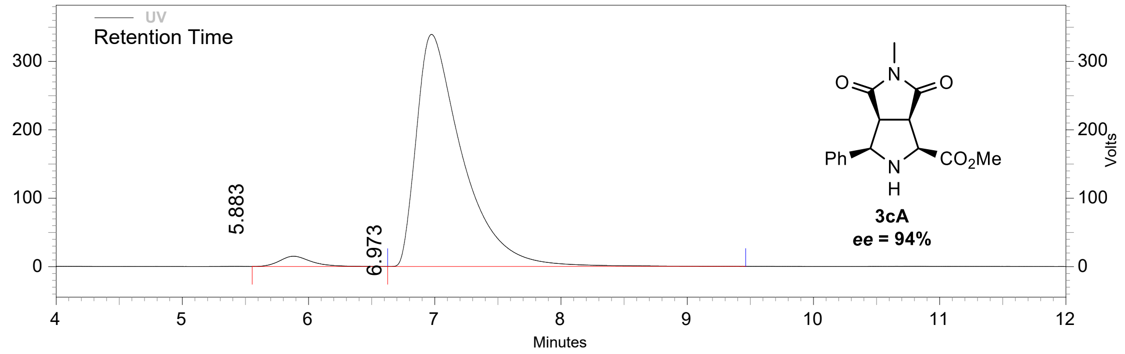

Retention Time	Height	Area	Area %
5.883	59712	1045840	2.96
6.973	1356982	34269802	97.04

3cA (rac.)

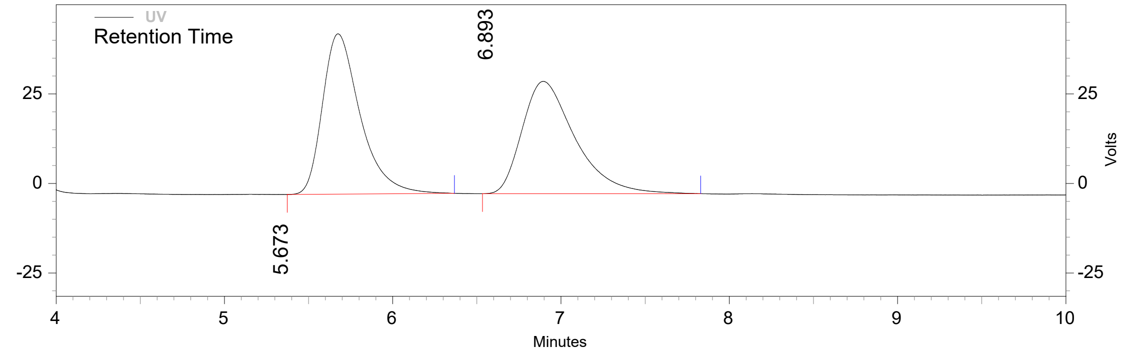

Retention Time	Height	Area	Area %
5.673	179085	2786873	50.37
6.893	125522	2745385	49.63

3aA, IH, cyclohexane:^{i}PrOH (50:50), 1.0 mL/min, 220 nm

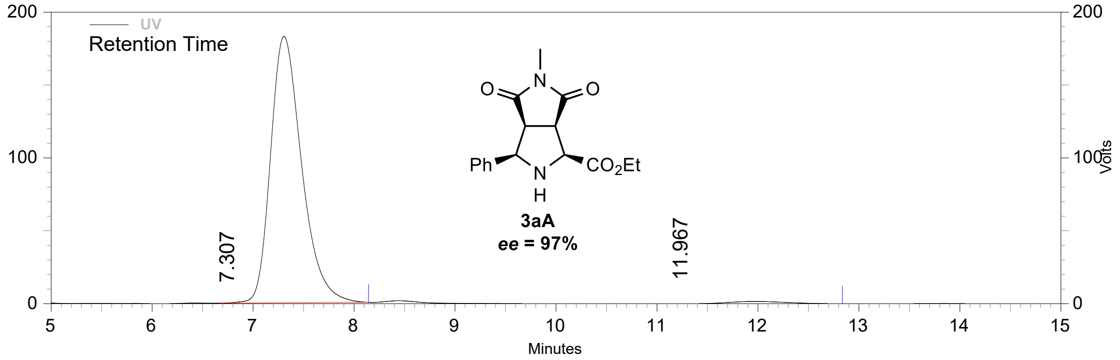

Retention Time	Height	Area	Area %
7.307	731391	15675844	98.40
11.967	6339	255570	1.60

3aA (rac.)

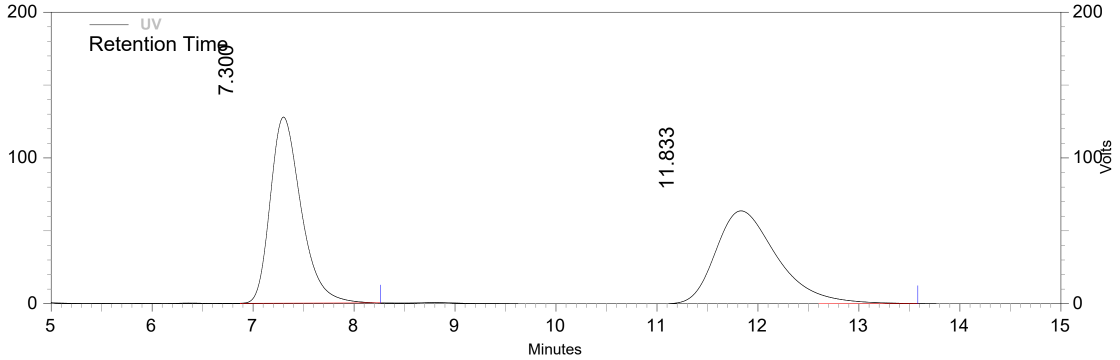

Retention Time	Height	Area	Area %
7.300	510593	10857630	50.18
11.833	255118	10779864	49.82

3dA, IA, cyclohexane:^{i}PrOH (52:48), 0.7 mL/min, 220 nm

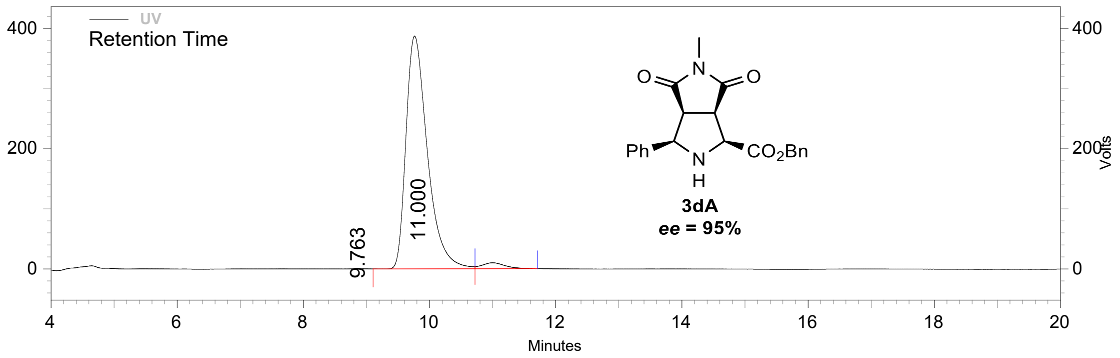

Retention Time	Height	Area	Area %
9.763	1550466	36597293	97.33
11.000	39663	1005345	2.67

3dA (rac.)

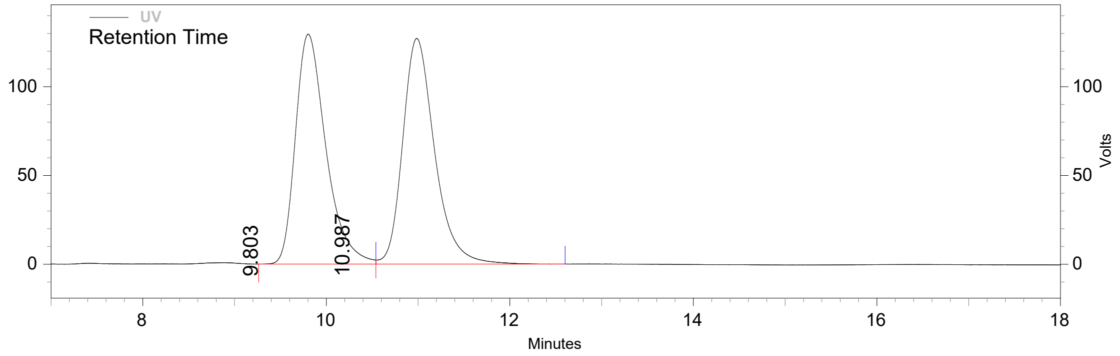

Retention Time	Height	Area	Area %
9.803	518231	11979266	49.40
10.987	508406	12269797	50.60

3eA, IH, cyclohexane:^{i}PrOH (50:50), 1.0 mL/min, 220 nm

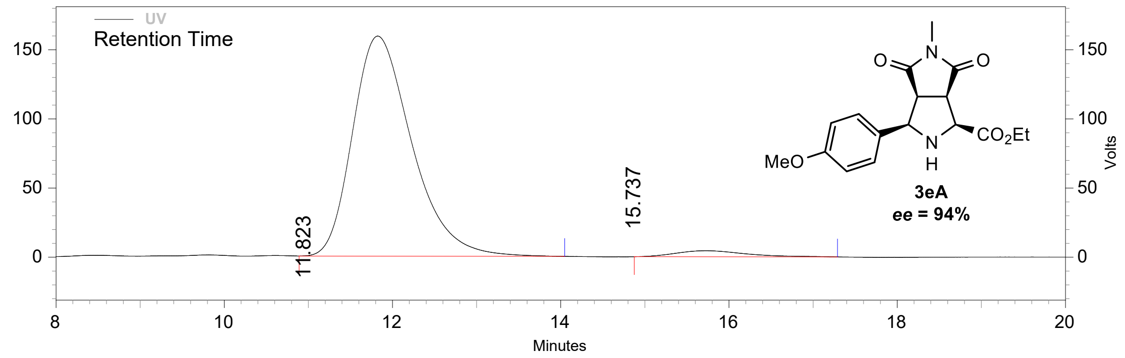

Retention Time	Height	Area	Area %
11.823	636499	31131880	96.85
15.737	17637	1012683	3.15

3eA (rac.)

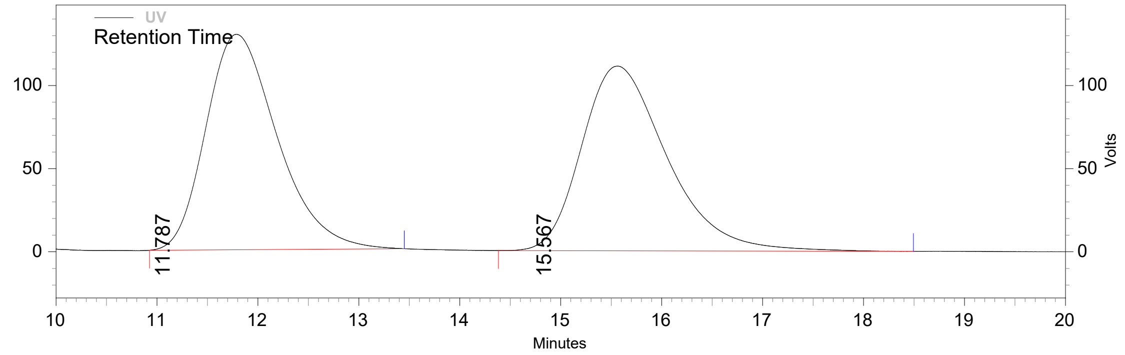

Retention Time	Height	Area	Area %
11.787	518293	25497873	49.43
15.567	444271	26081953	50.57

3fA, IH, cyclohexane:^{i}PrOH (50:50), 1.0 mL/min, 220 nm

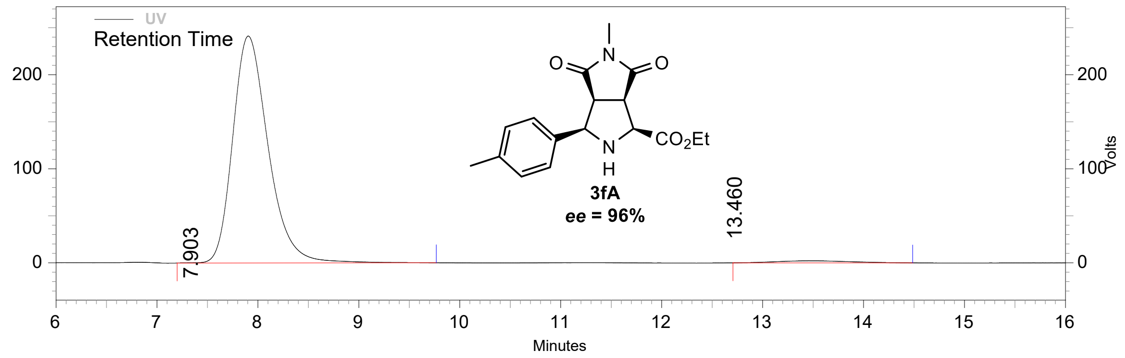

Retention Time	Height	Area	Area %
7.903	965394	23567659	98.13
13.460	8821	448931	1.87

3fA (rac.)

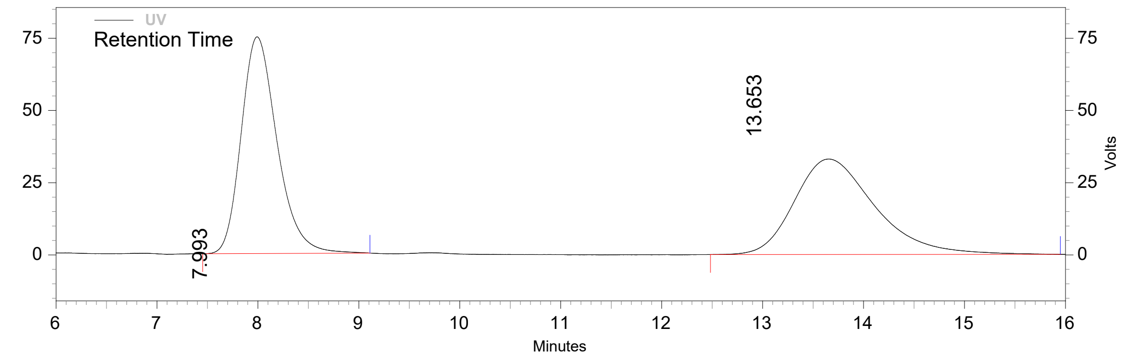

Retention Time	Height	Area	Area %
7.993	299987	7411278	50.30
13.653	132160	7323310	49.70

3gA, IH, cyclohexane:^{i}PrOH (50:50), 1.0 mL/min, 220 nm

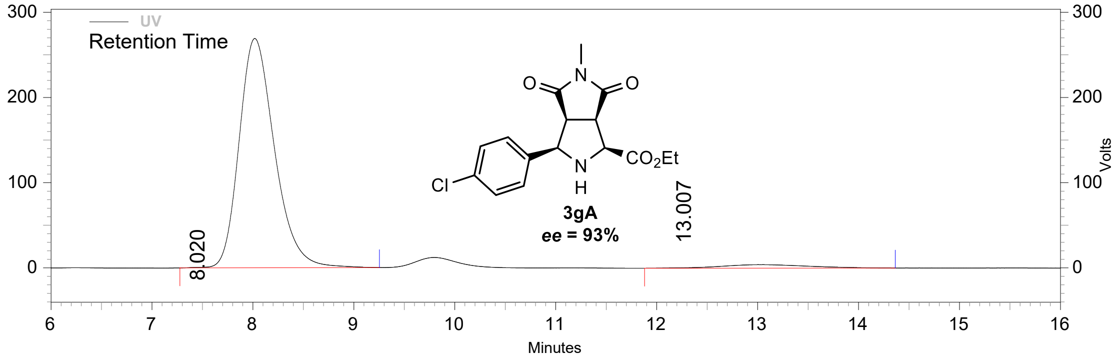

**Retention Time**	Height	Area	Area %
8.020	1076469	26259632	96.44
13.007	16090	969794	3.56

3gA (rac.)

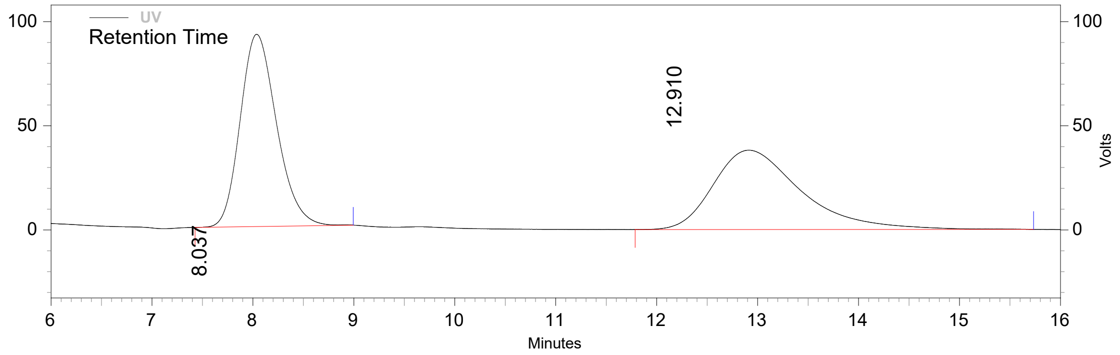

**Retention Time**	Height	Area	Area %
8.037	369115	9258529	50.16
12.910	152345	9198142	49.84

3hA, IH, cyclohexane:^{i}PrOH (50:50), 1.0 mL/min, 220 nm

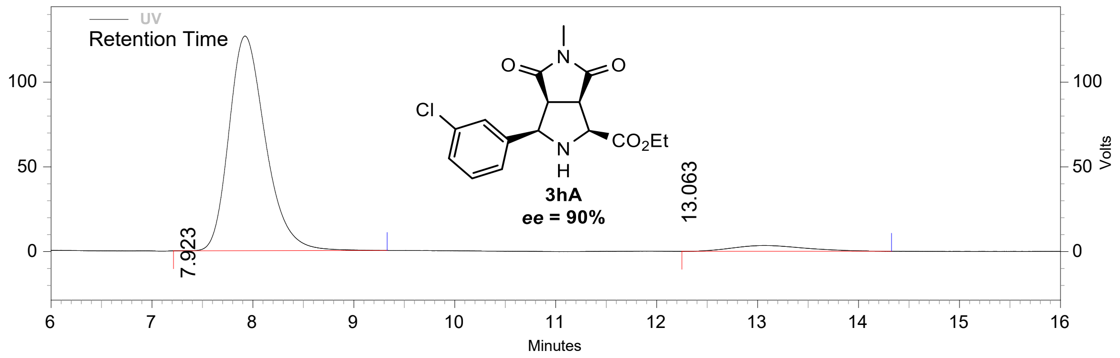

Retention Time	Height	Area	Area %
7.923	507011	12758797	94.82
13.063	13706	697417	5.18

3hA (rac.)

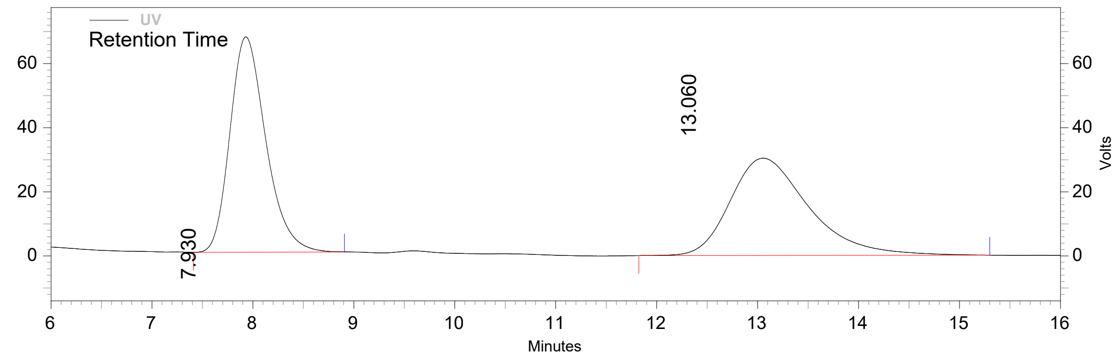

Retention Time	Height	Area	Area %
7.930	268732	6644894	50.33
13.060	121385	6557434	49.67

3iA, IH, cyclohexane:^{i}PrOH (50:50), 1.0 mL/min, 220 nm

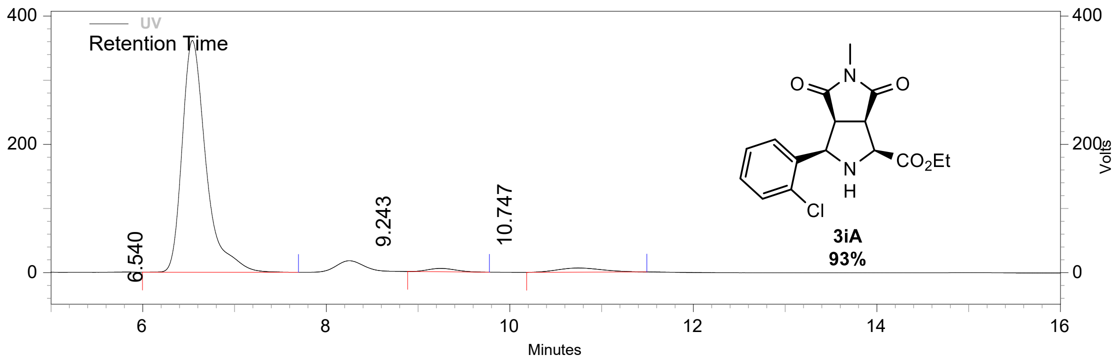

Retention Time	Height	Area	Area %
6.540	1445107	26060107	95.18
9.243	21188	444514	1.62
10.747	25915	875971	3.20

3iA (rac.)

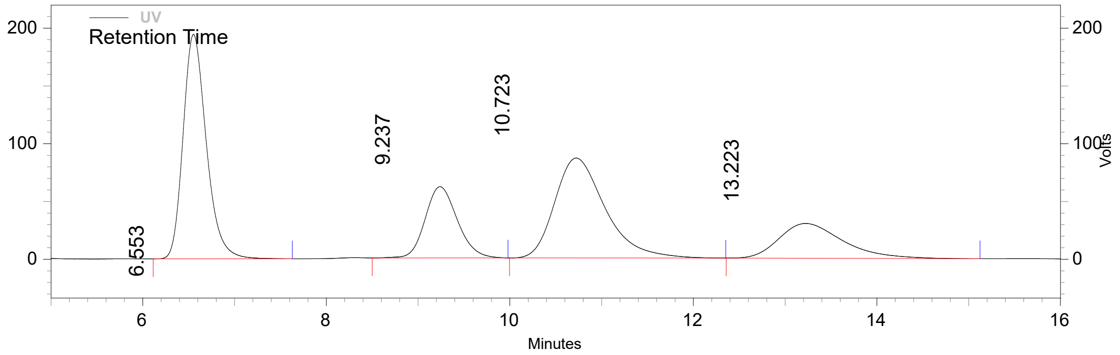

Retention Time	Height	Area	Area %
6.553	776864	13678735	35.36
9.237	246566	6006429	15.53
10.723	345748	13171447	34.05
13.223	120487	5826010	15.06

3jA, IH, cyclohexane:^{i}PrOH (50:50), 1.0 mL/min, 254 nm

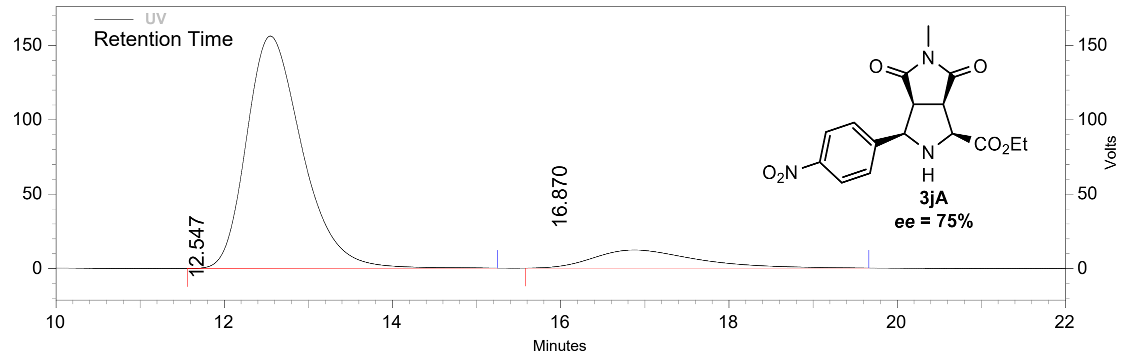

Retention Time	Height	Area	Area %
12.547	624972	28685631	87.46
16.870	48729	4112608	12.54

3jA (rac.)

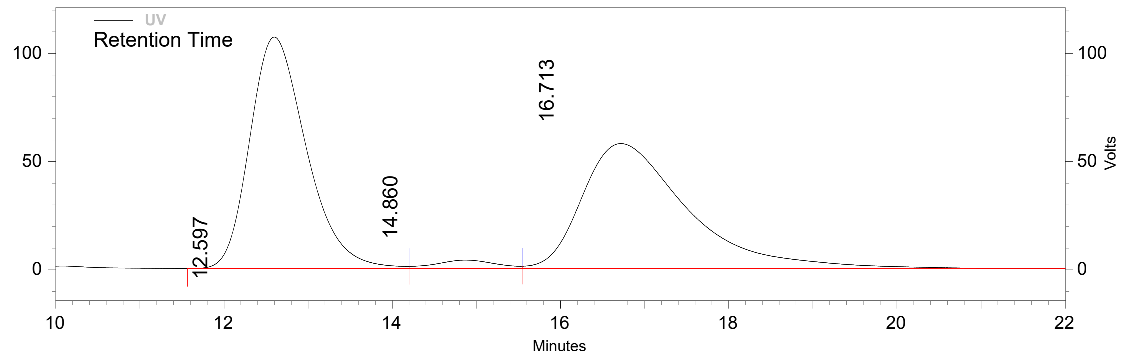

Retention Time	Height	Area	Area %
12.597	427691	19954163	48.77
14.860	15663	767608	1.88
16.713	230981	20190740	49.35

3aB, IH, cyclohexane:^{i}PrOH (50:50), 1.0 mL/min, 220 nm

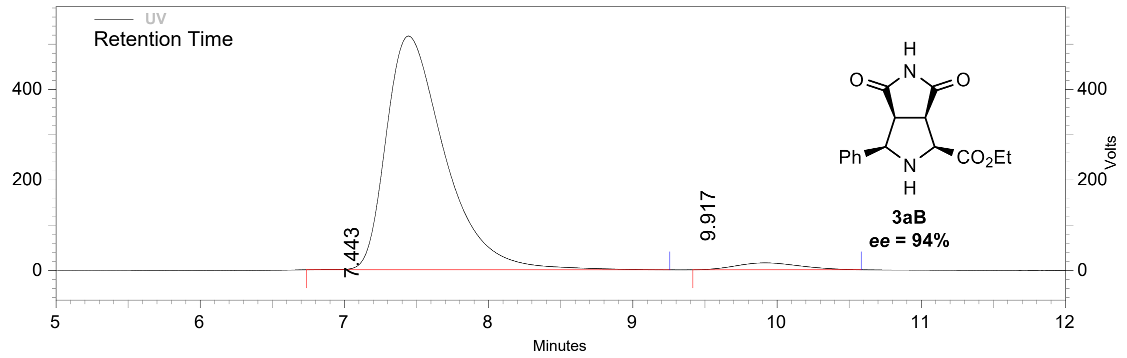

Retention Time	Height	Area	Area %
7.443	2066236	58252714	96.87
9.917	60848	1885014	3.13

3aB (rac.)

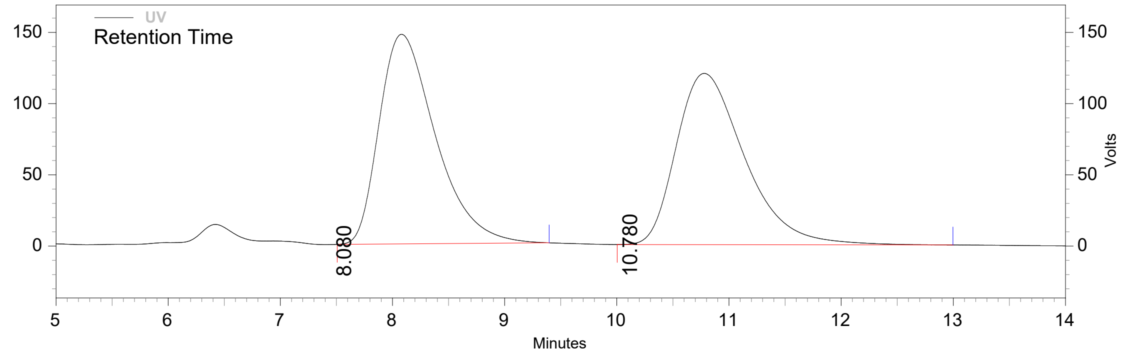

Retention Time	Height	Area	Area %
8.080	588507	20545540	50.23
10.780	480870	20355761	49.77

3aC, IH, cyclohexane:^{i}PrOH (50:50), 1.0 mL/min, 220 nm

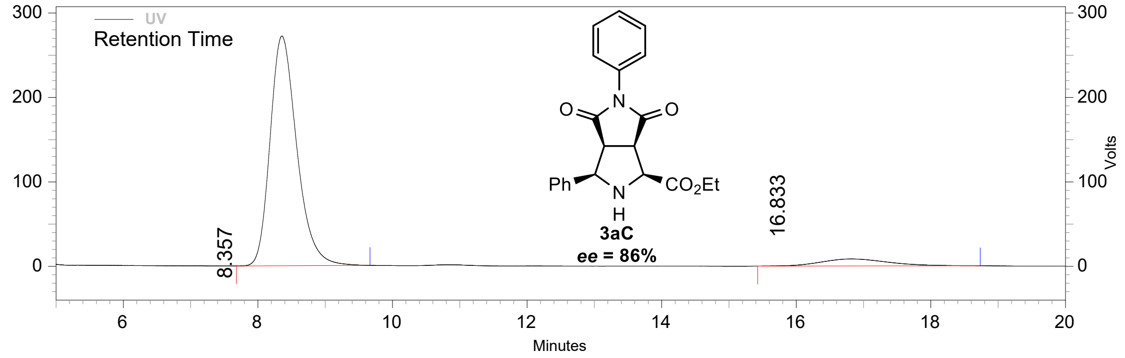

Retention Time	Height	Area	Area %
8.357	1088280	30011342	92.92
16.833	33138	2287972	7.08

3aC (rac.)

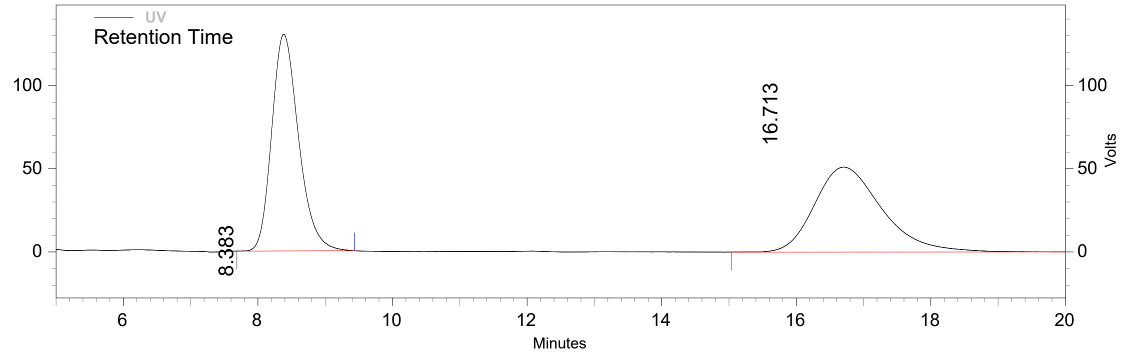

Retention Time	Height	Area	Area %
8.383	521041	14460828	50.45
16.713	204210	14203482	49.55

3aD, IH, cyclohexane:^{i}PrOH (50:50), 1.0 mL/min, 220 nm

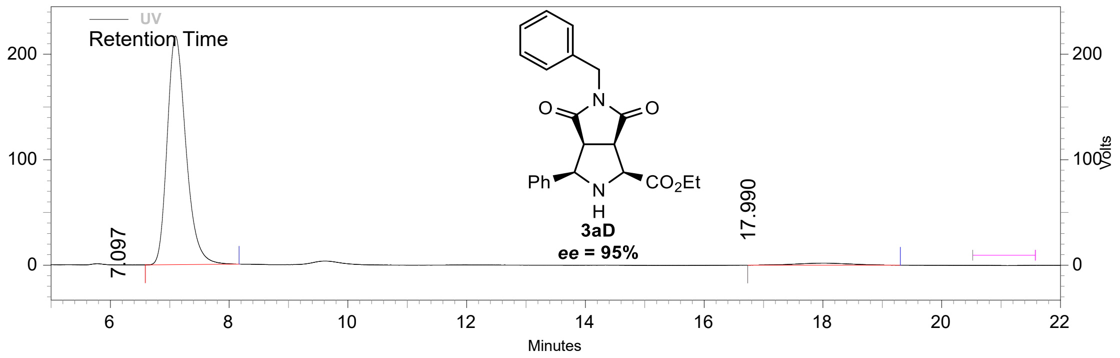

**Retention Time**	Height	Area	Area %
7.097	867042	18986398	97.43
17.990	7768	501512	2.57

3aD (rac.)

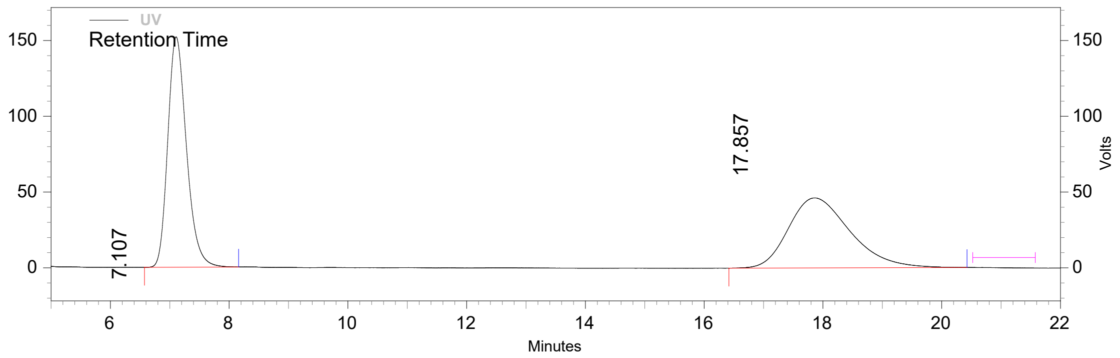

**Retention Time**	Height	Area	Area %
7.107	608078	13274674	49.93
17.857	184730	13313162	50.07

3bA, IA, cyclohexane:^{i}PrOH (70:30), 0.7 mL/min, 220 nm

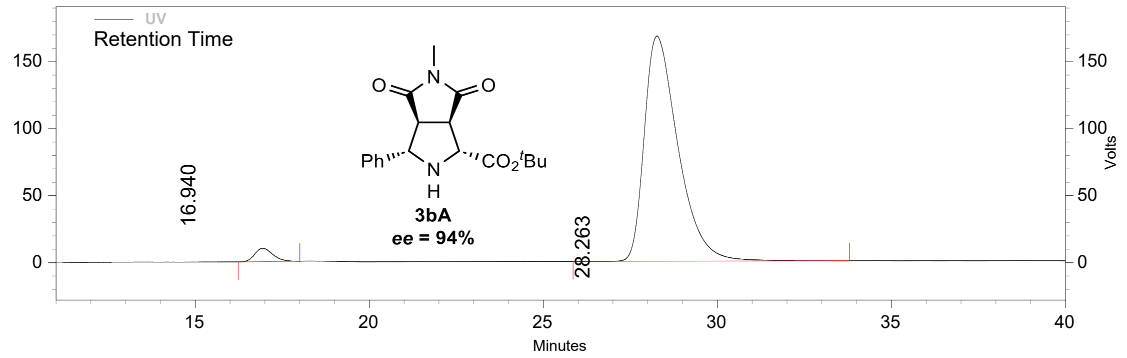

**Retention Time**	Height	Area	Area %
16.940	40088	1437072	3.07
28.263	672162	45330513	96.93

3bA (rac.)

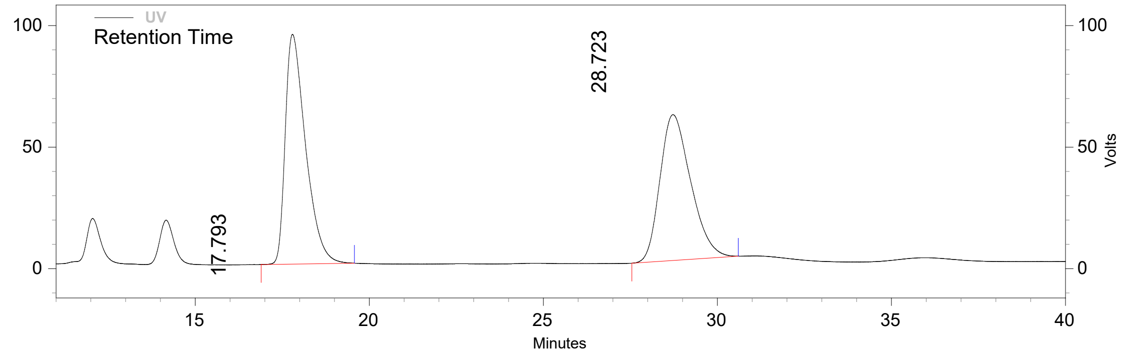

**Retention Time**	Height	Area	Area %
17.793	378237	15380919	51.10
28.723	240030	14721132	48.90

3IA, IA, cyclohexane:^{i}PrOH (50:50), 1.0 mL/min, 220 nm

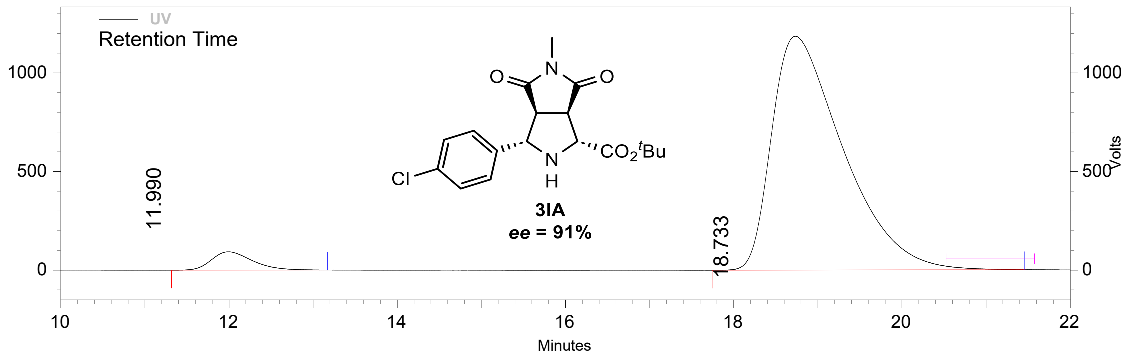

**Retention Time**	Height	Area	Area %
11.990	371006	12431746	4.26
18.733	4740865	279452431	95.74

3IA (rac.)

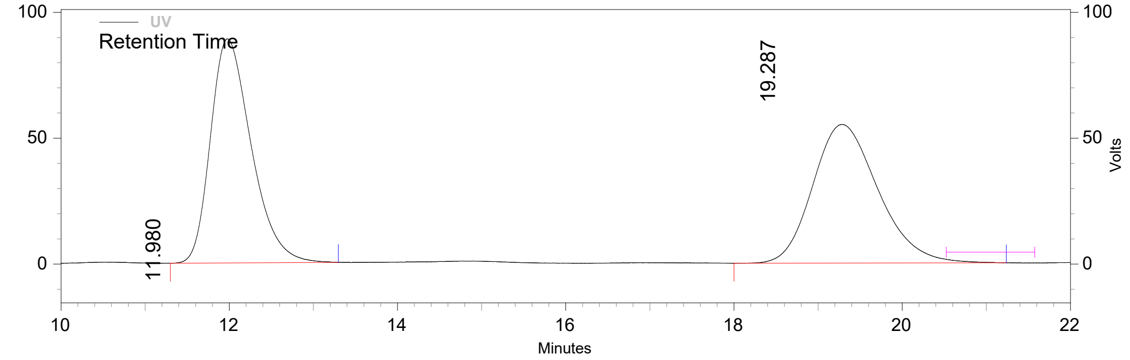

**Retention Time**	Height	Area	Area %
11.980	356075	11969780	50.13
19.287	220340	11909237	49.87

3kA, IA, cyclohexane:^{i}PrOH (50:50), 1.0 mL/min, 220 nm

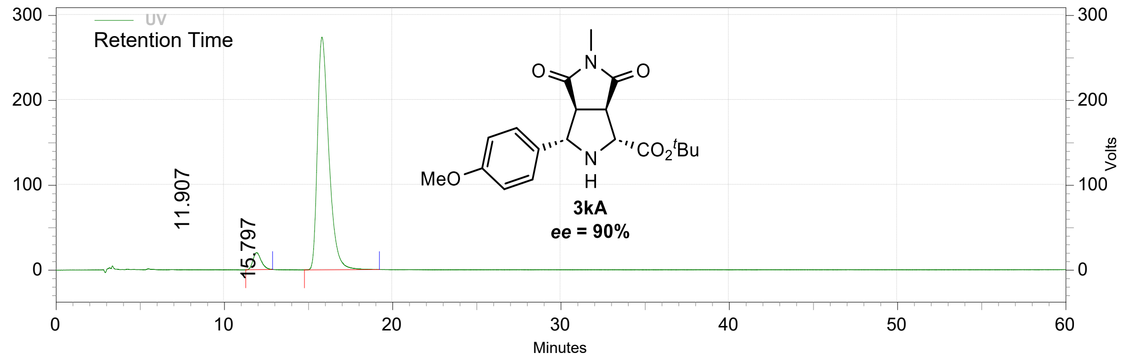

Retention Time	Height	Area	Area %
11.907	2751413	5.09	80547
15.797	51264650	94.91	1096671

3kA (rac.)

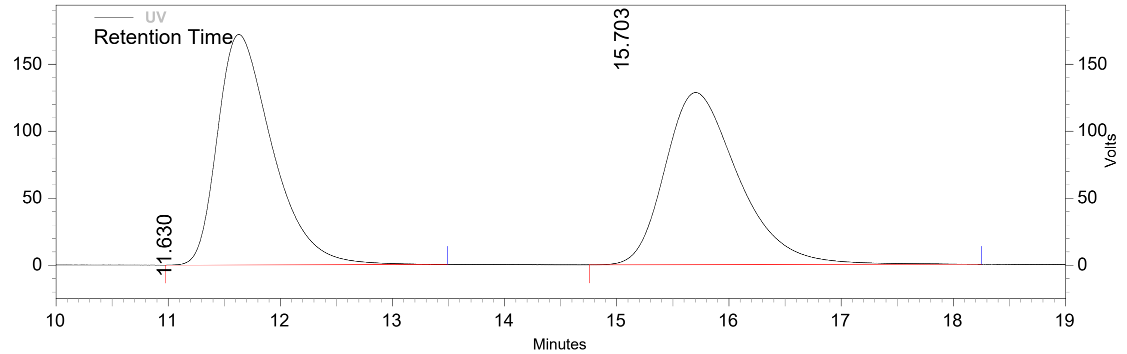

Retention Time	Height	Area	Area %
11.630	688024	23386039	49.96
15.703	513905	23422389	50.04

3kA, IA, cyclohexane:^{i}PrOH (50:50), 0.7 mL/min, 220 nm

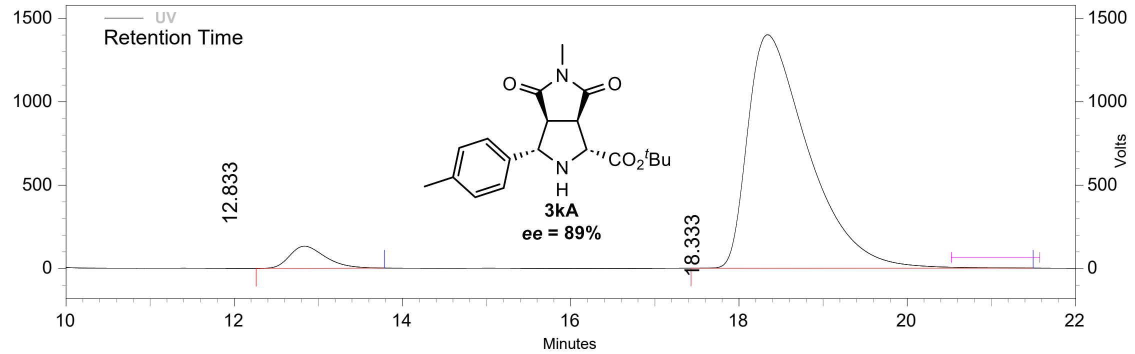

Retention Time	Height	Area	Area %
12.833	529978	15597878	5.28
18.333	5598893	280026879	94.72

3kA (rac.)

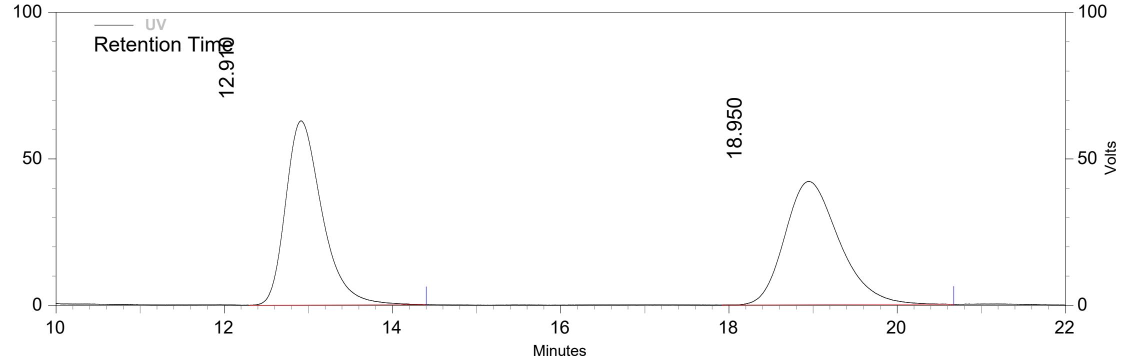

Retention Time	Height	Area	Area %
12.910	251550	7570452	49.97
18.950	168590	7578650	50.03

3nA, IA, cyclohexane:^{i}PrOH (50:50), 1.0 mL/min, 220 nm

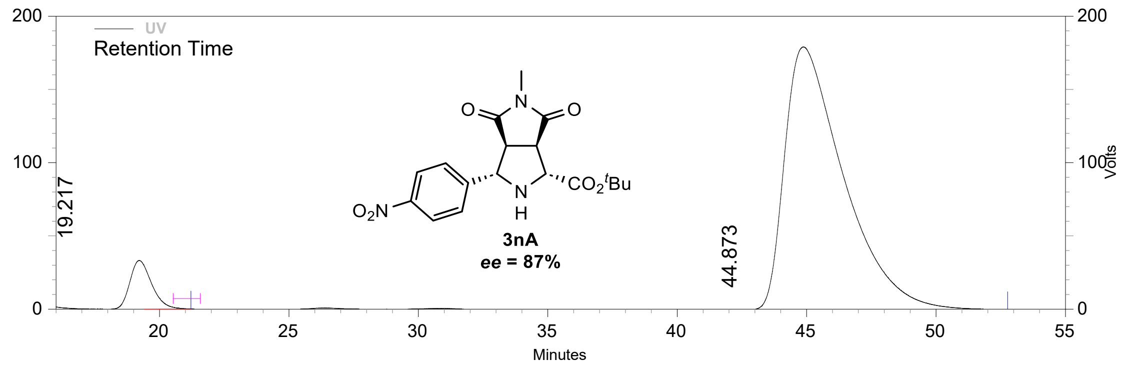

Retention Time	Height	Area	Area %
19.217	132850	7744622	6.38
44.873	717930	113676367	93.62

3nA (rac.)

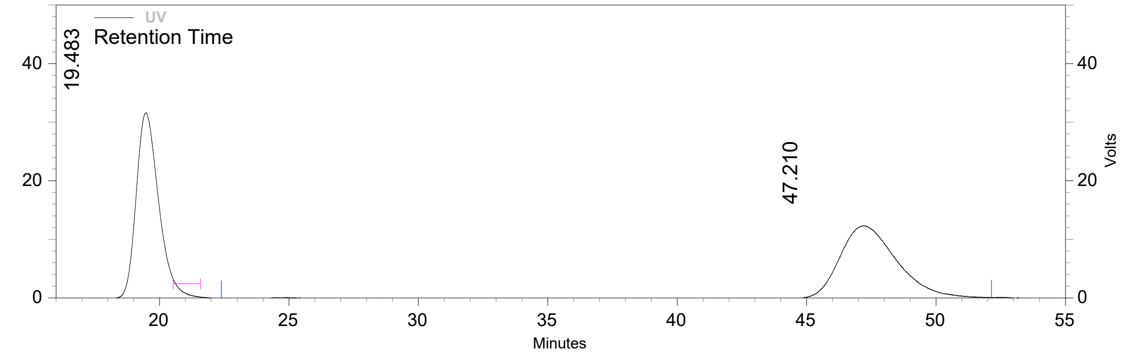

Retention Time	Height	Area	Area %
19.483	126928	7752960	50.38
47.210	49787	7635643	40.62

3bC, IA, cyclohexane:^{i}PrOH (50:50), 0.7 mL/min, 220 nm

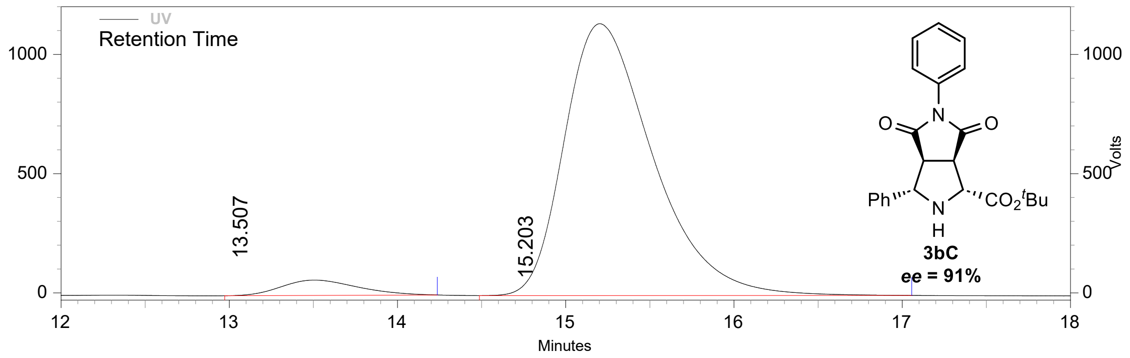

Retention Time	Height	Area	Area %
13.507	257404	8095237	4.63
15.203	45596	166804109	95.37

3bC (rac.)

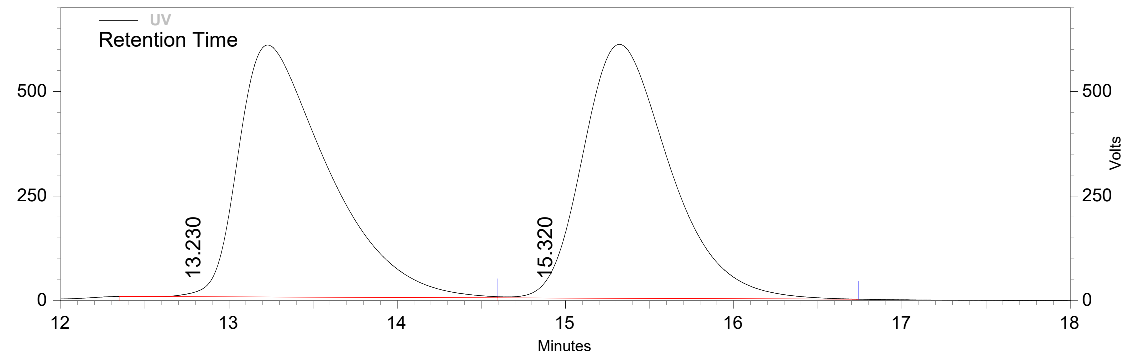

Retention Time	Height	Area	Area %
13.230	2409086	89044707	50.47
15.320	2427985	87389299	49.53

3bD, IA, cyclohexane:^{i}PrOH (50:50), 1.0 mL/min, 220 nm

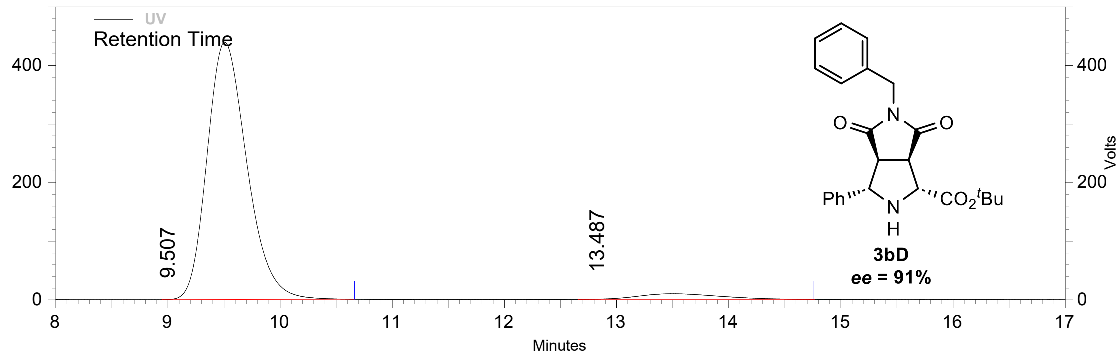

Retention Time	Height	Area	Area %
9.507	1758117	43417988	95.68
13.487	38684	1962537	4.32

3bD (rac.)

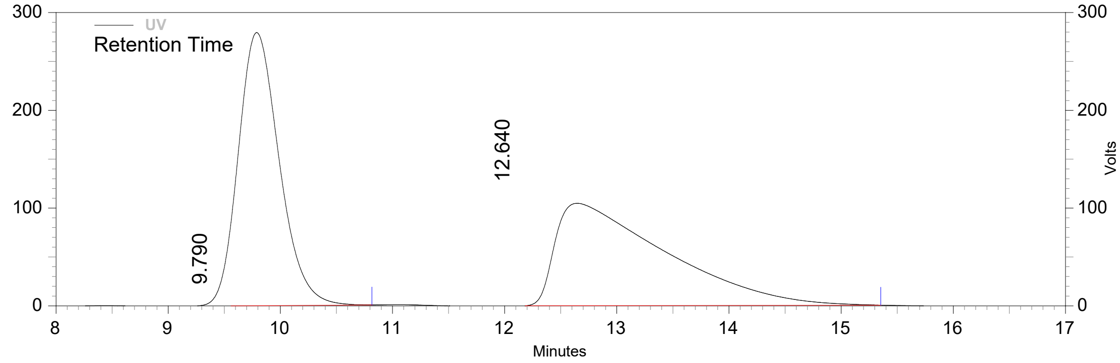

Retention Time	Height	Area	Area %
9.790	1117283	28395019	49.84
12.640	418754	28575893	50.16

18 *dr*-values of catalytic reactions

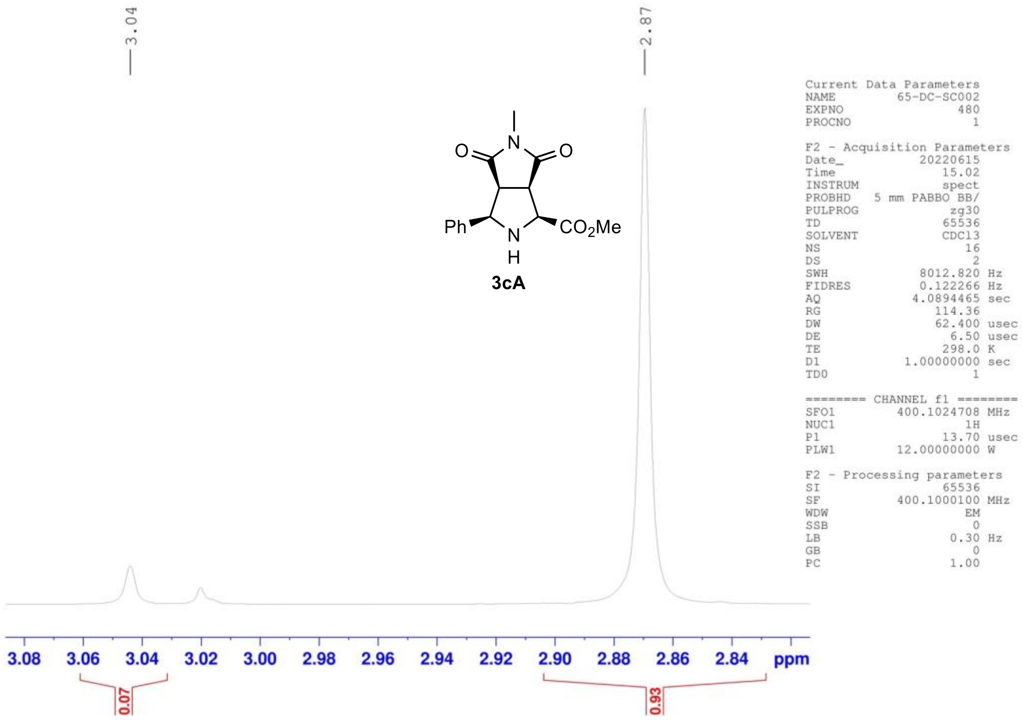

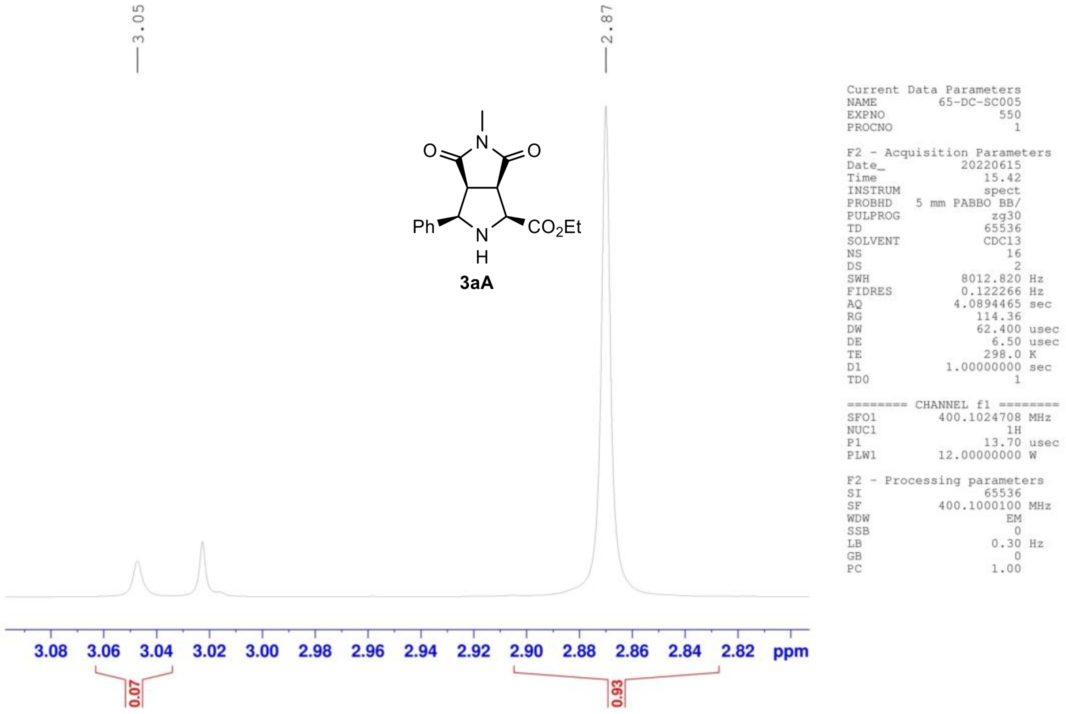
3.05
2.87
3aA
Ph
N
H
CO_2Et
O
O
N
Current Data Parameters
NAME 65-DC-SC005
EXPNO 550
PROCNO 1
F2 - Acquisition Parameters
Date_ 20220615
Time 15.42
INSTRUM spect
PROBHD 5 mm PABBO BB/
PULPROG zg30
TD 65536
SOLVENT CDCl3
NS 16
DS 2
SWH 8012.820 Hz
FIDRES 0.122266 Hz
AQ 4.0894465 sec
RG 114.36
DW 62.400 usec
DE 6.50 usec
TE 298.0 K
D1 1.00000000 sec
TD0 1
======== CHANNEL f1 ========
SFO1 400.1024708 MHz
NUC1 1H
P1 13.70 usec
PLW1 12.00000000 W
F2 - Processing parameters
SI 65536
SF 400.1000100 MHz
WDW EM
SSB 0
LB 0.30 Hz
GB 0
PC 1.00
3.08 3.06 3.04 3.02 3.00 2.98 2.96 2.94 2.92 2.90 2.88 2.86 2.84 2.82 ppm
0.07
0.93

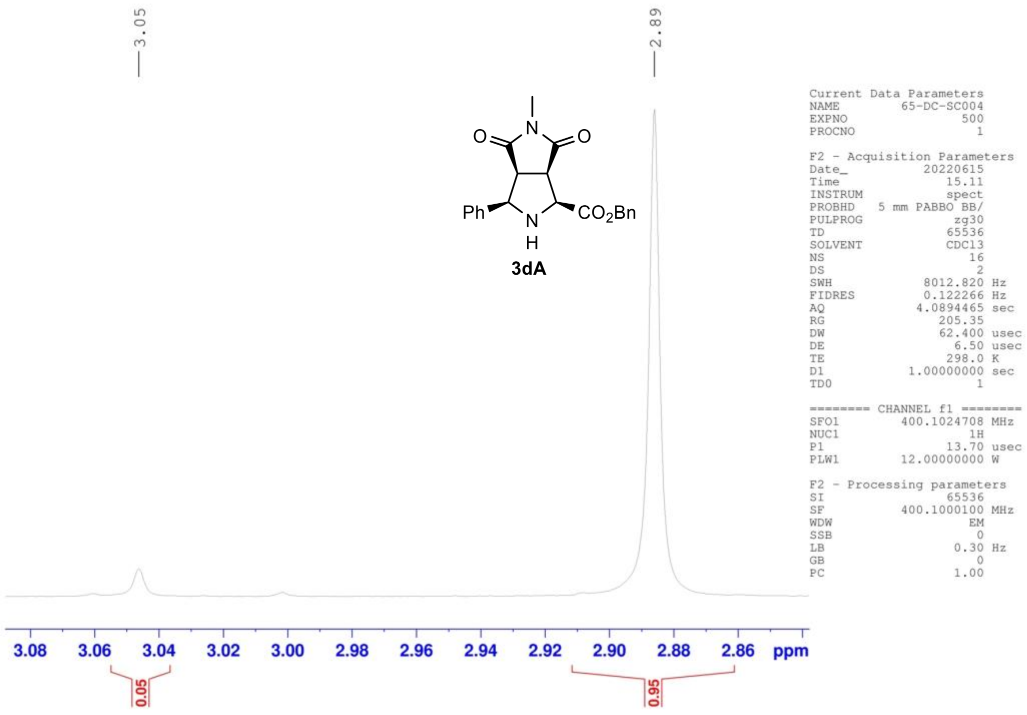
3.05
2.89
3dA
Ph
N
H
CO_2Bn
O
O
N
Current Data Parameters
NAME 65-DC-SC004
EXPNO 500
PROCNO 1
F2 - Acquisition Parameters
Date_ 20220615
Time 15.11
INSTRUM spect
PROBHD 5 mm PABBO BB/
PULPROG zg30
TD 65536
SOLVENT CDCl3
NS 16
DS 2
SWH 8012.820 Hz
FIDRES 0.122266 Hz
AQ 4.0894465 sec
RG 205.35
DW 62.400 usec
DE 6.50 usec
TE 298.0 K
D1 1.00000000 sec
TD0 1
======== CHANNEL f1 ========
SFO1 400.1024708 MHz
NUC1 1H
P1 13.70 usec
PLW1 12.00000000 W
F2 - Processing parameters
SI 65536
SF 400.1000100 MHz
WDW EM
SSB 0
LB 0.30 Hz
GB 0
PC 1.00
3.08 3.06 3.04 3.02 3.00 2.98 2.96 2.94 2.92 2.90 2.88 2.86 ppm
0.05
0.95

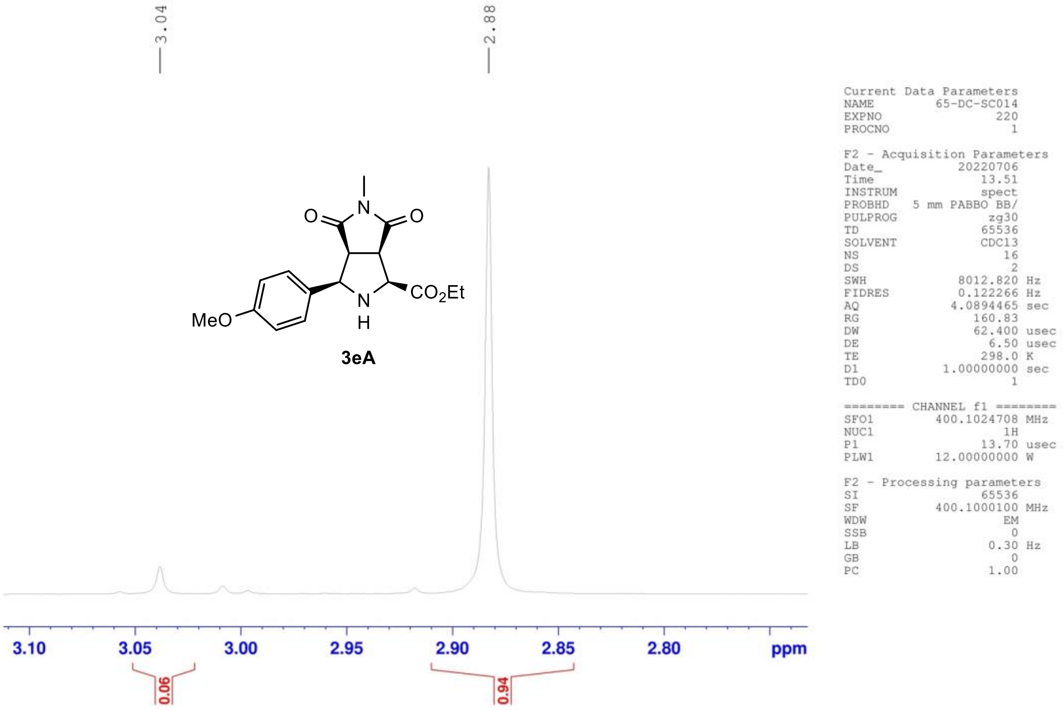
3.04
2.88
O
N
O
MeO
N
H
CO2Et
3eA
Current Data Parameters
NAME 65-DC-SC014
EXPNO 220
PROCNO 1
F2 - Acquisition Parameters
Date_ 20220706
Time 13.51
INSTRUM spect
PROBHD 5 mm PABBO BB/
PULPROG zg30
TD 65536
SOLVENT CDCl3
NS 16
DS 2
SWH 8012.820 Hz
FIDRES 0.122266 Hz
AQ 4.0894465 sec
RG 160.83
DW 62.400 usec
DE 6.50 usec
TE 298.0 K
D1 1.00000000 sec
TD0 1
======== CHANNEL f1 ========
SFO1 400.1024708 MHz
NUC1 1H
P1 13.70 usec
PLW1 12.00000000 W
F2 - Processing parameters
SI 65536
SF 400.1000100 MHz
WDW EM
SSB 0
LB 0.30 Hz
GB 0
PC 1.00
3.10
3.05
3.00
2.95
2.90
2.85
2.80
ppm
0.06
0.94

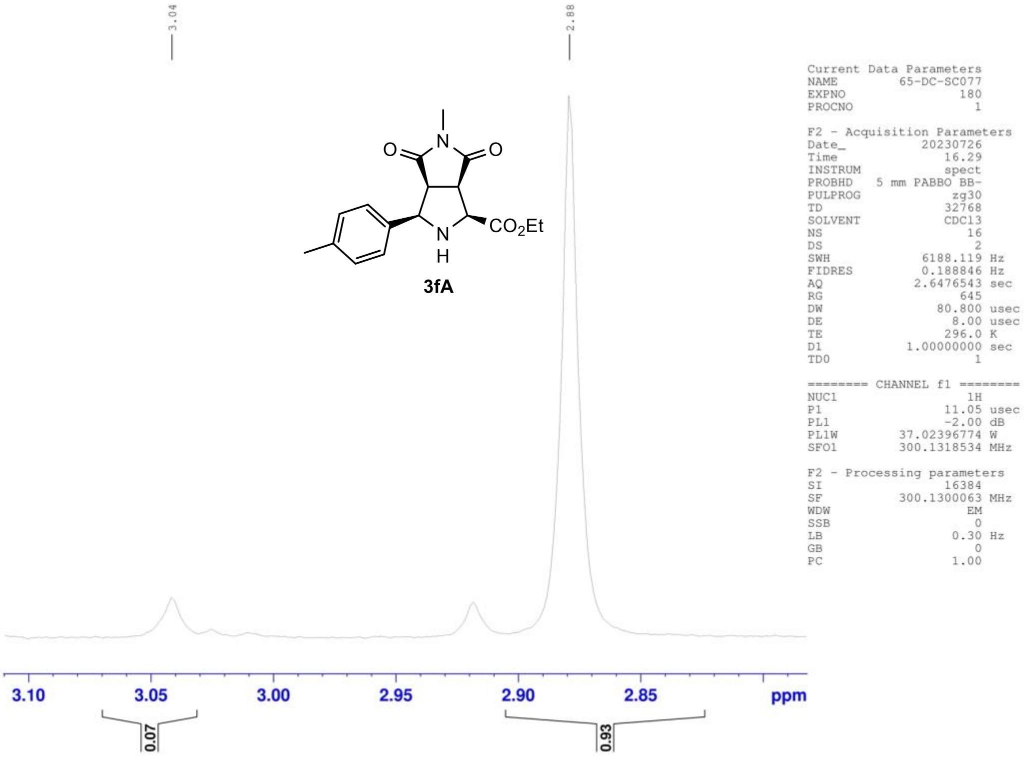
3.04
2.88
O
N
O
N
H
CO2Et
3fA
Current Data Parameters
NAME 65-DC-SC077
EXPNO 180
PROCNO 1
F2 - Acquisition Parameters
Date_ 20230726
Time 16.29
INSTRUM spect
PROBHD 5 mm PABBO BB-
PULPROG zg30
TD 32768
SOLVENT CDCl3
NS 16
DS 2
SWH 6188.119 Hz
FIDRES 0.188846 Hz
AQ 2.6476543 sec
RG 645
DW 80.800 usec
DE 8.00 usec
TE 296.0 K
D1 1.00000000 sec
TD0 1
======== CHANNEL f1 ========
NUC1 1H
P1 11.05 usec
PL1 -2.00 dB
PL1W 37.02396774 W
SFO1 300.1318534 MHz
F2 - Processing parameters
SI 16384
SF 300.1300063 MHz
WDW EM
SSB 0
LB 0.30 Hz
GB 0
PC 1.00
3.10
3.05
3.00
2.95
2.90
2.85
ppm
0.07
0.93

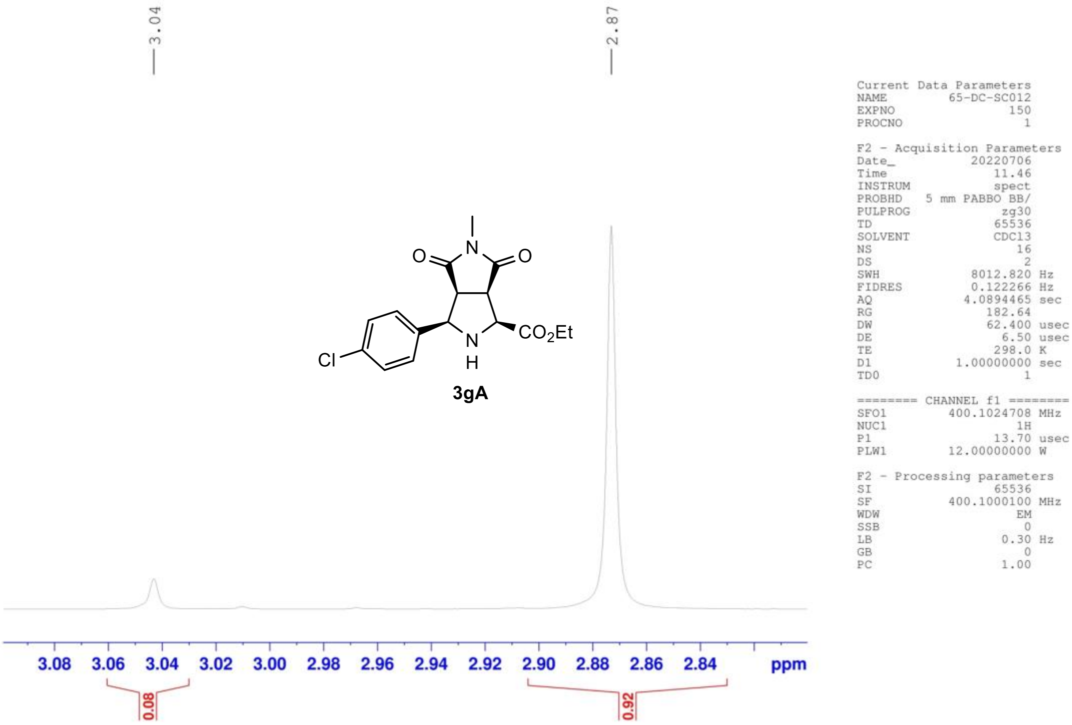
3.04
2.87
3gA
Current Data Parameters
NAME 65-DC-SC012
EXPNO 150
PROCNO 1
F2 - Acquisition Parameters
Date_ 20220706
Time 11.46
INSTRUM spect
PROBHD 5 mm PABBO BB/
PULPROG zg30
TD 65536
SOLVENT CDCl3
NS 16
DS 2
SWH 8012.820 Hz
FIDRES 0.122266 Hz
AQ 4.0894465 sec
RG 182.64
DW 62.400 usec
DE 6.50 usec
TE 298.0 K
D1 1.00000000 sec
TD0 1
======== CHANNEL f1 ========
SFO1 400.1024708 MHz
NUC1 1H
P1 13.70 usec
PLW1 12.00000000 W
F2 - Processing parameters
SI 65536
SF 400.1000100 MHz
WDW EM
SSB 0
LB 0.30 Hz
GB 0
PC 1.00
3.08 3.06 3.04 3.02 3.00 2.98 2.96 2.94 2.92 2.90 2.88 2.86 2.84 ppm
0.08
0.92

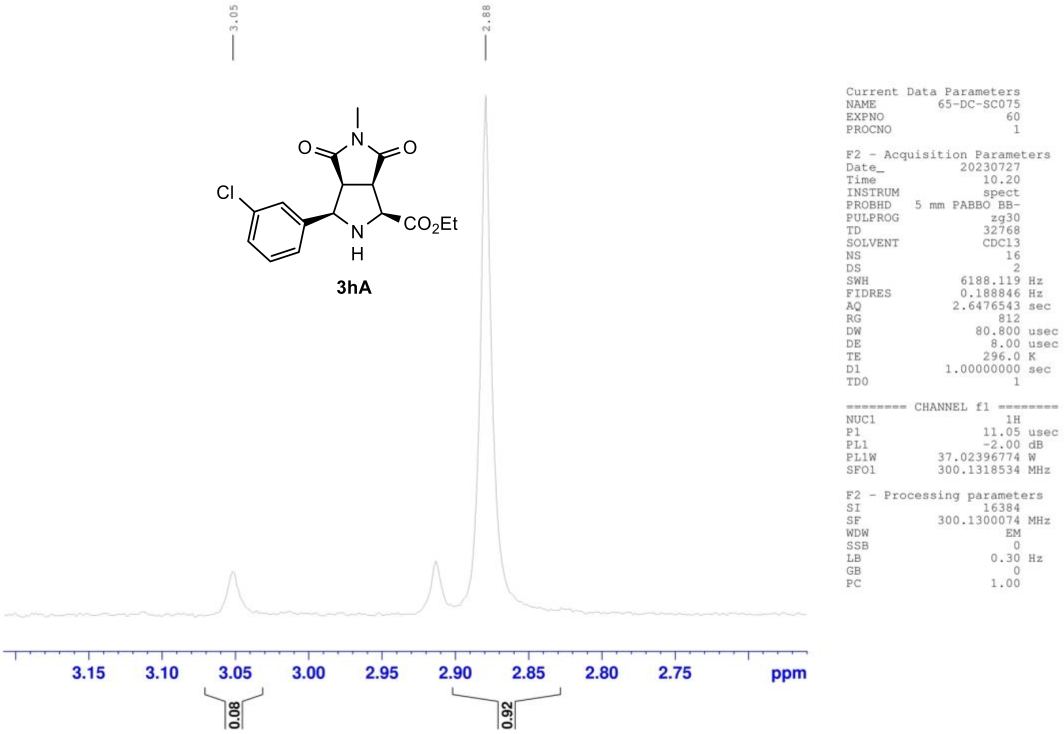
3.05
2.88
3hA
Current Data Parameters
NAME 65-DC-SC075
EXPNO 60
PROCNO 1
F2 - Acquisition Parameters
Date_ 20230727
Time 10.20
INSTRUM spect
PROBHD 5 mm PABBO BB-
PULPROG zg30
TD 32768
SOLVENT CDCl3
NS 16
DS 2
SWH 6188.119 Hz
FIDRES 0.188846 Hz
AQ 2.6476543 sec
RG 812
DW 80.800 usec
DE 8.00 usec
TE 296.0 K
D1 1.00000000 sec
TD0 1
======== CHANNEL f1 ========
NUC1 1H
P1 11.05 usec
PL1 -2.00 dB
PL1W 37.02396774 W
SFO1 300.1318534 MHz
F2 - Processing parameters
SI 16384
SF 300.1300074 MHz
WDW EM
SSB 0
LB 0.30 Hz
GB 0
PC 1.00
3.15 3.10 3.05 3.00 2.95 2.90 2.85 2.80 2.75 ppm
0.08
0.92

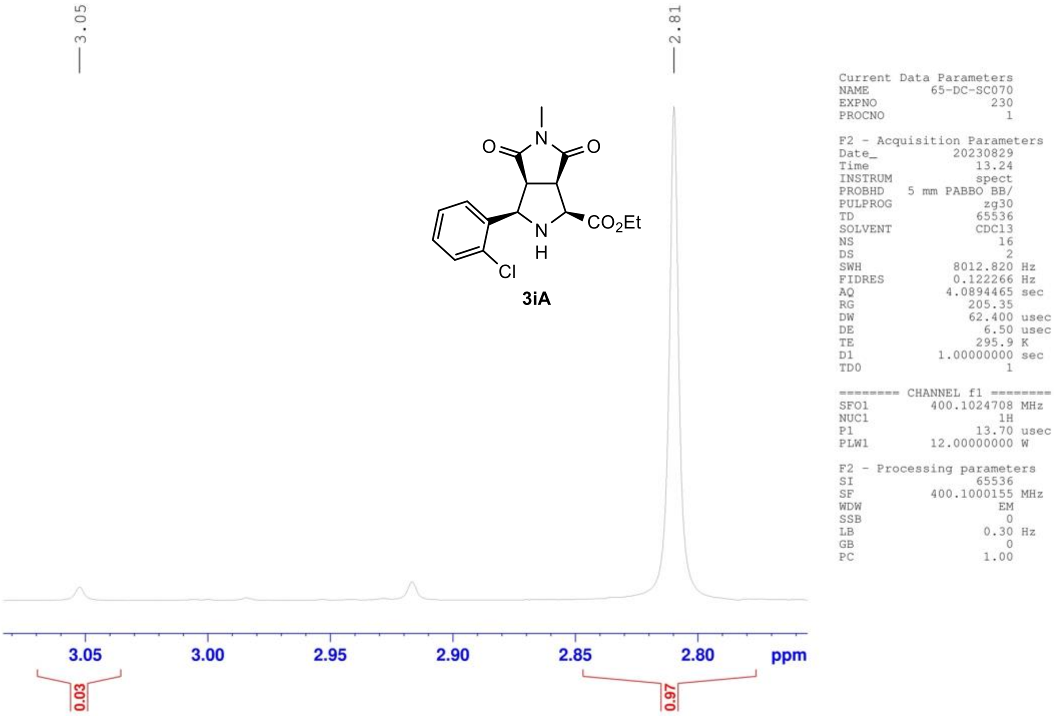
3.05
2.81
3iA
CO2Et
Cl
3.05 3.00 2.95 2.90 2.85 2.80 ppm
0.03
0.97
Current Data Parameters
NAME 65-DC-SC070
EXPNO 230
PROCNO 1
F2 - Acquisition Parameters
Date_ 20230829
Time 13.24
INSTRUM spect
PROBHD 5 mm PABBO BB/
PULPROG zg30
TD 65536
SOLVENT CDCl3
NS 16
DS 2
SWH 8012.820 Hz
FIDRES 0.122266 Hz
AQ 4.0894465 sec
RG 205.35
DW 62.400 usec
DE 6.50 usec
TE 295.9 K
D1 1.00000000 sec
TD0 1
======== CHANNEL f1 ========
SFO1 400.1024708 MHz
NUC1 1H
P1 13.70 usec
PLW1 12.00000000 W
F2 - Processing parameters
SI 65536
SF 400.1000155 MHz
WDW EM
SSB 0
LB 0.30 Hz
GB 0
PC 1.00

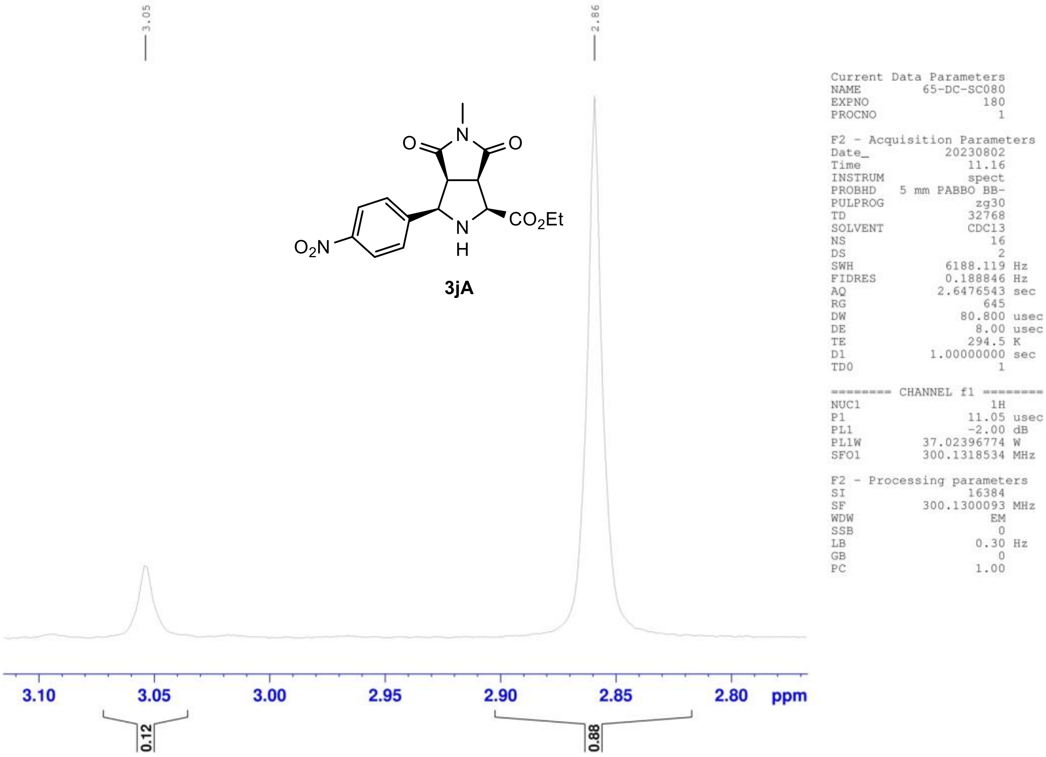
3.05
2.86
3jA
O2N
CO2Et
3.10 3.05 3.00 2.95 2.90 2.85 2.80 ppm
0.12
0.88
Current Data Parameters
NAME 65-DC-SC080
EXPNO 180
PROCNO 1
F2 - Acquisition Parameters
Date_ 20230802
Time 11.16
INSTRUM spect
PROBHD 5 mm PABBO BB-
PULPROG zg30
TD 32768
SOLVENT CDCl3
NS 16
DS 2
SWH 6188.119 Hz
FIDRES 0.188846 Hz
AQ 2.6476543 sec
RG 645
DW 80.800 usec
DE 8.00 usec
TE 294.5 K
D1 1.00000000 sec
TD0 1
======== CHANNEL f1 ========
NUC1 1H
P1 11.05 usec
PL1 -2.00 dB
PL1W 37.02396774 W
SFO1 300.1318534 MHz
F2 - Processing parameters
SI 16384
SF 300.1300093 MHz
WDW EM
SSB 0
LB 0.30 Hz
GB 0
PC 1.00

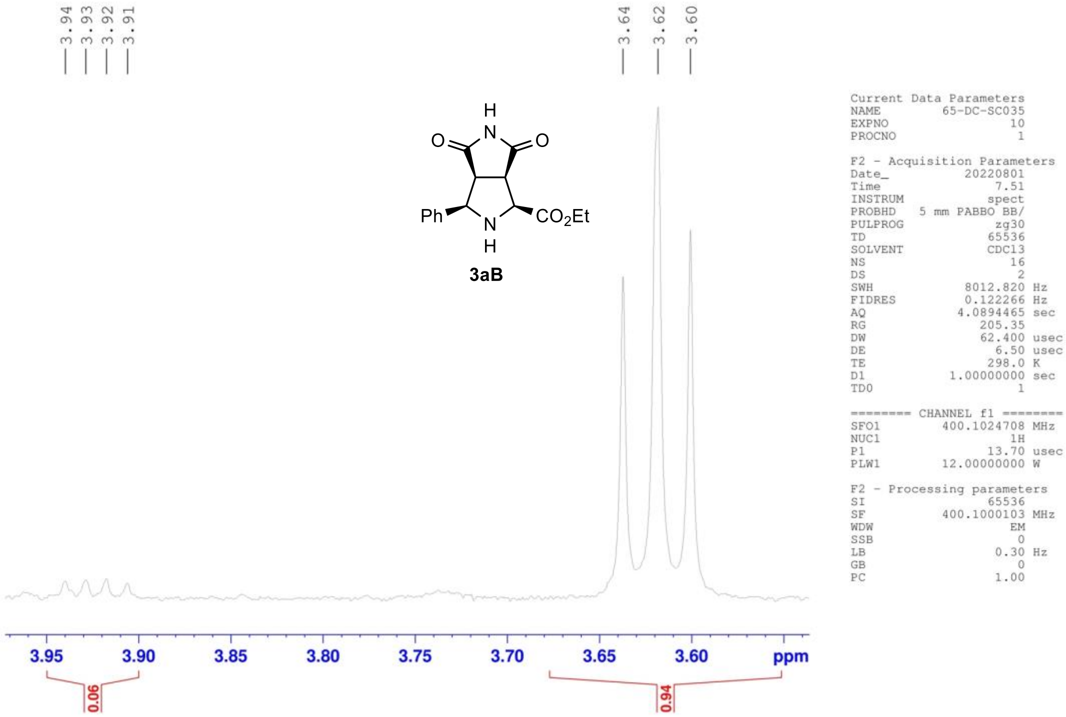
3.94
3.93
3.92
3.91
3.64
3.62
3.60
3aB
Ph
CO2Et
Current Data Parameters
NAME 65-DC-SC035
EXPNO 10
PROCNO 1
F2 - Acquisition Parameters
Date_ 20220801
Time 7.51
INSTRUM spect
PROBHD 5 mm PABBO BB/
PULPROG zg30
TD 65536
SOLVENT CDCl3
NS 16
DS 2
SWH 8012.820 Hz
FIDRES 0.122266 Hz
AQ 4.0894465 sec
RG 205.35
DW 62.400 usec
DE 6.50 usec
TE 298.0 K
D1 1.00000000 sec
TD0 1
======== CHANNEL f1 ========
SFO1 400.1024708 MHz
NUC1 1H
P1 13.70 usec
PLW1 12.00000000 W
F2 - Processing parameters
SI 65536
SF 400.1000103 MHz
WDW EM
SSB 0
LB 0.30 Hz
GB 0
PC 1.00
3.95 3.90 3.85 3.80 3.75 3.70 3.65 3.60 ppm
0.06
0.94

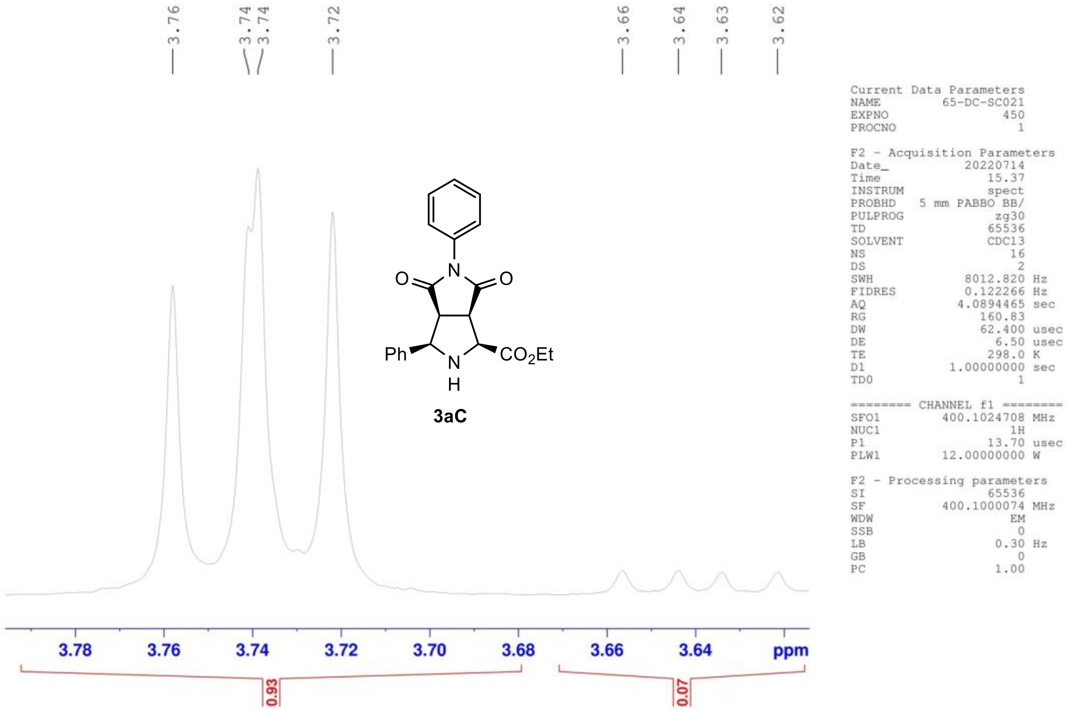
3.76
3.74
3.74
3.72
3.66
3.64
3.63
3.62
3aC
Ph
CO2Et
Current Data Parameters
NAME 65-DC-SC021
EXPNO 450
PROCNO 1
F2 - Acquisition Parameters
Date_ 20220714
Time 15.37
INSTRUM spect
PROBHD 5 mm PABBO BB/
PULPROG zg30
TD 65536
SOLVENT CDCl3
NS 16
DS 2
SWH 8012.820 Hz
FIDRES 0.122266 Hz
AQ 4.0894465 sec
RG 160.83
DW 62.400 usec
DE 6.50 usec
TE 298.0 K
D1 1.00000000 sec
TD0 1
======== CHANNEL f1 ========
SFO1 400.1024708 MHz
NUC1 1H
P1 13.70 usec
PLW1 12.00000000 W
F2 - Processing parameters
SI 65536
SF 400.1000074 MHz
WDW EM
SSB 0
LB 0.30 Hz
GB 0
PC 1.00
3.78 3.76 3.74 3.72 3.70 3.68 3.66 3.64 ppm
0.93
0.07

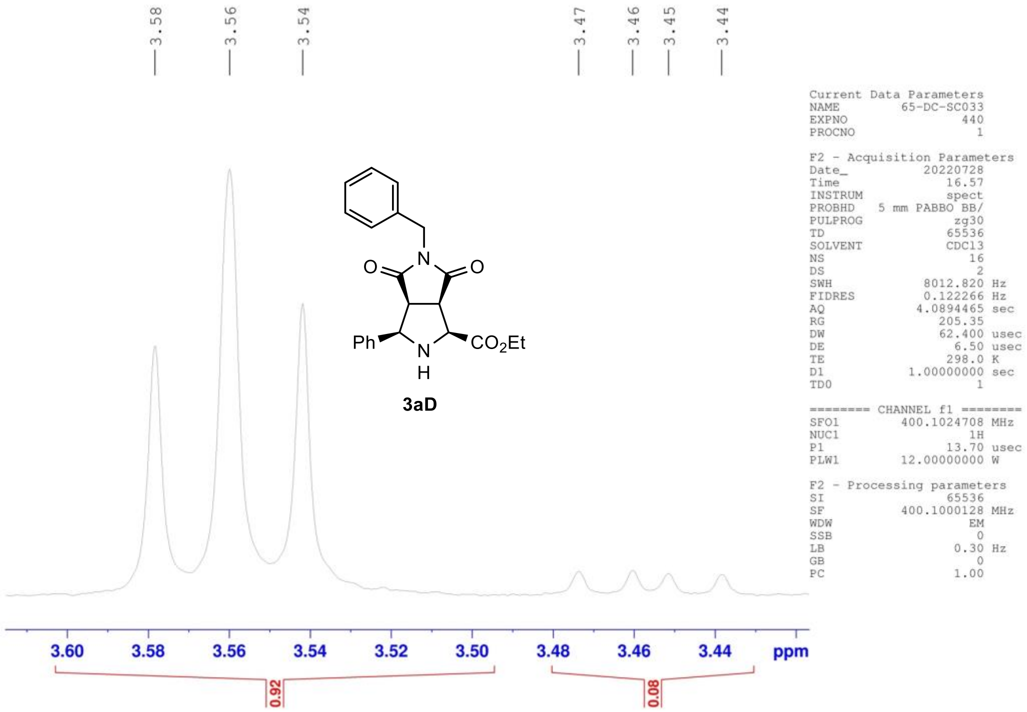
3.58
3.56
3.54
3.47
3.46
3.45
3.44
3aD
Ph
CO2Et
Current Data Parameters
NAME 65-DC-SC033
EXPNO 440
PROCNO 1
F2 - Acquisition Parameters
Date_ 20220728
Time 16.57
INSTRUM spect
PROBHD 5 mm PABBO BB/
PULPROG zg30
TD 65536
SOLVENT CDCl3
NS 16
DS 2
SWH 8012.820 Hz
FIDRES 0.122266 Hz
AQ 4.0894465 sec
RG 205.35
DW 62.400 usec
DE 6.50 usec
TE 298.0 K
D1 1.00000000 sec
TD0 1
======== CHANNEL f1 ========
SFO1 400.1024708 MHz
NUC1 1H
P1 13.70 usec
PLW1 12.00000000 W
F2 - Processing parameters
SI 65536
SF 400.1000128 MHz
WDW EM
SSB 0
LB 0.30 Hz
GB 0
PC 1.00
3.60 3.58 3.56 3.54 3.52 3.50 3.48 3.46 3.44 ppm
0.92
0.08

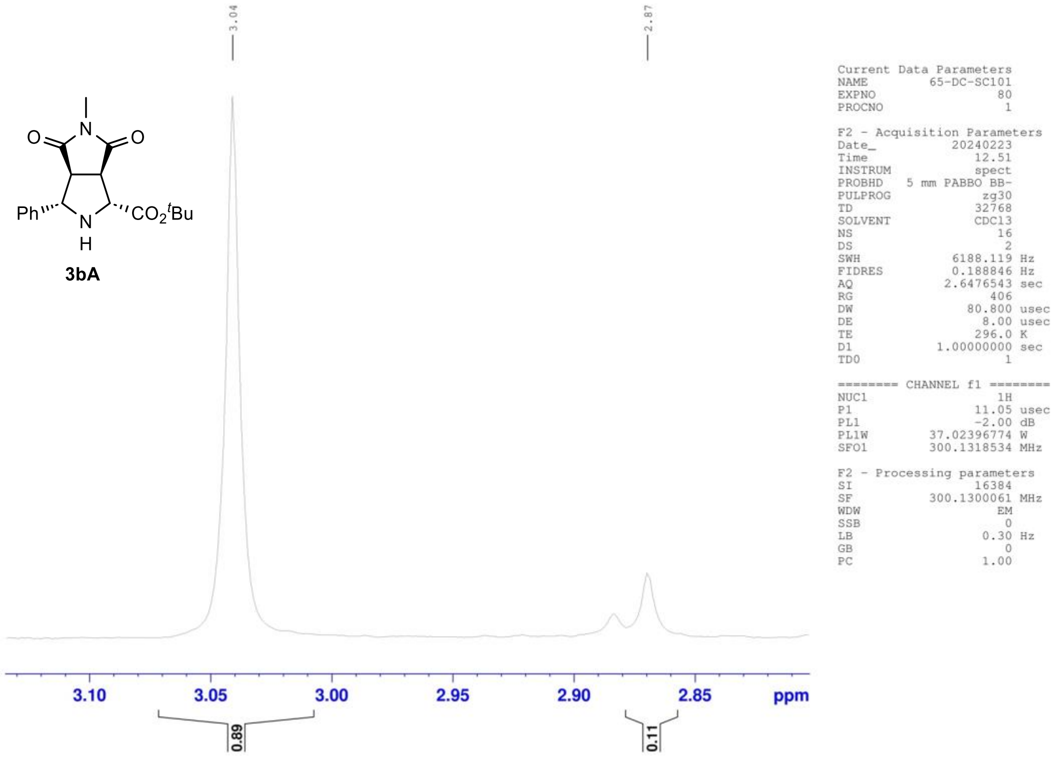
3.04
2.87
3bA
Ph
CO2tBu
Current Data Parameters
NAME 65-DC-SC101
EXPNO 80
PROCNO 1
F2 - Acquisition Parameters
Date_ 20240223
Time 12.51
INSTRUM spect
PROBHD 5 mm PABBO BB-
PULPROG zg30
TD 32768
SOLVENT CDCl3
NS 16
DS 2
SWH 6188.119 Hz
FIDRES 0.188846 Hz
AQ 2.6476543 sec
RG 406
DW 80.800 usec
DE 8.00 usec
TE 296.0 K
D1 1.00000000 sec
TD0 1
======== CHANNEL f1 ========
NUC1 1H
P1 11.05 usec
PL1 -2.00 dB
PL1W 37.02396774 W
SFO1 300.1318534 MHz
F2 - Processing parameters
SI 16384
SF 300.1300061 MHz
WDW EM
SSB 0
LB 0.30 Hz
GB 0
PC 1.00
3.10 3.05 3.00 2.95 2.90 2.85 ppm
0.89
0.11

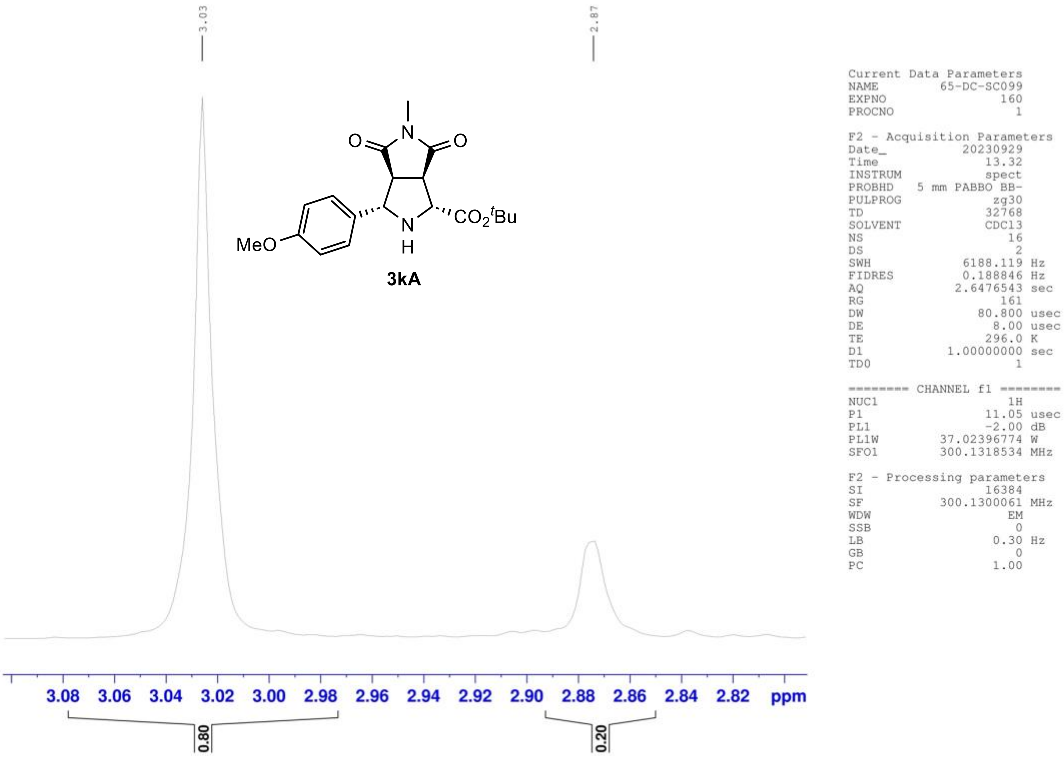
3.03
2.87
O
N
O
MeO
N
H
$CO_2{}^tBu$
3kA
Current Data Parameters
NAME 65-DC-SC099
EXPNO 160
PROCNO 1
F2 - Acquisition Parameters
Date_ 20230929
Time 13.32
INSTRUM spect
PROBHD 5 mm PABBO BB-
PULPROG zg30
TD 32768
SOLVENT CDCl3
NS 16
DS 2
SWH 6188.119 Hz
FIDRES 0.188846 Hz
AQ 2.6476543 sec
RG 161
DW 80.800 usec
DE 8.00 usec
TE 296.0 K
D1 1.00000000 sec
TD0 1
======== CHANNEL f1 ========
NUC1 1H
P1 11.05 usec
PL1 -2.00 dB
PL1W 37.02396774 W
SFO1 300.1318534 MHz
F2 - Processing parameters
SI 16384
SF 300.1300061 MHz
WDW EM
SSB 0
LB 0.30 Hz
GB 0
PC 1.00
3.08 3.06 3.04 3.02 3.00 2.98 2.96 2.94 2.92 2.90 2.88 2.86 2.84 2.82 ppm
0.80
0.20

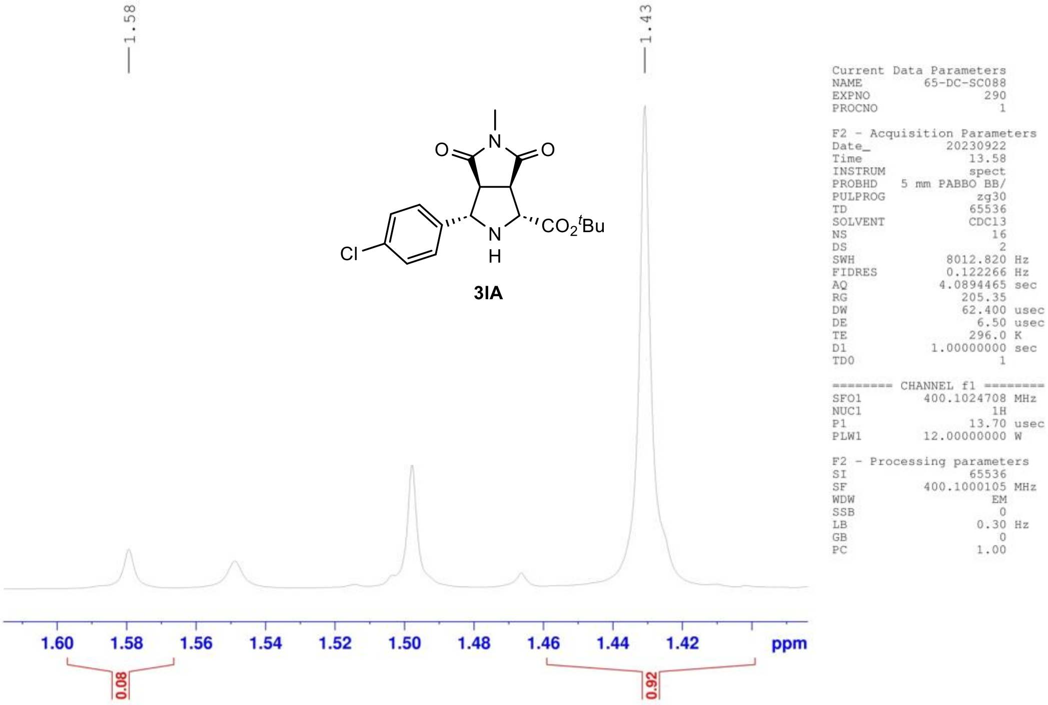
1.58
1.43
O
N
O
Cl
N
H
$CO_2{}^tBu$
3lA
Current Data Parameters
NAME 65-DC-SC088
EXPNO 290
PROCNO 1
F2 - Acquisition Parameters
Date_ 20230922
Time 13.58
INSTRUM spect
PROBHD 5 mm PABBO BB/
PULPROG zg30
TD 65536
SOLVENT CDCl3
NS 16
DS 2
SWH 8012.820 Hz
FIDRES 0.122266 Hz
AQ 4.0894465 sec
RG 205.35
DW 62.400 usec
DE 6.50 usec
TE 296.0 K
D1 1.00000000 sec
TD0 1
======== CHANNEL f1 ========
SFO1 400.1024708 MHz
NUC1 1H
P1 13.70 usec
PLW1 12.00000000 W
F2 - Processing parameters
SI 65536
SF 400.1000105 MHz
WDW EM
SSB 0
LB 0.30 Hz
GB 0
PC 1.00
1.60 1.58 1.56 1.54 1.52 1.50 1.48 1.46 1.44 1.42 ppm
0.08
0.92

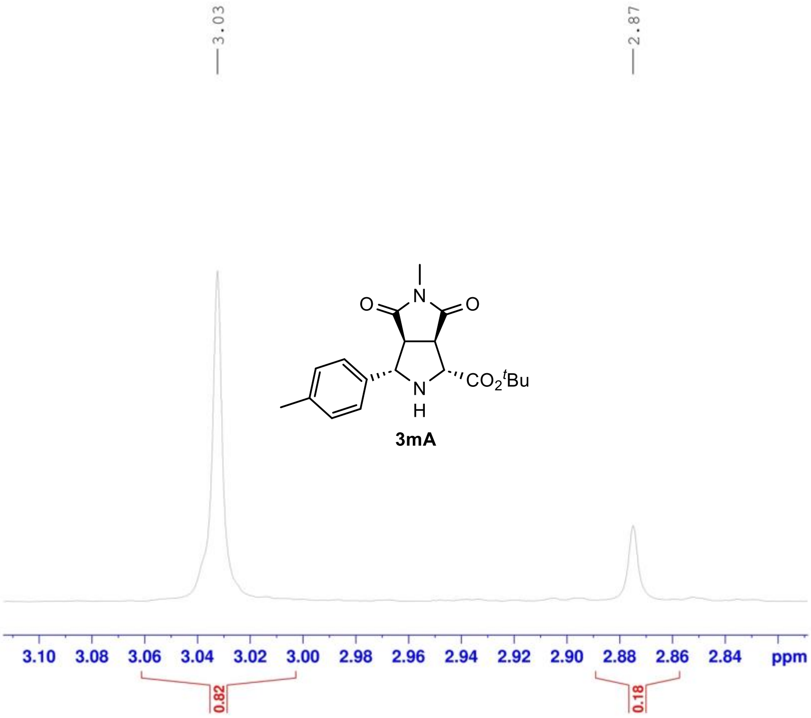


```
Current Data Parameters
NAME          65-DC-SC093
EXPNO                 350
PROCNO                  1

F2 - Acquisition Parameters
Date_            20230922
Time                14.34
INSTRUM             spect
PROBHD    5 mm PABBO BB/
PULPROG              zg30
TD                  65536
SOLVENT             CDCl3
NS                     16
DS                      2
SWH              8012.820 Hz
FIDRES           0.122266 Hz
AQ              4.0894465 sec
RG                 205.35
DW                 62.400 usec
DE                   6.50 usec
TE                  295.9 K
D1             1.00000000 sec
TD0                     1

======== CHANNEL f1 ========
SFO1         400.1024708 MHz
NUC1                   1H
P1                  13.70 usec
PLW1          12.00000000 W

F2 - Processing parameters
SI                  65536
SF           400.1000104 MHz
WDW                    EM
SSB                     0
LB                   0.30 Hz
GB                      0
PC                   1.00
```

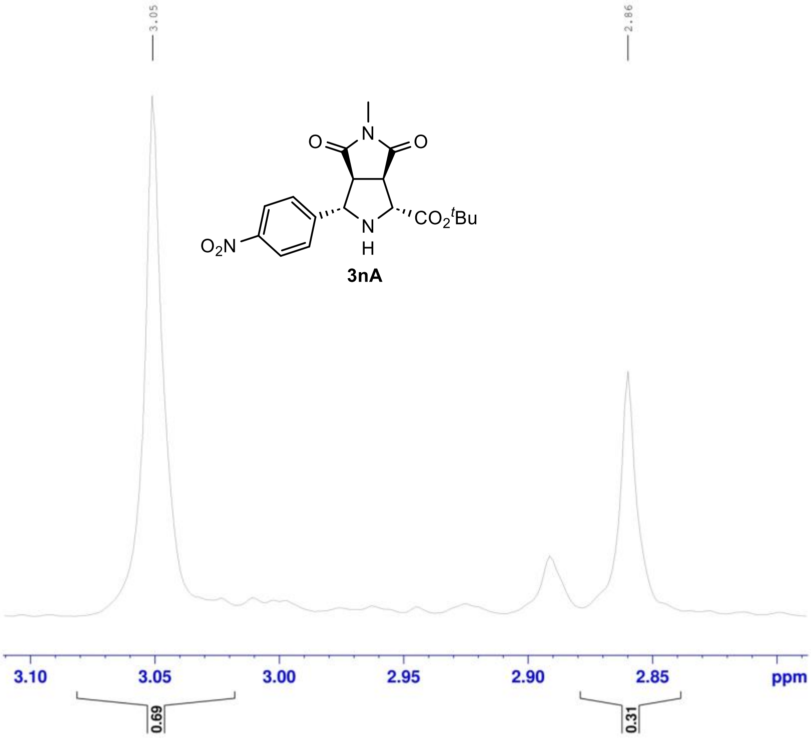


```
Current Data Parameters
NAME          65-DC-SC097
EXPNO                  50
PROCNO                  1

F2 - Acquisition Parameters
Date_            20230929
Time                10.28
INSTRUM             spect
PROBHD    5 mm PABBO BB-
PULPROG              zg30
TD                  32768
SOLVENT             CDCl3
NS                     16
DS                      2
SWH              6188.119 Hz
FIDRES           0.188846 Hz
AQ              2.6476543 sec
RG                    203
DW                 80.800 usec
DE                   8.00 usec
TE                  296.0 K
D1             1.00000000 sec
TD0                     1

======== CHANNEL f1 ========
NUC1                   1H
P1                  11.05 usec
PL1                 -2.00 dB
PL1W          37.02396774 W
SFO1         300.1318534 MHz

F2 - Processing parameters
SI                  16384
SF           300.1300061 MHz
WDW                    EM
SSB                     0
LB                   0.30 Hz
GB                      0
PC                   1.00
```

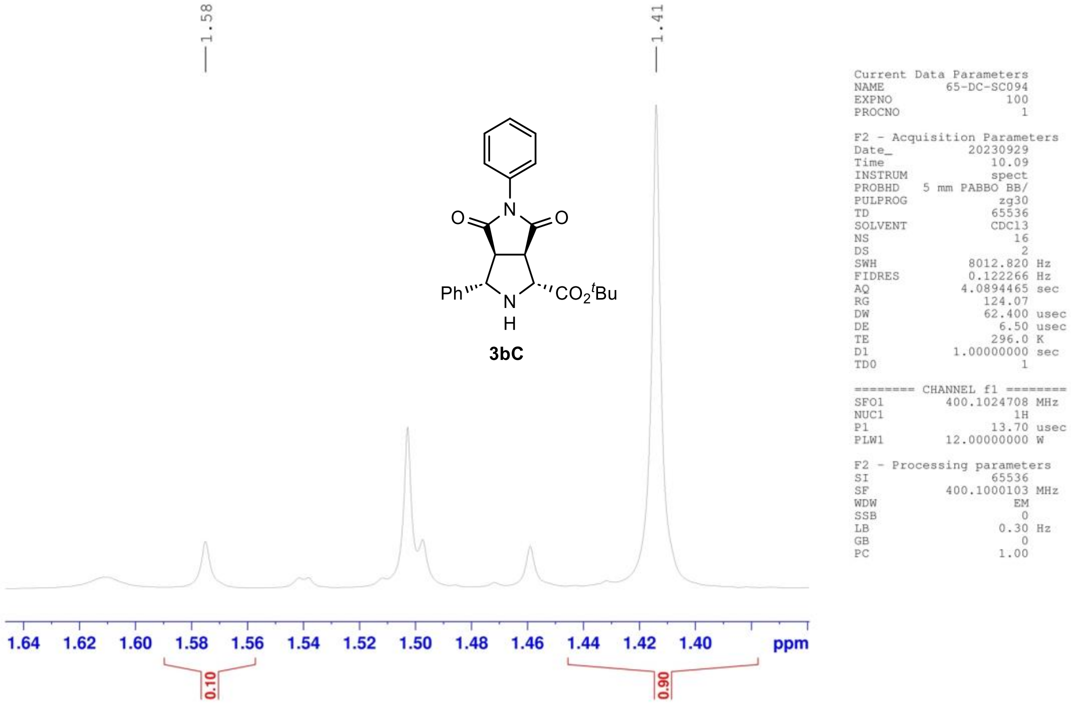
1.58
1.41
Ph
N
H
$CO_2{}^tBu$
O
O
N
3bC
Current Data Parameters
NAME 65-DC-SC094
EXPNO 100
PROCNO 1
F2 - Acquisition Parameters
Date_ 20230929
Time 10.09
INSTRUM spect
PROBHD 5 mm PABBO BB/
PULPROG zg30
TD 65536
SOLVENT CDCl3
NS 16
DS 2
SWH 8012.820 Hz
FIDRES 0.122266 Hz
AQ 4.0894465 sec
RG 124.07
DW 62.400 usec
DE 6.50 usec
TE 296.0 K
D1 1.00000000 sec
TD0 1
======== CHANNEL f1 ========
SFO1 400.1024708 MHz
NUC1 1H
P1 13.70 usec
PLW1 12.00000000 W
F2 - Processing parameters
SI 65536
SF 400.1000103 MHz
WDW EM
SSB 0
LB 0.30 Hz
GB 0
PC 1.00
1.64 1.62 1.60 1.58 1.56 1.54 1.52 1.50 1.48 1.46 1.44 1.42 1.40 ppm
0.10
0.90

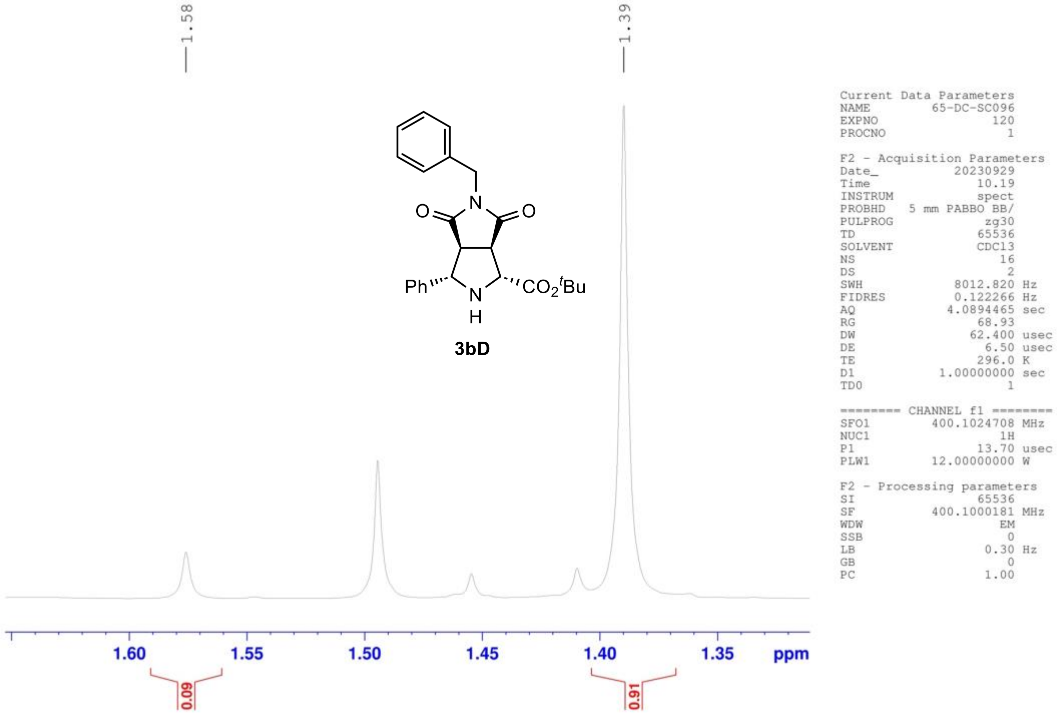
1.58
1.39
Ph
N
H
$CO_2{}^tBu$
O
O
N
3bD
Current Data Parameters
NAME 65-DC-SC096
EXPNO 120
PROCNO 1
F2 - Acquisition Parameters
Date_ 20230929
Time 10.19
INSTRUM spect
PROBHD 5 mm PABBO BB/
PULPROG zg30
TD 65536
SOLVENT CDCl3
NS 16
DS 2
SWH 8012.820 Hz
FIDRES 0.122266 Hz
AQ 4.0894465 sec
RG 68.93
DW 62.400 usec
DE 6.50 usec
TE 296.0 K
D1 1.00000000 sec
TD0 1
======== CHANNEL f1 ========
SFO1 400.1024708 MHz
NUC1 1H
P1 13.70 usec
PLW1 12.00000000 W
F2 - Processing parameters
SI 65536
SF 400.1000181 MHz
WDW EM
SSB 0
LB 0.30 Hz
GB 0
PC 1.00
1.60 1.55 1.50 1.45 1.40 1.35 ppm
0.09
0.91

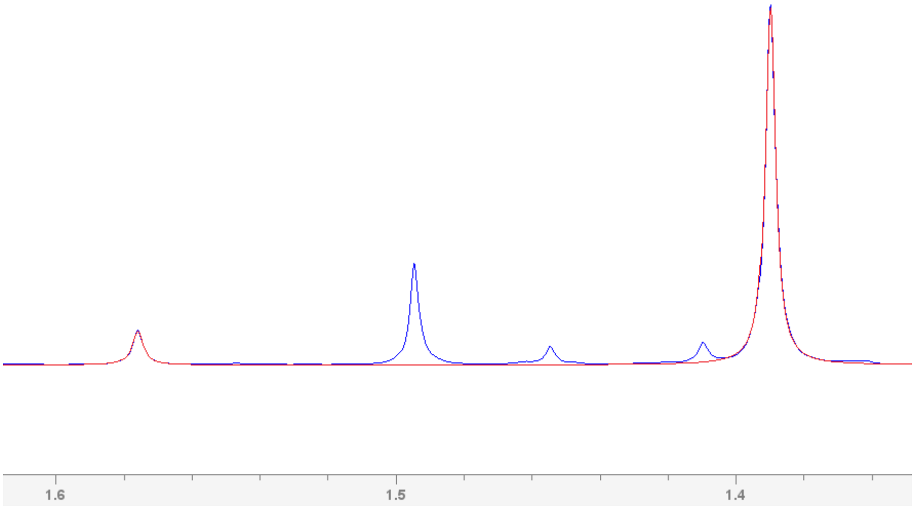

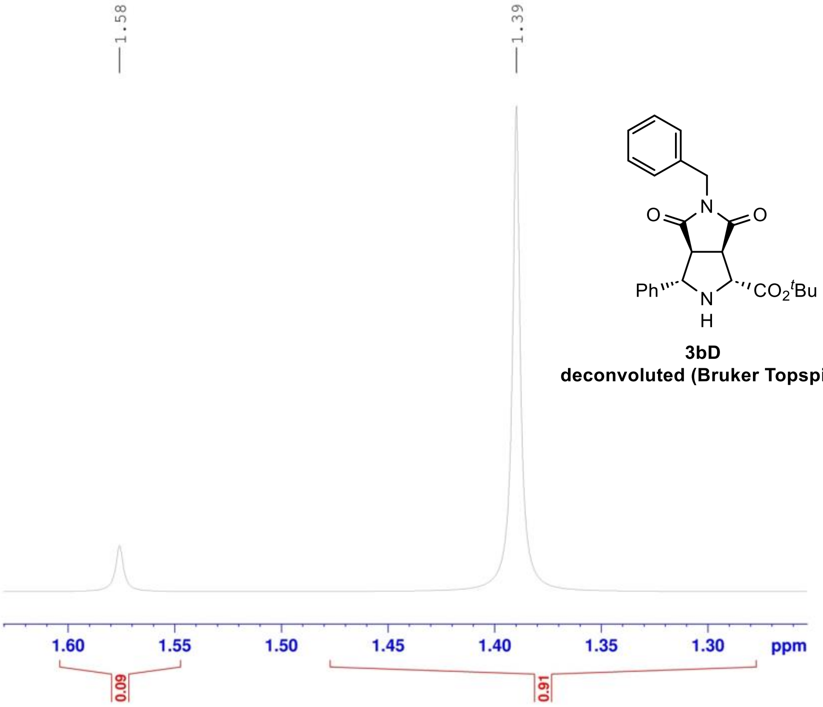

3bD
deconvoluted (Bruker Topspin)

```
Current Data Parameters
NAME          65-DC-SC096
EXPNO                 120
PROCNO                999

F2 - Acquisition Parameters
Date_            20230929
Time                10.19
INSTRUM             spect
PROBHD    5 mm PABBO BB/
PULPROG              zg30
TD                  65536
SOLVENT             CDCl3
NS                     16
DS                      2
SWH              8012.820 Hz
FIDRES           0.122266 Hz
AQ              4.0894465 sec
RG                  68.93
DW                 62.400 usec
DE                   6.50 usec
TE                  296.0 K
D1             1.00000000 sec
TD0                     1

======== CHANNEL f1 ========
SFO1        400.1024708 MHz
NUC1                   1H
P1                  13.70 usec
PLW1          12.00000000 W

F2 - Processing parameters
SI                  65536
SF          400.1000181 MHz
WDW                    EM
SSB                     0
LB                   0.30 Hz
GB                      0
PC                   1.00
```

19 References

[1] W. L. F. Armarego, C. L. L. Chai, *Purification of laboratory chemicals*, Butterworth-Heinemann, Amsterdam, Heidelberg, **2009**.

[2] F. Willig, J. Lang, A. C. Hans, M. R. Ringenberg, D. Pfeffer, W. Frey, R. Peters, *J. Am. Chem. Soc.* **2019**, *141*, 12029–12043.

[3] V. Miskov-Pajic, F. Willig, D. M. Wanner, W. Frey, R. Peters, *Angew. Chem.* **2020**, *132*, 20045–20049.

[4] D. M. Wanner, P. M. Becker, S. Suhr, N. Wannenmacher, S. Ziegler, J. Herrmann, F. Willig, J. Gabler, K. Jangid, J. Schmid et al., *Angew. Chem.* **2023**, *135*.

[5] A. C. Hans, P. M. Becker, J. Haußmann, S. Suhr, D. M. Wanner, V. Lederer, F. Willig, W. Frey, B. Sarkar, J. Kästner et al., *Angew. Chem. Int. Ed.* **2023**, *62*, e202217519.

[6] J. Schmid, W. Frey, R. Peters, *Organometallics* **2017**, *36*, 4313–4324.

[7] M. C. Walton, Y.-F. Yang, X. Hong, K. N. Houk, L. E. Overman, *Org. Lett.* **2015**, *17*, 6166–6169.

[8] M. Ayerbe, A. Arrieta, F. P. Cossío, A. Linden, *J. Org. Chem.* **1998**, *63*, 1795–1805.

[9] H. Liu, Y. Wu, Y. Zhao, Z. Li, L. Zhang, W. Yang, H. Jiang, C. Jing, H. Yu, B. Wang et al., *J. Am. Chem. Soc.* **2014**, *136*, 2625–2629.

[10] M. J. O'Donnell, W. D. Bennett, W. A. Bruder, W. N. Jacobsen, K. Knuth, B. LeClef, R. L. Polt, F. G. Bordwell, S. R. Mrozack, T. A. Cripe, *J. Am. Chem. Soc.* **1988**, *110*, 8520–8525.

[11] S. N. Greszler, G. Zhao, M. Buchman, X. B. Searle, B. Liu, E. A. Voight, *J. Org. Chem.* **2020**, *85*, 7620–7632.

[12] S. D. Sharma, S. Kanwar, S. Rajpoot, *J. Heterocycl. Chem.* **2006**, *43*, 11–19.

[13] T. OGURI, T. SHIOIRI, S. YAMADA, *Chem. Pharm. Bull.* **1977**, *25*, 2287–2291.

[14] E. E. Maroto, S. Filippone, M. Suárez, R. Martínez-Álvarez, A. de Cózar, F. P. Cossío, N. Martín, *J. Am. Chem. Soc.* **2014**, *136*, 705–712.

[15] T. Ooi, M. Takeuchi, M. Kameda, K. Maruoka, *J. Am. Chem. Soc.* **2000**, *122*, 5228–5229.

[16] N. A. Aslam, S. A. Babu, *Tetrahedron* **2014**, *70*, 6402–6419.

[17] V. A. Ioutsi, A. A. Zadorin, P. A. Khavrel, N. M. Belov, N. S. Ovchinnikova, A. A. Goryunkov, O. N. Kharybin, E. N. Nikolaev, M. A. Yurovskaya, L. N. Sidorov, *Tetrahedron* **2010**, *66*, 3037–3041.

[18] A. C. Hans, P. M. Becker, J. Haußmann, S. Suhr, D. M. Wanner, V. Lederer, F. Willig, W. Frey, B. Sarkar, J. Kästner et al., *Angew. Chem.* **2023**, *135*.

[19] J. M. Longmire, B. Wang, X. Zhang, *J. Am. Chem. Soc.* **2002**, *124*, 13400–13401.

[20] O. Tsuge, S. Kanemasa, M. Ohe, K. Yorozu, S. Takenaka, K. Ueno, *Chem. Lett.* **1986**, *15*, 1271–1274.

[21] C. Nájera, M. de Gracia Retamosa, J. M. Sansano, A. de Cózar, F. P. Cossío, *Tetrahedron: Asymmetry* **2008**, *19*, 2913–2923.
[22] S. V. Kumar, P. J. Guiry, *Angew. Chem. Int. Ed.* **2022**, *61*, e202205516.
[23] S. Yan, C. Zhang, Y.-H. Wang, Z. Cao, Z. Zheng, X.-P. Hu, *Tetrahedron Lett.* **2013**, *54*, 3669–3672.
[24] H. Chen, Q. Chen, *Chin. J. Org. Chem.* **2013**, *33*, 848.
[25] C. D.-T. Nielsen, J. Burés, *Chem. Sci.* **2019**, *10*, 348–353.
[26] J. Burés, *Angew. Chem. Int. Ed.* **2016**, *55*, 2028–2031.
[27] J. Burés, *Angew. Chem. Int. Ed.* **2016**, *55*, 16084–16087.
[28] H. Eyring, *J. Chem. Phys.* **1935**, *3*, 107–115.
[29] S.-N. Li, Q.-G. Zhai, M.-C. Hu, Y.-C. Jiang, *J. Chem. Crystallogr.* **2011**, *41*, 12–16.
[30] J. M. Abad, M. Revenga-Parra, T. García, M. Gamero, E. Lorenzo, F. Pariente, *Phys. Chem. Chem. Phys.* **2011**, *13*, 5668–5678.
[31] S. Stoll, A. Schweiger, *J. Magn. Reson.* **2006**, *178*, 42–55.
[32] a) D. F. Evans, *J. Chem. Soc.* **1959**, 2003; b) C. Piguet, *J. Chem. Educ.* **1997**, *74*, 815.
[33] G. A. Bain, J. F. Berry, *J. Chem. Educ.* **2008**, *85*, 532.
[34] H. B. Kagan, *Adv. Synth. Catal.* **2001**, *343*, 227–233.
[35] T. Satyanarayana, S. Abraham, H. B. Kagan, *Angew. Chem. Int. Ed.* **2009**, *48*, 456–494.
[36] J. Georges, *Spectrochim. Acta, Part A* **1995**, *51*, 985–994.
[37] P. Pracht, S. Grimme, C. Bannwarth, F. Bohle, S. Ehlert, G. Feldmann, J. Gorges, M. Müller, T. Neudecker, C. Plett et al., *J. Chem. Phys.* **2024**, *160*.
[38] P. Pracht, F. Bohle, S. Grimme, *Phys. Chem. Chem. Phys.* **2020**, *22*, 7169–7192.
[39] J. Kästner, J. M. Carr, T. W. Keal, W. Thiel, A. Wander, P. Sherwood, *J. Phys. Chem. A* **2009**, *113*, 11856–11865.
[40] a) P. Sherwood, A. H. de Vries, M. F. Guest, G. Schreckenbach, C. A. Catlow, S. A. French, A. A. Sokol, S. T. Bromley, W. Thiel, A. J. Turner et al., *J. Mol. Struct. THEOCHEM* **2003**, *632*, 1–28; b) S. Metz, J. Kästner, A. A. Sokol, T. W. Keal, P. Sherwood, *Wiley Comput. Mol. Sci.* **2014**, *4*, 101–110.
[41] C. Bannwarth, S. Ehlert, S. Grimme, *J. Chem. Theory Comput.* **2019**, *15*, 1652–1671.
[42] TURBOMOLE V7.5 2020, a development of University of Karlsruhe and Forschungszentrum Karlsruhe GmbH, 1989-2007, TURBOMOLE GmbH, since 2007, available from https://www.turbomole.org.
[43] S. Grimme, J. G. Brandenburg, C. Bannwarth, A. Hansen, *J. Chem. Phys.* **2015**, *143*, 54107.
[44] a) A. D. Becke, *Phys. Rev. A* **1988**, *38*, 3098–3100; b) C. Lee, W. Yang, R. G. Parr, *Phys. Rev. B* **1988**, *37*, 785–789; c) A. D. Becke, *J. Chem. Phys.* **1993**, *98*, 5648–5652;

d) P. J. Stephens, F. J. Devlin, C. F. Chabalowski, M. J. Frisch, *J. Phys. Chem.* **1994**, *98*, 11623–11627.

[45] a) S. Grimme, J. Antony, S. Ehrlich, H. Krieg, *J. Chem. Phys.* **2010**, *132*, 154104; b) S. Grimme, S. Ehrlich, L. Goerigk, *J. Comput. Chem.* **2011**, *32*, 1456–1465.

[46] a) A. Schäfer, H. Horn, R. Ahlrichs, *J. Chem. Phys.* **1992**, *97*, 2571–2577; b) A. Schäfer, C. Huber, R. Ahlrichs, *J. Chem. Phys.* **1994**, *100*, 5829–5835; c) F. Weigend, R. Ahlrichs, *Phys. Chem. Chem. Phys.* **2005**, *7*, 3297–3305; d) F. Weigend, *Phys. Chem. Chem. Phys.* **2006**, *8*, 1057–1065.

[47] A. Klamt, G. Schüürmann, *J. Chem. Soc., Perkin Trans. 2* **1993**, 799–805.

[48] A. Klamt, V. Jonas, T. Bürger, J. C. W. Lohrenz, *J. Phys. Chem. A* **1998**, *102*, 5074–5085.

[49] F. Eckert, A. Klamt, *AIChE J.* **2002**, *48*, 369–385.

[50] Gaussian 16, M. J. Frisch, G. W. Trucks, H. B. Schlegel, G. E. Scuseria, M. A. Robb, J. R. Cheeseman, G. Scalmani, V. Barone, G. A. Petersson, H. Nakatsuji, X. Li, M. Caricato, A. V. Marenich, J. Bloino, B. G. Janesko, R. Gomperts, B. Mennucci, H. P. Hratchian, J. V. Ortiz, A. F. Izmaylov, J. L. Sonnenberg, D. Williams-Young, F. Ding, F. Lipparini, F. Egidi, J. Goings, B. Peng, A. Petrone, T. Henderson, D. Ranasinghe, V. G. Zakrzewski, J. Gao, N. Rega, G. Zheng, W. Liang, M. Hada, M. Ehara, K. Toyota, R. Fukuda, J. Hasegawa, M. Ishida, T. Nakajima, Y. Honda, O. Kitao, H. Nakai, T. Vreven, K. Throssell, J. A. Montgomery, Jr., J. E. Peralta, F. Ogliaro, M. J. Bearpark, J. J. Heyd, E. N. Brothers, K. N. Kudin, V. N. Staroverov, T. A. Keith, R. Kobayashi, J. Normand, K. Raghavachari, A. P. Rendell, J. C. Burant, S. S. Iyengar, J. Tomasi, M. Cossi, J. M. Millam, M. Klene, C. Adamo, R. Cammi, J. W. Ochterski, R. L. Martin, K. Morokuma, O. Farkas, J. B. Foresman, and D. J. Fox, Gaussian, Inc., Wallingford CT, 2016.

[51] a) G. Hörner, B. Weber, *Eur. J. Inorg. Chem.* **2024**, *27*; b) S. Thies, H. Sell, C. Bornholdt, C. Schütt, F. Köhler, F. Tuczek, R. Herges, *Chem. Eur. J.* **2012**, *18*, 16358–16368; c) M. Dommaschk, C. Schütt, S. Venkataramani, U. Jana, C. Näther, F. D. Sönnichsen, R. Herges, *Dalton Trans.* **2014**, *43*, 17395–17405; d) H. Brandenburg, J. Krahmer, K. Fischer, B. Schwager, B. Flöser, C. Näther, F. Tuczek, *Eur. J. Inorg. Chem.* **2018**, *2018*, 576–585; e) F. Gutzeit, M. Dommaschk, N. Levin, A. Buchholz, E. Schaub, W. Plass, C. Näther, R. Herges, *Inorg. Chem.* **2019**, *58*, 12542–12546.

[52] a) J. P. Perdew, K. Burke, M. Ernzerhof, *Phys. Rev. Lett.* **1996**, *77*, 3865–3868; b) J. P. Perdew, K. Burke, M. Ernzerhof, *Phys. Rev. Lett.* **1997**, *78*, 1396; c) J. P. Perdew, M. Ernzerhof, K. Burke, *J. Chem. Phys.* **1996**, *105*, 9982–9985; d) C. Adamo, V. Barone, *J. Chem. Phys.* **1999**, *110*, 6158–6170.

[53] Y. Zhao, D. G. Truhlar, *Theor. Chem. Account.* **2008**, *120*, 215–241.

[54] A. Klamt, *J. Phys. Chem.* **1995**, *99*, 2224–2235.

[55] J. Heitkämper, J. Herrmann, M. Titze, S. M. Bauch, R. Peters, J. Kästner, *ACS Catal.* **2022**, *12*, 1497–1507.
